# Supplementary material for: ChromBPNet: bias factorized, base-resolution deep learning models of chromatin accessibility reveal cis-regulatory sequence syntax, transcription factor footprints and regulatory variants
Source: bioRxiv. 2025 Jan 8:2024.12.25.630221. Preprint. [Version 2] doi: 10.1101/2024.12.25.630221 (PMC11741299; doi:10.1101/2024.12.25.630221)
Supplement: Supplement 2 [file media-2.zip › supplementary_files_1/4_ATAC_tobias_corrected_bpnet/fig1j_gm12878_ATAC_tobias_corrected_softmax_bpnet_profile_modisco.pdf]

pattern	num_seqlets	cwm_fwd	cwm_rev	TOMTOM_match	TOMTOM_qval	TOMTOM_match_logo
pos_patterns.pattern_0	12552			TN5_1	5.663620e-09	
pos_patterns.pattern_1	5748			TN5_1	4.147250e-07	
pos_patterns.pattern_2	1322			SP4_HUMAN.H11MO.0.A	2.359510e-04	
pos_patterns.pattern_3	1079			NFYB_HUMAN.H11MO.0.A	4.048520e-05	
pos_patterns.pattern_4	1040			CTCF_MA0139.1	5.939280e-12	
pos_patterns.pattern_5	746			SP2_HUMAN.H11MO.0.A	1.788920e-03	
pos_patterns.pattern_6	643			ELK1_ETS_1	1.436010e-03	
pos_patterns.pattern_7	437			JUND_MOUSE.H11MO.0.A	5.345460e-07	
pos_patterns.pattern_8	434			ETS1_HUMAN.H11MO.0.A	2.060740e-03	
pos_patterns.pattern_9	362			NRF1_MA0506.1	5.675820e-06	
pos_patterns.pattern_10	288			USF2_HUMAN.H11MO.0.A	6.085520e-03	
pos_patterns.pattern_11	232			IRF4_HUMAN.H11MO.0.A	4.422980e-03	
pos_patterns.pattern_12	202			IRF4_HUMAN.H11MO.0.A	8.420850e-13	
pos_patterns.pattern_13	168			SP3_HUMAN.H11MO.0.B	3.366770e-03	
pos_patterns.pattern_14	151			SP2_HUMAN.H11MO.0.A	4.925510e-03	
pos_patterns.pattern_15	99			NFYB_HUMAN.H11MO.0.A	3.037540e-04	
pos_patterns.pattern_16	91			ELK1_ETS_4	5.428270e-04	
pos_patterns.pattern_17	57			TN5_6	6.151010e-08	
pos_patterns.pattern_18	49			ZN143_MOUSE.H11MO.0.A	1.217090e-03	
pos_patterns.pattern_19	47			NaN	NaN	
pos_patterns.pattern_20	39			Gabpa_MA0062.2	1.149790e-01	
pos_patterns.pattern_21	29			JUND_MOUSE.H11MO.0.A	4.139050e-03	
pos_patterns.pattern_22	20			TN5_1	9.834180e-01	
neg_patterns.pattern_0	2365			NFAC4_HUMAN.H11MO.0.C	1.000000e+00	
neg_patterns.pattern_1	1768			SP1_HUMAN.H11MO.0.A	1.451810e-06	
neg_patterns.pattern_2	1354			ZNF384_MA1125.1	4.172670e-03	
neg_patterns.pattern_3	991			NFATC1_NFAT_1	1.000000e+00	
neg_patterns.pattern_4	981			SP2_HUMAN.H11MO.0.A	1.182470e-07	
neg_patterns.pattern_5	945			ZFX_MOUSE.H11MO.0.B	3.481630e-04	
neg_patterns.pattern_6	864			ZFX_MOUSE.H11MO.0.B	8.140090e-04	

pattern	num_seqlets	cwm_fwd	cwm_rev	TOMTOM_match	TOMTOM_qval	TOMTOM_match_logo
neg_patterns.pattern_7	773	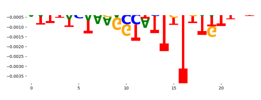	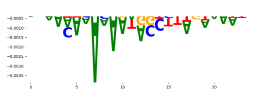	ZNF713_C2H2_1	1.000000e+00	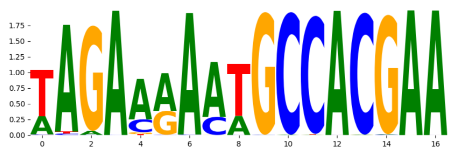
neg_patterns.pattern_8	759	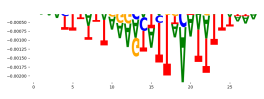	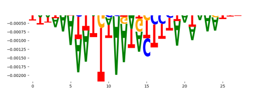	NFAC2_HUMAN.H11MO.0.B	1.000000e+00	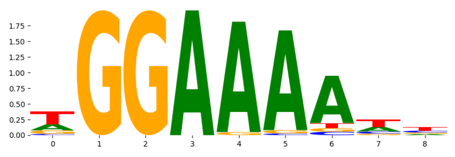
neg_patterns.pattern_9	650	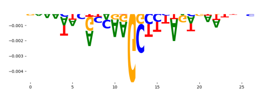	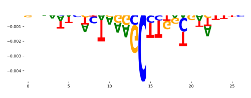	PATZ1_HUMAN.H11MO.0.C	1.924410e-03	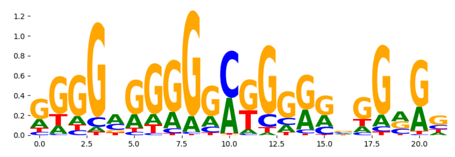
neg_patterns.pattern_10	630	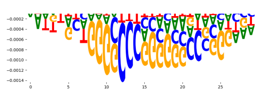	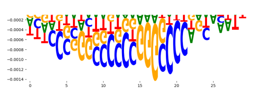	SP1_HUMAN.H11MO.0.A	2.269010e-04	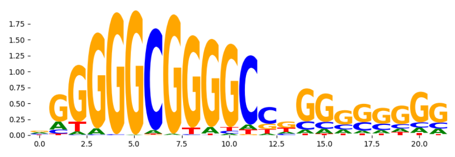
neg_patterns.pattern_11	625	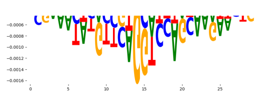	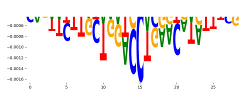	ZFX_MOUSE.H11MO.0.B	7.234320e-05	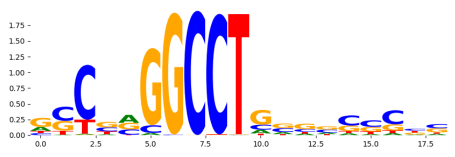
neg_patterns.pattern_12	586	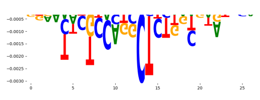	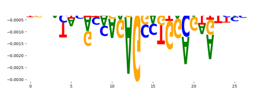	WT1_HUMAN.H11MO.0.C	1.638130e-05	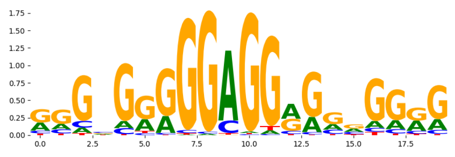
neg_patterns.pattern_13	407	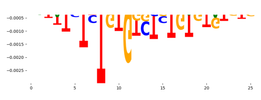	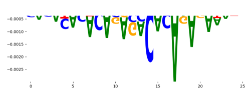	RREB1_MA0073.1	9.233300e-01	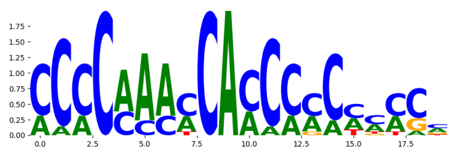
neg_patterns.pattern_14	377	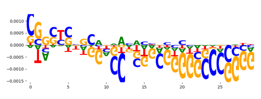	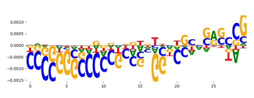	SP2_HUMAN.H11MO.0.A	8.961810e-06	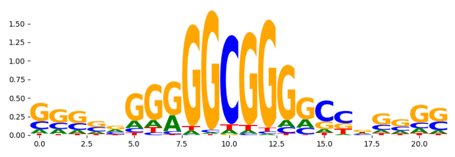
neg_patterns.pattern_15	376	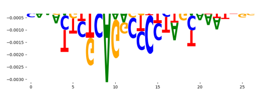	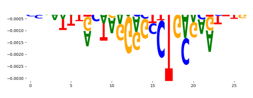	ZFX_MOUSE.H11MO.0.B	1.339560e-08	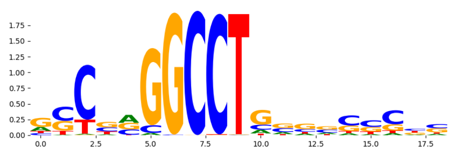
neg_patterns.pattern_16	358	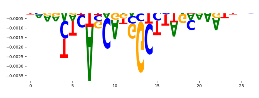	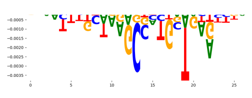	ZFX_MOUSE.H11MO.0.B	4.109220e-04	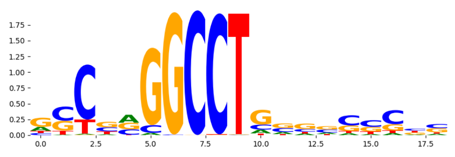
neg_patterns.pattern_17	140	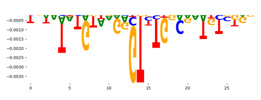	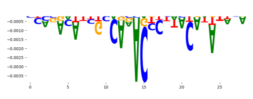	RREB1_MA0073.1	9.889390e-01	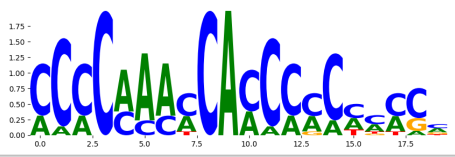
