# Supplementary material for: ChromBPNet: bias factorized, base-resolution deep learning models of chromatin accessibility reveal cis-regulatory sequence syntax, transcription factor footprints and regulatory variants
Source: bioRxiv. 2025 Jan 8:2024.12.25.630221. Preprint. [Version 2] doi: 10.1101/2024.12.25.630221 (PMC11741299; doi:10.1101/2024.12.25.630221)
Supplement: Supplement 2 [file media-2.zip › supplementary_files_1/4_ATAC_tobias_corrected_bpnet/fig1k_gm12878_ATAC_tobias_corrected_shifted_bpnet_counts_modisco.pdf]

pattern	num_seqlets	cwm_fwd	cwm_rev	TOMTOM_match	TOMTOM_qval	TOMTOM_match_logo
pos_patterns.pattern_0	3666			IRF1_MOUSE.H11MO.0.A	1.230180e-03	
pos_patterns.pattern_1	2970			BC11A_HUMAN.H11MO.0.A	2.846230e-04	
pos_patterns.pattern_2	2743			ELK1_ETS_1	7.783460e-02	
pos_patterns.pattern_3	2019			ATF3_MOUSE.H11MO.0.A	4.503730e-03	
pos_patterns.pattern_4	1920			KLF12_HUMAN.H11MO.0.C	1.621440e-05	
pos_patterns.pattern_5	1729			RELA_MA0107.1	1.783870e-04	
pos_patterns.pattern_6	1221			RUNX1_HUMAN.H11MO.0.A	2.627720e-04	
pos_patterns.pattern_7	854			IRF4_HUMAN.H11MO.0.A	2.717980e-11	
pos_patterns.pattern_8	783			NFKB2_HUMAN.H11MO.0.B	3.426330e-03	
pos_patterns.pattern_9	626			CTCF_HUMAN.H11MO.0.A	3.118780e-12	
pos_patterns.pattern_10	586			Atf1_MA0604.1	4.157550e-03	
pos_patterns.pattern_11	583			NRF1_HUMAN.H11MO.0.A	3.174730e-07	
pos_patterns.pattern_12	567			IRF5_IRF_2	4.258940e-02	
pos_patterns.pattern_13	530			NFYB_HUMAN.H11MO.0.A	2.407970e-04	
pos_patterns.pattern_14	219			THA11_HUMAN.H11MO.0.B	9.242580e-12	
pos_patterns.pattern_15	39			JUN_HUMAN.H11MO.0.A	2.541160e-01	
pos_patterns.pattern_16	32			NRF1_MA0506.1	5.971230e-01	
pos_patterns.pattern_17	27			RUNX2_MA0511.2	3.848450e-01	
neg_patterns.pattern_0	440			CTCF_MA0139.1	2.022710e-07	
neg_patterns.pattern_1	74			NFAC2_HUMAN.H11MO.0.B	1.933690e-02	
neg_patterns.pattern_2	38			SP2_HUMAN.H11MO.0.A	7.257830e-09	
