# Supplementary material for: ChromBPNet: bias factorized, base-resolution deep learning models of chromatin accessibility reveal cis-regulatory sequence syntax, transcription factor footprints and regulatory variants
Source: bioRxiv. 2025 Jan 8:2024.12.25.630221. Preprint. [Version 2] doi: 10.1101/2024.12.25.630221 (PMC11741299; doi:10.1101/2024.12.25.630221)
Supplement: Supplement 2 [file media-2.zip › supplementary_files_1/4_ATAC_tobias_corrected_bpnet/fig1l_gm12878_ATAC_tobias_corrected_shifted_bpnet_profile_modisco.pdf]

pattern	num_seqlets	cwm_fwd	cwm_rev	TOMTOM_match	TOMTOM_qval	TOMTOM_match_logo
pos_patterns.pattern_0	7247			TN5_1	5.966190e-07	
pos_patterns.pattern_1	6293			TN5_1	4.587720e-09	
pos_patterns.pattern_2	2916			CTCF_MA0139.1	3.954930e-14	
pos_patterns.pattern_3	1182			SP1_HUMAN.H11MO.0.A	2.542470e-06	
pos_patterns.pattern_4	1098			IRF1_MOUSE.H11MO.0.A	1.347470e-05	
pos_patterns.pattern_5	966			ZFX_MOUSE.H11MO.0.B	9.952670e-05	
pos_patterns.pattern_6	964			ATF3_MOUSE.H11MO.0.A	4.114900e-03	
pos_patterns.pattern_7	956			ETV4_MOUSE.H11MO.0.B	1.387850e-04	
pos_patterns.pattern_8	673			TN5_1	1.052150e-03	
pos_patterns.pattern_9	606			NFYB_HUMAN.H11MO.0.A	5.641940e-05	
pos_patterns.pattern_10	540			SPIB_MOUSE.H11MO.0.A	6.272120e-08	
pos_patterns.pattern_11	422			NFKB1_HUMAN.H11MO.1.B	4.463950e-06	
pos_patterns.pattern_12	410			SP2_HUMAN.H11MO.0.A	6.773570e-05	
pos_patterns.pattern_13	383			ATF1_MOUSE.H11MO.0.B	1.533150e-03	
pos_patterns.pattern_14	383			NRF1_HUMAN.H11MO.0.A	6.465060e-08	
pos_patterns.pattern_15	225			ZFX_MOUSE.H11MO.0.B	1.450120e-04	
pos_patterns.pattern_16	225			THA11_HUMAN.H11MO.0.B	1.142100e-07	
pos_patterns.pattern_17	210			NRF1_MA0506.1	1.000000e+00	
pos_patterns.pattern_18	140			TN5_3	7.479780e-04	
pos_patterns.pattern_19	105			ZFX_MOUSE.H11MO.0.B	2.247100e-05	
pos_patterns.pattern_20	101			SP2_HUMAN.H11MO.0.A	5.687900e-05	
pos_patterns.pattern_21	50			BACH1_HUMAN.H11MO.0.A	5.408480e-03	
pos_patterns.pattern_22	44			TF65_HUMAN.H11MO.0.A	3.422850e-04	
pos_patterns.pattern_23	34			Gabpa_MA0062.2	3.521410e-01	
pos_patterns.pattern_24	27			SP1_HUMAN.H11MO.0.A	4.457970e-04	
pos_patterns.pattern_25	20			RUNX1_MA0002.2	3.934640e-03	
