# Supplementary material for: ChromBPNet: bias factorized, base-resolution deep learning models of chromatin accessibility reveal cis-regulatory sequence syntax, transcription factor footprints and regulatory variants
Source: bioRxiv. 2025 Jan 8:2024.12.25.630221. Preprint. [Version 2] doi: 10.1101/2024.12.25.630221 (PMC11741299; doi:10.1101/2024.12.25.630221)
Supplement: Supplement 2 [file media-2.zip › supplementary_files_1/5_ATAC_bpnet_w_simple_cnn_bias/fig1n_gm12878_ATAC_raw_bpnet_w_simple_cnn_bias_profile_modisco.pdf]

pattern	num_seqlets	cwm_fwd	cwm_rev	TOMTOM_match	TOMTOM_qval	TOMTOM_match_logo
pos_patterns.pattern_0	4237			ZKSC1_HUMAN.H11MO.0.B	5.527610e-01	
pos_patterns.pattern_1	4113			CTCF_MA0139.1	1.703100e-12	
pos_patterns.pattern_2	2360			JDP2_MA0655.1	4.416050e-04	
pos_patterns.pattern_3	1775			SPIB_MOUSE.H11MO.0.A	5.722130e-07	
pos_patterns.pattern_4	1189			FOXL1_forkhead_2	7.336600e-01	
pos_patterns.pattern_5	1120			RUNX1_HUMAN.H11MO.0.A	1.091480e-06	
pos_patterns.pattern_6	1071			IRF1_MOUSE.H11MO.0.A	9.615410e-04	
pos_patterns.pattern_7	898			REL_MA0101.1	1.281250e-04	
pos_patterns.pattern_8	795			NaN	NaN	
pos_patterns.pattern_9	521			NFYB_HUMAN.H11MO.0.A	9.825340e-10	
pos_patterns.pattern_10	518			IRF1_MOUSE.H11MO.0.A	3.547410e-07	
pos_patterns.pattern_11	474			IRF4_HUMAN.H11MO.0.A	7.017240e-06	
pos_patterns.pattern_12	352			IRF1_HUMAN.H11MO.0.A	2.010720e-04	
pos_patterns.pattern_13	306			FOSL2+JUN_MA1131.1	6.691600e-05	
pos_patterns.pattern_14	176			KLF12_HUMAN.H11MO.0.C	3.817100e-04	
pos_patterns.pattern_15	120			Pou2f2.mouse_POU_2	2.084390e-03	
pos_patterns.pattern_16	116			RUNX2_MA0511.2	5.678670e-01	
pos_patterns.pattern_17	111			NRF1_MOUSE.H11MO.0.A	9.917260e-05	
pos_patterns.pattern_18	89			ELK1_MOUSE.H11MO.0.B	7.022940e-05	
pos_patterns.pattern_19	83			ZNF76_HUMAN.H11MO.0.C	1.241290e-10	
pos_patterns.pattern_20	63			PEBB_HUMAN.H11MO.0.C	3.851560e-03	
pos_patterns.pattern_21	37			Hic1.mouse_C2H2_1	4.453450e-01	
pos_patterns.pattern_22	33			TN5_8	1.000000e+00	
pos_patterns.pattern_23	30			PAX6_HUMAN.H11MO.0.C	1.253830e-01	
neg_patterns.pattern_0	26			SPIB_MOUSE.H11MO.0.A	1.395230e-01	
