# Supplementary material for: ChromBPNet: bias factorized, base-resolution deep learning models of chromatin accessibility reveal cis-regulatory sequence syntax, transcription factor footprints and regulatory variants
Source: bioRxiv. 2025 Jan 8:2024.12.25.630221. Preprint. [Version 2] doi: 10.1101/2024.12.25.630221 (PMC11741299; doi:10.1101/2024.12.25.630221)
Supplement: Supplement 2 [file media-2.zip › supplementary_files_1/3_ATAC_bpnet_w_tobias_bias/fig1g_gm12878_ATAC_raw_bpnet_w_tobias_bias_counts_modisco.pdf]

pattern	num_seqlets	cwm_fwd	cwm_rev	TOMTOM_match	TOMTOM_qval	TOMTOM_match_logo
pos_patterns.pattern_0	4566			IRF1_MOUSE.H11MO.0.A	1.760010e-04	
pos_patterns.pattern_1	4137			CTCF_MA0139.1	1.931650e-15	
pos_patterns.pattern_2	3065			ELF5_HUMAN.H11MO.0.A	2.072470e-07	
pos_patterns.pattern_3	2206			RUNX3_HUMAN.H11MO.0.A	3.068200e-03	
pos_patterns.pattern_4	2175			NFKB1_HUMAN.H11MO.1.B	5.736500e-04	
pos_patterns.pattern_5	1832			ATF3_MOUSE.H11MO.0.A	3.824140e-03	
pos_patterns.pattern_6	726			BC11A_HUMAN.H11MO.0.A	2.651010e-07	
pos_patterns.pattern_7	697			KLF12_HUMAN.H11MO.0.C	2.164240e-04	
pos_patterns.pattern_8	626			BATF_MOUSE.H11MO.0.A	8.122900e-03	
pos_patterns.pattern_9	305			NRF1_HUMAN.H11MO.0.A	1.397670e-06	
pos_patterns.pattern_10	301			NFKB2_HUMAN.H11MO.0.B	8.742870e-02	
pos_patterns.pattern_11	299			BATF_HUMAN.H11MO.0.A	4.902450e-06	
pos_patterns.pattern_12	287			IRF4_MOUSE.H11MO.0.A	1.275080e-02	
pos_patterns.pattern_13	281			CREB1_HUMAN.H11MO.0.A	6.425180e-07	
pos_patterns.pattern_14	264			NFYB_HUMAN.H11MO.0.A	1.239030e-03	
pos_patterns.pattern_15	197			IRF9_IRF_1	1.062330e-03	
pos_patterns.pattern_16	157			Pou2f2.mouse_POU_2	1.603020e-04	
pos_patterns.pattern_17	151			ZNF76_HUMAN.H11MO.0.C	9.513880e-18	
pos_patterns.pattern_18	96			PAX5_MOUSE.H11MO.0.A	8.247180e-08	
pos_patterns.pattern_19	72			HNF1A_HUMAN.H11MO.0.C	7.857330e-06	
pos_patterns.pattern_20	40			PAX2_PAX_1	5.599190e-04	
pos_patterns.pattern_21	35			ZBTB33_MA0527.1	3.479680e-04	
pos_patterns.pattern_22	29			Rfx1_MA0509.1	9.618820e-09	
neg_patterns.pattern_0	99			TF65_HUMAN.H11MO.0.A	5.033530e-01	
neg_patterns.pattern_1	53			ZN770_HUMAN.H11MO.0.C	1.642020e-01	
neg_patterns.pattern_2	48			FOXB1_forkhead_2	5.103930e-01	
neg_patterns.pattern_3	34			ZNF384_MA1125.1	4.184840e-02	
neg_patterns.pattern_4	20			Alx4.mouse_homeodomain_1	2.879690e-01	
