# Supplementary material for: ChromBPNet: bias factorized, base-resolution deep learning models of chromatin accessibility reveal cis-regulatory sequence syntax, transcription factor footprints and regulatory variants
Source: bioRxiv. 2025 Jan 8:2024.12.25.630221. Preprint. [Version 2] doi: 10.1101/2024.12.25.630221 (PMC11741299; doi:10.1101/2024.12.25.630221)

pattern	num_seqlets	cwm_fwd	cwm_rev	TOMTOM_match	TOMTOM_qval	TOMTOM_match_logo
pos_patterns.pattern_0	4348			CTCF_MA0139.1	4.109980e-14	
pos_patterns.pattern_1	3584			TN5_8	2.094890e-02	
pos_patterns.pattern_2	1461			FOSL1+JUN_MA1128.1	4.101000e-04	
pos_patterns.pattern_3	1240			TN5_8	1.000000e+00	
pos_patterns.pattern_4	1213			TN5_7	1.774580e-04	
pos_patterns.pattern_5	1115			TN5_3	8.297080e-02	
pos_patterns.pattern_6	928			SPIB_MOUSE.H11MO.0.A	8.512980e-07	
pos_patterns.pattern_7	699			IRF4_HUMAN.H11MO.0.A	1.934270e-08	
pos_patterns.pattern_8	689			RUNX2_HUMAN.H11MO.0.A	1.112250e-01	
pos_patterns.pattern_9	672			RELB_HUMAN.H11MO.0.C	8.182670e-08	
pos_patterns.pattern_10	625			TN5_3	1.388560e-01	
pos_patterns.pattern_11	616			IRF1_HUMAN.H11MO.0.A	1.789220e-09	
pos_patterns.pattern_12	523			TN5_8	4.741650e-02	
pos_patterns.pattern_13	508			TBX21_MOUSE.H11MO.0.A	1.181510e-02	
pos_patterns.pattern_14	492			Klf1_MA0493.1	6.737760e-01	
pos_patterns.pattern_15	482			MAZ_HUMAN.H11MO.0.A	6.974330e-03	
pos_patterns.pattern_16	465			IRF1_MOUSE.H11MO.0.A	1.186230e-03	
pos_patterns.pattern_17	447			TN5_6	7.206550e-04	
pos_patterns.pattern_18	290			TN5_7	4.677140e-02	
pos_patterns.pattern_19	284			MEF2B_MA0660.1	1.000000e+00	
pos_patterns.pattern_20	238			Zfp740.mouse_C2H2_1	6.316440e-02	
pos_patterns.pattern_21	210			NFYB_HUMAN.H11MO.0.A	2.563190e-07	
pos_patterns.pattern_22	113			ZN490_HUMAN.H11MO.0.C	1.000000e+00	
pos_patterns.pattern_23	45			RUNX1_HUMAN.H11MO.0.A	2.950580e-01	
pos_patterns.pattern_24	37			RUNX2_RUNX_2	4.670560e-01	
pos_patterns.pattern_25	36			ZN143_MOUSE.H11MO.0.A	7.631770e-09	
pos_patterns.pattern_26	31			MEF2D_MA0773.1	1.000000e+00	
pos_patterns.pattern_27	23			RUNX3_MOUSE.H11MO.0.A	3.420230e-01	
pos_patterns.pattern_28	21			SP1_MA0079.3	1.409690e-03	

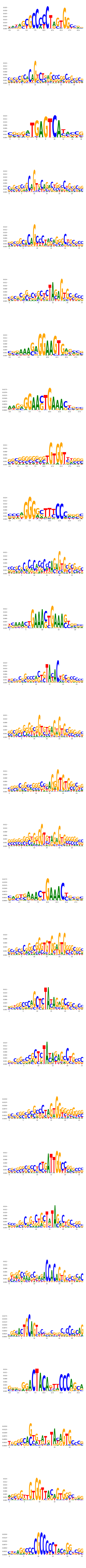
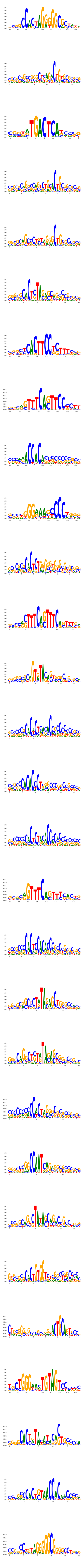
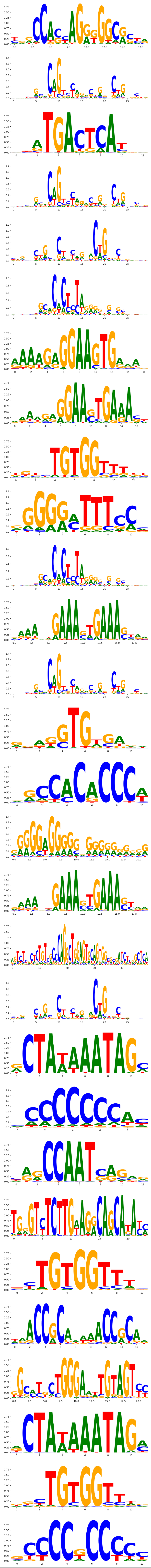

Supplement: Supplement 2 [file media-2.zip › supplementary_files_1/3_ATAC_bpnet_w_tobias_bias/fig1h_gm12878_ATAC_raw_bpnet_w_tobias_bias_profile_modisco.pdf]
