# Supplementary material for: ChromBPNet: bias factorized, base-resolution deep learning models of chromatin accessibility reveal cis-regulatory sequence syntax, transcription factor footprints and regulatory variants
Source: bioRxiv. 2025 Jan 8:2024.12.25.630221. Preprint. [Version 2] doi: 10.1101/2024.12.25.630221 (PMC11741299; doi:10.1101/2024.12.25.630221)

pattern	num_seqlets	cwm_fwd	cwm_rev	TOMTOM_match	TOMTOM_qval	TOMTOM_match_logo
pos_patterns.pattern_0	7085			TN5_1	2.170570e-09	
pos_patterns.pattern_1	4939			TN5_2	1.456940e-02	
pos_patterns.pattern_2	3628			CTCF_MA0139.1	3.028070e-14	
pos_patterns.pattern_3	1634			TN5_3	1.245300e-12	
pos_patterns.pattern_4	897			KLF4_MA0039.3	1.000000e+00	
pos_patterns.pattern_5	856			BATF+JUN_MA0462.1	1.740240e-06	
pos_patterns.pattern_6	673			IRF1_MOUSE.H11MO.0.A	1.321160e-05	
pos_patterns.pattern_7	651			TN5_4	3.105130e-04	
pos_patterns.pattern_8	632			TN5_4	8.225450e-04	
pos_patterns.pattern_9	577			SPIB_MOUSE.H11MO.0.A	1.448930e-04	
pos_patterns.pattern_10	427			IRF4_HUMAN.H11MO.0.A	7.396960e-07	
pos_patterns.pattern_11	417			TN5_4	1.145880e-01	
pos_patterns.pattern_12	385			TN5_6	2.160850e-18	
pos_patterns.pattern_13	195			NFKB1_HUMAN.H11MO.1.B	9.020920e-06	
pos_patterns.pattern_14	95			NFKB2_HUMAN.H11MO.0.B	2.098310e-04	
pos_patterns.pattern_15	90			RUNX2_MA0511.2	1.000000e+00	
pos_patterns.pattern_16	88			TN5_7	1.235670e-06	
pos_patterns.pattern_17	67			TN5_6	5.583200e-06	
pos_patterns.pattern_18	46			NFKB1_HUMAN.H11MO.1.B	3.280860e-04	
pos_patterns.pattern_19	24			NFKB1_MA0105.4	2.501510e-04	
pos_patterns.pattern_20	20			STAT1+STAT2_MA0517.1	1.265250e-01	

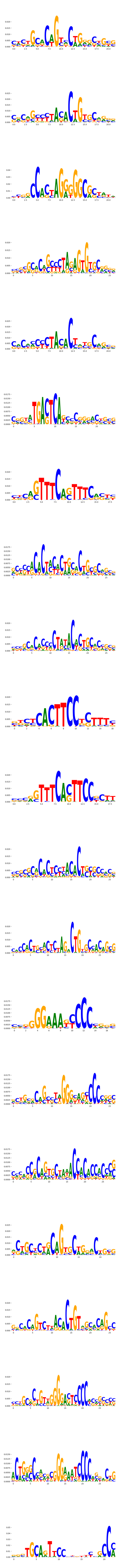

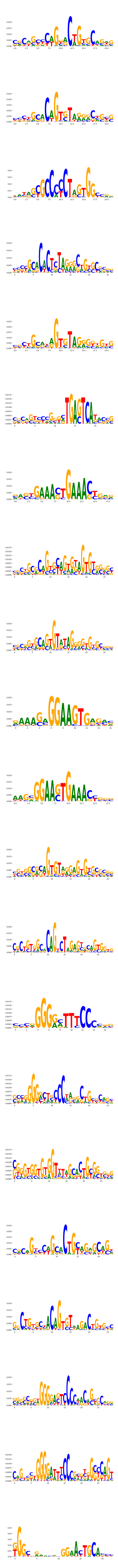

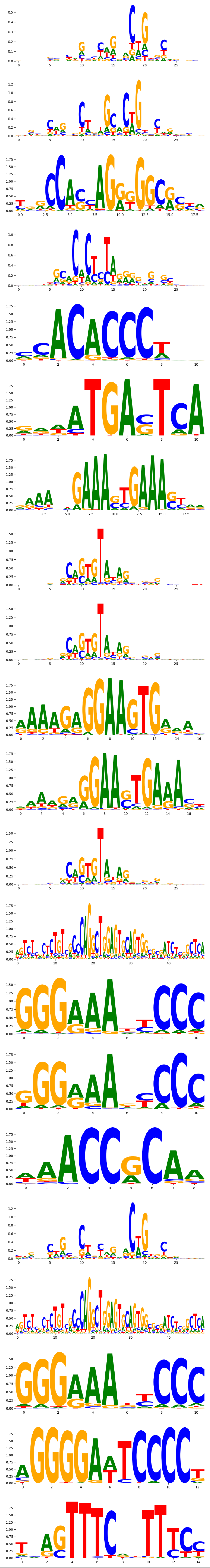

Supplement: Supplement 2 [file media-2.zip › supplementary_files_1/1_ATAC_raw_bpnet_uncorrected/fig1d_gm12878_ATAC_raw_bpnet_uncorrected_profile_modisco.pdf]
