# Supplementary material for: ChromBPNet: bias factorized, base-resolution deep learning models of chromatin accessibility reveal cis-regulatory sequence syntax, transcription factor footprints and regulatory variants
Source: bioRxiv. 2025 Jan 8:2024.12.25.630221. Preprint. [Version 2] doi: 10.1101/2024.12.25.630221 (PMC11741299; doi:10.1101/2024.12.25.630221)
Supplement: Supplement 2 [file media-2.zip › supplementary_files_1/1_ATAC_raw_bpnet_uncorrected/fig1a_k562_ATAC_raw_bpnet_uncorrected_counts_modisco.pdf]

pattern	num_seqlets	cwm_fwd	cwm_rev	TOMTOM_match	TOMTOM_qval	TOMTOM_match_logo
pos_patterns.pattern_0	3831			GATA4_HUMAN.H11MO.0.A	9.627200e-03	
pos_patterns.pattern_1	3616			KLF12_HUMAN.H11MO.0.C	2.789230e-05	
pos_patterns.pattern_2	1654			BACH2_MOUSE.H11MO.0.A	2.755450e-04	
pos_patterns.pattern_3	1639			CTCF_MOUSE.H11MO.0.A	4.015900e-16	
pos_patterns.pattern_4	1513			ELK1_ETS_1	1.183960e-01	
pos_patterns.pattern_5	700			NFYB_HUMAN.H11MO.0.A	9.192150e-03	
pos_patterns.pattern_6	518			NRF1_MOUSE.H11MO.0.A	6.193500e-07	
pos_patterns.pattern_7	447			NRF1_NRF_1	7.521700e-03	
pos_patterns.pattern_8	422			FOSL2+JUN_MA1131.1	1.396980e-04	
pos_patterns.pattern_9	376			TAF1_HUMAN.H11MO.0.A	1.155700e-01	
pos_patterns.pattern_10	318			SPIB_MOUSE.H11MO.0.A	9.220600e-04	
pos_patterns.pattern_11	282			E2F2_HUMAN.H11MO.0.B	1.321570e-01	
pos_patterns.pattern_12	274			ATF4_MOUSE.H11MO.0.A	9.997960e-06	
pos_patterns.pattern_13	239			NFIC_HUMAN.H11MO.0.A	2.630340e-07	
pos_patterns.pattern_14	204			ZN143_MOUSE.H11MO.0.A	5.362850e-20	
pos_patterns.pattern_15	169			SP2_HUMAN.H11MO.0.A	8.109120e-04	
pos_patterns.pattern_16	144			NRF1_MA0506.1	8.529800e-02	
pos_patterns.pattern_17	135			TYY1_HUMAN.H11MO.0.A	7.331310e-03	
pos_patterns.pattern_18	78			STAT1_HUMAN.H11MO.0.A	1.615170e-02	
pos_patterns.pattern_19	75			ETV6_ETS_1	5.495850e-05	
pos_patterns.pattern_20	40			ZNF76_HUMAN.H11MO.0.C	4.157780e-13	
pos_patterns.pattern_21	38			SRY_MA0084.1	1.000000e+00	
pos_patterns.pattern_22	37			FLI1_ETS_3	2.185180e-01	
pos_patterns.pattern_23	36			KLF5_MA0599.1	5.070640e-03	
pos_patterns.pattern_24	27			NaN	NaN	
pos_patterns.pattern_25	21			NaN	NaN	
neg_patterns.pattern_0	1188			CTCF_MA0139.1	1.172910e-08	
neg_patterns.pattern_1	171			HIC2_C2H2_1	9.044890e-01	
neg_patterns.pattern_2	170			REST_MA0138.2	2.159730e-11	
neg_patterns.pattern_3	125			NFYA_MA0060.3	1.558440e-02	

pattern	num_seqlets	cwm_fwd	cwm_rev	TOMTOM_match	TOMTOM_qval	TOMTOM_match_logo
neg_patterns.pattern_4	60	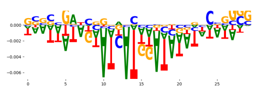	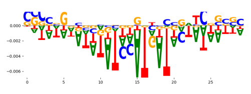	Z354A_HUMAN.H11MO.0.C	1.083500e-01	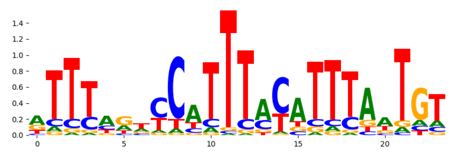
neg_patterns.pattern_5	57	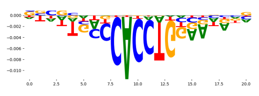	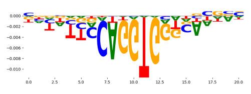	SNAI1_HUMAN.H11MO.0.C	1.380430e-03	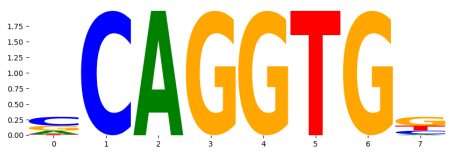
neg_patterns.pattern_6	36	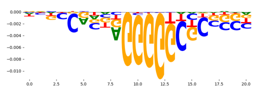	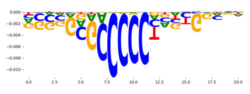	CTCFL_MOUSE.H11MO.0.A	4.582550e-05	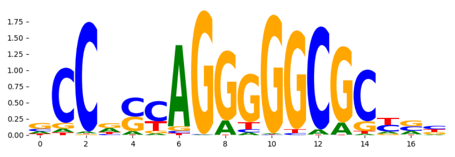
neg_patterns.pattern_7	31	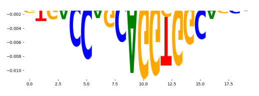	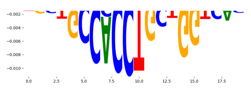	CTCFL_HUMAN.H11MO.0.A	7.590780e-04	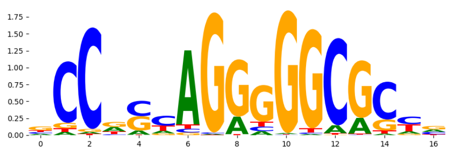
neg_patterns.pattern_8	29	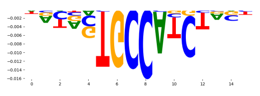	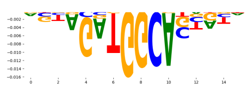	NEUROD1_MA1109.1	5.070610e-01	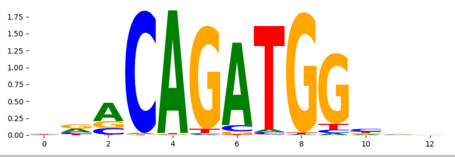
