# Supplementary material for: ChromBPNet: bias factorized, base-resolution deep learning models of chromatin accessibility reveal cis-regulatory sequence syntax, transcription factor footprints and regulatory variants
Source: bioRxiv. 2025 Jan 8:2024.12.25.630221. Preprint. [Version 2] doi: 10.1101/2024.12.25.630221 (PMC11741299; doi:10.1101/2024.12.25.630221)
Supplement: Supplement 2 [file media-2.zip › supplementary_files_1/1_ATAC_raw_bpnet_uncorrected/fig1c_gm12878_ATAC_raw_bpnet_uncorrected_counts_modisco.pdf]

pattern	num_seqlets	cwm_fwd	cwm_rev	TOMTOM_match	TOMTOM_qval	TOMTOM_match_logo
pos_patterns.pattern_0	3928			IRF1_MOUSE.H11MO.0.A	3.923590e-03	
pos_patterns.pattern_1	3289			ELF5_HUMAN.H11MO.0.A	7.641830e-04	
pos_patterns.pattern_2	2562			NFKB1_HUMAN.H11MO.1.B	8.201100e-05	
pos_patterns.pattern_3	2267			FOS_HUMAN.H11MO.0.A	2.146600e-03	
pos_patterns.pattern_4	2039			RUNX1_HUMAN.H11MO.0.A	1.367720e-03	
pos_patterns.pattern_5	1466			ELK1_ETS_1	9.781250e-02	
pos_patterns.pattern_6	1071			KLF12_HUMAN.H11MO.0.C	1.145100e-04	
pos_patterns.pattern_7	1029			CTCF_HUMAN.H11MO.0.A	6.543820e-13	
pos_patterns.pattern_8	563			NFYA_MA0060.3	4.821560e-01	
pos_patterns.pattern_9	554			Atf3_MA0605.1	2.034030e-02	
pos_patterns.pattern_10	462			NRF1_HUMAN.H11MO.0.A	1.946740e-07	
pos_patterns.pattern_11	364			Pou2f2.mouse_POU_2	7.033640e-03	
pos_patterns.pattern_12	346			EBF1_EBF_1	2.177400e-05	
pos_patterns.pattern_13	200			MEF2D_MOUSE.H11MO.0.A	4.565780e-07	
pos_patterns.pattern_14	185			PAX5_MOUSE.H11MO.0.A	2.481870e-14	
pos_patterns.pattern_15	168			HNF1B_HUMAN.H11MO.0.A	3.538430e-06	
pos_patterns.pattern_16	160			SPI1_ETS_1	1.479060e-02	
pos_patterns.pattern_17	151			JUND_MA0492.1	3.078890e-03	
pos_patterns.pattern_18	136			KAISO_HUMAN.H11MO.0.A	2.645110e-03	
pos_patterns.pattern_19	132			TYY1_HUMAN.H11MO.0.A	5.620560e-06	
pos_patterns.pattern_20	130			PAX1_MA0779.1	2.194780e-06	
pos_patterns.pattern_21	125			ZN143_HUMAN.H11MO.0.A	5.216290e-10	
pos_patterns.pattern_22	50			FOSL1_MA0477.1	4.760960e-03	
pos_patterns.pattern_23	39			RFX2_HUMAN.H11MO.0.A	2.563000e-09	
pos_patterns.pattern_24	31			ZNF76_HUMAN.H11MO.0.C	1.010970e-10	
pos_patterns.pattern_25	26			Rfx3.mouse_RFX_1	3.691570e-02	
pos_patterns.pattern_26	22			IRF7_HUMAN.H11MO.0.C	2.195690e-03	
pos_patterns.pattern_27	20			ZNF76_HUMAN.H11MO.0.C	3.744990e-09	
neg_patterns.pattern_0	116			NFATC2_MA0152.1	7.336560e-01	

pattern	num_seqlets	cwm_fwd	cwm_rev	TOMTOM_match	TOMTOM_qval	TOMTOM_match_logo
neg_patterns.pattern_1	69	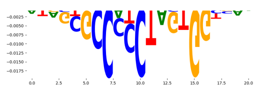	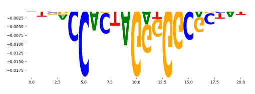	CTCF_MA0139.1	4.306850e-07	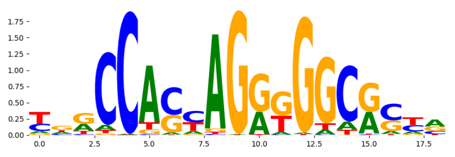
neg_patterns.pattern_2	46	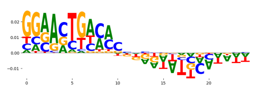	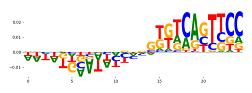	STAT1+STAT2_MA0517.1	6.559600e-03	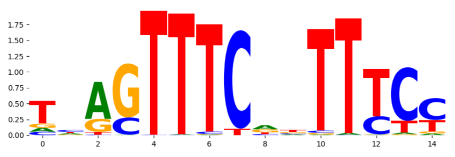
neg_patterns.pattern_3	29	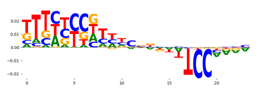	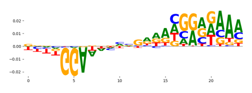	NFATC3_MA0625.1	1.162920e-01	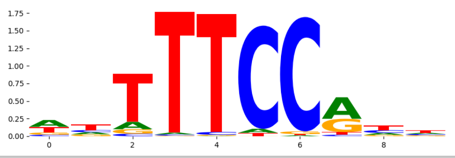
