# Supplementary material for: ChromBPNet: bias factorized, base-resolution deep learning models of chromatin accessibility reveal cis-regulatory sequence syntax, transcription factor footprints and regulatory variants
Source: bioRxiv. 2025 Jan 8:2024.12.25.630221. Preprint. [Version 2] doi: 10.1101/2024.12.25.630221 (PMC11741299; doi:10.1101/2024.12.25.630221)
Supplement: Supplement 2 [file media-2.zip › supplementary_files_1/1_ATAC_raw_bpnet_uncorrected/fig1b_k562_ATAC_raw_bpnet_uncorrected_profile_modisco.pdf]

pattern	num_seqlets	cwm_fwd	cwm_rev	TOMTOM_match	TOMTOM_qval	TOMTOM_match_logo
pos_patterns.pattern_0	6900			TN5_2	6.560760e-08	
pos_patterns.pattern_1	5483			TN5_2	2.815270e-04	
pos_patterns.pattern_2	4514			CTCF_MA0139.1	5.112140e-16	
pos_patterns.pattern_3	845			Bach1+Mafk_MA0591.1	2.250170e-06	
pos_patterns.pattern_4	473			TN5_3	2.936680e-08	
pos_patterns.pattern_5	382			TN5_6	1.881390e-20	
pos_patterns.pattern_6	380			SP2_HUMAN.H11MO.0.A	2.601250e-08	
pos_patterns.pattern_7	375			SP5_MOUSE.H11MO.0.C	2.782670e-03	
pos_patterns.pattern_8	286			SP2_HUMAN.H11MO.0.A	2.561090e-06	
pos_patterns.pattern_9	285			NFYC_HUMAN.H11MO.0.A	1.534090e-04	
pos_patterns.pattern_10	243			KLF5_MA0599.1	1.671040e-05	
pos_patterns.pattern_11	194			TAL1_HUMAN.H11MO.0.A	4.687400e-08	
pos_patterns.pattern_12	131			KLF5_MA0599.1	1.147150e-02	
pos_patterns.pattern_13	119			KLF9_MA1107.1	6.118750e-04	
pos_patterns.pattern_14	114			NFYA_MA0060.3	1.603150e-07	
pos_patterns.pattern_15	90			ZN143_MOUSE.H11MO.0.A	1.170090e-10	
pos_patterns.pattern_16	74			ATF4_MOUSE.H11MO.0.A	1.631440e-06	
pos_patterns.pattern_17	69			REST_HUMAN.H11MO.0.A	4.836510e-12	
pos_patterns.pattern_18	61			GATA3_GATA_2	2.123370e-03	
pos_patterns.pattern_19	31			KLF4_HUMAN.H11MO.0.A	5.955100e-04	
pos_patterns.pattern_20	30			TN5_1	3.216320e-01	
pos_patterns.pattern_21	30			NRF1_MA0506.1	4.268540e-04	
pos_patterns.pattern_22	30			Gata1_MA0035.3	2.141770e-02	
