# Supplementary material for: ChromBPNet: bias factorized, base-resolution deep learning models of chromatin accessibility reveal cis-regulatory sequence syntax, transcription factor footprints and regulatory variants
Source: bioRxiv. 2025 Jan 8:2024.12.25.630221. Preprint. [Version 2] doi: 10.1101/2024.12.25.630221 (PMC11741299; doi:10.1101/2024.12.25.630221)
Supplement: Supplement 2 [file media-2.zip › supplementary_files_1/2_ATAC_hint_corrected_bpnet/fig1f_gm12878_ATAC_hint_corrected_bpnet_profile_modisco.pdf]

pattern	num_seqlets	cwm_fwd	cwm_rev	TOMTOM_match	TOMTOM_qval	TOMTOM_match_logo
pos_patterns.pattern_0	9994			NFIB_NFI_1	1.000000e+00	
pos_patterns.pattern_1	3447			CTCF_MA0139.1	7.712760e-15	
pos_patterns.pattern_2	2589			MSC_MA0665.1	2.364090e-02	
pos_patterns.pattern_3	1666			IRF1_MOUSE.H11MO.0.A	6.778660e-05	
pos_patterns.pattern_4	942			FOSL1+JUN_MA1128.1	3.929150e-04	
pos_patterns.pattern_5	841			SPIB_MOUSE.H11MO.0.A	5.683400e-05	
pos_patterns.pattern_6	713			TBX1_TBX_4	7.178580e-01	
pos_patterns.pattern_7	581			TF65_MOUSE.H11MO.0.A	7.645550e-01	
pos_patterns.pattern_8	555			TF65_HUMAN.H11MO.0.A	8.829450e-05	
pos_patterns.pattern_9	405			STAT1_MOUSE.H11MO.0.A	3.632700e-01	
pos_patterns.pattern_10	343			GLI2_C2H2_2	5.547620e-01	
pos_patterns.pattern_11	219			IRF1_MA0050.2	2.263410e-02	
pos_patterns.pattern_12	197			KLF3_HUMAN.H11MO.0.B	2.732320e-04	
pos_patterns.pattern_13	157			NFYB_HUMAN.H11MO.0.A	1.703180e-09	
pos_patterns.pattern_14	152			MESP1_bHLH_1	6.930440e-01	
pos_patterns.pattern_15	68			GABPA_HUMAN.H11MO.0.A	1.668740e-01	
pos_patterns.pattern_16	64			BATF_HUMAN.H11MO.0.A	5.450350e-05	
pos_patterns.pattern_17	56			TN5_1	1.686230e-02	
pos_patterns.pattern_18	55			ZNF76_HUMAN.H11MO.0.C	4.258900e-08	
pos_patterns.pattern_19	27			FOXI1_HUMAN.H11MO.0.B	6.648740e-02	
