# Supplementary material for: ChromBPNet: bias factorized, base-resolution deep learning models of chromatin accessibility reveal cis-regulatory sequence syntax, transcription factor footprints and regulatory variants
Source: bioRxiv. 2025 Jan 8:2024.12.25.630221. Preprint. [Version 2] doi: 10.1101/2024.12.25.630221 (PMC11741299; doi:10.1101/2024.12.25.630221)
Supplement: Supplement 2 [file media-2.zip › supplementary_files_1/2_ATAC_hint_corrected_bpnet/fig1e_gm12878_ATAC_hint_corrected_bpnet_counts_modisco.pdf]

pattern	num_seqlets	cwm_fwd	cwm_rev	TOMTOM_match	TOMTOM_qval	TOMTOM_match_logo
pos_patterns.pattern_0	3860			IRF1_MOUSE.H11MO.0.A	4.563880e-03	
pos_patterns.pattern_1	3463			SPIB_MOUSE.H11MO.0.A	2.153090e-06	
pos_patterns.pattern_2	2344			FOSL2+JUN_MA1130.1	1.388970e-03	
pos_patterns.pattern_3	1831			RUNX3_HUMAN.H11MO.0.A	2.386630e-03	
pos_patterns.pattern_4	1530			Elk3.mouse_ETS_1	1.035640e-04	
pos_patterns.pattern_5	1515			TF65_MOUSE.H11MO.0.A	1.629520e-05	
pos_patterns.pattern_6	1381			CTCF_MA0139.1	3.875760e-16	
pos_patterns.pattern_7	1336			KLF12_HUMAN.H11MO.0.C	5.353910e-04	
pos_patterns.pattern_8	660			NFKB2_HUMAN.H11MO.0.B	5.708390e-04	
pos_patterns.pattern_9	523			ATF1_HUMAN.H11MO.0.B	1.046210e-04	
pos_patterns.pattern_10	519			NFYB_HUMAN.H11MO.0.A	1.056160e-02	
pos_patterns.pattern_11	484			BATF3_HUMAN.H11MO.0.B	2.836750e-08	
pos_patterns.pattern_12	430			NRF1_MOUSE.H11MO.0.A	1.867060e-06	
pos_patterns.pattern_13	353			POU5F1_MA1115.1	3.183180e-03	
pos_patterns.pattern_14	307			EBF1_EBF_1	1.949320e-05	
pos_patterns.pattern_15	161			SPI1_ETS_1	2.228180e-02	
pos_patterns.pattern_16	148			TYY1_HUMAN.H11MO.0.A	1.164150e-07	
pos_patterns.pattern_17	144			ZBTB33_MA0527.1	5.212800e-02	
pos_patterns.pattern_18	120			HNF1B_MOUSE.H11MO.0.A	8.984210e-06	
pos_patterns.pattern_19	110			ZNF76_HUMAN.H11MO.0.C	1.296790e-20	
pos_patterns.pattern_20	55			MEF2D_HUMAN.H11MO.0.A	1.008160e-03	
pos_patterns.pattern_21	50			FOXJ3_HUMAN.H11MO.0.A	2.261780e-01	
pos_patterns.pattern_22	45			THA11_MOUSE.H11MO.0.B	5.394040e-11	
pos_patterns.pattern_23	39			PAX2_PAX_1	9.979950e-07	
pos_patterns.pattern_24	31			MEF2B_MA0660.1	1.470570e-04	
pos_patterns.pattern_25	30			JUN_MA0489.1	3.706570e-03	
neg_patterns.pattern_0	127			TEAD1_MOUSE.H11MO.0.A	5.340050e-01	
