# Supplementary material for: ChromBPNet: bias factorized, base-resolution deep learning models of chromatin accessibility reveal cis-regulatory sequence syntax, transcription factor footprints and regulatory variants
Source: bioRxiv. 2025 Jan 8:2024.12.25.630221. Preprint. [Version 2] doi: 10.1101/2024.12.25.630221 (PMC11741299; doi:10.1101/2024.12.25.630221)
Supplement: Supplement 2 [file media-2.zip › supplementary_files_1/6_ATAC_chrombpnet/fig1p_gm12878_ATAC_raw_chrombpnet_profile_modisco.pdf]

pattern	num_seqlets	cwm_fwd	cwm_rev	TOMTOM_match	TOMTOM_qval	TOMTOM_match_logo
pos_patterns.pattern_0	4606			CTCF_MA0139.1	3.632500e-14	
pos_patterns.pattern_1	3246			STAT1+STAT2_MA0517.1	1.433520e-01	
pos_patterns.pattern_2	2725			JDP2_MA0655.1	2.768140e-03	
pos_patterns.pattern_3	2423			BC11A_HUMAN.H11MO.0.A	6.951580e-05	
pos_patterns.pattern_4	2254			RUNX1_HUMAN.H11MO.0.A	5.247210e-02	
pos_patterns.pattern_5	2050			TF65_HUMAN.H11MO.0.A	1.136670e-05	
pos_patterns.pattern_6	1980			IRF8_HUMAN.H11MO.0.B	6.938850e-06	
pos_patterns.pattern_7	1632			KLF12_HUMAN.H11MO.0.C	4.112910e-05	
pos_patterns.pattern_8	885			NFYB_HUMAN.H11MO.0.A	3.936070e-04	
pos_patterns.pattern_9	775			Gabpa_MA0062.2	6.718830e-05	
pos_patterns.pattern_10	590			NRF1_MA0506.1	1.852890e-08	
pos_patterns.pattern_11	453			POU5F1_MA1115.1	4.135300e-03	
pos_patterns.pattern_12	409			BATF_HUMAN.H11MO.0.A	7.153110e-08	
pos_patterns.pattern_13	336			FOSL2+JUN_MA1131.1	1.302120e-04	
pos_patterns.pattern_14	268			TYY1_MOUSE.H11MO.0.A	8.967910e-07	
pos_patterns.pattern_15	259			JUN_MA0488.1	4.398200e-03	
pos_patterns.pattern_16	244			IRF9_IRF_1	4.245900e-04	
pos_patterns.pattern_17	243			ZN143_MOUSE.H11MO.0.A	2.222570e-18	
pos_patterns.pattern_18	160			MITF_HUMAN.H11MO.0.A	5.099030e-05	
pos_patterns.pattern_19	138			HNF1A_HUMAN.H11MO.0.C	7.704190e-05	
pos_patterns.pattern_20	118			SPI1_ETS_1	1.635400e-02	
pos_patterns.pattern_21	113			RELB_MA1117.1	7.435450e-01	
pos_patterns.pattern_22	108			ATF3_MOUSE.H11MO.0.A	3.099670e-01	
pos_patterns.pattern_23	85			PAX2_PAX_1	7.215390e-07	
pos_patterns.pattern_24	51			Hic1.mouse_C2H2_1	7.661810e-01	
pos_patterns.pattern_25	43			PAX6_PAX_1	1.275950e-04	
pos_patterns.pattern_26	42			Rfx1_MA0509.1	8.271380e-09	
pos_patterns.pattern_27	34			MEF2A_MA0052.3	9.347320e-07	
pos_patterns.pattern_28	32			KLF12_HUMAN.H11MO.0.C	2.487710e-05	

pattern	num_seqlets	cwm_fwd	cwm_rev	TOMTOM_match	TOMTOM_qval	TOMTOM_match_logo
pos_patterns.pattern_29	31			PRDM1_HUMAN.H11MO.0.A	5.607540e-01	
pos_patterns.pattern_30	27			PAX5_MOUSE.H11MO.0.A	3.016880e-07	
pos_patterns.pattern_31	27			ZBTB33_MA0527.1	4.313920e-05	
pos_patterns.pattern_32	27			POU2F2_MA0507.1	1.456120e-01	
pos_patterns.pattern_33	22			JUN_MA0488.1	4.814100e-03	
neg_patterns.pattern_0	796			NFATC2_MA0152.1	3.082730e-01	
neg_patterns.pattern_1	476			ZBT7A_HUMAN.H11MO.0.A	8.705380e-04	
neg_patterns.pattern_2	276			RREB1_MA0073.1	1.530550e-02	
neg_patterns.pattern_3	190			IRF4_HUMAN.H11MO.0.A	9.010000e-03	
neg_patterns.pattern_4	189			NFAC1_HUMAN.H11MO.0.B	5.006260e-01	
neg_patterns.pattern_5	160			NFAC3_HUMAN.H11MO.0.B	2.500410e-01	
neg_patterns.pattern_6	150			SPIB_MOUSE.H11MO.0.A	2.806570e-04	
neg_patterns.pattern_7	139			ZNF384_MA1125.1	5.876630e-03	
neg_patterns.pattern_8	136			DNASE_2	1.400110e-01	
neg_patterns.pattern_9	133			SMCA5_MOUSE.H11MO.0.C	1.223470e-01	
neg_patterns.pattern_10	129			RELA_MA0107.1	1.777080e-03	
neg_patterns.pattern_11	93			JDP2_bZIP_1	4.234990e-05	
neg_patterns.pattern_12	90			ZNF384_MA1125.1	3.263780e-01	
neg_patterns.pattern_13	86			TBX3_HUMAN.H11MO.0.C	1.000000e+00	
neg_patterns.pattern_14	85			ZNF384_MA1125.1	4.805640e-01	
neg_patterns.pattern_15	76			ZBT7A_HUMAN.H11MO.0.A	1.071990e-01	
neg_patterns.pattern_16	75			IRF3_HUMAN.H11MO.0.B	1.635960e-01	
neg_patterns.pattern_17	70			IRF8_HUMAN.H11MO.0.B	5.473260e-05	
neg_patterns.pattern_18	69			ELK4_ETS_1	7.451550e-04	
neg_patterns.pattern_19	66			SPI1_HUMAN.H11MO.0.A	4.871920e-02	
neg_patterns.pattern_20	58			ONECUT3_CUT_1	2.024900e-01	
neg_patterns.pattern_21	56			NaN	NaN	
neg_patterns.pattern_22	51			CTCF_HUMAN.H11MO.0.A	1.000000e+00	
neg_patterns.pattern_23	44			BCL6B_C2H2_1	1.000000e+00	
neg_patterns.pattern_24	44			SOX7_HMG_3	2.522630e-01	

pattern	num_seqlets	cwm_fwd	cwm_rev	TOMTOM_match	TOMTOM_qval	TOMTOM_match_logo
neg_patterns.pattern_25	36	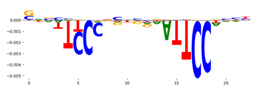	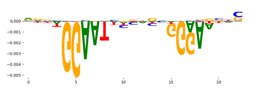	TEAD3_MA0808.1	3.810550e-01	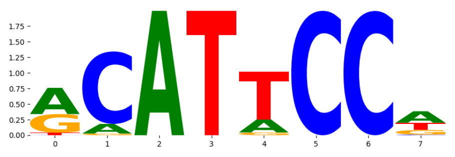
neg_patterns.pattern_26	32	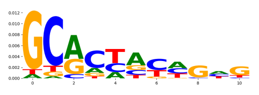	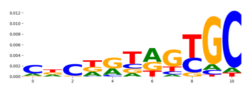	ZN264_HUMAN.H11MO.0.C	1.000000e+00	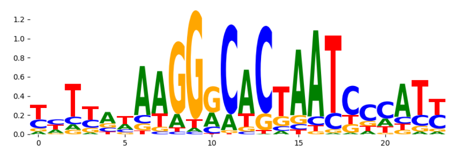
neg_patterns.pattern_27	29	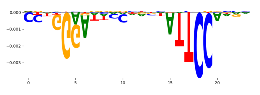	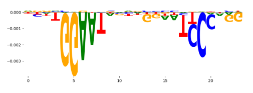	TEAD3_MA0808.1	3.227880e-01	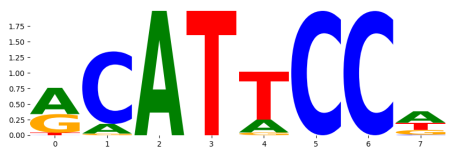
neg_patterns.pattern_28	28	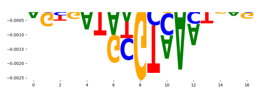	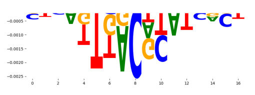	Foxg1.mouse_forkhead_1	3.832810e-01	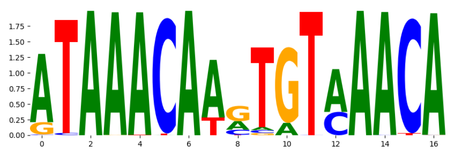
neg_patterns.pattern_29	27	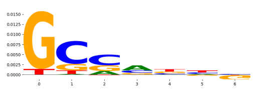	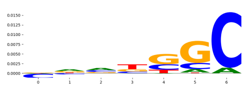	ZEB1_MA0103.3	1.000000e+00	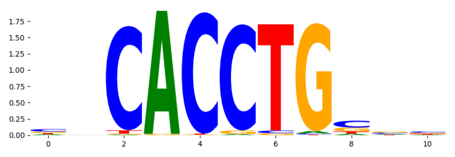
neg_patterns.pattern_30	26	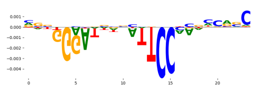	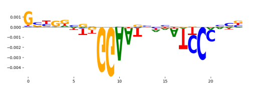	SOX18_HMG_3	7.999790e-01	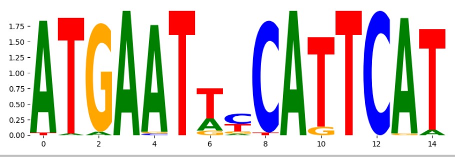
