# Supplementary material for: ChromBPNet: bias factorized, base-resolution deep learning models of chromatin accessibility reveal cis-regulatory sequence syntax, transcription factor footprints and regulatory variants
Source: bioRxiv. 2025 Jan 8:2024.12.25.630221. Preprint. [Version 2] doi: 10.1101/2024.12.25.630221 (PMC11741299; doi:10.1101/2024.12.25.630221)
Supplement: Supplement 2 [file media-2.zip › supplementary_files_1/6_ATAC_chrombpnet/fig1o_gm12878_ATAC_raw_chrombpnet_counts_modisco.pdf]

pattern	num_seqlets	cwm_fwd	cwm_rev	TOMTOM_match	TOMTOM_qval	TOMTOM_match_logo
pos_patterns.pattern_0	3247			CTCF_MA0139.1	5.308560e-13	
pos_patterns.pattern_1	2898			IRF4_MOUSE.H11MO.0.A	4.201300e-02	
pos_patterns.pattern_2	2751			ELF5_HUMAN.H11MO.0.A	2.734420e-04	
pos_patterns.pattern_3	2049			FOS+JUND_MA1141.1	2.575030e-03	
pos_patterns.pattern_4	1904			IRF1_MOUSE.H11MO.0.A	9.016690e-06	
pos_patterns.pattern_5	1821			RUNX3_HUMAN.H11MO.0.A	1.828630e-02	
pos_patterns.pattern_6	1612			RELB_HUMAN.H11MO.0.C	3.794730e-06	
pos_patterns.pattern_7	807			SP1_HUMAN.H11MO.0.A	1.591760e-05	
pos_patterns.pattern_8	799			ELK4_HUMAN.H11MO.0.A	1.005590e-05	
pos_patterns.pattern_9	375			REL_MA0101.1	1.826090e-01	
pos_patterns.pattern_10	335			NFYC_HUMAN.H11MO.0.A	6.717320e-03	
pos_patterns.pattern_11	322			NFKB1_HUMAN.H11MO.1.B	6.422330e-03	
pos_patterns.pattern_12	309			Pou2f2.mouse_POU_2	5.568920e-03	
pos_patterns.pattern_13	282			BATF_HUMAN.H11MO.0.A	1.126030e-06	
pos_patterns.pattern_14	277			NRF1_MOUSE.H11MO.0.A	2.666620e-07	
pos_patterns.pattern_15	217			Mafb.mouse_bZIP_3	1.999360e-04	
pos_patterns.pattern_16	212			ETS1_HUMAN.H11MO.0.A	2.905130e-04	
pos_patterns.pattern_17	200			COE1_MOUSE.H11MO.0.A	2.760570e-06	
pos_patterns.pattern_18	171			ZN143_MOUSE.H11MO.0.A	4.361840e-14	
pos_patterns.pattern_19	163			MEF2D_HUMAN.H11MO.0.A	2.501050e-10	
pos_patterns.pattern_20	149			SPIB_ETS_1	1.969570e-02	
pos_patterns.pattern_21	123			PAX2_PAX_1	3.134400e-08	
pos_patterns.pattern_22	89			HNF1B_HUMAN.H11MO.0.A	9.561310e-06	
pos_patterns.pattern_23	85			PAX5_MOUSE.H11MO.0.A	8.230710e-08	
pos_patterns.pattern_24	83			TYY1_HUMAN.H11MO.0.A	2.496250e-07	
pos_patterns.pattern_25	35			ZBTB33_MA0527.1	6.928390e-06	
pos_patterns.pattern_26	34			PAX2_PAX_1	4.710900e-04	
pos_patterns.pattern_27	21			THRA_nuclearreceptor_1	2.809530e-02	
neg_patterns.pattern_0	33			NFATC2_MA0152.1	3.798600e-02	
