# Supplementary material for: ChromBPNet: bias factorized, base-resolution deep learning models of chromatin accessibility reveal cis-regulatory sequence syntax, transcription factor footprints and regulatory variants
Source: bioRxiv. 2025 Jan 8:2024.12.25.630221. Preprint. [Version 2] doi: 10.1101/2024.12.25.630221 (PMC11741299; doi:10.1101/2024.12.25.630221)
Supplement: Supplement 3 [file media-3.zip › supplementary_files_2/3_DNASE_bpnet_w_simple_cnn_bias/fig2e_gm12878_DNASE_raw_bpnet_w_simple_cnn_bias_counts_modisco.pdf]

pattern	num_seqlets	cwm_fwd	cwm_rev	TOMTOM_match	TOMTOM_qval	TOMTOM_match_logo
pos_patterns.pattern_0	5233			ELF5_HUMAN.H11MO.0.A	6.305480e-05	
pos_patterns.pattern_1	4788			STAT2_HUMAN.H11MO.0.A	3.986260e-03	
pos_patterns.pattern_2	3148			CTCF_MA0139.1	1.280350e-10	
pos_patterns.pattern_3	3127			RUNX1_HUMAN.H11MO.0.A	5.697370e-04	
pos_patterns.pattern_4	2375			ATF3_MOUSE.H11MO.0.A	3.701810e-03	
pos_patterns.pattern_5	1886			IRF4_HUMAN.H11MO.0.A	2.849450e-07	
pos_patterns.pattern_6	1098			RELB_HUMAN.H11MO.0.C	2.919520e-05	
pos_patterns.pattern_7	683			NFKB2_HUMAN.H11MO.0.B	8.284950e-04	
pos_patterns.pattern_8	627			NRF1_MA0506.1	7.182100e-06	
pos_patterns.pattern_9	602			KLF12_HUMAN.H11MO.0.C	8.946070e-05	
pos_patterns.pattern_10	582			ATF3_HUMAN.H11MO.0.A	7.083360e-04	
pos_patterns.pattern_11	459			NFYB_HUMAN.H11MO.0.A	5.790940e-03	
pos_patterns.pattern_12	386			ZN143_MOUSE.H11MO.0.A	4.892370e-19	
pos_patterns.pattern_13	362			POU5F1_MA1115.1	3.450920e-03	
pos_patterns.pattern_14	147			COE1_HUMAN.H11MO.0.A	1.096840e-04	
pos_patterns.pattern_15	83			ZBTB33_MA0527.1	2.438900e-04	
pos_patterns.pattern_16	38			RUNX3_RUNX_1	1.218610e-02	
pos_patterns.pattern_17	37			MEF2D_MOUSE.H11MO.0.A	5.839710e-05	
pos_patterns.pattern_18	27			RFX2_HUMAN.H11MO.0.A	2.804740e-09	
pos_patterns.pattern_19	23			Gabpa_MA0062.2	6.330340e-01	
pos_patterns.pattern_20	20			RUNX3_HUMAN.H11MO.0.A	3.905670e-01	
pos_patterns.pattern_21	20			RELA_MA0107.1	2.682180e-01	
