# Supplementary material for: ChromBPNet: bias factorized, base-resolution deep learning models of chromatin accessibility reveal cis-regulatory sequence syntax, transcription factor footprints and regulatory variants
Source: bioRxiv. 2025 Jan 8:2024.12.25.630221. Preprint. [Version 2] doi: 10.1101/2024.12.25.630221 (PMC11741299; doi:10.1101/2024.12.25.630221)
Supplement: Supplement 3 [file media-3.zip › supplementary_files_2/2_DNASE_hint_corrected_bpnet/fig2c_gm12878_DNASE_hint_corrected_bpnet_counts_modisco.pdf]

pattern	num_seqlets	cwm_fwd	cwm_rev	TOMTOM_match	TOMTOM_qval	TOMTOM_match_logo
pos_patterns.pattern_0	4738			IRF1_MOUSE.H11MO.0.A	9.414170e-04	
pos_patterns.pattern_1	3534			ETV4_MOUSE.H11MO.0.B	3.151370e-03	
pos_patterns.pattern_2	2787			FOS+JUND_MA1141.1	1.577420e-04	
pos_patterns.pattern_3	2637			RUNX3_HUMAN.H11MO.0.A	1.304710e-03	
pos_patterns.pattern_4	2212			TF65_MOUSE.H11MO.0.A	3.804810e-08	
pos_patterns.pattern_5	2139			CTCF_MA0139.1	2.293180e-13	
pos_patterns.pattern_6	1991			ELK4_HUMAN.H11MO.0.A	3.103140e-04	
pos_patterns.pattern_7	1796			KLF12_HUMAN.H11MO.0.C	1.251460e-05	
pos_patterns.pattern_8	1426			IRF4_HUMAN.H11MO.0.A	6.666610e-06	
pos_patterns.pattern_9	794			NFYB_HUMAN.H11MO.0.A	3.717810e-03	
pos_patterns.pattern_10	780			ATF3_HUMAN.H11MO.0.A	5.651600e-03	
pos_patterns.pattern_11	739			NRF1_HUMAN.H11MO.0.A	2.481590e-06	
pos_patterns.pattern_12	530			COE1_MOUSE.H11MO.0.A	8.134510e-07	
pos_patterns.pattern_13	507			TF65_HUMAN.H11MO.0.A	1.923230e-01	
pos_patterns.pattern_14	484			POU5F1_MA1115.1	3.022450e-03	
pos_patterns.pattern_15	295			ZN143_MOUSE.H11MO.0.A	5.551070e-16	
pos_patterns.pattern_16	211			MEF2D_HUMAN.H11MO.0.A	9.715190e-09	
pos_patterns.pattern_17	205			TYY1_HUMAN.H11MO.0.A	2.217760e-05	
pos_patterns.pattern_18	180			SPIB_ETS_1	9.319850e-03	
pos_patterns.pattern_19	173			ZBTB33_MA0527.1	1.252540e-03	
pos_patterns.pattern_20	121			PAX2_PAX_1	1.150330e-07	
pos_patterns.pattern_21	100			PAX1_MA0779.1	2.645540e-02	
pos_patterns.pattern_22	94			PAX2_PAX_1	5.707590e-14	
pos_patterns.pattern_23	84			ETV2_HUMAN.H11MO.0.B	3.544090e-01	
pos_patterns.pattern_24	83			HNF1A_HUMAN.H11MO.0.C	1.495850e-05	
pos_patterns.pattern_25	57			RFX2_MOUSE.H11MO.0.A	1.580220e-11	
pos_patterns.pattern_26	45			NFIA_HUMAN.H11MO.0.C	1.386330e-04	
pos_patterns.pattern_27	40			PAX5_MOUSE.H11MO.0.A	1.032360e-04	
pos_patterns.pattern_28	39			MEF2D_HUMAN.H11MO.0.A	7.279740e-03	

pattern	num_seqlets	cwm_fwd	cwm_rev	TOMTOM_match	TOMTOM_qval	TOMTOM_match_logo
pos_patterns.pattern_29	34	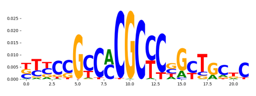	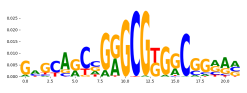	KLF12_HUMAN.H11MO.0.C	4.235060e-04	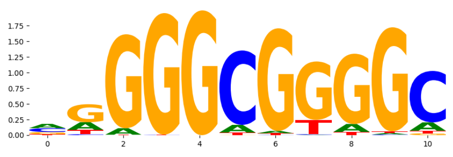
pos_patterns.pattern_30	32	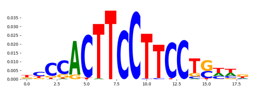	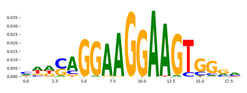	EWSR1-FLI1_MA0149.1	7.417030e-02	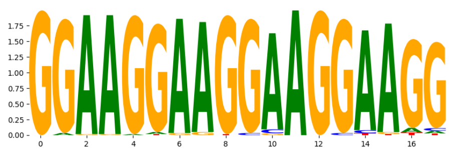
pos_patterns.pattern_31	32	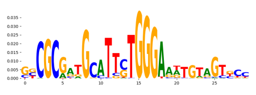	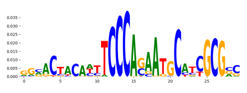	ZNF76_HUMAN.H11MO.0.C	4.371540e-09	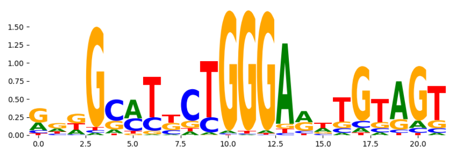
pos_patterns.pattern_32	29	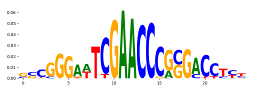	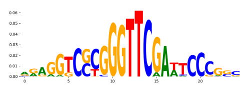	ZNF524_C2H2_2	7.440270e-01	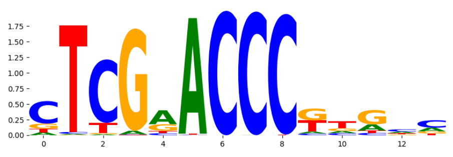
pos_patterns.pattern_33	21	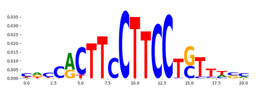	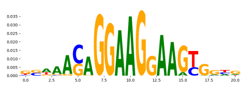	ETV6_MOUSE.H11MO.0.C	2.074270e-01	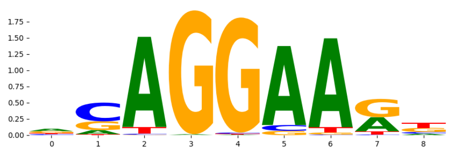
neg_patterns.pattern_0	212	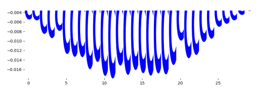	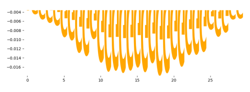	SP5_MOUSE.H11MO.0.C	2.232230e-07	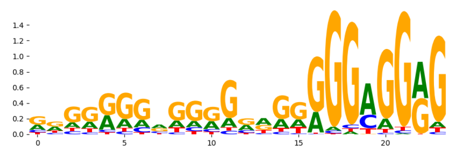
neg_patterns.pattern_1	25	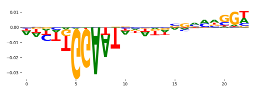	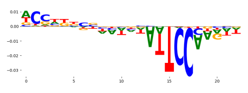	HXB7_HUMAN.H11MO.0.C	7.349900e-01	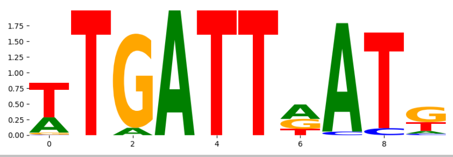
