# Supplementary material for: ChromBPNet: bias factorized, base-resolution deep learning models of chromatin accessibility reveal cis-regulatory sequence syntax, transcription factor footprints and regulatory variants
Source: bioRxiv. 2025 Jan 8:2024.12.25.630221. Preprint. [Version 2] doi: 10.1101/2024.12.25.630221 (PMC11741299; doi:10.1101/2024.12.25.630221)
Supplement: Supplement 3 [file media-3.zip › supplementary_files_2/1_DNASE_raw_bpnet_uncorrected/fig2a_gm12878_DNASE_raw_bpnet_uncorrected_counts_modisco.pdf]

pattern	num_seqlets	cwm_fwd	cwm_rev	TOMTOM_match	TOMTOM_qval	TOMTOM_match_logo
pos_patterns.pattern_0	4409			IRF1_MOUSE.H11MO.0.A	7.444490e-04	
pos_patterns.pattern_1	3684			SPIB_MOUSE.H11MO.0.A	1.480820e-05	
pos_patterns.pattern_2	3044			RUNX1_HUMAN.H11MO.0.A	4.132100e-03	
pos_patterns.pattern_3	2536			JUNB_HUMAN.H11MO.0.A	2.812630e-03	
pos_patterns.pattern_4	2111			NFKB1_HUMAN.H11MO.1.B	2.768810e-08	
pos_patterns.pattern_5	2085			CTCF_MA0139.1	1.274450e-11	
pos_patterns.pattern_6	1796			SP1_HUMAN.H11MO.0.A	5.770860e-05	
pos_patterns.pattern_7	1684			Gabpa_MA0062.2	4.774250e-05	
pos_patterns.pattern_8	1089			ATF3_HUMAN.H11MO.0.A	4.449200e-03	
pos_patterns.pattern_9	982			NRF1_MA0506.1	5.413410e-07	
pos_patterns.pattern_10	802			NFYB_HUMAN.H11MO.0.A	1.421390e-03	
pos_patterns.pattern_11	382			RELB_HUMAN.H11MO.0.C	1.000000e+00	
pos_patterns.pattern_12	339			POU5F1_MA1115.1	3.582870e-03	
pos_patterns.pattern_13	299			EBF1_EBF_1	1.746020e-05	
pos_patterns.pattern_14	290			ZNF76_HUMAN.H11MO.0.C	8.680800e-22	
pos_patterns.pattern_15	261			IRF4_MOUSE.H11MO.0.A	6.509460e-04	
pos_patterns.pattern_16	187			PAX1_MA0779.1	2.123530e-06	
pos_patterns.pattern_17	144			ZBTB33_MA0527.1	1.845290e-04	
pos_patterns.pattern_18	111			MEF2D_MOUSE.H11MO.0.A	1.276620e-06	
pos_patterns.pattern_19	65			NFIA_NFI_1	7.049680e-04	
pos_patterns.pattern_20	63			TYY1_HUMAN.H11MO.0.A	4.536160e-05	
pos_patterns.pattern_21	53			RFX2_HUMAN.H11MO.0.A	2.041250e-08	
pos_patterns.pattern_22	41			Gabpa_MA0062.2	1.960340e-01	
pos_patterns.pattern_23	41			PAX2_PAX_1	4.355510e-07	
pos_patterns.pattern_24	39			HNF1B_HUMAN.H11MO.0.A	9.153840e-06	
pos_patterns.pattern_25	37			PRDM6_HUMAN.H11MO.0.C	4.408250e-02	
pos_patterns.pattern_26	37			SP1_HUMAN.H11MO.0.A	5.460660e-07	
pos_patterns.pattern_27	23			PAX2_PAX_1	2.247030e-03	
pos_patterns.pattern_28	21			PRD14_HUMAN.H11MO.0.A	1.000000e+00	

pattern	num_seqlets	cwm_fwd	cwm_rev	TOMTOM_match	TOMTOM_qval	TOMTOM_match_logo
pos_patterns.pattern_29	20	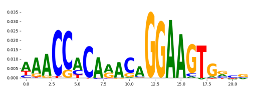	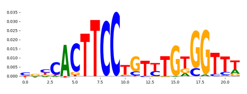	ETV4_MOUSE.H11MO.0.B	9.482910e-03	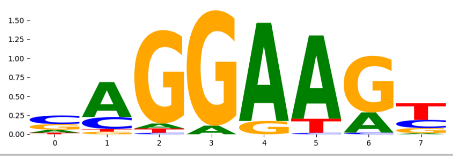
