# Supplementary material for: ChromBPNet: bias factorized, base-resolution deep learning models of chromatin accessibility reveal cis-regulatory sequence syntax, transcription factor footprints and regulatory variants
Source: bioRxiv. 2025 Jan 8:2024.12.25.630221. Preprint. [Version 2] doi: 10.1101/2024.12.25.630221 (PMC11741299; doi:10.1101/2024.12.25.630221)
Supplement: Supplement 3 [file media-3.zip › supplementary_files_2/1_DNASE_raw_bpnet_uncorrected/fig2b_gm12878_DNASE_raw_bpnet_uncorrected_profile_modisco.pdf]

pattern	num_seqlets	cwm_fwd	cwm_rev	TOMTOM_match	TOMTOM_qval	TOMTOM_match_logo
pos_patterns.pattern_0	4753			CTCF_MA0139.1	2.748960e-12	
pos_patterns.pattern_1	2984			ELF5_HUMAN.H11MO.0.A	9.836690e-05	
pos_patterns.pattern_2	2932			RUNX1_HUMAN.H11MO.0.A	8.685900e-02	
pos_patterns.pattern_3	2884			ATF3_MOUSE.H11MO.0.A	4.129770e-03	
pos_patterns.pattern_4	1979			IRF8_HUMAN.H11MO.0.B	1.040800e-05	
pos_patterns.pattern_5	1883			SIX2_MA1119.1	5.779520e-02	
pos_patterns.pattern_6	825			RELB_HUMAN.H11MO.0.C	1.352190e-07	
pos_patterns.pattern_7	785			RARA_nuclearreceptor_5	1.000000e+00	
pos_patterns.pattern_8	658			KLF12_HUMAN.H11MO.0.C	1.278430e-06	
pos_patterns.pattern_9	584			NFYC_HUMAN.H11MO.0.A	8.614680e-06	
pos_patterns.pattern_10	571			NRF1_MOUSE.H11MO.0.A	1.211210e-05	
pos_patterns.pattern_11	355			MITF_MA0620.2	3.577560e-06	
pos_patterns.pattern_12	341			NFKB1_HUMAN.H11MO.1.B	1.266870e-03	
pos_patterns.pattern_13	319			FOSL2+JUN_MA1131.1	4.061170e-05	
pos_patterns.pattern_14	310			NaN	NaN	
pos_patterns.pattern_15	277			MAZ_HUMAN.H11MO.0.A	1.695200e-07	
pos_patterns.pattern_16	240			COE1_MOUSE.H11MO.0.A	4.520040e-05	
pos_patterns.pattern_17	233			PO2F2_HUMAN.H11MO.0.A	9.060280e-04	
pos_patterns.pattern_18	232			PAX1_MA0779.1	1.601050e-05	
pos_patterns.pattern_19	188			EGR2_HUMAN.H11MO.0.A	2.405910e-04	
pos_patterns.pattern_20	173			ZN143_HUMAN.H11MO.0.A	4.017130e-15	
pos_patterns.pattern_21	133			NFIA_HUMAN.H11MO.0.C	8.056880e-05	
pos_patterns.pattern_22	88			RFX3_MOUSE.H11MO.0.C	1.898380e-10	
pos_patterns.pattern_23	62			SPIB_ETS_1	5.293210e-02	
pos_patterns.pattern_24	48			ZBTB33_MA0527.1	6.110090e-03	
pos_patterns.pattern_25	25			ZN770_HUMAN.H11MO.0.C	4.335230e-02	
pos_patterns.pattern_26	20			PAX6_PAX_1	2.708400e-04	
neg_patterns.pattern_0	88			NFATC2_MA0152.1	6.944530e-01	
neg_patterns.pattern_1	72			DNASE_2	1.447250e-01	
neg_patterns.pattern_2	70			DNASE_2	2.132820e-01	

pattern	num_seqlets	cwm_fwd	cwm_rev	TOMTOM_match	TOMTOM_qval	TOMTOM_match_logo
neg_patterns.pattern_3	39	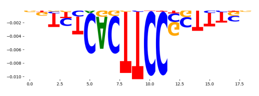	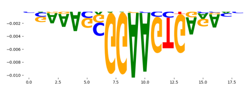	SPIB_MOUSE.H11MO.0.A	2.046650e-05	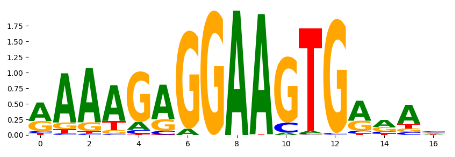
neg_patterns.pattern_4	27	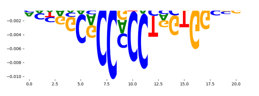	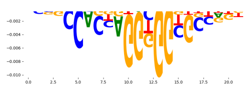	CTCF_MA0139.1	7.212070e-04	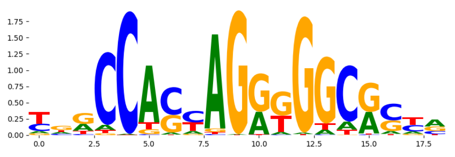
neg_patterns.pattern_5	22	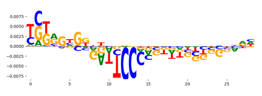	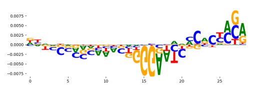	FLI1_ETS_4	1.000000e+00	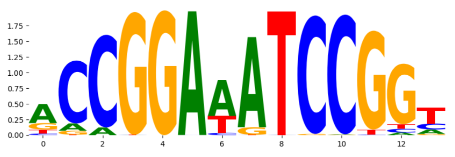
neg_patterns.pattern_6	22	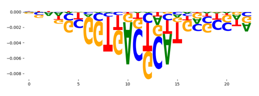	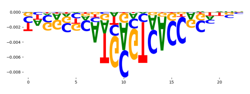	FOS_MA0476.1	3.004900e-02	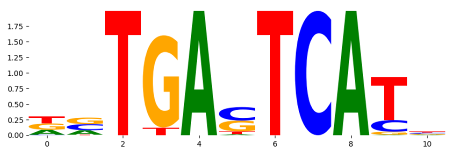
neg_patterns.pattern_7	21	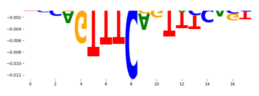	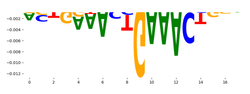	STAT1+STAT2_MA0517.1	2.546030e-05	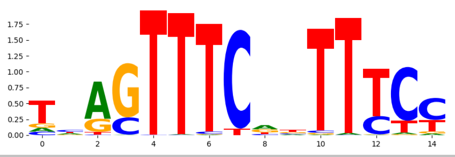
