# Supplementary material for: ChromBPNet: bias factorized, base-resolution deep learning models of chromatin accessibility reveal cis-regulatory sequence syntax, transcription factor footprints and regulatory variants
Source: bioRxiv. 2025 Jan 8:2024.12.25.630221. Preprint. [Version 2] doi: 10.1101/2024.12.25.630221 (PMC11741299; doi:10.1101/2024.12.25.630221)
Supplement: Supplement 3 [file media-3.zip › supplementary_files_2/4_DNASE_chrombpnet/fig2g_gm12878_DNASE_raw_chrombpnet_profile_modisco.pdf]

pattern	num_seqlets	cwm_fwd	cwm_rev	TOMTOM_match	TOMTOM_qval	TOMTOM_match_logo
pos_patterns.pattern_0	5066			ELF5_HUMAN.H11MO.0.A	1.428730e-05	
pos_patterns.pattern_1	3975			CTCF_MA0139.1	1.062330e-12	
pos_patterns.pattern_2	2871			FOS_HUMAN.H11MO.0.A	1.183150e-03	
pos_patterns.pattern_3	2799			STAT2_HUMAN.H11MO.0.A	7.865400e-03	
pos_patterns.pattern_4	2576			RUNX1_HUMAN.H11MO.0.A	1.778140e-02	
pos_patterns.pattern_5	2534			IRF4_MOUSE.H11MO.0.A	3.367440e-05	
pos_patterns.pattern_6	2333			NFKB1_HUMAN.H11MO.1.B	2.431960e-08	
pos_patterns.pattern_7	1064			KLF12_HUMAN.H11MO.0.C	1.081220e-05	
pos_patterns.pattern_8	762			NRF1_MA0506.1	1.646800e-06	
pos_patterns.pattern_9	548			Pou2f2.mouse_POU_2	5.314080e-03	
pos_patterns.pattern_10	497			FOSB+JUNB_MA1136.1	4.589460e-05	
pos_patterns.pattern_11	466			NFYB_HUMAN.H11MO.0.A	1.320010e-03	
pos_patterns.pattern_12	465			COE1_MOUSE.H11MO.0.A	1.073500e-06	
pos_patterns.pattern_13	420			RELB_HUMAN.H11MO.0.C	6.833930e-01	
pos_patterns.pattern_14	339			ZN143_MOUSE.H11MO.0.A	5.872660e-15	
pos_patterns.pattern_15	314			ETS1_HUMAN.H11MO.0.A	2.869920e-02	
pos_patterns.pattern_16	216			ARNTL_bHLH_1	4.374330e-05	
pos_patterns.pattern_17	149			SPIB_ETS_1	4.166820e-02	
pos_patterns.pattern_18	132			PAX9_MA0781.1	6.841950e-08	
pos_patterns.pattern_19	107			MEF2D_HUMAN.H11MO.0.A	1.326780e-04	
pos_patterns.pattern_20	107			ZBTB33_MA0527.1	5.588200e-06	
pos_patterns.pattern_21	101			HNF1B_MOUSE.H11MO.0.A	1.047060e-05	
pos_patterns.pattern_22	56			TYY1_HUMAN.H11MO.0.A	1.989540e-05	
pos_patterns.pattern_23	43			BATF+JUN_MA0462.1	1.396300e-03	
pos_patterns.pattern_24	38			PAX6_PAX_1	1.729180e-02	
pos_patterns.pattern_25	27			ETV6_ETS_1	1.637700e-04	
pos_patterns.pattern_26	25			Rfx1_MA0509.1	9.727740e-09	
pos_patterns.pattern_27	21			ZNF524_C2H2_2	1.000000e+00	
