# Supplementary material for: ChromBPNet: bias factorized, base-resolution deep learning models of chromatin accessibility reveal cis-regulatory sequence syntax, transcription factor footprints and regulatory variants
Source: bioRxiv. 2025 Jan 8:2024.12.25.630221. Preprint. [Version 2] doi: 10.1101/2024.12.25.630221 (PMC11741299; doi:10.1101/2024.12.25.630221)
Supplement: Supplement 4 [file media-4.zip › supplementary_files_3/hepg2_ATAC_bpnet_bias_model/hepg2_ATAC_raw_bpnet_bias_fold4_counts_modisco.pdf]

pattern	num_seqlets	cwm_fwd	cwm_rev	TOMTOM_match	TOMTOM_qval	TOMTOM_match_logo
pos_patterns.pattern_0	2818			ZN320_HUMAN.H11MO.0.C	4.594080e-01	
pos_patterns.pattern_1	1941			ZFX_MOUSE.H11MO.0.B	9.701540e-02	
pos_patterns.pattern_2	1904			ZNF384_MA1125.1	1.421240e-01	
pos_patterns.pattern_3	1566			SP3_HUMAN.H11MO.0.B	4.920160e-02	
pos_patterns.pattern_4	963			GATA1+TAL1_MA0140.2	1.000000e+00	
pos_patterns.pattern_5	908			NFATC3_MA0625.1	1.632030e-01	
pos_patterns.pattern_6	827			DNASE_1	1.000000e+00	
pos_patterns.pattern_7	785			SNAI1_HUMAN.H11MO.0.C	2.386940e-02	
pos_patterns.pattern_8	706			PLAG1_MA0163.1	1.562540e-02	
pos_patterns.pattern_9	685			NR2F1_nuclearreceptor_3	8.579050e-02	
pos_patterns.pattern_10	626			SP1_HUMAN.H11MO.0.A	4.324510e-04	
pos_patterns.pattern_11	529			RREB1_MA0073.1	7.257090e-02	
pos_patterns.pattern_12	522			GATA1+TAL1_MA0140.2	1.000000e+00	
pos_patterns.pattern_13	492			SP1_MOUSE.H11MO.0.A	7.476610e-02	
pos_patterns.pattern_14	471			Hnf4a.mouse_nuclearreceptor_1	9.999860e-01	
pos_patterns.pattern_15	439			NFAC1_MOUSE.H11MO.0.A	1.000000e+00	
pos_patterns.pattern_16	339			RREB1_MA0073.1	2.545570e-02	
pos_patterns.pattern_17	286			NaN	NaN	
pos_patterns.pattern_18	274			MYBL2_MYB_2	9.312390e-01	
pos_patterns.pattern_19	243			SP1_HUMAN.H11MO.0.A	1.366920e-01	
pos_patterns.pattern_20	222			RREB1_MA0073.1	2.125040e-03	
pos_patterns.pattern_21	195			FOXK1_MA0852.2	1.000000e+00	
pos_patterns.pattern_22	165			PBX3_MA1114.1	1.000000e+00	
pos_patterns.pattern_23	83			Hoxa9_MA0594.1	1.000000e+00	
pos_patterns.pattern_24	33			SP1_MOUSE.H11MO.0.A	5.009200e-02	
neg_patterns.pattern_0	406			TN5_2	3.693140e-07	
neg_patterns.pattern_1	271			TN5_2	1.349410e-03	
neg_patterns.pattern_2	122			ZIC1_C2H2_1	1.572720e-01	
neg_patterns.pattern_3	112			SP2_HUMAN.H11MO.0.A	1.000060e-05	
neg_patterns.pattern_4	96			CTCF_MA0139.1	2.905270e-07	

pattern	num_seqlets	cwm_fwd	cwm_rev	TOMTOM_match	TOMTOM_qval	TOMTOM_match_logo
neg_patterns.pattern_5	95	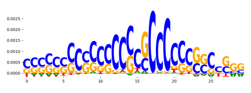	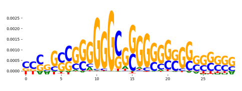	ZBT17_HUMAN.H11MO.0.A	4.379260e-03	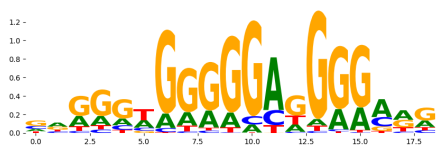
neg_patterns.pattern_6	91	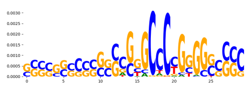	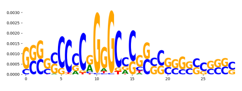	AP2C_HUMAN.H11MO.0.A	5.706460e-01	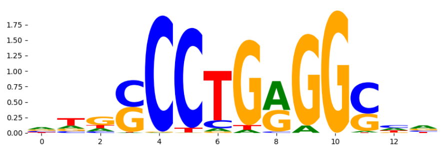
neg_patterns.pattern_7	81	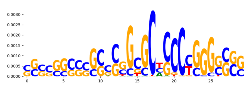	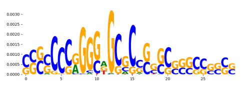	ZN528_HUMAN.H11MO.0.C	1.000000e+00	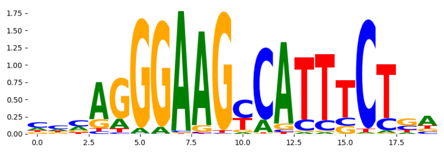
neg_patterns.pattern_8	35	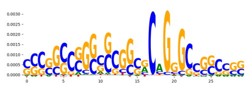	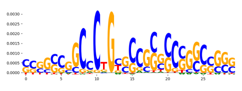	TN5_1	2.591020e-01	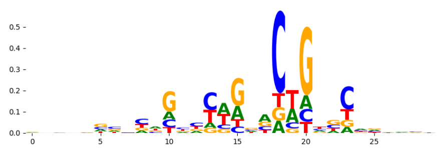
neg_patterns.pattern_9	33	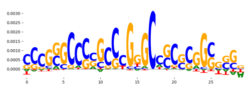	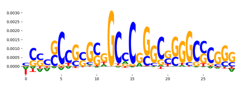	PAX5_HUMAN.H11MO.0.A	1.000000e+00	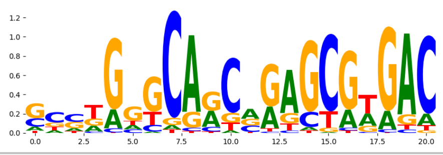
