# Supplementary material for: ChromBPNet: bias factorized, base-resolution deep learning models of chromatin accessibility reveal cis-regulatory sequence syntax, transcription factor footprints and regulatory variants
Source: bioRxiv. 2025 Jan 8:2024.12.25.630221. Preprint. [Version 2] doi: 10.1101/2024.12.25.630221 (PMC11741299; doi:10.1101/2024.12.25.630221)
Supplement: Supplement 4 [file media-4.zip › supplementary_files_3/hepg2_ATAC_bpnet_bias_model/hepg2_ATAC_raw_bpnet_bias_fold2_counts_modisco.pdf]

pattern	num_seqlets	cwm_fwd	cwm_rev	TOMTOM_match	TOMTOM_qval	TOMTOM_match_logo
pos_patterns.pattern_0	6183			SP2_HUMAN.H11MO.0.A	4.561370e-02	
pos_patterns.pattern_1	3409			SP2_HUMAN.H11MO.0.A	3.201500e-07	
pos_patterns.pattern_2	1329			ZNF384_MA1125.1	4.313160e-02	
pos_patterns.pattern_3	861			RUNX2_HUMAN.H11MO.0.A	1.000000e+00	
pos_patterns.pattern_4	688			ZN667_HUMAN.H11MO.0.C	1.000000e+00	
pos_patterns.pattern_5	685			SP2_MA0516.1	7.453490e-02	
pos_patterns.pattern_6	562			LEF1_HMG_1	1.233180e-01	
pos_patterns.pattern_7	549			COE1_MOUSE.H11MO.0.A	3.453010e-02	
pos_patterns.pattern_8	540			Rara.mouse_nuclearreceptor_3	5.771810e-01	
pos_patterns.pattern_9	539			SOX18_HMG_3	9.060570e-02	
pos_patterns.pattern_10	537			RARG_nuclearreceptor_5	9.315850e-01	
pos_patterns.pattern_11	503			ZN320_HUMAN.H11MO.0.C	5.317270e-01	
pos_patterns.pattern_12	485			E2F6_HUMAN.H11MO.0.A	1.000000e+00	
pos_patterns.pattern_13	342			CDX2_MOUSE.H11MO.0.A	5.878200e-01	
pos_patterns.pattern_14	335			ONECUT3_CUT_1	2.049560e-01	
pos_patterns.pattern_15	319			ZN274_HUMAN.H11MO.0.A	1.000000e+00	
pos_patterns.pattern_16	198			ESRRA_nuclearreceptor_1	1.999700e-01	
pos_patterns.pattern_17	54			CPEB1_RRM_1	1.000000e+00	
pos_patterns.pattern_18	20			RORA_HUMAN.H11MO.0.C	3.656980e-01	
neg_patterns.pattern_0	587			TN5_4	7.512680e-03	
neg_patterns.pattern_1	204			SP1_HUMAN.H11MO.0.A	2.496090e-04	
neg_patterns.pattern_2	171			USF2_HUMAN.H11MO.0.A	3.041830e-02	
neg_patterns.pattern_3	153			ZN331_HUMAN.H11MO.0.C	2.041380e-03	
neg_patterns.pattern_4	134			SP2_HUMAN.H11MO.0.A	4.150630e-03	
neg_patterns.pattern_5	86			ZFX_MOUSE.H11MO.0.B	1.071070e-02	
neg_patterns.pattern_6	60			E2F3_MOUSE.H11MO.0.A	1.026310e-02	
neg_patterns.pattern_7	36			TN5_2	7.760040e-01	
