# Supplementary material for: ChromBPNet: bias factorized, base-resolution deep learning models of chromatin accessibility reveal cis-regulatory sequence syntax, transcription factor footprints and regulatory variants
Source: bioRxiv. 2025 Jan 8:2024.12.25.630221. Preprint. [Version 2] doi: 10.1101/2024.12.25.630221 (PMC11741299; doi:10.1101/2024.12.25.630221)
Supplement: Supplement 4 [file media-4.zip › supplementary_files_3/hepg2_ATAC_bpnet_bias_model/hepg2_ATAC_raw_bpnet_bias_fold1_counts_modisco.pdf]

pattern	num_seqlets	cwm_fwd	cwm_rev
pos_patterns.pattern_0	693		
pos_patterns.pattern_1	478		
pos_patterns.pattern_2	173		
pos_patterns.pattern_3	172		
pos_patterns.pattern_4	133		
pos_patterns.pattern_5	129		
pos_patterns.pattern_6	120		
pos_patterns.pattern_7	118		
pos_patterns.pattern_8	115		
pos_patterns.pattern_9	96		
pos_patterns.pattern_10	69		
pos_patterns.pattern_11	61		
pos_patterns.pattern_12	36		
pos_patterns.pattern_13	28		
pos_patterns.pattern_14	25		
neg_patterns.pattern_0	8321		
neg_patterns.pattern_1	1780		
neg_patterns.pattern_2	1513		
neg_patterns.pattern_3	1481		
neg_patterns.pattern_4	1029		
neg_patterns.pattern_5	962		
neg_patterns.pattern_6	863		
neg_patterns.pattern_7	786		

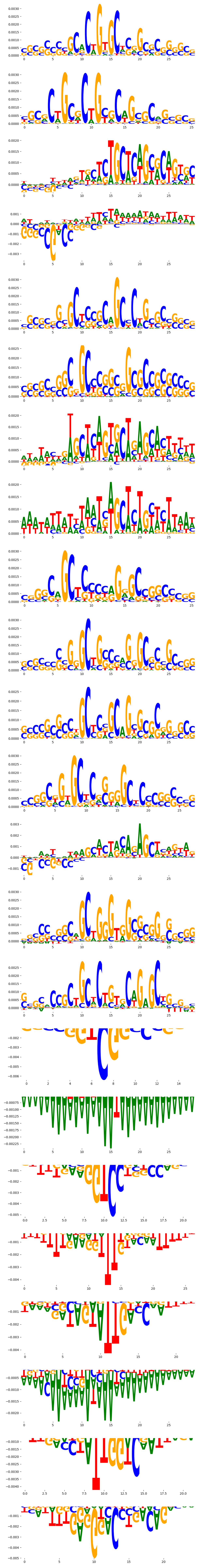

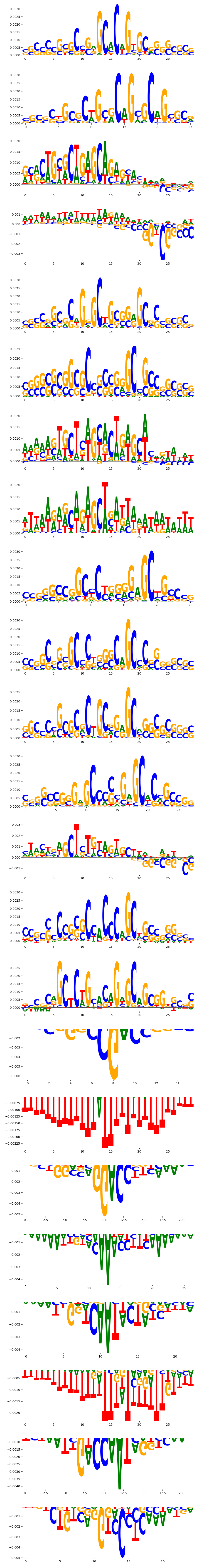

pattern	num_seqlets	cwm_fwd	cwm_rev
neg_patterns.pattern_8	765	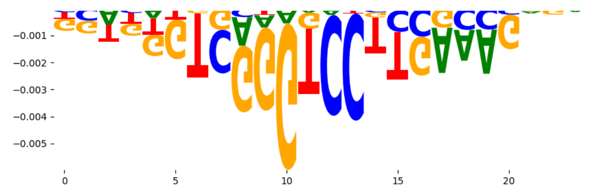	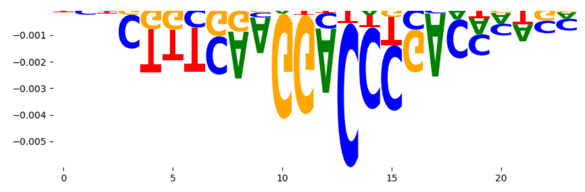
neg_patterns.pattern_9	757	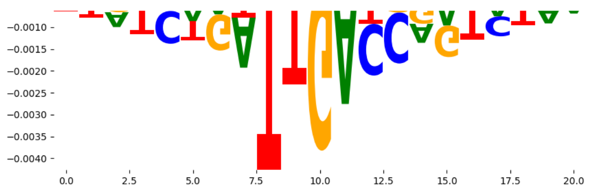	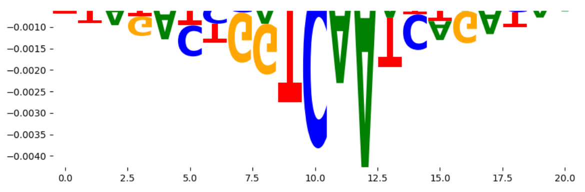
neg_patterns.pattern_10	674	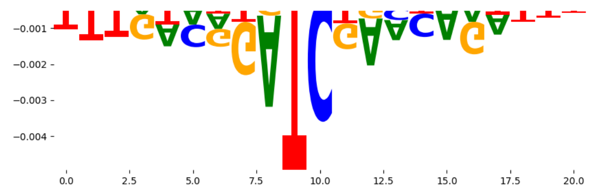	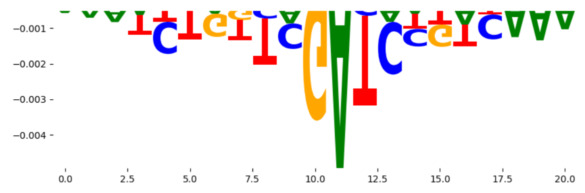
neg_patterns.pattern_11	665	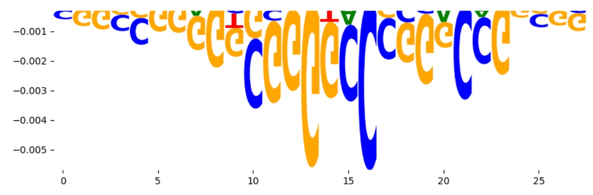	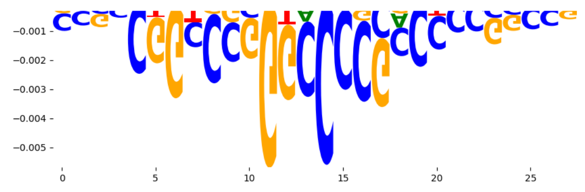
neg_patterns.pattern_12	644	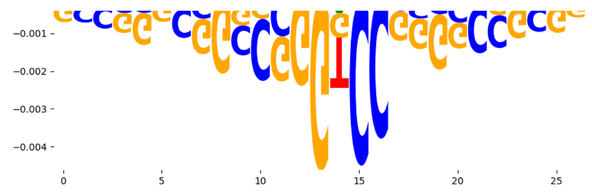	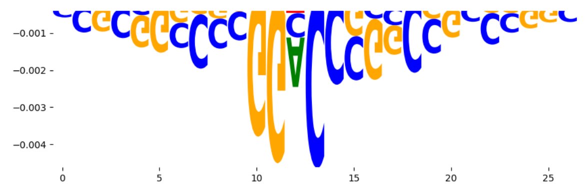
neg_patterns.pattern_13	489	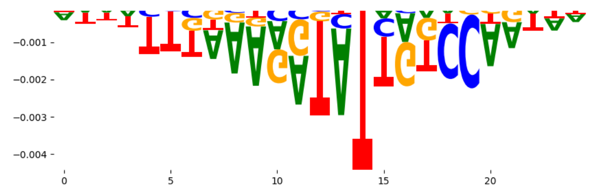	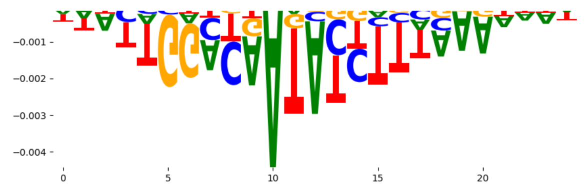
neg_patterns.pattern_14	425	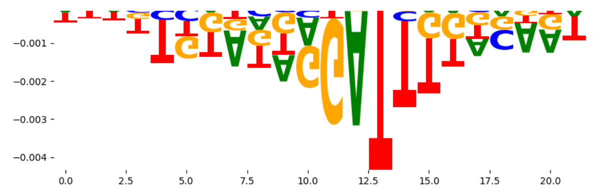	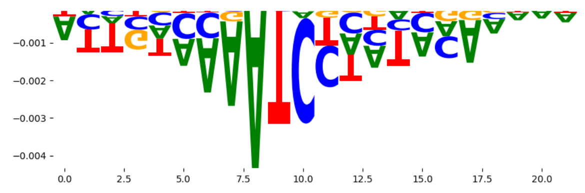
neg_patterns.pattern_15	254	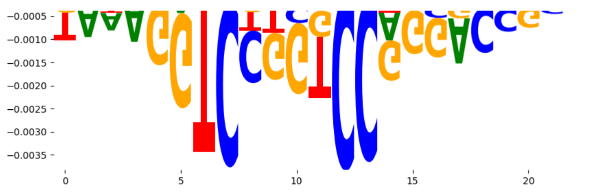	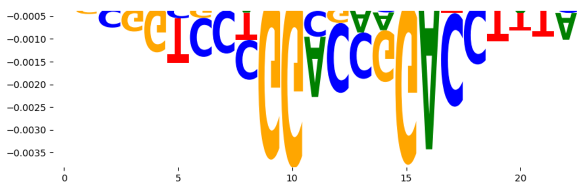
neg_patterns.pattern_16	251	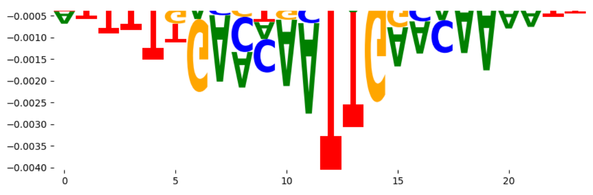	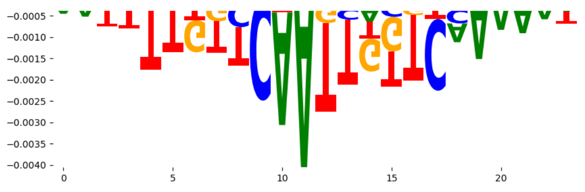
neg_patterns.pattern_17	227	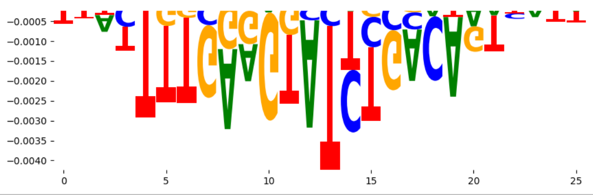	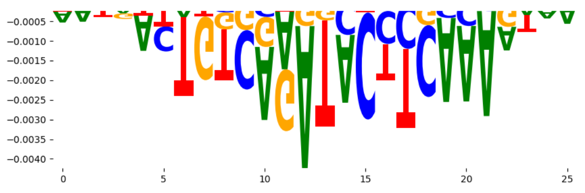
