# Supplementary material for: ChromBPNet: bias factorized, base-resolution deep learning models of chromatin accessibility reveal cis-regulatory sequence syntax, transcription factor footprints and regulatory variants
Source: bioRxiv. 2025 Jan 8:2024.12.25.630221. Preprint. [Version 2] doi: 10.1101/2024.12.25.630221 (PMC11741299; doi:10.1101/2024.12.25.630221)
Supplement: Supplement 4 [file media-4.zip › supplementary_files_3/hepg2_ATAC_bpnet_bias_model/hepg2_ATAC_raw_bpnet_bias_fold3_counts_modisco.pdf]

pattern	num_seqlets	cwm_fwd	cwm_rev	TOMTOM_match	TOMTOM_qval	TOMTOM_match_logo
pos_patterns.pattern_0	4279			SP1_HUMAN.H11MO.0.A	5.256980e-01	
pos_patterns.pattern_1	2891			ZFX_MOUSE.H11MO.0.B	4.984460e-03	
pos_patterns.pattern_2	1631			ZNF384_MA1125.1	9.365280e-02	
pos_patterns.pattern_3	1628			SP1_HUMAN.H11MO.0.A	8.003940e-03	
pos_patterns.pattern_4	892			Arid5a_MA0602.1	1.000000e+00	
pos_patterns.pattern_5	855			HNF4A_nuclearreceptor_4	4.039120e-01	
pos_patterns.pattern_6	774			HXA13_HUMAN.H11MO.0.C	9.324020e-01	
pos_patterns.pattern_7	748			SP2_HUMAN.H11MO.0.A	3.694840e-09	
pos_patterns.pattern_8	730			ZIC3_HUMAN.H11MO.0.B	2.580640e-01	
pos_patterns.pattern_9	730			PLAG1_MA0163.1	6.112310e-01	
pos_patterns.pattern_10	706			SP2_HUMAN.H11MO.0.A	2.042260e-03	
pos_patterns.pattern_11	703			BHA15_HUMAN.H11MO.0.B	3.479100e-01	
pos_patterns.pattern_12	627			MAZ_HUMAN.H11MO.0.A	1.240200e-02	
pos_patterns.pattern_13	354			ZN281_MOUSE.H11MO.0.A	6.040920e-05	
pos_patterns.pattern_14	337			ZN281_MOUSE.H11MO.0.A	7.705600e-04	
pos_patterns.pattern_15	298			SP1_HUMAN.H11MO.0.A	5.859200e-03	
pos_patterns.pattern_16	294			PRDM5_MOUSE.H11MO.0.A	1.000000e+00	
pos_patterns.pattern_17	155			SP1_HUMAN.H11MO.0.A	2.231530e-03	
pos_patterns.pattern_18	126			PRDM5_MOUSE.H11MO.0.A	7.308600e-02	
pos_patterns.pattern_19	115			ZNF384_MA1125.1	1.502460e-01	
pos_patterns.pattern_20	68			BARX1_MOUSE.H11MO.0.C	1.000000e+00	
neg_patterns.pattern_0	252			TN5_2	2.599430e-01	
neg_patterns.pattern_1	217			TN5_7	6.788550e-01	
neg_patterns.pattern_2	211			TN5_2	3.704520e-01	
neg_patterns.pattern_3	190			TN5_2	1.006620e-01	
neg_patterns.pattern_4	180			MAX_bHLH_1	6.772120e-03	
neg_patterns.pattern_5	150			SP2_HUMAN.H11MO.0.A	9.060400e-03	
neg_patterns.pattern_6	147			SP2_HUMAN.H11MO.0.A	1.204180e-01	
neg_patterns.pattern_7	102			KLF5_MOUSE.H11MO.0.A	8.517020e-02	

pattern	num_seqlets	cwm_fwd	cwm_rev	TOMTOM_match	TOMTOM_qval	TOMTOM_match_logo
neg_patterns.pattern_8	71	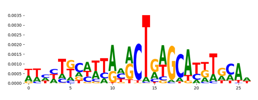	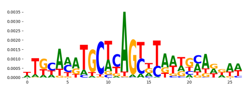	ZFX_MOUSE.H11MO.0.B	1.936040e-01	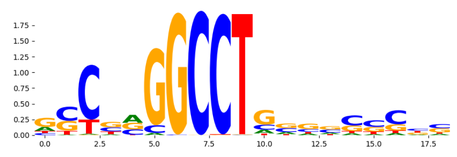
neg_patterns.pattern_9	71	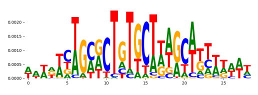	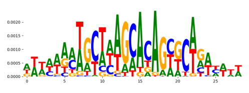	TN5_2	5.279140e-03	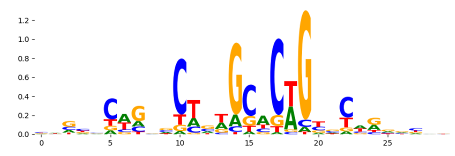
neg_patterns.pattern_10	30	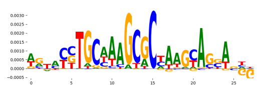	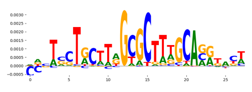	SMAD4_HUMAN.H11MO.0.B	3.478060e-01	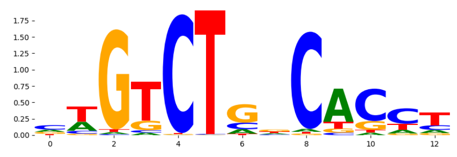
neg_patterns.pattern_11	27	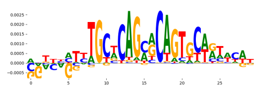	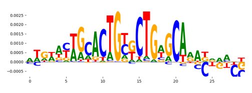	TN5_1	1.748220e-01	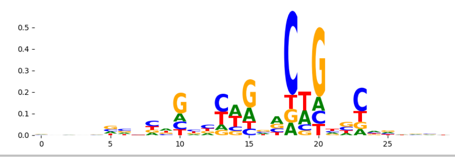
