# Supplementary material for: ChromBPNet: bias factorized, base-resolution deep learning models of chromatin accessibility reveal cis-regulatory sequence syntax, transcription factor footprints and regulatory variants
Source: bioRxiv. 2025 Jan 8:2024.12.25.630221. Preprint. [Version 2] doi: 10.1101/2024.12.25.630221 (PMC11741299; doi:10.1101/2024.12.25.630221)
Supplement: Supplement 4 [file media-4.zip › supplementary_files_3/hepg2_ATAC_bpnet_bias_model/hepg2_ATAC_raw_bpnet_bias_fold1_profile_modisco.pdf]

pattern	num_seqlets	cwm_fwd	cwm_rev
pos_patterns.pattern_0	8242	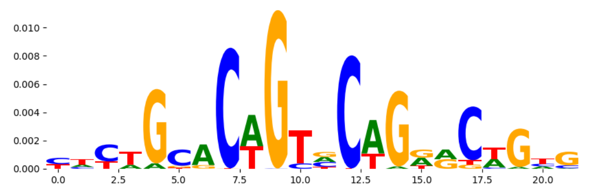	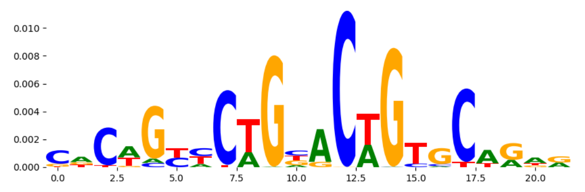
pos_patterns.pattern_1	5518	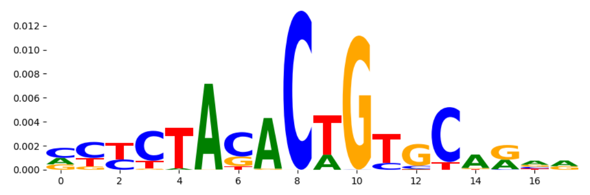	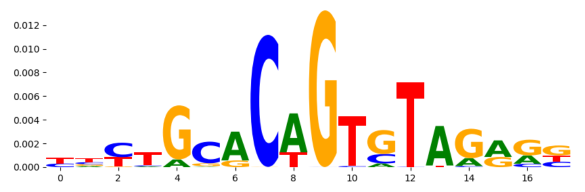
pos_patterns.pattern_2	3906	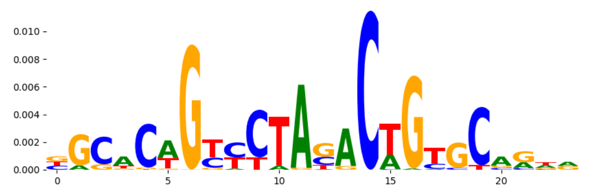	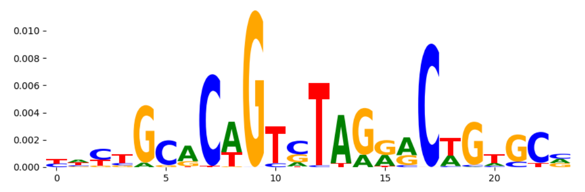
pos_patterns.pattern_3	3728	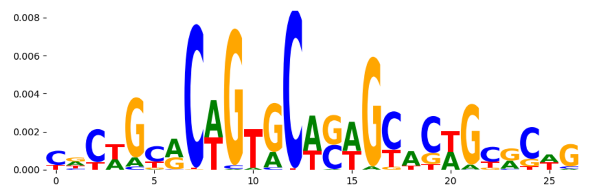	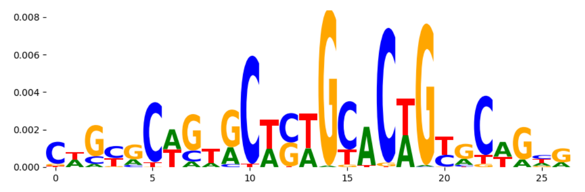
pos_patterns.pattern_4	2671	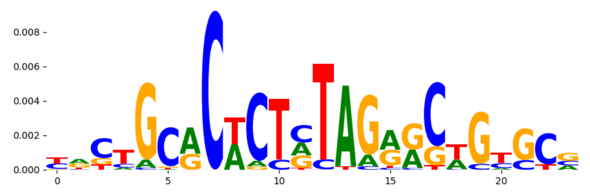	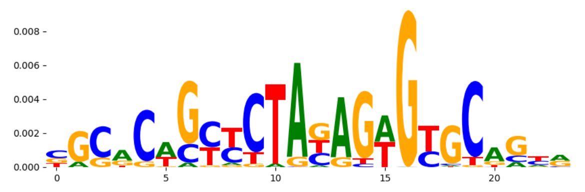
pos_patterns.pattern_5	1975	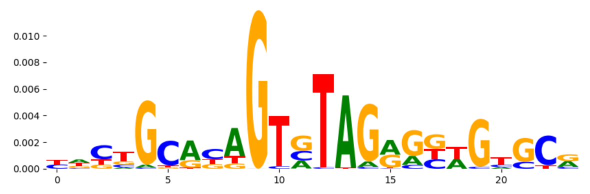	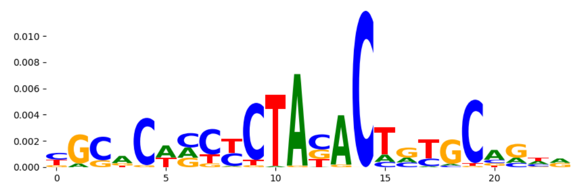
pos_patterns.pattern_6	1745	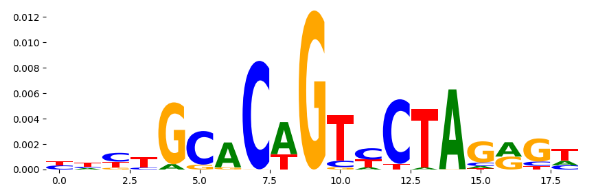	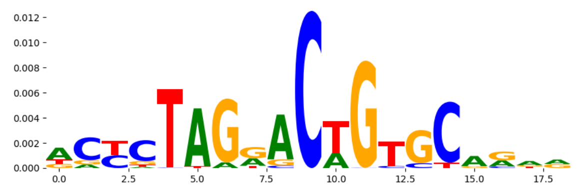
pos_patterns.pattern_7	718	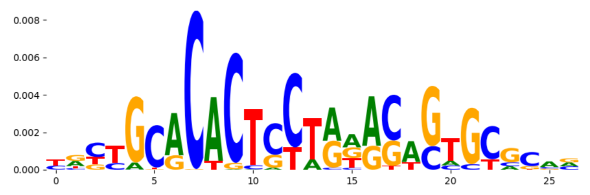	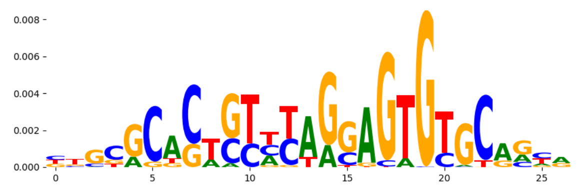
pos_patterns.pattern_8	352	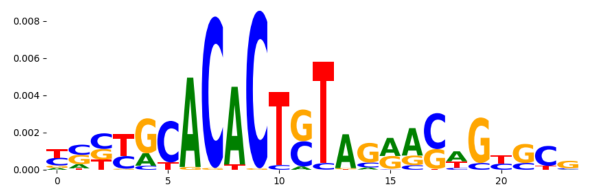	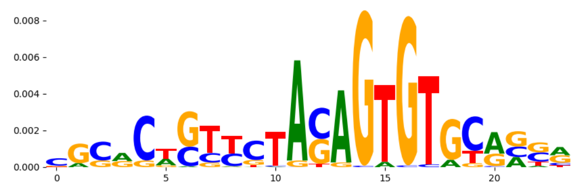
pos_patterns.pattern_9	335	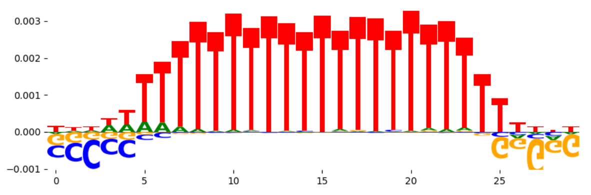	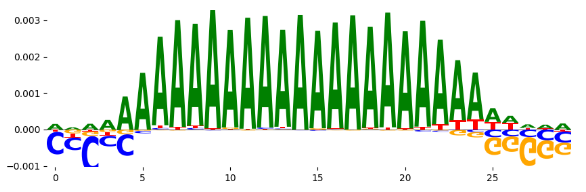
pos_patterns.pattern_10	85	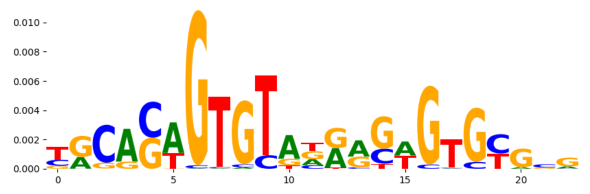	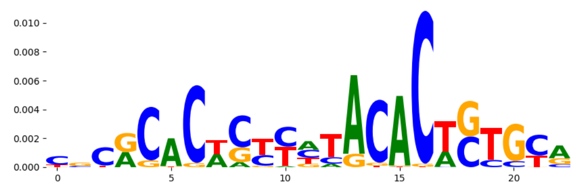
pos_patterns.pattern_11	67	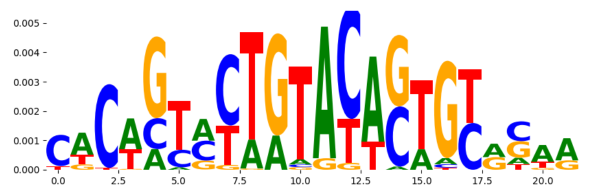	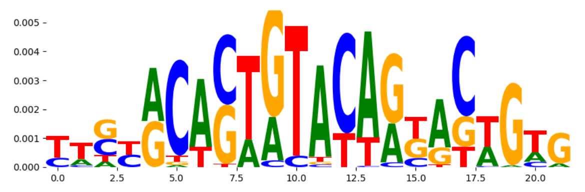
pos_patterns.pattern_12	48	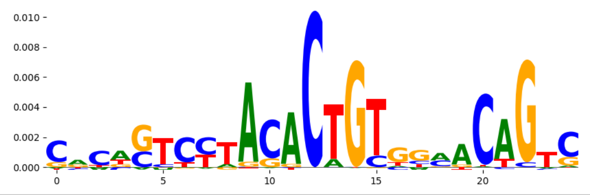	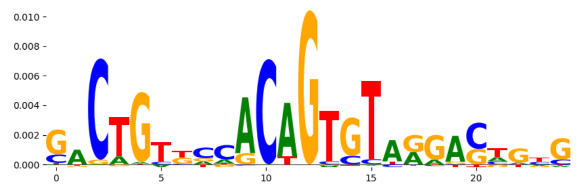
