# Supplementary material for: ChromBPNet: bias factorized, base-resolution deep learning models of chromatin accessibility reveal cis-regulatory sequence syntax, transcription factor footprints and regulatory variants
Source: bioRxiv. 2025 Jan 8:2024.12.25.630221. Preprint. [Version 2] doi: 10.1101/2024.12.25.630221 (PMC11741299; doi:10.1101/2024.12.25.630221)
Supplement: Supplement 4 [file media-4.zip › supplementary_files_3/hepg2_ATAC_bpnet_bias_model/hepg2_ATAC_raw_bpnet_bias_fold0_profile_modisco.pdf]

pattern	num_seqlets	cwm_fwd	cwm_rev	TOMTOM_match	TOMTOM_qval	TOMTOM_match_logo
pos_patterns.pattern_0	8439			TN5_1	2.849130e-09	
pos_patterns.pattern_1	5533			TN5_4	1.550140e-02	
pos_patterns.pattern_2	3691			TN5_2	1.503020e-18	
pos_patterns.pattern_3	3616			TN5_1	1.840240e-08	
pos_patterns.pattern_4	2606			TN5_3	9.716780e-02	
pos_patterns.pattern_5	904			TN5_3	2.618600e-03	
pos_patterns.pattern_6	877			TN5_3	1.583450e-06	
pos_patterns.pattern_7	759			TN5_3	4.058660e-11	
pos_patterns.pattern_8	758			TN5_7	1.226630e-03	
pos_patterns.pattern_9	592			TN5_3	1.456730e-06	
pos_patterns.pattern_10	533			TN5_3	1.134060e-04	
pos_patterns.pattern_11	359			TN5_4	2.923300e-06	
pos_patterns.pattern_12	353			PRDM6_HUMAN.H11MO.0.C	7.689710e-02	
pos_patterns.pattern_13	302			TN5_6	4.560730e-17	
pos_patterns.pattern_14	80			MTF1_HUMAN.H11MO.0.C	5.953400e-01	
pos_patterns.pattern_15	74			TN5_8	2.289830e-04	
pos_patterns.pattern_16	72			EGR2_HUMAN.H11MO.0.A	1.000000e+00	
pos_patterns.pattern_17	45			TN5_7	5.396470e-06	
