# Supplementary material for: ChromBPNet: bias factorized, base-resolution deep learning models of chromatin accessibility reveal cis-regulatory sequence syntax, transcription factor footprints and regulatory variants
Source: bioRxiv. 2025 Jan 8:2024.12.25.630221. Preprint. [Version 2] doi: 10.1101/2024.12.25.630221 (PMC11741299; doi:10.1101/2024.12.25.630221)
Supplement: Supplement 4 [file media-4.zip › supplementary_files_3/hepg2_ATAC_bpnet_bias_model/hepg2_ATAC_raw_bpnet_bias_fold3_profile_modisco.pdf]

pattern	num_seqlets	cwm_fwd	cwm_rev	TOMTOM_match	TOMTOM_qval	TOMTOM_match_logo
pos_patterns.pattern_0	12329	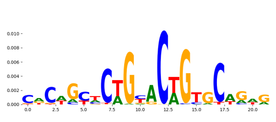	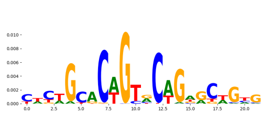	TN5_2	6.058040e-09	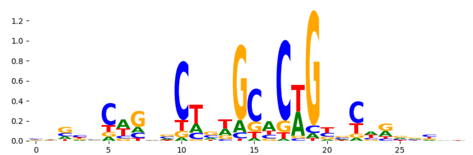
pos_patterns.pattern_1	6736	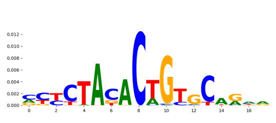	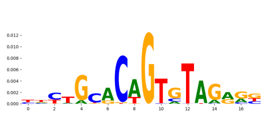	TN5_4	2.121400e-02	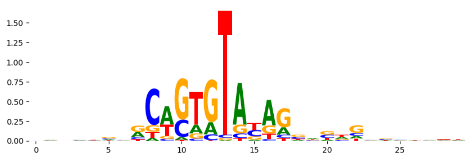
pos_patterns.pattern_2	3753	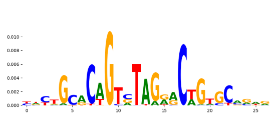	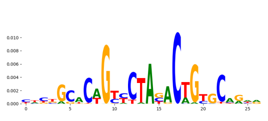	TN5_1	2.584820e-08	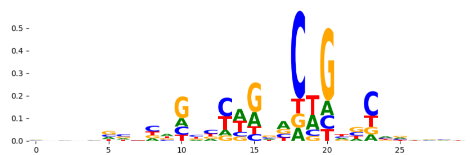
pos_patterns.pattern_3	1653	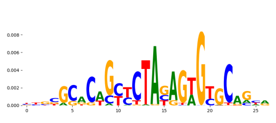	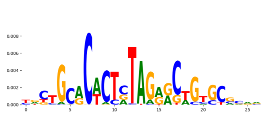	TN5_3	1.433350e-10	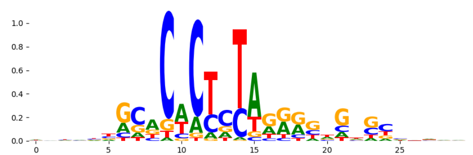
pos_patterns.pattern_4	1349	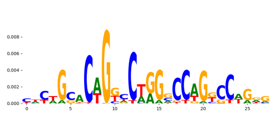	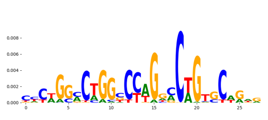	TN5_1	4.617570e-05	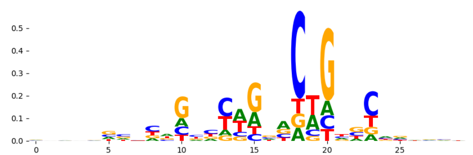
pos_patterns.pattern_5	1164	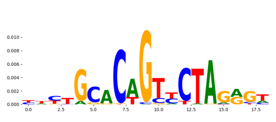	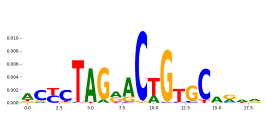	TN5_7	2.616660e-02	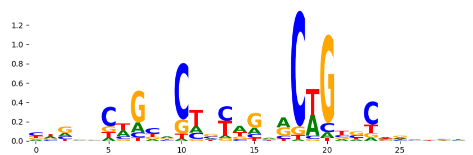
pos_patterns.pattern_6	592	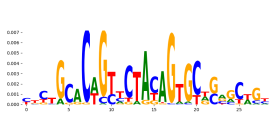	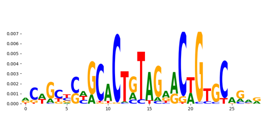	TN5_7	1.742780e-10	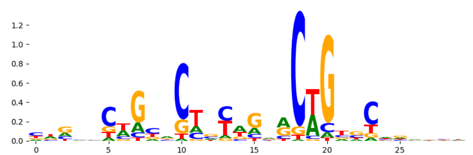
pos_patterns.pattern_7	572	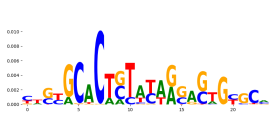	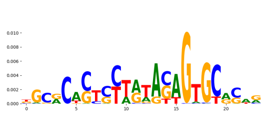	TN5_4	4.545890e-04	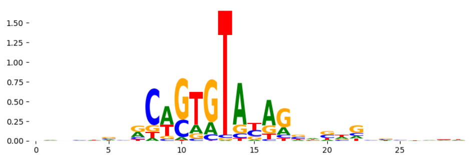
pos_patterns.pattern_8	386	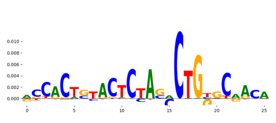	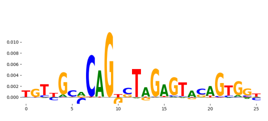	TN5_6	2.613380e-13	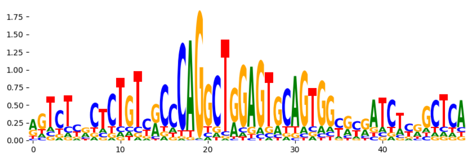
pos_patterns.pattern_9	373	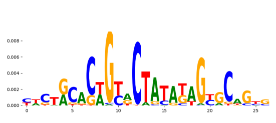	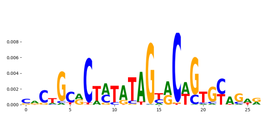	TN5_3	1.141960e-02	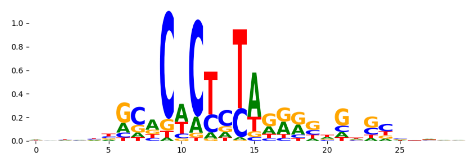
pos_patterns.pattern_10	265	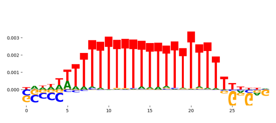	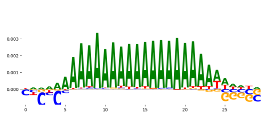	ZNF384_MA1125.1	6.346600e-02	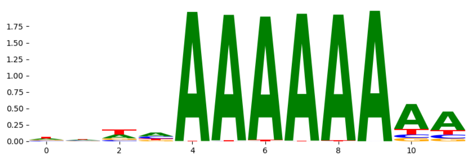
pos_patterns.pattern_11	66	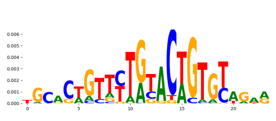	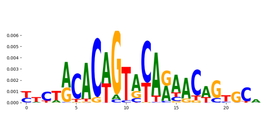	TN5_2	6.194690e-02	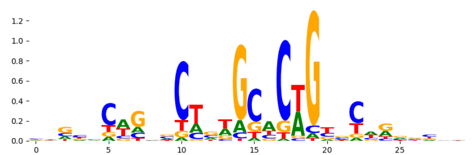
pos_patterns.pattern_12	20	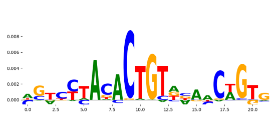	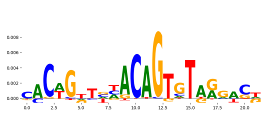	TN5_7	2.104270e-01	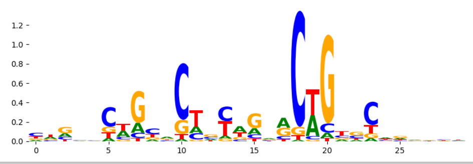
