# Supplementary material for: ChromBPNet: bias factorized, base-resolution deep learning models of chromatin accessibility reveal cis-regulatory sequence syntax, transcription factor footprints and regulatory variants
Source: bioRxiv. 2025 Jan 8:2024.12.25.630221. Preprint. [Version 2] doi: 10.1101/2024.12.25.630221 (PMC11741299; doi:10.1101/2024.12.25.630221)
Supplement: Supplement 4 [file media-4.zip › supplementary_files_3/hepg2_ATAC_bpnet_bias_model/hepg2_ATAC_raw_bpnet_bias_fold2_profile_modisco.pdf]

pattern	num_seqlets	cwm_fwd	cwm_rev	TOMTOM_match	TOMTOM_qval	TOMTOM_match_logo
pos_patterns.pattern_0	10489	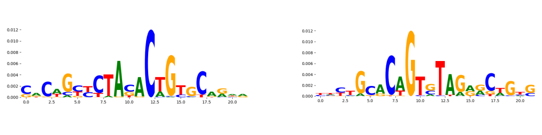		TN5_2	1.259030e-03	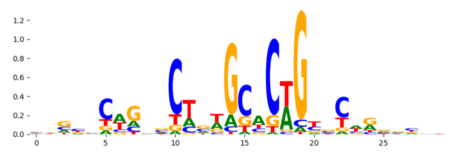
pos_patterns.pattern_1	8038	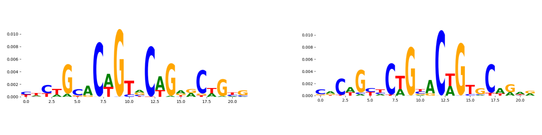		TN5_1	1.409980e-09	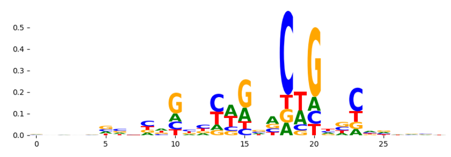
pos_patterns.pattern_2	4083	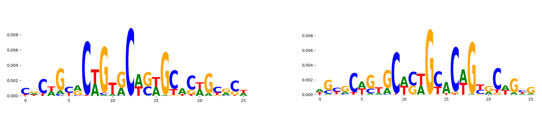		TN5_2	6.249720e-12	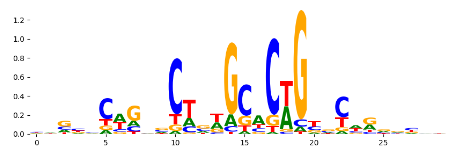
pos_patterns.pattern_3	1486	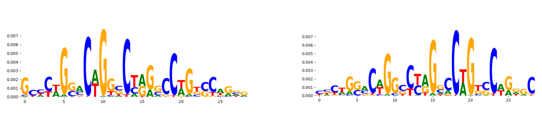		TN5_1	1.672570e-03	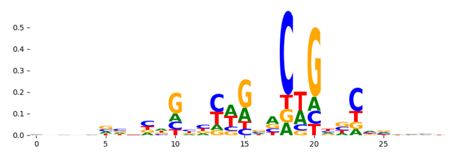
pos_patterns.pattern_4	1076	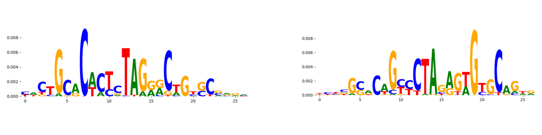		TN5_3	3.619950e-06	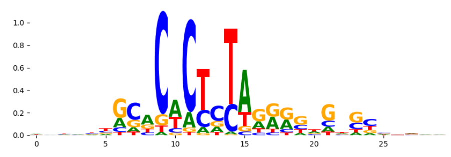
pos_patterns.pattern_5	976	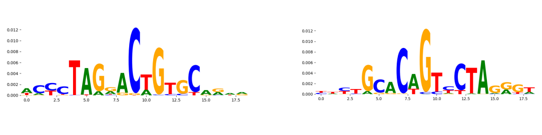		TN5_1	3.774520e-02	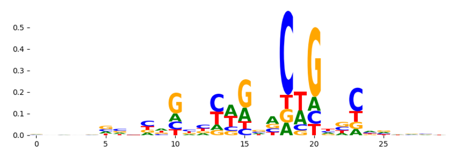
pos_patterns.pattern_6	822	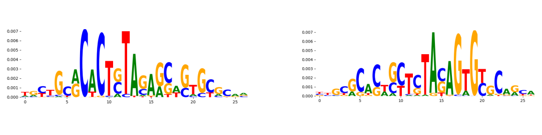		TN5_3	2.721520e-12	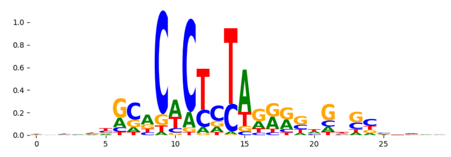
pos_patterns.pattern_7	676	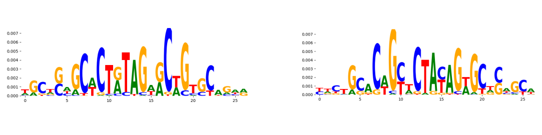		TN5_7	2.487450e-06	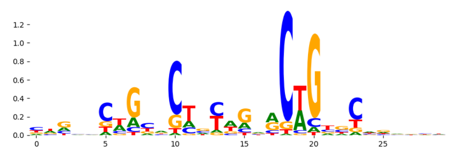
pos_patterns.pattern_8	481	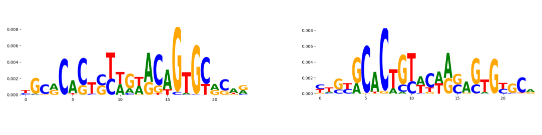		TN5_3	6.509280e-06	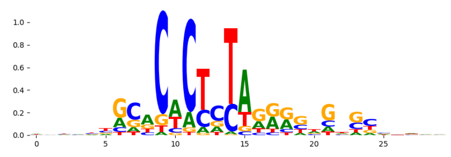
pos_patterns.pattern_9	408	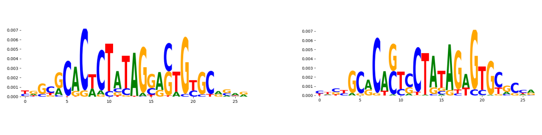		TN5_3	9.950710e-07	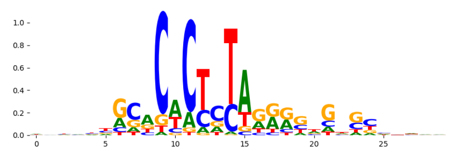
pos_patterns.pattern_10	375	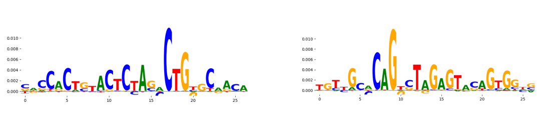		TN5_6	5.347200e-17	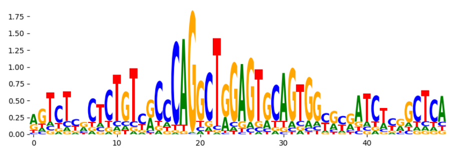
pos_patterns.pattern_11	373	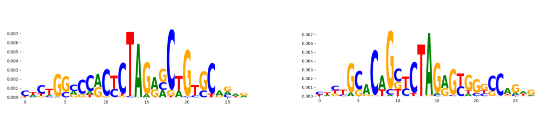		TN5_1	2.307860e-03	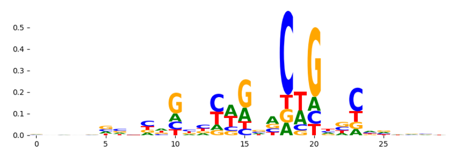
pos_patterns.pattern_12	264	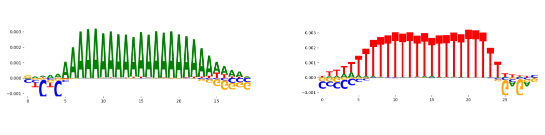		PRDM6_HUMAN.H11MO.0.C	8.077350e-02	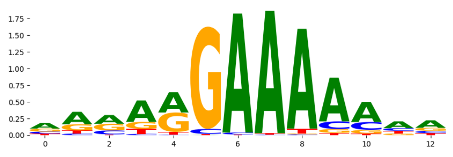
pos_patterns.pattern_13	62	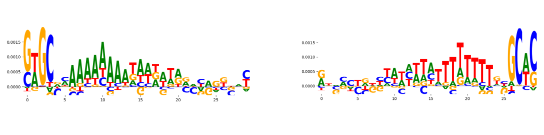		MEF2A_MOUSE.H11MO.0.A	9.872390e-02	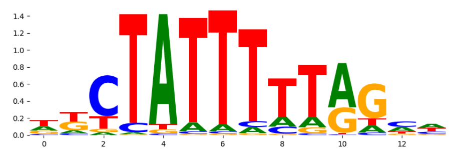
pos_patterns.pattern_14	24	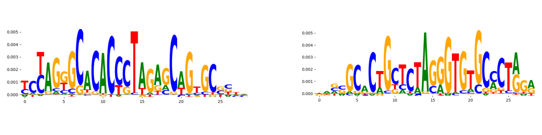		TN5_3	4.916390e-03	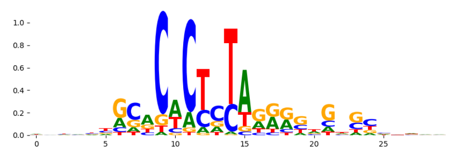
pos_patterns.pattern_15	24	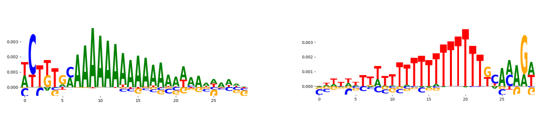		ZNF384_MA1125.1	6.650060e-03	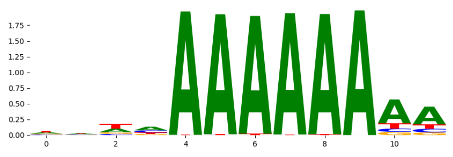
pos_patterns.pattern_16	22	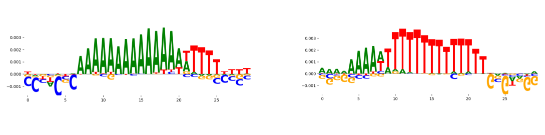		ZNF384_MA1125.1	3.137470e-02	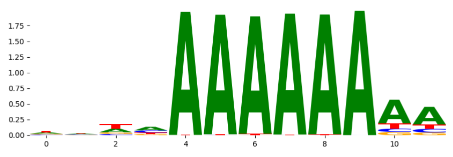
pos_patterns.pattern_17	20	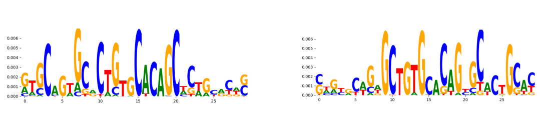		TN5_2	1.072050e-02	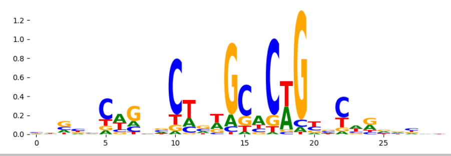
