# Supplementary material for: ChromBPNet: bias factorized, base-resolution deep learning models of chromatin accessibility reveal cis-regulatory sequence syntax, transcription factor footprints and regulatory variants
Source: bioRxiv. 2025 Jan 8:2024.12.25.630221. Preprint. [Version 2] doi: 10.1101/2024.12.25.630221 (PMC11741299; doi:10.1101/2024.12.25.630221)
Supplement: Supplement 4 [file media-4.zip › supplementary_files_3/hepg2_ATAC_bpnet_bias_model/hepg2_ATAC_raw_bpnet_bias_fold4_profile_modisco.pdf]

pattern	num_seqlets	cwm_fwd	cwm_rev	TOMTOM_match	TOMTOM_qval	TOMTOM_match_logo
pos_patterns.pattern_0	7951	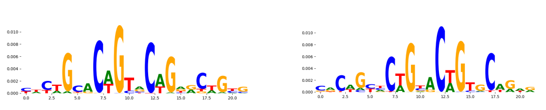		TN5_1	1.121210e-09	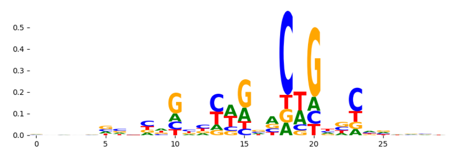
pos_patterns.pattern_1	6047	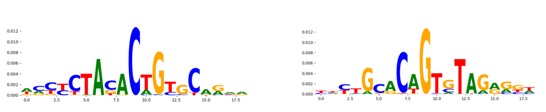		TN5_8	4.005420e-02	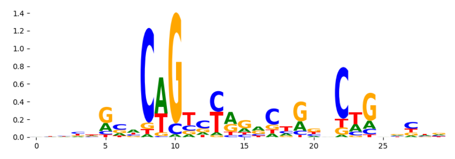
pos_patterns.pattern_2	4203	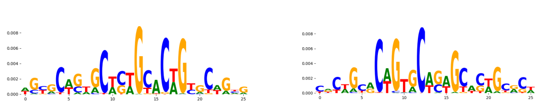		TN5_2	1.132310e-16	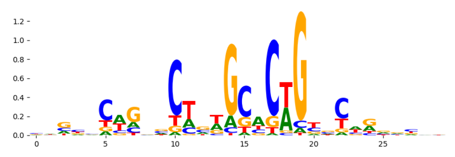
pos_patterns.pattern_3	4050	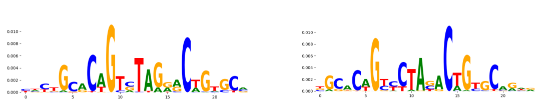		TN5_1	1.441260e-05	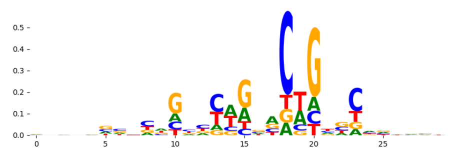
pos_patterns.pattern_4	1755	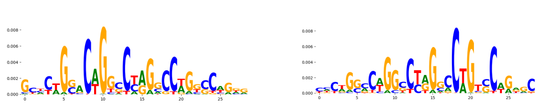		TN5_1	1.550630e-03	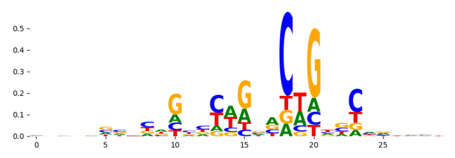
pos_patterns.pattern_5	1283	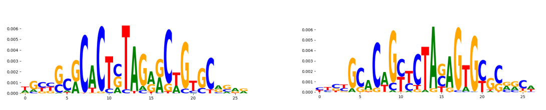		TN5_3	1.283030e-08	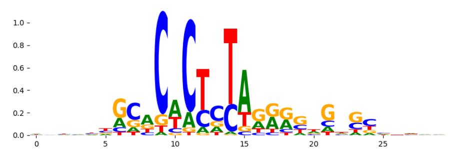
pos_patterns.pattern_6	827	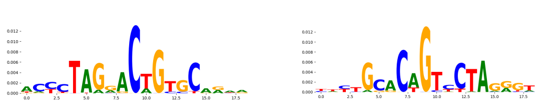		TN5_1	4.122530e-02	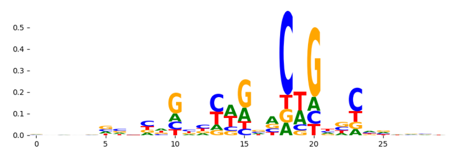
pos_patterns.pattern_7	737	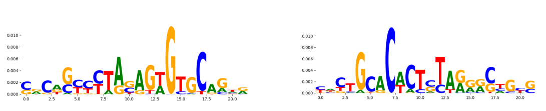		TN5_3	5.631160e-08	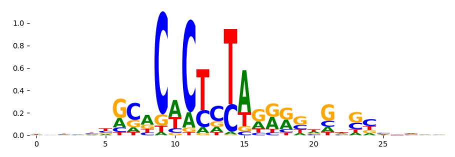
pos_patterns.pattern_8	650	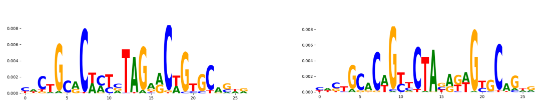		TN5_3	1.579030e-08	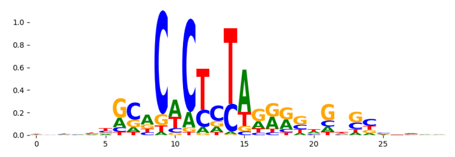
pos_patterns.pattern_9	582	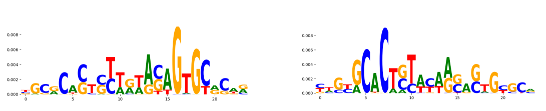		TN5_4	1.092690e-03	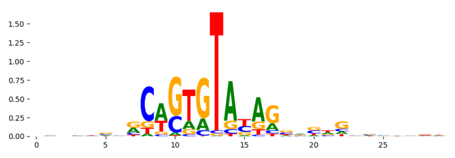
pos_patterns.pattern_10	443	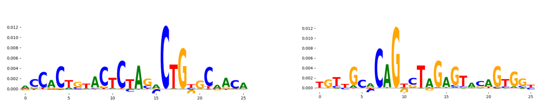		TN5_6	2.224700e-15	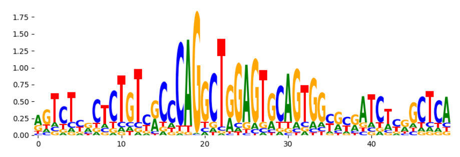
pos_patterns.pattern_11	407	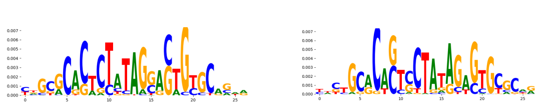		TN5_3	2.228240e-06	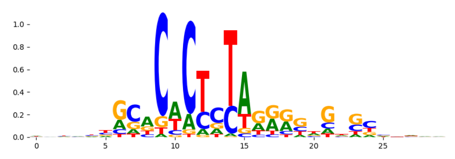
pos_patterns.pattern_12	249	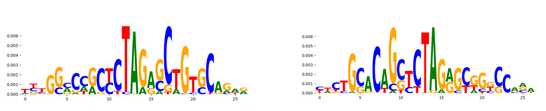		TN5_3	9.178750e-02	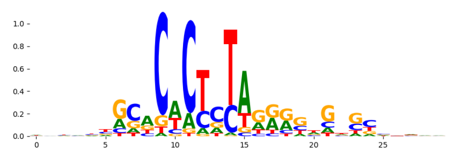
pos_patterns.pattern_13	248	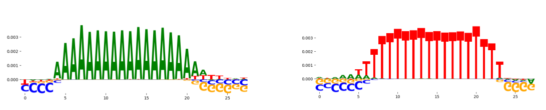		PRDM6_HUMAN.H11MO.0.C	5.646740e-02	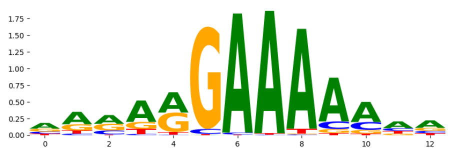
pos_patterns.pattern_14	97	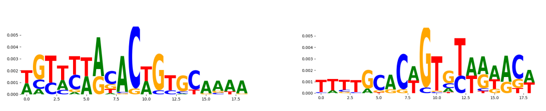		TN5_1	1.857470e-03	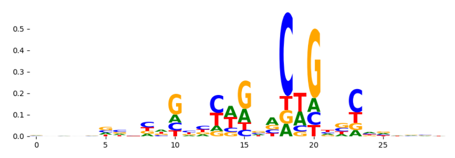
pos_patterns.pattern_15	68	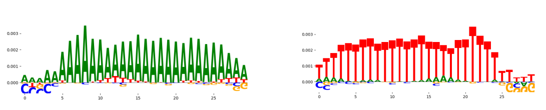		ZNF384_MA1125.1	7.701890e-02	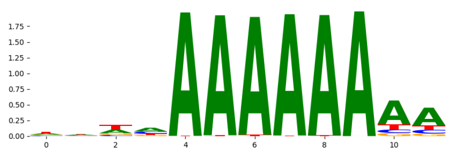
pos_patterns.pattern_16	50	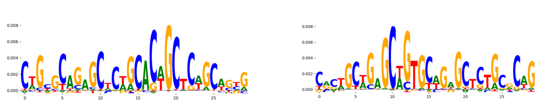		TN5_2	4.346460e-02	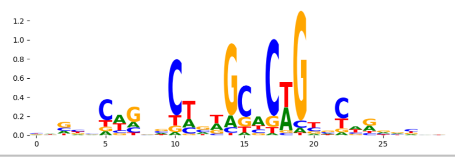
