# Supplementary material for: ChromBPNet: bias factorized, base-resolution deep learning models of chromatin accessibility reveal cis-regulatory sequence syntax, transcription factor footprints and regulatory variants
Source: bioRxiv. 2025 Jan 8:2024.12.25.630221. Preprint. [Version 2] doi: 10.1101/2024.12.25.630221 (PMC11741299; doi:10.1101/2024.12.25.630221)

pattern	num_seqlets	cwm_fwd	cwm_rev	TOMTOM_match	TOMTOM_qval	TOMTOM_match_logo
pos_patterns.pattern_0	8644			DNASE_1	1.563260e-05	
pos_patterns.pattern_1	7404			DNASE_2	4.595570e-05	
pos_patterns.pattern_2	1744			DNASE_3	4.614350e-05	
pos_patterns.pattern_3	1155			ZNF384_MA1125.1	8.986210e-02	
pos_patterns.pattern_4	604			SP1_MOUSE.H11MO.0.A	7.067970e-08	
pos_patterns.pattern_5	433			ZN770_HUMAN.H11MO.0.C	7.469870e-07	
pos_patterns.pattern_6	428			ZNF384_MA1125.1	1.577380e-02	
pos_patterns.pattern_7	424			ZNF384_MA1125.1	6.927550e-03	
pos_patterns.pattern_8	406			ZNF384_MA1125.1	4.129360e-02	
pos_patterns.pattern_9	328			MEF2B_HUMAN.H11MO.0.A	2.334880e-01	
pos_patterns.pattern_10	294			SP2_HUMAN.H11MO.0.A	5.131330e-04	
pos_patterns.pattern_11	278			DNASE_5	8.018960e-04	
pos_patterns.pattern_12	229			FOXC1_forkhead_1	3.569100e-02	
pos_patterns.pattern_13	161			ITF2_HUMAN.H11MO.0.C	1.000000e+00	
pos_patterns.pattern_14	139			FOXK1_forkhead_1	1.814010e-01	
pos_patterns.pattern_15	133			ZN770_HUMAN.H11MO.0.C	3.435680e-08	
pos_patterns.pattern_16	129			HINFP1_C2H2_2	6.911260e-01	
pos_patterns.pattern_17	115			TN5_6	2.484630e-10	
pos_patterns.pattern_18	109			TN5_6	1.937960e-10	
pos_patterns.pattern_19	104			ZN770_HUMAN.H11MO.0.C	2.052260e-08	
pos_patterns.pattern_20	99			HINFP1_C2H2_2	6.626120e-01	
pos_patterns.pattern_21	81			DNASE_5	1.908840e-03	
pos_patterns.pattern_22	49			ITF2_HUMAN.H11MO.0.C	9.048080e-01	
pos_patterns.pattern_23	47			RARA_HUMAN.H11MO.0.A	3.262920e-01	
pos_patterns.pattern_24	45			DNASE_5	1.605470e-03	
pos_patterns.pattern_25	36			TN5_6	6.432370e-17	
pos_patterns.pattern_26	26			DNASE_5	2.565630e-03	
pos_patterns.pattern_27	20			RREB1_MA0073.1	1.000000e+00	

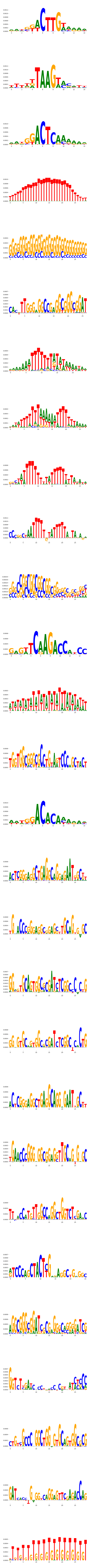
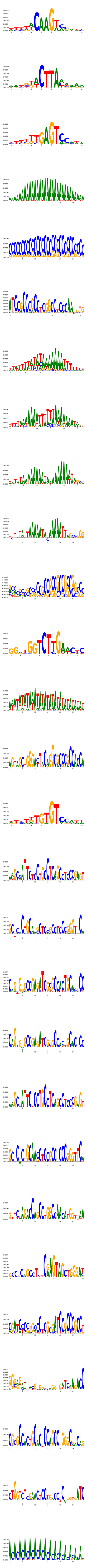
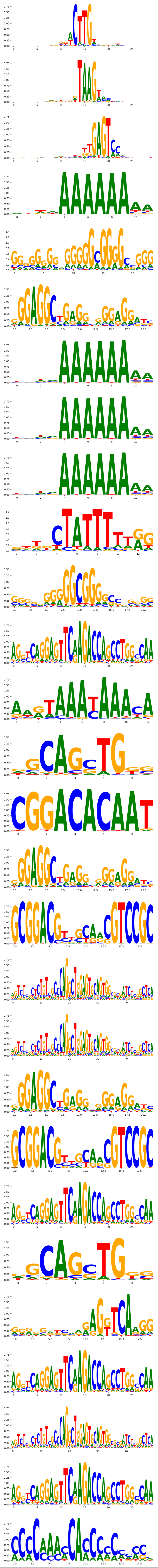

Supplement: Supplement 4 [file media-4.zip › supplementary_files_3/hepg2_DNASE_bpnet_bias_model/hepg2_DNASE_raw_bpnet_bias_fold4_profile_modisco.pdf]
