# Supplementary material for: ChromBPNet: bias factorized, base-resolution deep learning models of chromatin accessibility reveal cis-regulatory sequence syntax, transcription factor footprints and regulatory variants
Source: bioRxiv. 2025 Jan 8:2024.12.25.630221. Preprint. [Version 2] doi: 10.1101/2024.12.25.630221 (PMC11741299; doi:10.1101/2024.12.25.630221)
Supplement: Supplement 4 [file media-4.zip › supplementary_files_3/hepg2_DNASE_bpnet_bias_model/hepg2_DNASE_raw_bpnet_bias_fold1_counts_modisco.pdf]

pattern	num_seqlets	cwm_fwd	cwm_rev	TOMTOM_match	TOMTOM_qval	TOMTOM_match_logo
pos_patterns.pattern_0	9078			DNASE_1	1.175780e-03	
pos_patterns.pattern_1	6751			DNASE_2	6.828250e-06	
pos_patterns.pattern_2	2755			DNASE_3	6.802010e-06	
pos_patterns.pattern_3	937			PRDM6_HUMAN.H11MO.0.C	7.674420e-02	
pos_patterns.pattern_4	691			SP1_HUMAN.H11MO.0.A	9.222830e-05	
pos_patterns.pattern_5	480			MEF2B_HUMAN.H11MO.0.A	1.850760e-01	
pos_patterns.pattern_6	480			SP1_MOUSE.H11MO.0.A	4.801290e-08	
pos_patterns.pattern_7	479			ZNF384_MA1125.1	6.805550e-05	
pos_patterns.pattern_8	451			ZNF384_MA1125.1	2.566890e-03	
pos_patterns.pattern_9	435			ZNF384_MA1125.1	3.180030e-02	
pos_patterns.pattern_10	255			SP1_HUMAN.H11MO.0.A	6.240570e-01	
pos_patterns.pattern_11	242			SP2_HUMAN.H11MO.0.A	1.087770e-04	
pos_patterns.pattern_12	211			TN5_6	7.072850e-18	
pos_patterns.pattern_13	207			DNASE_2	4.810540e-01	
pos_patterns.pattern_14	200			ZN770_HUMAN.H11MO.0.C	2.351320e-08	
pos_patterns.pattern_15	180			ZN770_HUMAN.H11MO.0.C	1.758660e-08	
pos_patterns.pattern_16	122			ZN449_HUMAN.H11MO.0.C	6.649680e-01	
pos_patterns.pattern_17	79			ITF2_HUMAN.H11MO.0.C	8.150400e-01	
pos_patterns.pattern_18	60			KLF4_MA0039.3	7.845210e-01	
pos_patterns.pattern_19	54			DNASE_2	1.000000e+00	
pos_patterns.pattern_20	41			PRDM6_HUMAN.H11MO.0.C	9.679110e-02	
pos_patterns.pattern_21	35			ZNF384_MA1125.1	2.287250e-01	
pos_patterns.pattern_22	30			DNASE_3	6.597560e-01	
pos_patterns.pattern_23	28			DNASE_4	1.000000e+00	
neg_patterns.pattern_0	34			Foxj3.mouse_forkhead_4	1.192890e-01	
neg_patterns.pattern_1	27			GATA6_MA1104.1	6.350240e-02	
neg_patterns.pattern_2	26			EGR1_HUMAN.H11MO.0.A	6.696370e-03	
neg_patterns.pattern_3	26			ZN281_HUMAN.H11MO.0.A	1.594680e-04	
