# Supplementary material for: ChromBPNet: bias factorized, base-resolution deep learning models of chromatin accessibility reveal cis-regulatory sequence syntax, transcription factor footprints and regulatory variants
Source: bioRxiv. 2025 Jan 8:2024.12.25.630221. Preprint. [Version 2] doi: 10.1101/2024.12.25.630221 (PMC11741299; doi:10.1101/2024.12.25.630221)
Supplement: Supplement 4 [file media-4.zip › supplementary_files_3/hepg2_DNASE_bpnet_bias_model/hepg2_DNASE_raw_bpnet_bias_fold0_profile_modisco.pdf]

pattern	num_seqlets	cwm_fwd	cwm_rev	TOMTOM_match	TOMTOM_qval	TOMTOM_match_logo
pos_patterns.pattern_0	8153			DNASE_1	7.557850e-06	
pos_patterns.pattern_1	6297			DNASE_2	2.269380e-06	
pos_patterns.pattern_2	1408			DNASE_3	1.066990e-06	
pos_patterns.pattern_3	973			PRDM6_HUMAN.H11MO.0.C	7.770000e-02	
pos_patterns.pattern_4	751			KLF6_HUMAN.H11MO.0.A	4.581690e-02	
pos_patterns.pattern_5	663			MEF2B_HUMAN.H11MO.0.A	2.418480e-01	
pos_patterns.pattern_6	615			ZNF384_MA1125.1	4.853220e-03	
pos_patterns.pattern_7	530			DNASE_4	9.556290e-03	
pos_patterns.pattern_8	447			SP1_MOUSE.H11MO.0.A	3.542410e-09	
pos_patterns.pattern_9	427			ZNF384_MA1125.1	1.599120e-02	
pos_patterns.pattern_10	328			SP2_HUMAN.H11MO.0.A	5.814950e-06	
pos_patterns.pattern_11	307			DNASE_5	1.986510e-15	
pos_patterns.pattern_12	304			DNASE_5	2.254080e-05	
pos_patterns.pattern_13	302			FOXK1_forkhead_1	9.335290e-01	
pos_patterns.pattern_14	230			ZN770_HUMAN.H11MO.0.C	5.335500e-01	
pos_patterns.pattern_15	198			FOXC1_forkhead_1	1.091330e-01	
pos_patterns.pattern_16	175			TN5_6	2.307430e-20	
pos_patterns.pattern_17	117			DNASE_6	7.359140e-20	
pos_patterns.pattern_18	106			PRDM6_HUMAN.H11MO.0.C	3.849030e-02	
pos_patterns.pattern_19	94			ZN770_HUMAN.H11MO.0.C	6.576460e-01	
pos_patterns.pattern_20	85			KLF1_HUMAN.H11MO.0.A	9.492460e-01	
pos_patterns.pattern_21	58			ZN770_HUMAN.H11MO.0.C	3.368640e-08	
pos_patterns.pattern_22	41			IRF3_HUMAN.H11MO.0.B	1.958740e-02	
pos_patterns.pattern_23	32			RORC_MA1151.1	1.000000e+00	
pos_patterns.pattern_24	20			ITF2_HUMAN.H11MO.0.C	1.000000e+00	
pos_patterns.pattern_25	20			ZN431_MOUSE.H11MO.0.C	1.000000e+00	
neg_patterns.pattern_0	46			ZBT17_MOUSE.H11MO.0.A	1.353000e-03	
neg_patterns.pattern_1	29			ZNF384_MA1125.1	5.826750e-01	
neg_patterns.pattern_2	21			GATA3_HUMAN.H11MO.0.A	1.457100e-01	
