# Supplementary material for: ChromBPNet: bias factorized, base-resolution deep learning models of chromatin accessibility reveal cis-regulatory sequence syntax, transcription factor footprints and regulatory variants
Source: bioRxiv. 2025 Jan 8:2024.12.25.630221. Preprint. [Version 2] doi: 10.1101/2024.12.25.630221 (PMC11741299; doi:10.1101/2024.12.25.630221)
Supplement: Supplement 4 [file media-4.zip › supplementary_files_3/hepg2_DNASE_bpnet_bias_model/hepg2_DNASE_raw_bpnet_bias_fold3_profile_modisco.pdf]

pattern	num_seqlets	cwm_fwd	cwm_rev	TOMTOM_match	TOMTOM_qval	TOMTOM_match_logo
pos_patterns.pattern_0	8341			DNASE_1	6.687330e-06	
pos_patterns.pattern_1	7110			DNASE_2	5.230500e-06	
pos_patterns.pattern_2	1704			DNASE_3	1.665470e-05	
pos_patterns.pattern_3	1004			PRDM6_HUMAN.H11MO.0.C	8.248720e-02	
pos_patterns.pattern_4	584			MEF2B_HUMAN.H11MO.0.A	1.442120e-01	
pos_patterns.pattern_5	568			DNASE_2	1.277190e-03	
pos_patterns.pattern_6	560			ZNF384_MA1125.1	2.859830e-01	
pos_patterns.pattern_7	500			SP2_HUMAN.H11MO.0.A	1.941950e-06	
pos_patterns.pattern_8	443			SP1_MOUSE.H11MO.0.A	5.340220e-08	
pos_patterns.pattern_9	352			RARG_HUMAN.H11MO.0.B	1.000000e+00	
pos_patterns.pattern_10	293			DNASE_5	1.097480e-03	
pos_patterns.pattern_11	286			ZNF384_MA1125.1	6.332730e-03	
pos_patterns.pattern_12	236			ZN770_HUMAN.H11MO.0.C	8.896830e-08	
pos_patterns.pattern_13	228			TN5_6	1.509230e-18	
pos_patterns.pattern_14	206			ZN770_HUMAN.H11MO.0.C	2.245180e-08	
pos_patterns.pattern_15	204			FOXK1_forkhead_1	6.119230e-01	
pos_patterns.pattern_16	105			KLF4_MA0039.3	6.748550e-01	
pos_patterns.pattern_17	92			SP1_MOUSE.H11MO.0.A	1.599910e-01	
pos_patterns.pattern_18	71			ITF2_HUMAN.H11MO.0.C	1.000000e+00	
pos_patterns.pattern_19	69			DNASE_2	6.228950e-02	
pos_patterns.pattern_20	64			FOXB1_forkhead_2	8.627750e-01	
pos_patterns.pattern_21	63			PRDM6_HUMAN.H11MO.0.C	6.924050e-02	
pos_patterns.pattern_22	34			NaN	NaN	
pos_patterns.pattern_23	33			SMCA1_HUMAN.H11MO.0.C	1.000000e+00	
pos_patterns.pattern_24	24			DNASE_4	1.619750e-01	
