# Supplementary material for: ChromBPNet: bias factorized, base-resolution deep learning models of chromatin accessibility reveal cis-regulatory sequence syntax, transcription factor footprints and regulatory variants
Source: bioRxiv. 2025 Jan 8:2024.12.25.630221. Preprint. [Version 2] doi: 10.1101/2024.12.25.630221 (PMC11741299; doi:10.1101/2024.12.25.630221)
Supplement: Supplement 4 [file media-4.zip › supplementary_files_3/hepg2_DNASE_bpnet_bias_model/hepg2_DNASE_raw_bpnet_bias_fold2_counts_modisco.pdf]

pattern	num_seqlets	cwm_fwd	cwm_rev	TOMTOM_match	TOMTOM_qval	TOMTOM_match_logo
pos_patterns.pattern_0	8284			DNASE_1	2.248810e-03	
pos_patterns.pattern_1	7048			DNASE_2	8.051420e-05	
pos_patterns.pattern_2	1930			DNASE_3	2.153620e-04	
pos_patterns.pattern_3	1100			SP2_HUMAN.H11MO.0.A	2.449150e-05	
pos_patterns.pattern_4	1011			PRDM6_HUMAN.H11MO.0.C	9.279250e-02	
pos_patterns.pattern_5	673			ZNF384_MA1125.1	2.531240e-02	
pos_patterns.pattern_6	669			ZNF384_MA1125.1	1.176740e-02	
pos_patterns.pattern_7	461			SP1_MOUSE.H11MO.0.A	2.062780e-07	
pos_patterns.pattern_8	422			MEF2B_HUMAN.H11MO.0.A	2.217960e-01	
pos_patterns.pattern_9	322			FOXC1_forkhead_1	6.186800e-02	
pos_patterns.pattern_10	306			TN5_6	3.650790e-19	
pos_patterns.pattern_11	256			ZN770_HUMAN.H11MO.0.C	3.455030e-08	
pos_patterns.pattern_12	242			RARG_HUMAN.H11MO.0.B	7.042440e-01	
pos_patterns.pattern_13	191			DNASE_5	1.644540e-15	
pos_patterns.pattern_14	155			KLF4_MA0039.3	8.473110e-01	
pos_patterns.pattern_15	151			ZN770_HUMAN.H11MO.0.C	2.559420e-08	
pos_patterns.pattern_16	56			IKZF1_HUMAN.H11MO.0.C	7.480660e-01	
pos_patterns.pattern_17	53			ZN770_HUMAN.H11MO.0.C	1.505930e-06	
pos_patterns.pattern_18	51			ZN322_HUMAN.H11MO.0.B	1.000000e+00	
pos_patterns.pattern_19	48			DNASE_2	2.637300e-01	
pos_patterns.pattern_20	48			EWSR1-FLI1_MA0149.1	8.131390e-02	
pos_patterns.pattern_21	43			ITF2_HUMAN.H11MO.0.C	1.000000e+00	
pos_patterns.pattern_22	30			ZNF384_MA1125.1	3.726430e-01	
neg_patterns.pattern_0	41			MAZ_HUMAN.H11MO.0.A	6.652480e-05	
neg_patterns.pattern_1	22			FOXJ3_HUMAN.H11MO.0.A	7.529350e-02	
neg_patterns.pattern_2	22			ZNF384_MA1125.1	6.889630e-01	
neg_patterns.pattern_3	21			ZNF384_MA1125.1	1.970300e-02	
