# Supplementary material for: ChromBPNet: bias factorized, base-resolution deep learning models of chromatin accessibility reveal cis-regulatory sequence syntax, transcription factor footprints and regulatory variants
Source: bioRxiv. 2025 Jan 8:2024.12.25.630221. Preprint. [Version 2] doi: 10.1101/2024.12.25.630221 (PMC11741299; doi:10.1101/2024.12.25.630221)
Supplement: Supplement 4 [file media-4.zip › supplementary_files_3/k562_DNASE_bpnet_bias_model/k562_DNASE_raw_bpnet_bias_fold2_counts_modisco.pdf]

| pattern | num_seqlets | cwm_fwd | cwm_rev |
|---|---|---|---|
| pos_patterns.pattern_0 | 1900 | 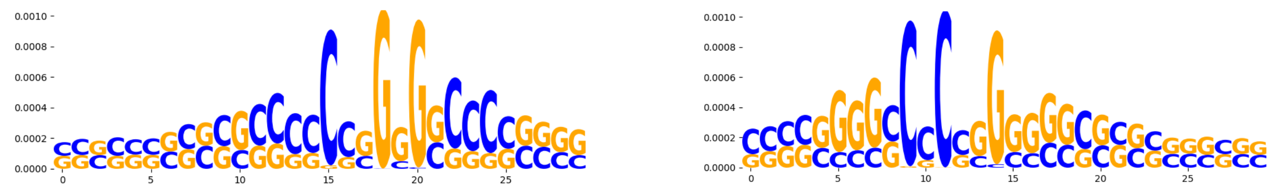 | |
| pos_patterns.pattern_1 | 1605 | 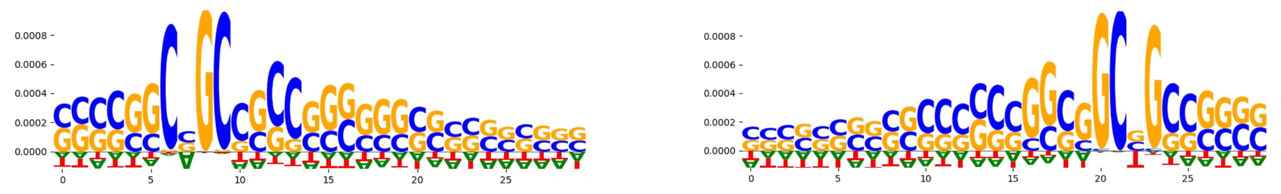 | |
| pos_patterns.pattern_2 | 1295 | 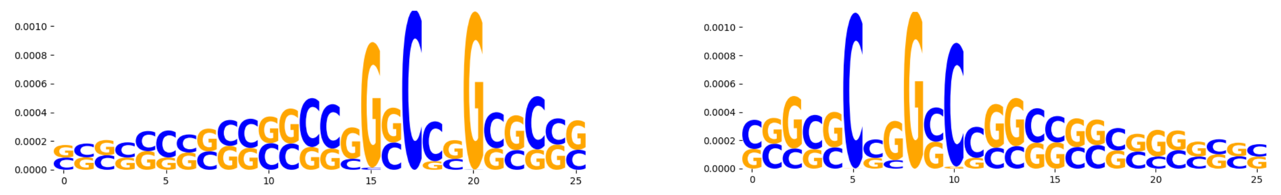 | |
| pos_patterns.pattern_3 | 837 | 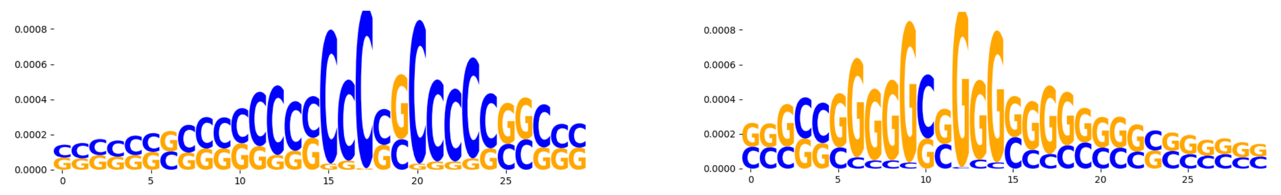 | |
| pos_patterns.pattern_4 | 337 | 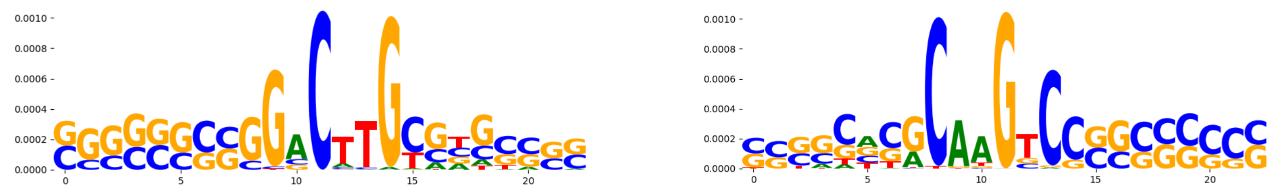 | |
| pos_patterns.pattern_5 | 336 | 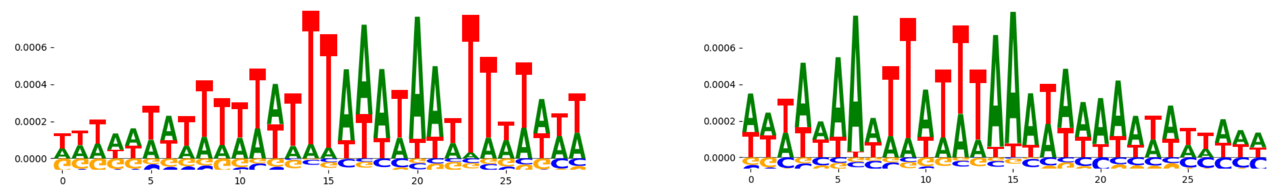 | |
| pos_patterns.pattern_6 | 292 | 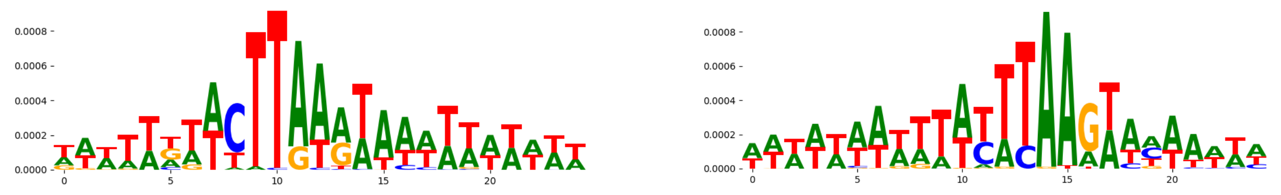 | |
| pos_patterns.pattern_7 | 255 | 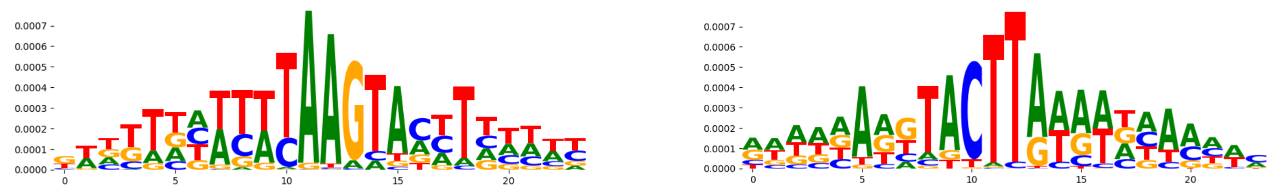 | |
| pos_patterns.pattern_8 | 218 | 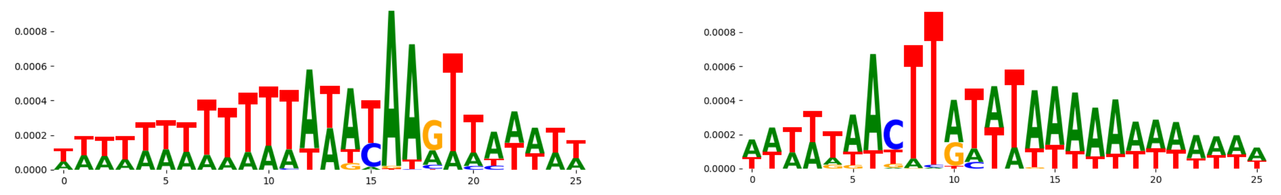 | |
| pos_patterns.pattern_9 | 214 | 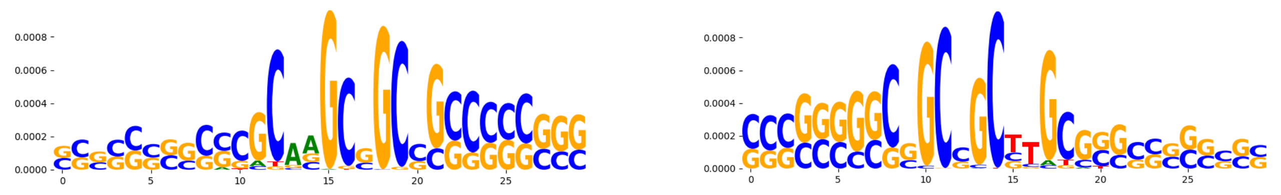 | |
| pos_patterns.pattern_10 | 189 | 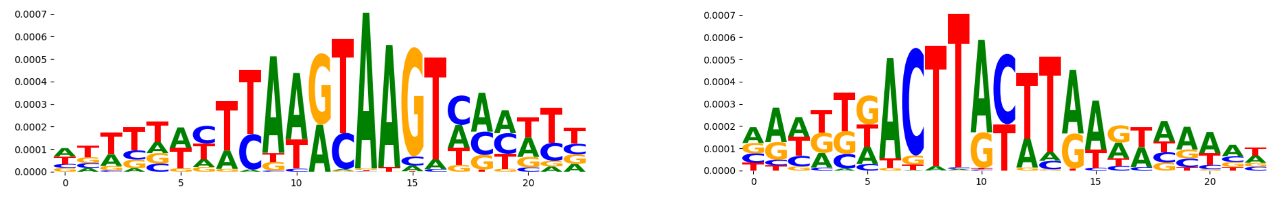 | |
| pos_patterns.pattern_11 | 172 | 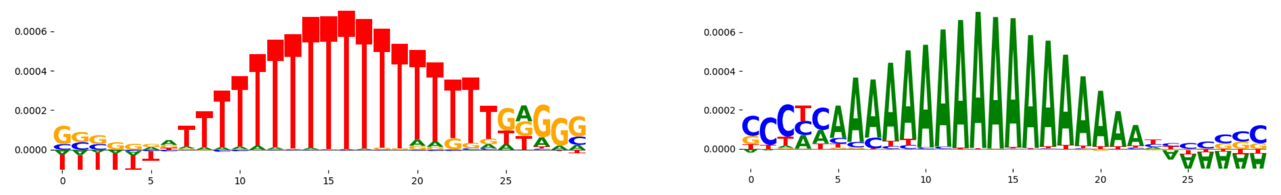 | |
| pos_patterns.pattern_12 | 118 | 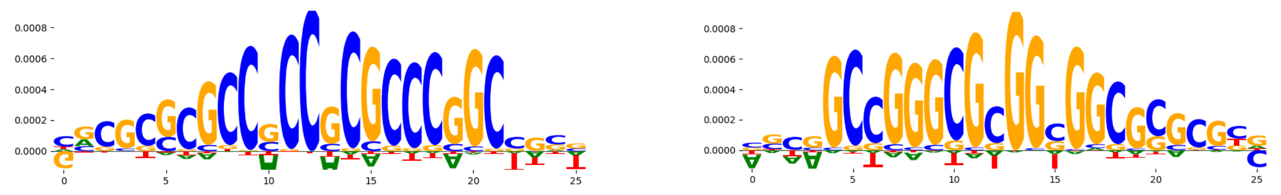 | |
| pos_patterns.pattern_13 | 109 | 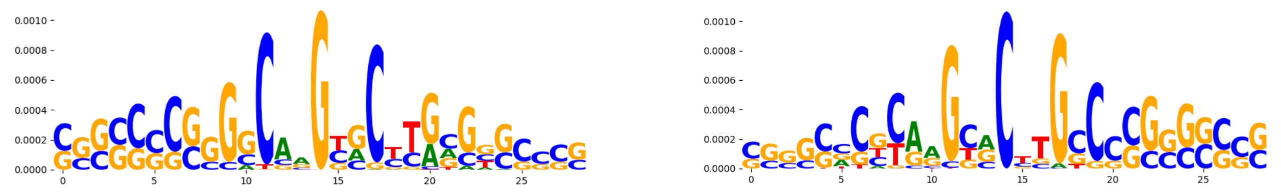 | |
| pos_patterns.pattern_14 | 100 | 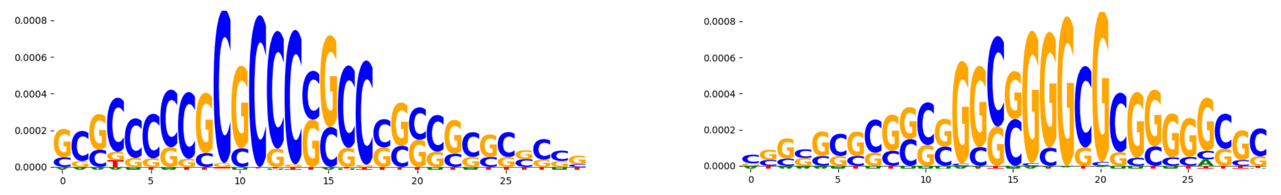 | |
| pos_patterns.pattern_15 | 62 | 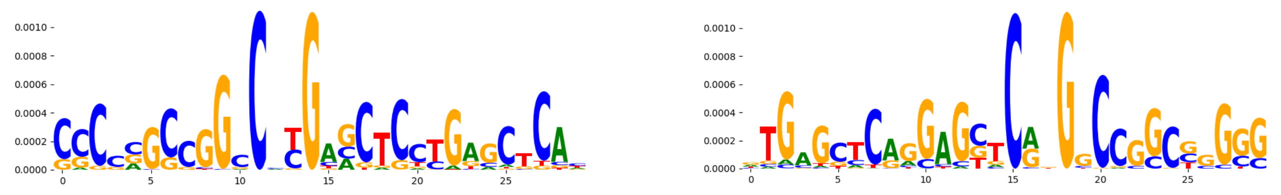 | |
| pos_patterns.pattern_16 | 44 | 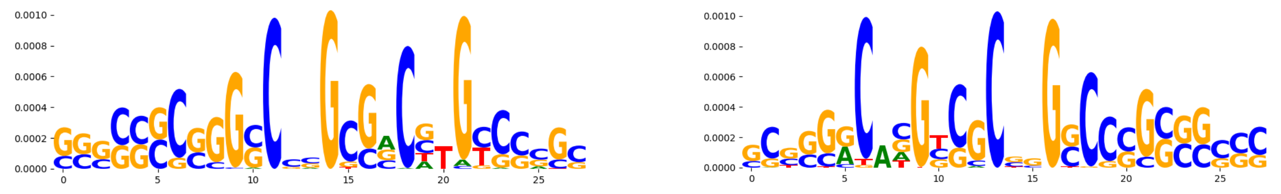 | |
| pos_patterns.pattern_17 | 37 | 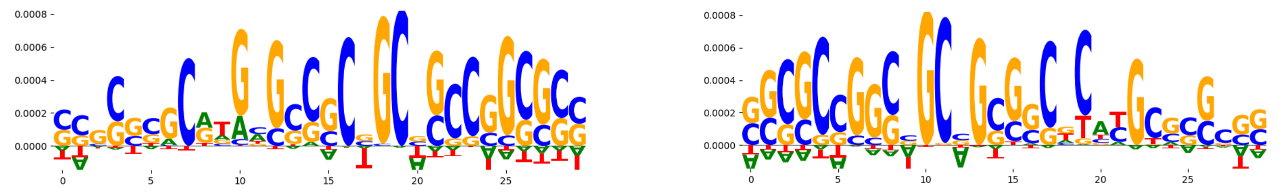 | |
| pos_patterns.pattern_18 | 36 | 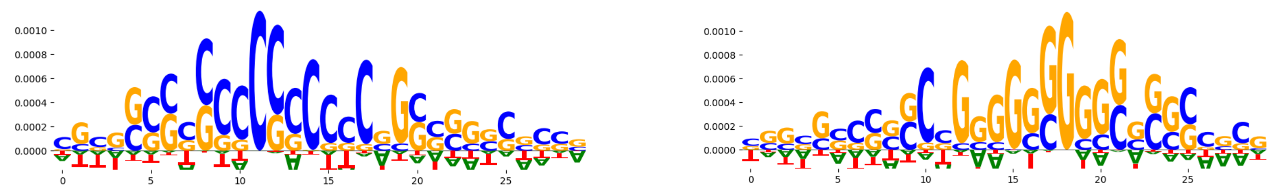 | |
| pos_patterns.pattern_19 | 35 | 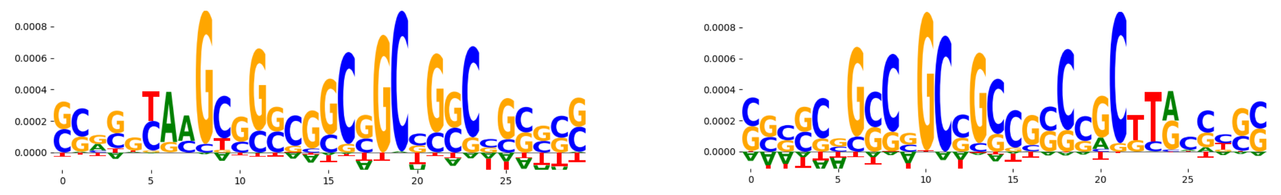 | |
| pos_patterns.pattern_20 | 34 | 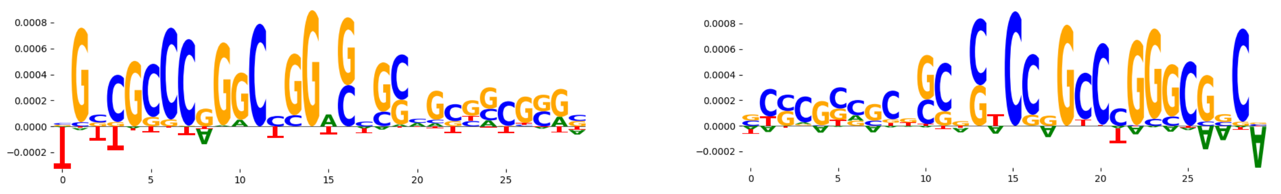 | |
| pos_patterns.pattern_21 | 21 | 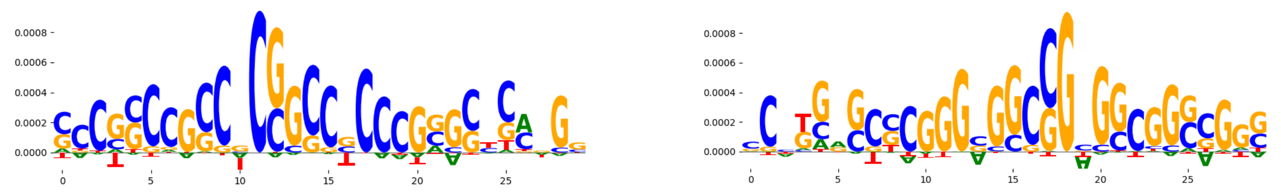 | |
| neg_patterns.pattern_0 | 1635 | 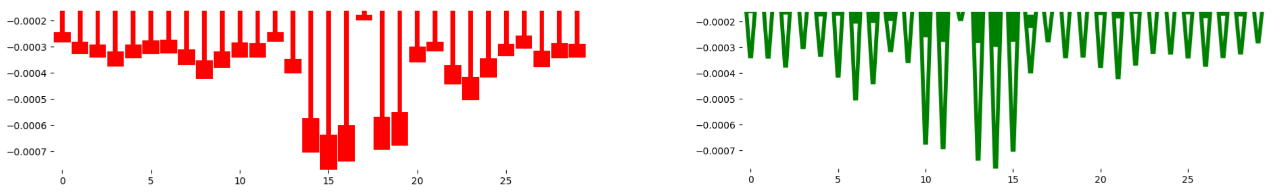 | |

| pattern | num_seqlets | cwm_fwd / cwm_rev |
| --- | --- | --- |
| neg_patterns.pattern_1 | 1616 | 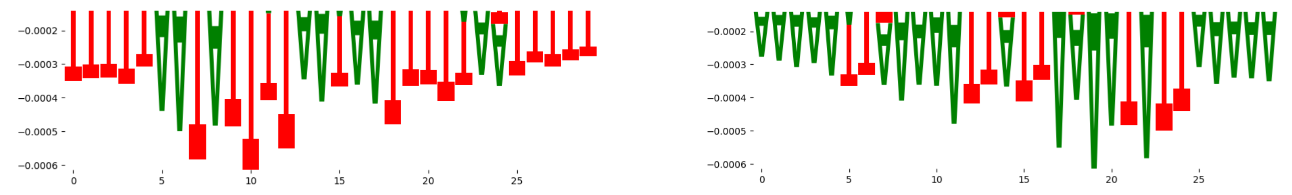 |
| neg_patterns.pattern_2 | 986 | 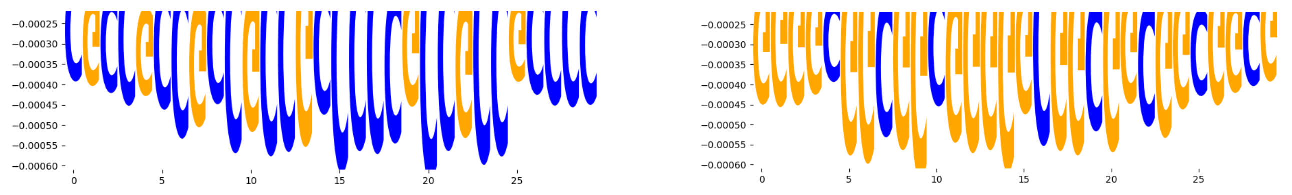 |
| neg_patterns.pattern_3 | 393 | 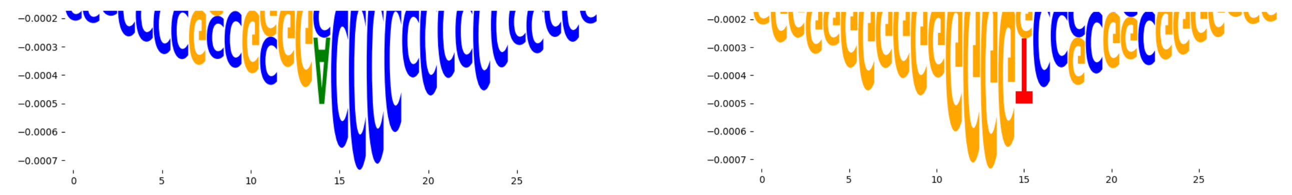 |
| neg_patterns.pattern_4 | 287 | 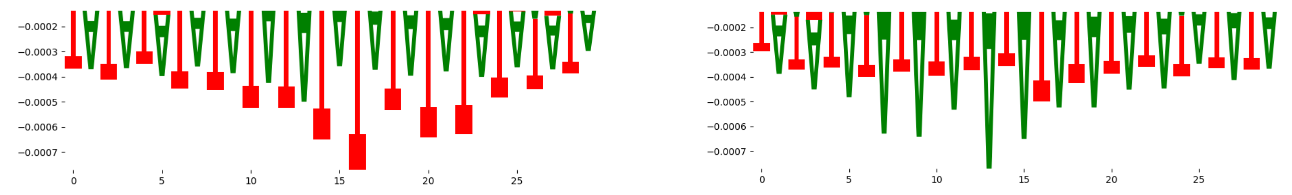 |
| neg_patterns.pattern_5 | 279 | 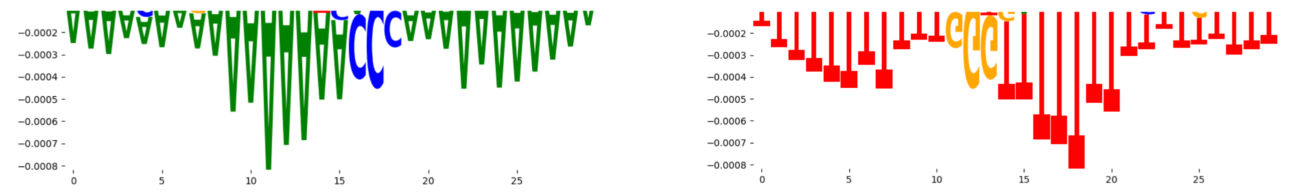 |
| neg_patterns.pattern_6 | 279 | 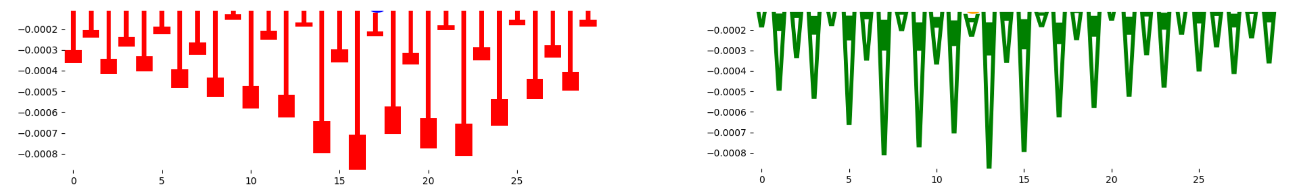 |
| neg_patterns.pattern_7 | 257 | 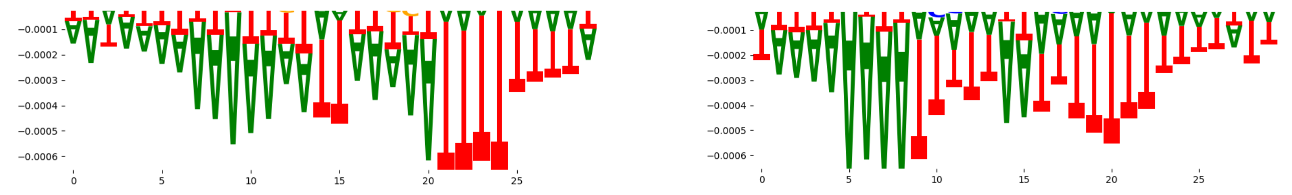 |
| neg_patterns.pattern_8 | 233 | 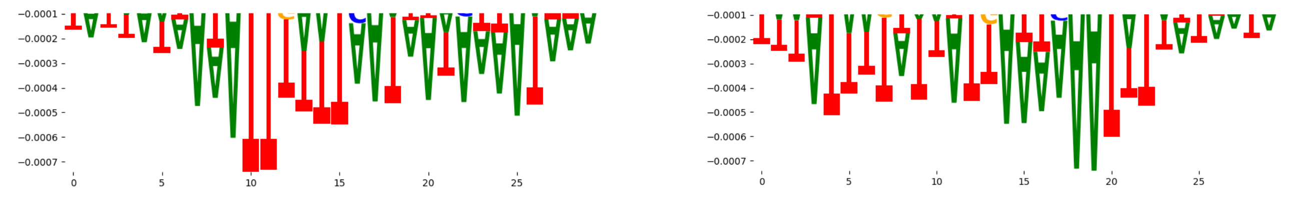 |
| neg_patterns.pattern_9 | 229 | 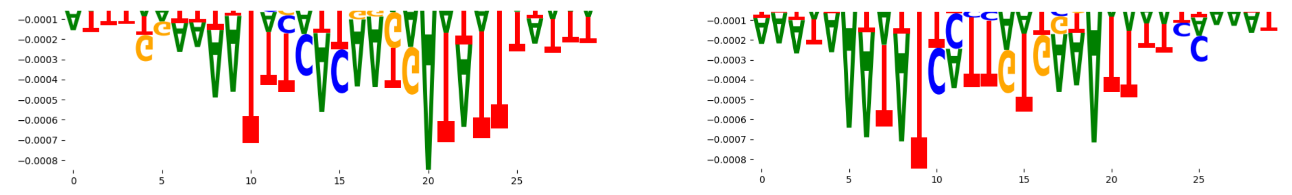 |
| neg_patterns.pattern_10 | 227 | 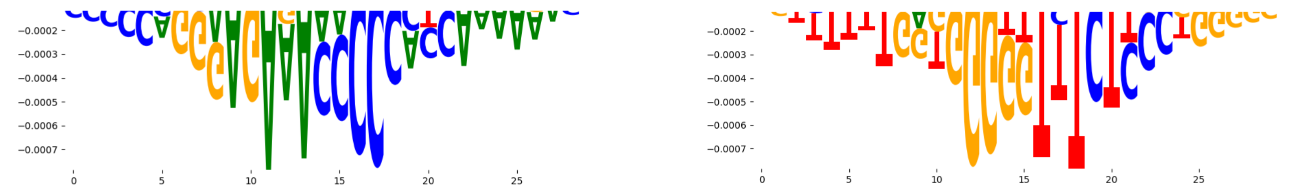 |
| neg_patterns.pattern_11 | 214 | 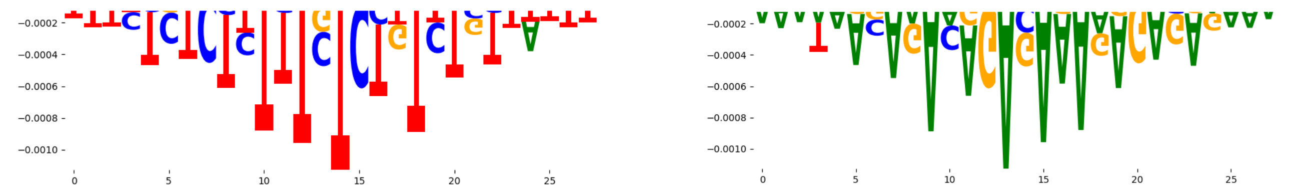 |
| neg_patterns.pattern_12 | 177 | 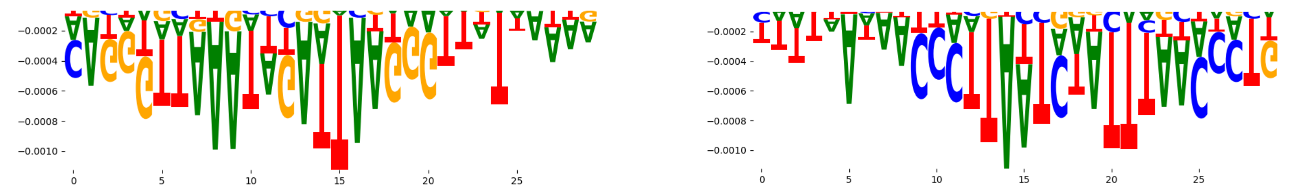 |
| neg_patterns.pattern_13 | 168 | 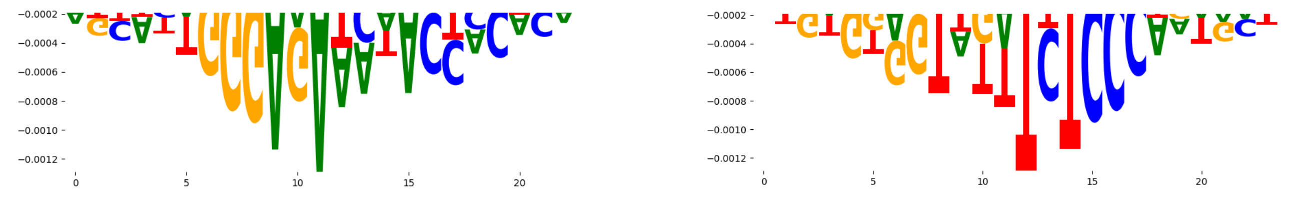 |
| neg_patterns.pattern_14 | 164 | 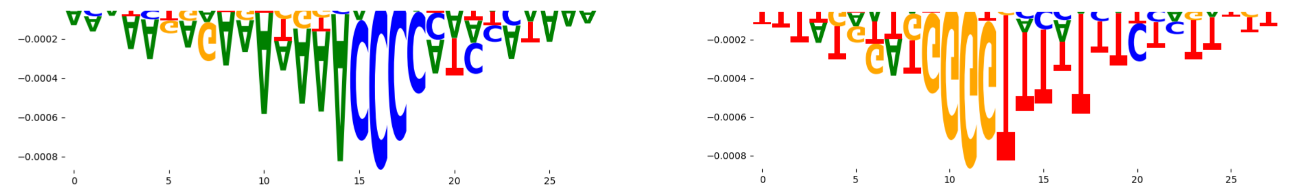 |
| neg_patterns.pattern_15 | 156 | 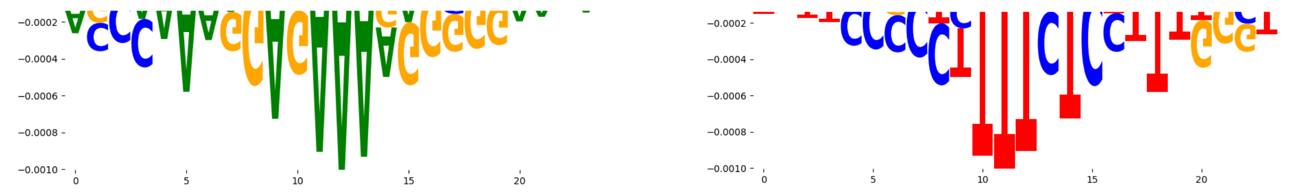 |
| neg_patterns.pattern_16 | 129 | 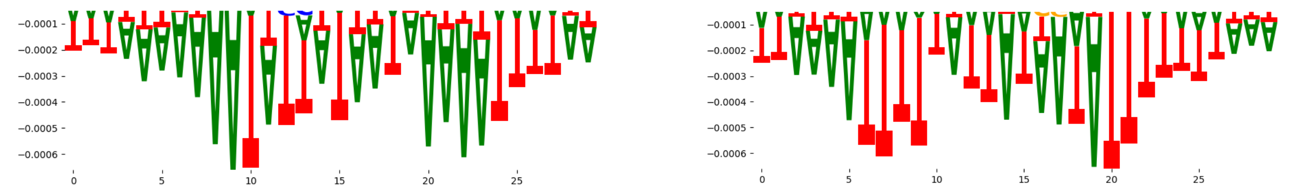 |
| neg_patterns.pattern_17 | 122 | 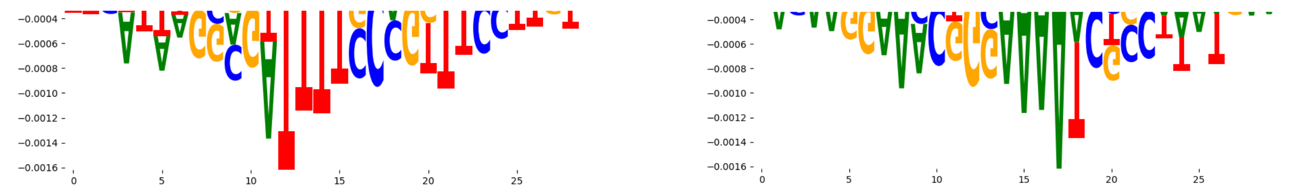 |
| neg_patterns.pattern_18 | 117 | 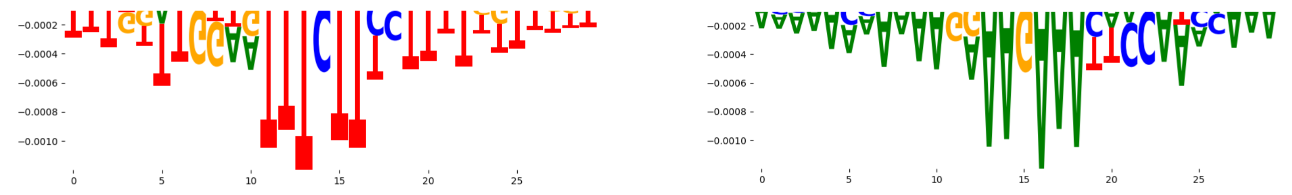 |
| neg_patterns.pattern_19 | 117 | 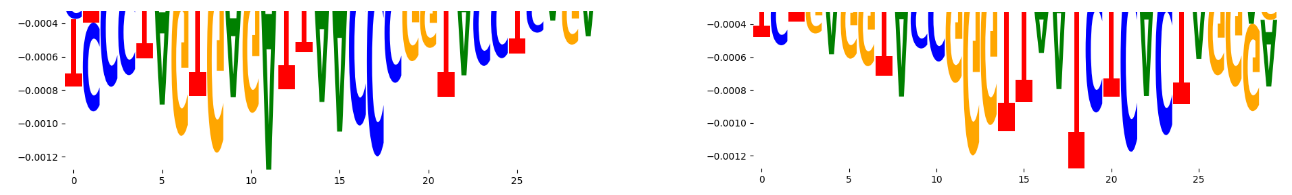 |
| neg_patterns.pattern_20 | 113 | 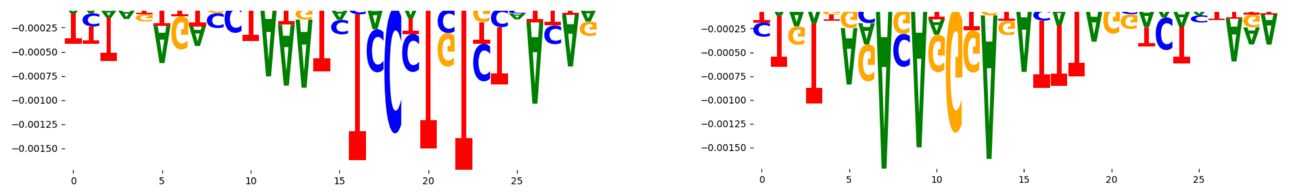 |
| neg_patterns.pattern_21 | 113 | 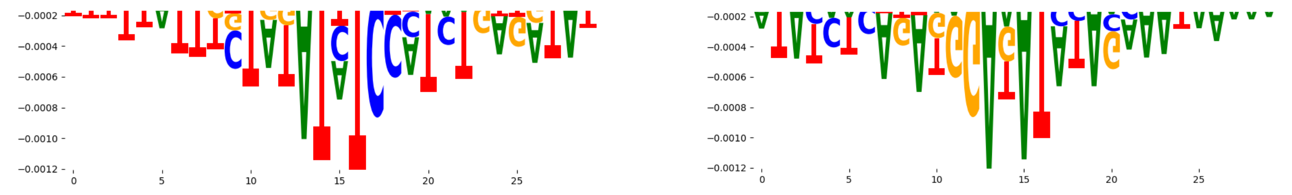 |
| neg_patterns.pattern_22 | 95 | 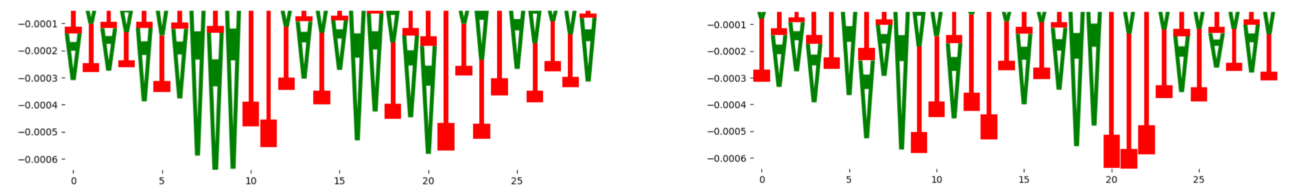 |
| neg_patterns.pattern_23 | 93 | 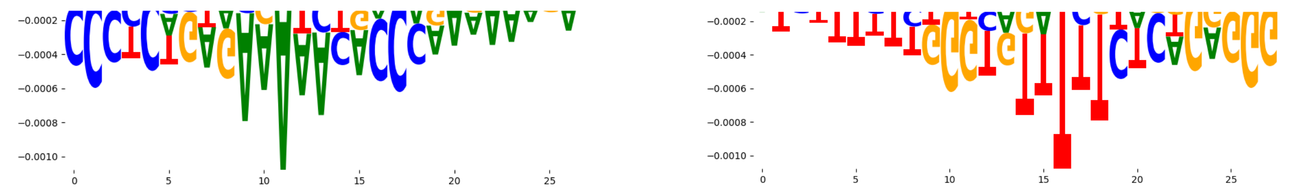 |

| pattern | num_seqlets | cwm_fwd | cwm_rev |
|---|---|---|---|
| neg_patterns.pattern_24 | 81 | 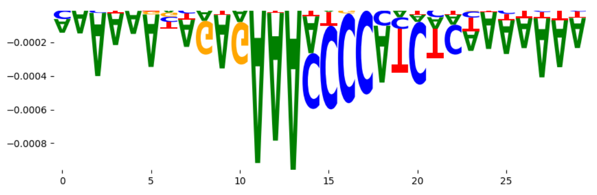 | 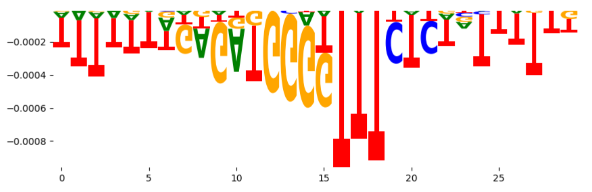 |
| neg_patterns.pattern_25 | 53 | 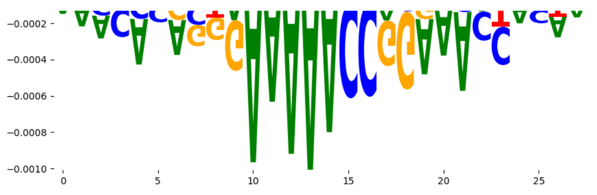 | 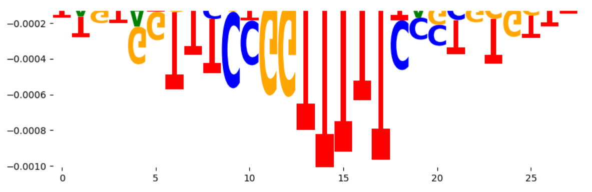 |
| neg_patterns.pattern_26 | 50 | 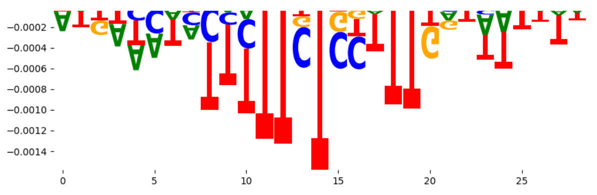 | 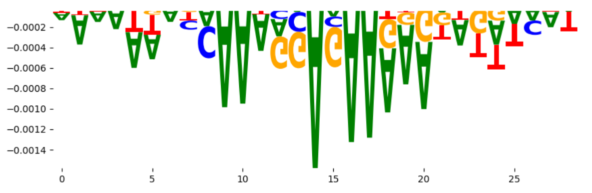 |
| neg_patterns.pattern_27 | 45 | 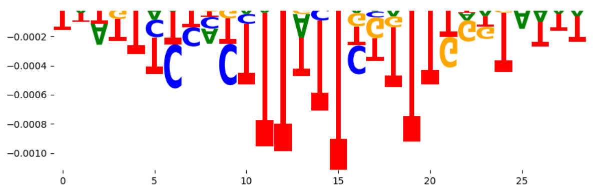 | 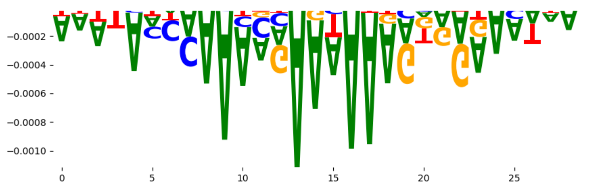 |
| neg_patterns.pattern_28 | 24 | 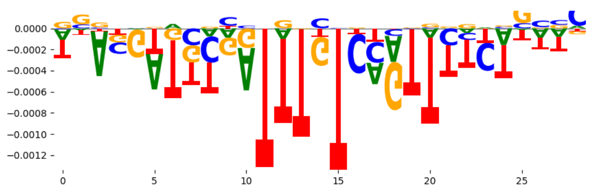 | 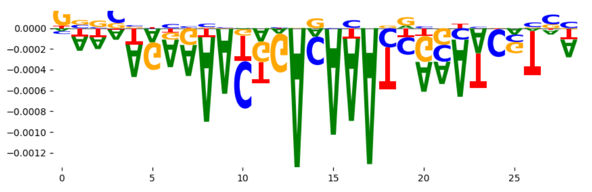 |
| neg_patterns.pattern_29 | 23 | 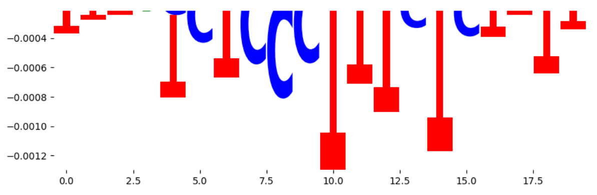 | 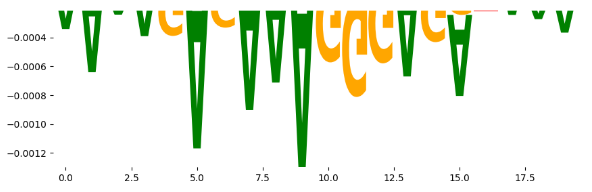 |
| neg_patterns.pattern_30 | 21 | 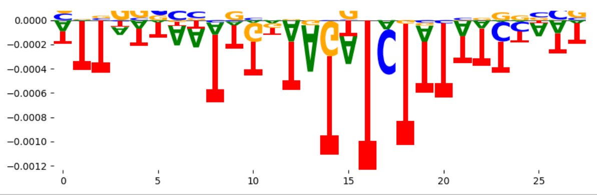 | 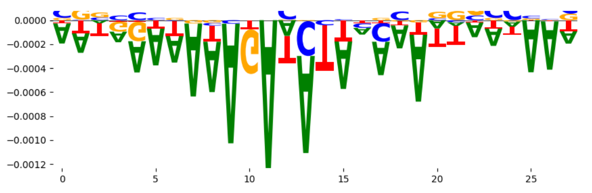 |
