# Supplementary material for: ChromBPNet: bias factorized, base-resolution deep learning models of chromatin accessibility reveal cis-regulatory sequence syntax, transcription factor footprints and regulatory variants
Source: bioRxiv. 2025 Jan 8:2024.12.25.630221. Preprint. [Version 2] doi: 10.1101/2024.12.25.630221 (PMC11741299; doi:10.1101/2024.12.25.630221)
Supplement: Supplement 4 [file media-4.zip › supplementary_files_3/k562_DNASE_bpnet_bias_model/k562_DNASE_raw_bpnet_bias_fold1_counts_modisco.pdf]

pattern	num_seqlets	cwm_fwd	cwm_rev	TOMTOM_match	TOMTOM_qval	TOMTOM_match_logo
pos_patterns.pattern_0	2926			SP1_MOUSE.H11MO.0.A	4.141100e-10	
pos_patterns.pattern_1	2716			SP2_HUMAN.H11MO.0.A	6.082560e-07	
pos_patterns.pattern_2	1847			PRDM6_HUMAN.H11MO.0.C	8.515960e-03	
pos_patterns.pattern_3	1091			ZNF740_C2H2_1	1.877430e-02	
pos_patterns.pattern_4	933			RREB1_MA0073.1	5.720710e-01	
pos_patterns.pattern_5	933			DNASE_2	1.457250e-01	
pos_patterns.pattern_6	740			SOX17_HUMAN.H11MO.0.C	1.000000e+00	
pos_patterns.pattern_7	595			SP2_HUMAN.H11MO.0.A	1.906920e-05	
pos_patterns.pattern_8	384			RUNX3_RUNX_3	1.000000e+00	
pos_patterns.pattern_9	275			RELB_MA1117.1	1.000000e+00	
pos_patterns.pattern_10	255			NFAC4_HUMAN.H11MO.0.C	6.388840e-01	
pos_patterns.pattern_11	207			ZN431_MOUSE.H11MO.0.C	8.170400e-01	
pos_patterns.pattern_12	188			LYL1_HUMAN.H11MO.0.A	5.938620e-01	
pos_patterns.pattern_13	180			ZN257_HUMAN.H11MO.0.C	1.000000e+00	
pos_patterns.pattern_14	72			ISX_homeodomain_1	1.460030e-01	
pos_patterns.pattern_15	49			TN5_6	5.986190e-04	
pos_patterns.pattern_16	42			EWSR1-FLI1_MA0149.1	5.623700e-11	
pos_patterns.pattern_17	40			VDR_MA0693.2	1.000000e+00	
pos_patterns.pattern_18	24			SMAD2+SMAD3+SMAD4_MA0513.1	7.998340e-02	
pos_patterns.pattern_19	24			ZN770_HUMAN.H11MO.0.C	7.065100e-07	
pos_patterns.pattern_20	21			TN5_6	2.675810e-06	
neg_patterns.pattern_0	2688			DNASE_2	1.934110e-03	
neg_patterns.pattern_1	1712			DNASE_2	5.153310e-01	
neg_patterns.pattern_2	807			EGR1_MOUSE.H11MO.0.A	5.715480e-03	
neg_patterns.pattern_3	463			BARHL2_homeodomain_2	1.000000e+00	
neg_patterns.pattern_4	410			PRDM6_HUMAN.H11MO.0.C	2.721510e-02	
neg_patterns.pattern_5	373			DNASE_1	1.658490e-01	
neg_patterns.pattern_6	357			E2F7_HUMAN.H11MO.0.B	1.000000e+00	
neg_patterns.pattern_7	241			PPARG_MA0066.1	8.912570e-01	
neg_patterns.pattern_8	227			LYL1_HUMAN.H11MO.0.A	3.529440e-01	

pattern	num_seqlets	cwm_fwd	cwm_rev	TOMTOM_match	TOMTOM_qval	TOMTOM_match_logo
neg_patterns.pattern_9	174	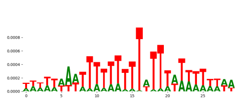	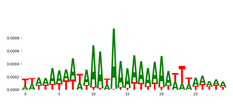	ELF5_MOUSE.H11MO.0.A	7.360410e-04	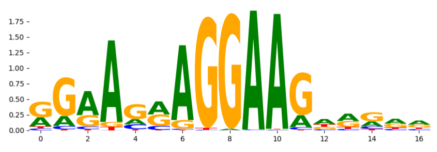
neg_patterns.pattern_10	126	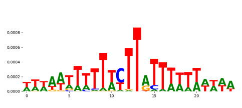	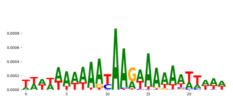	PRDM6_HUMAN.H11MO.0.C	9.146250e-02	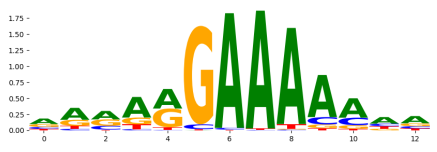
neg_patterns.pattern_11	104	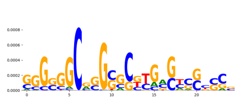	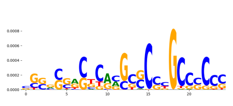	ZN257_HUMAN.H11MO.0.C	1.000000e+00	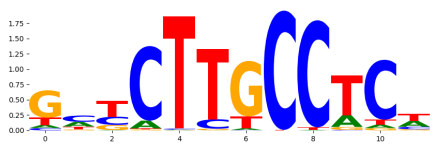
neg_patterns.pattern_12	93	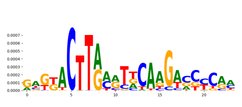	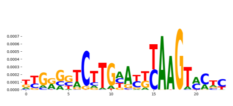	NKX28_HUMAN.H11MO.0.C	1.000000e+00	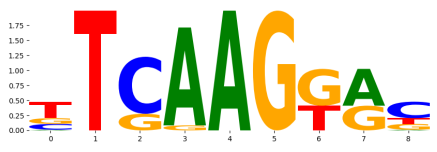
neg_patterns.pattern_13	80	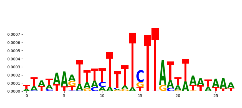	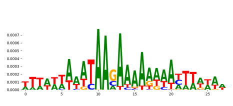	GATA3_GATA_1	5.050270e-02	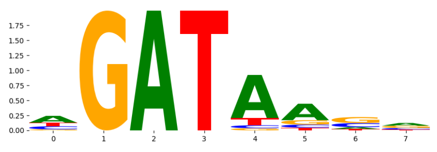
neg_patterns.pattern_14	72	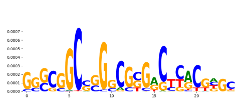	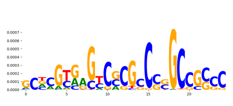	P73_HUMAN.H11MO.0.A	2.057810e-01	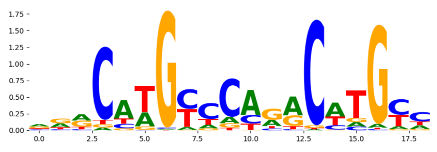
neg_patterns.pattern_15	50	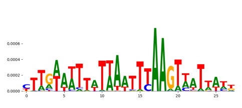	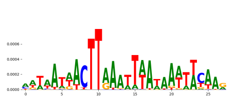	NKX2-3_MA0672.1	1.918850e-01	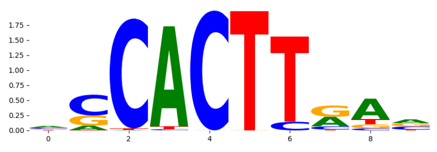
neg_patterns.pattern_16	22	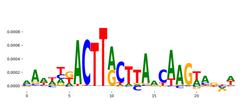	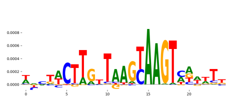	DNASE_6	1.708030e-01	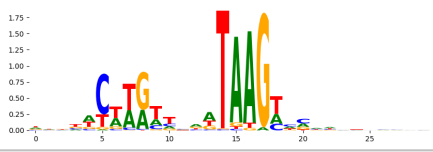
