# Supplementary material for: ChromBPNet: bias factorized, base-resolution deep learning models of chromatin accessibility reveal cis-regulatory sequence syntax, transcription factor footprints and regulatory variants
Source: bioRxiv. 2025 Jan 8:2024.12.25.630221. Preprint. [Version 2] doi: 10.1101/2024.12.25.630221 (PMC11741299; doi:10.1101/2024.12.25.630221)
Supplement: Supplement 4 [file media-4.zip › supplementary_files_3/k562_DNASE_bpnet_bias_model/k562_DNASE_raw_bpnet_bias_fold2_profile_modisco.pdf]

pattern	num_seqlets	cwm_fwd	cwm_rev
pos_patterns.pattern_0	5585	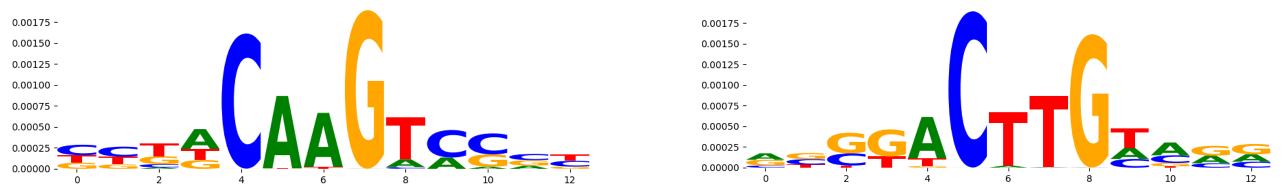	
pos_patterns.pattern_1	3642	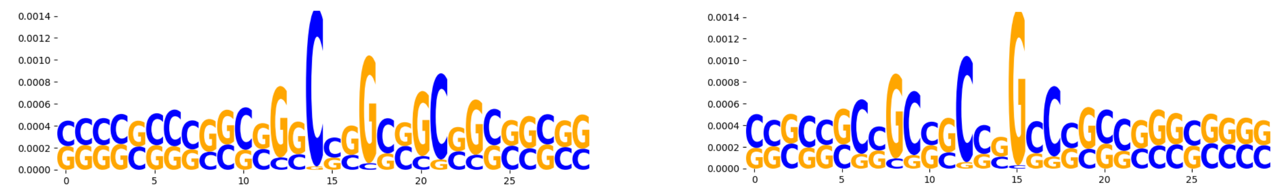	
pos_patterns.pattern_2	2531	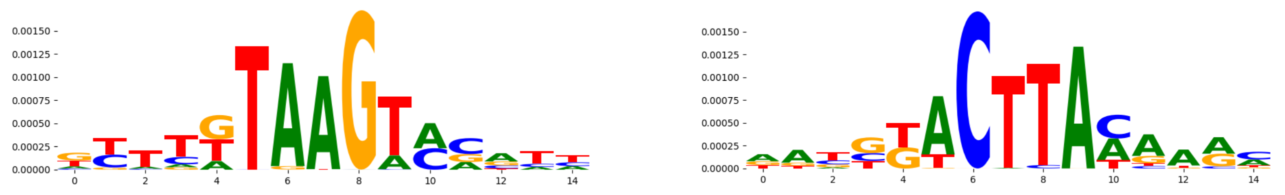	
pos_patterns.pattern_3	1830	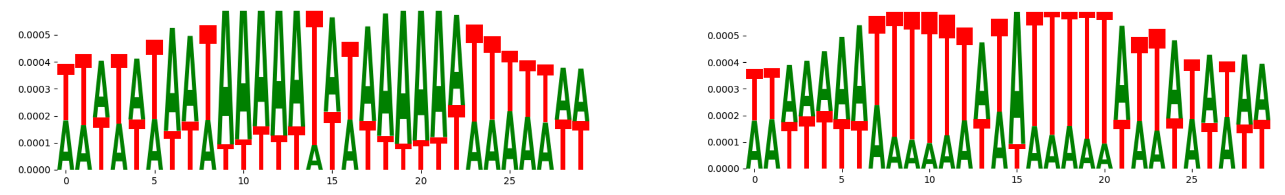	
pos_patterns.pattern_4	959	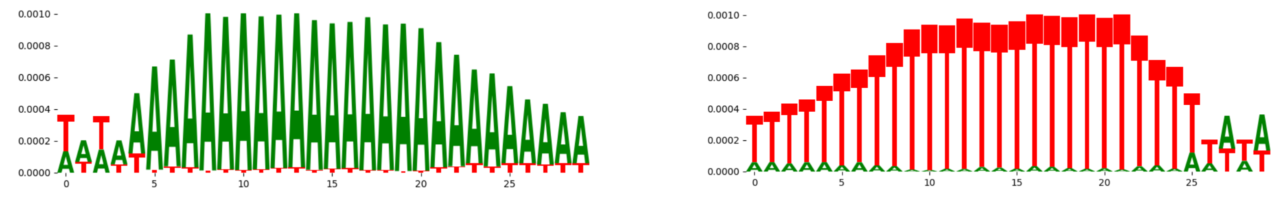	
pos_patterns.pattern_5	934	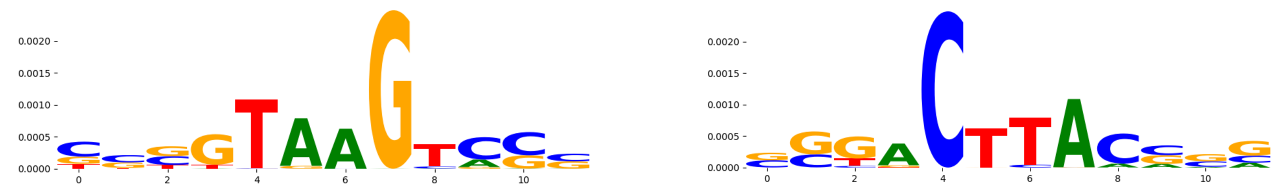	
pos_patterns.pattern_6	875	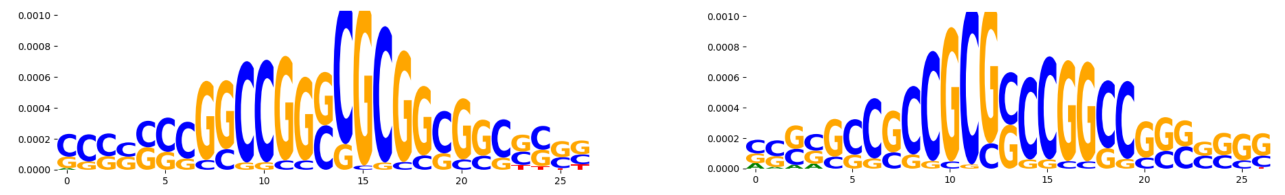	
pos_patterns.pattern_7	787	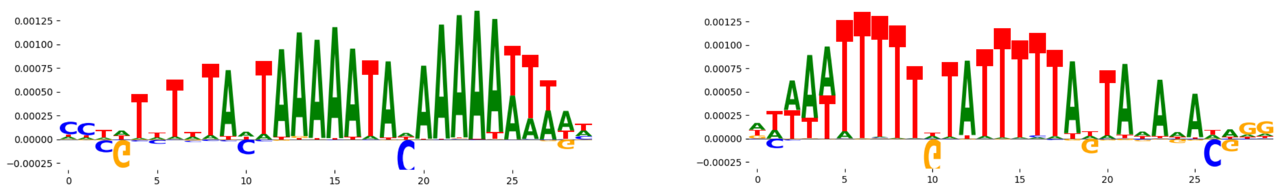	
pos_patterns.pattern_8	422	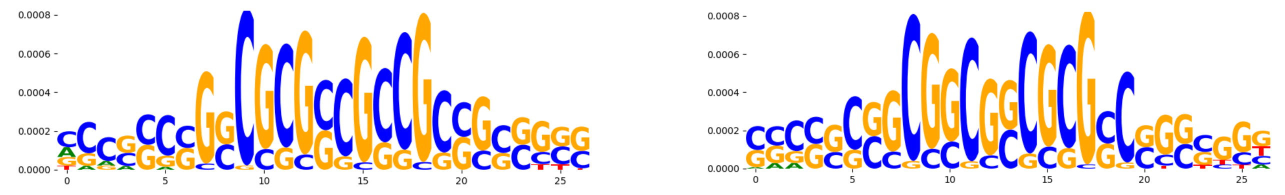	
pos_patterns.pattern_9	317	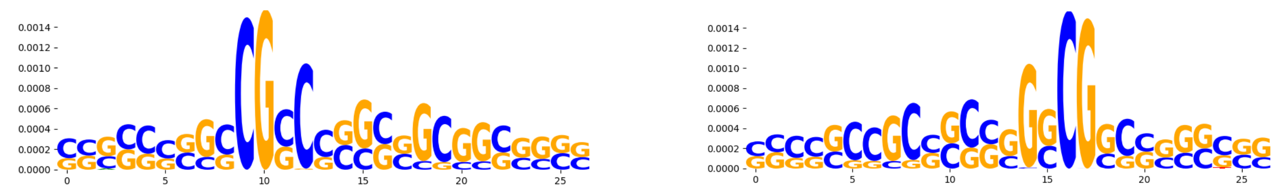	
pos_patterns.pattern_10	316	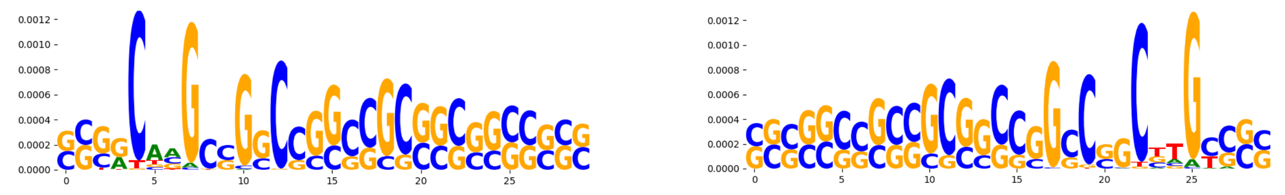	
pos_patterns.pattern_11	240	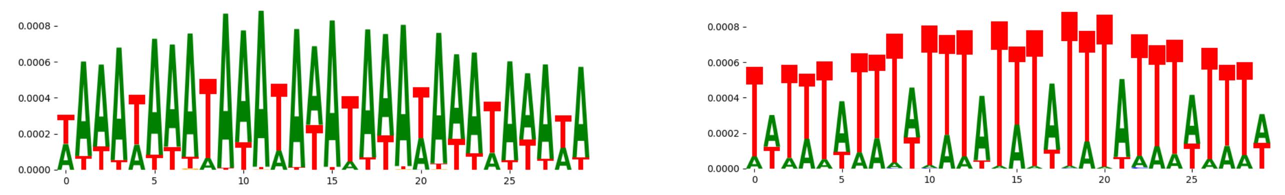	
pos_patterns.pattern_12	211	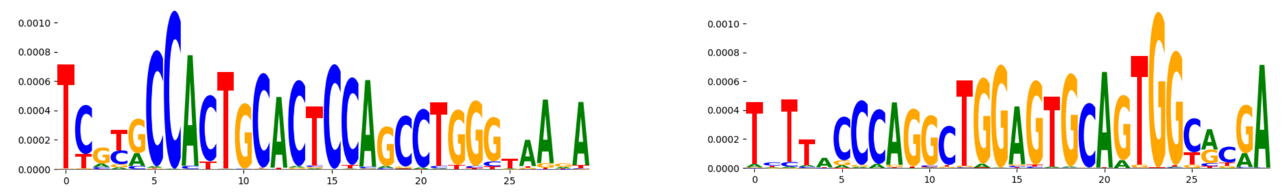	
pos_patterns.pattern_13	192	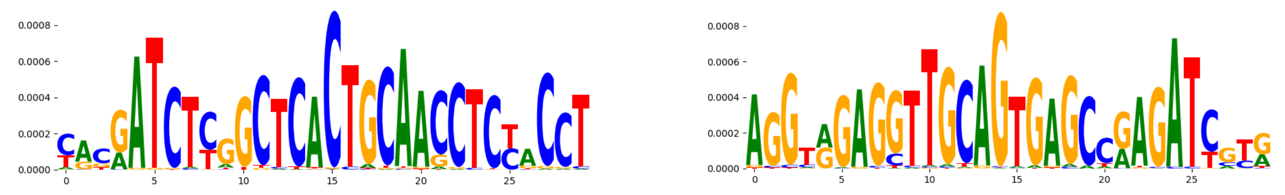	
pos_patterns.pattern_14	183	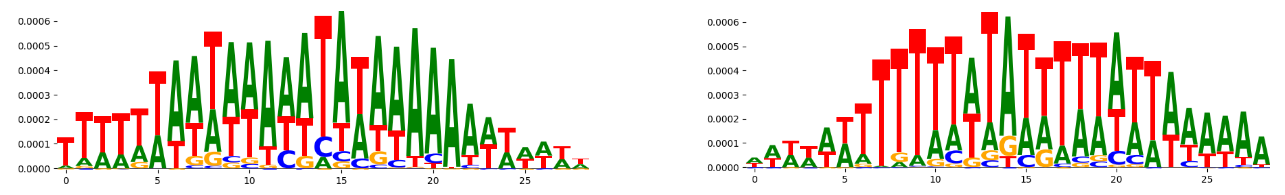	
pos_patterns.pattern_15	177	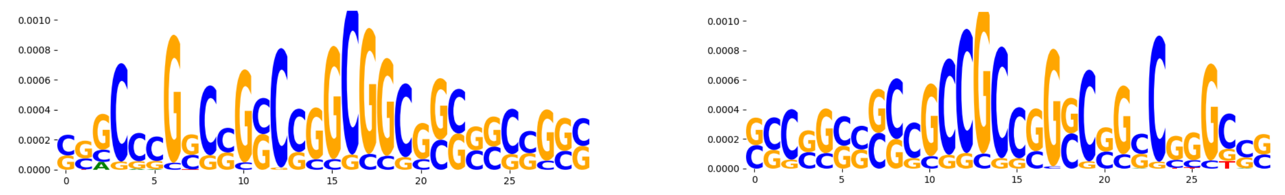	
pos_patterns.pattern_16	166	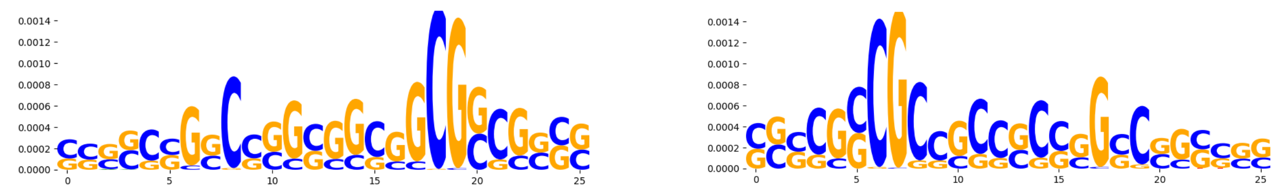	
pos_patterns.pattern_17	165	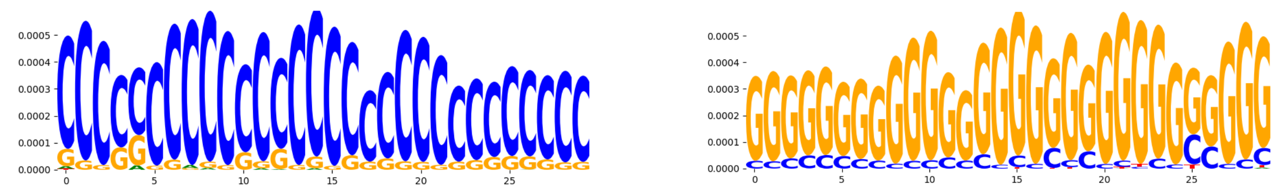	
pos_patterns.pattern_18	135	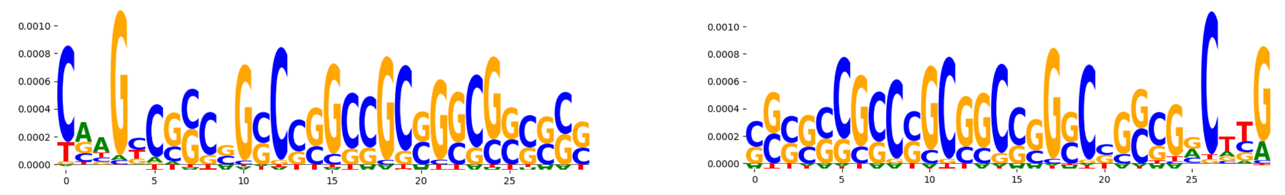	
pos_patterns.pattern_19	117	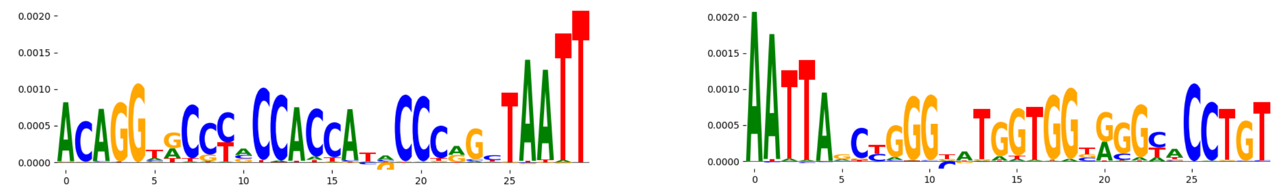	
pos_patterns.pattern_20	100	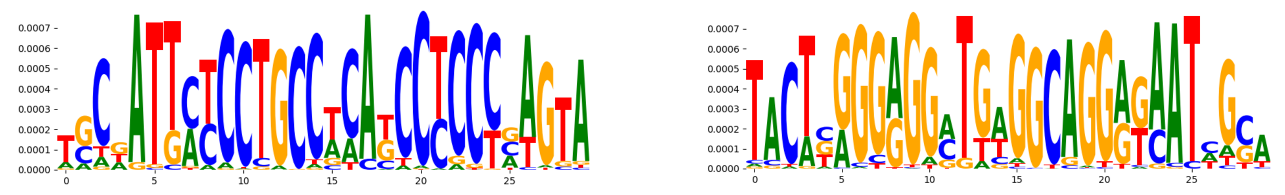	
pos_patterns.pattern_21	81	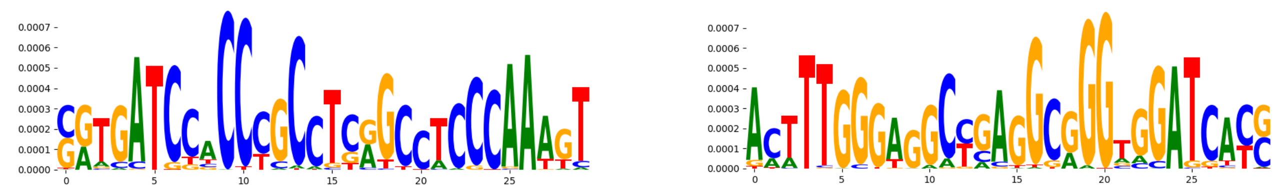	
pos_patterns.pattern_22	70	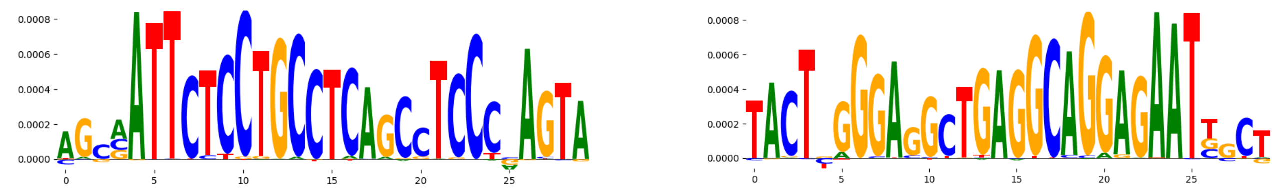	

pattern	num_seqlets	cwm_fwd	cwm_rev
pos_patterns.pattern_23	64	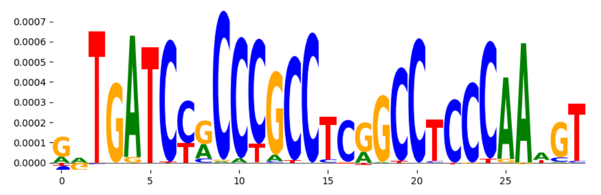	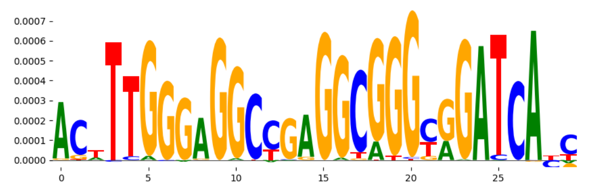
pos_patterns.pattern_24	40	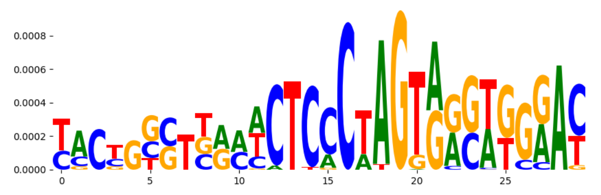	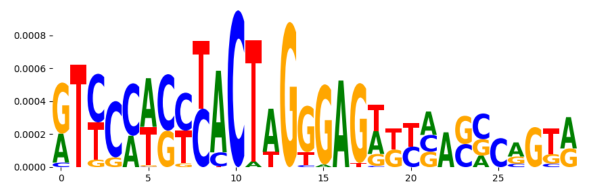
pos_patterns.pattern_25	38	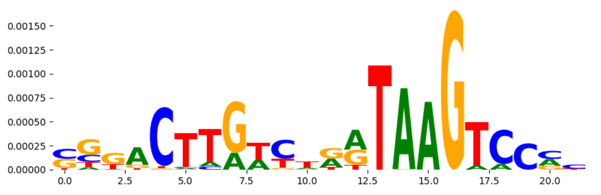	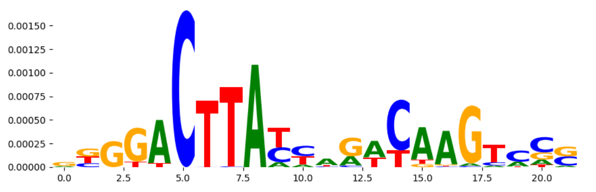
pos_patterns.pattern_26	34	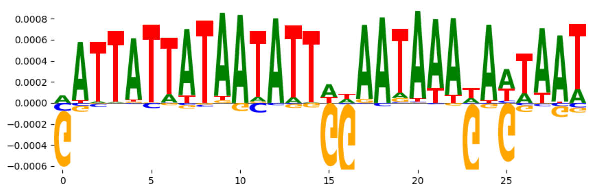	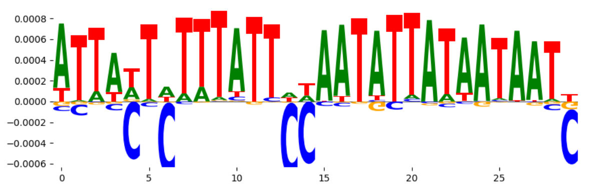
pos_patterns.pattern_27	32	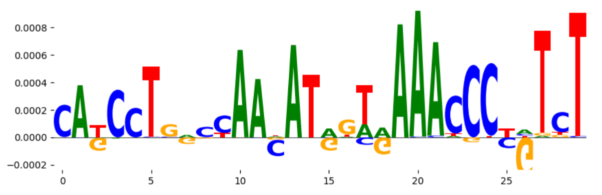	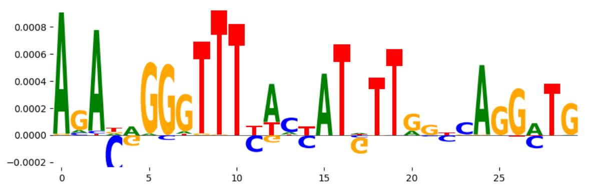
pos_patterns.pattern_28	27	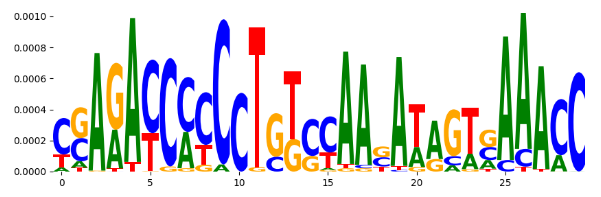	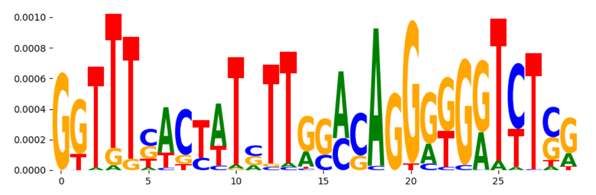
neg_patterns.pattern_0	25	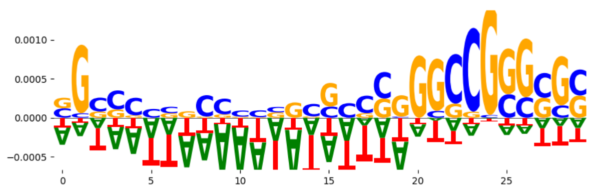	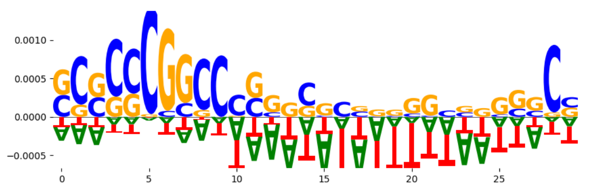
neg_patterns.pattern_1	22	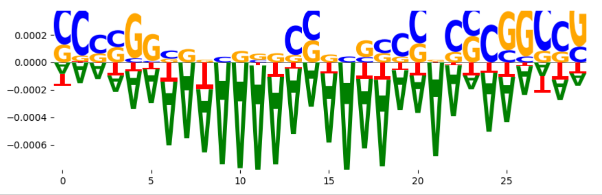	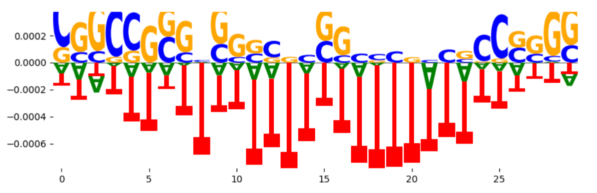
