# Supplementary material for: ChromBPNet: bias factorized, base-resolution deep learning models of chromatin accessibility reveal cis-regulatory sequence syntax, transcription factor footprints and regulatory variants
Source: bioRxiv. 2025 Jan 8:2024.12.25.630221. Preprint. [Version 2] doi: 10.1101/2024.12.25.630221 (PMC11741299; doi:10.1101/2024.12.25.630221)
Supplement: Supplement 4 [file media-4.zip › supplementary_files_3/k562_DNASE_bpnet_bias_model/k562_DNASE_raw_bpnet_bias_fold3_profile_modisco.pdf]

pattern	num_seqlets	cwm_fwd	cwm_rev	TOMTOM_match	TOMTOM_qval	TOMTOM_match_logo
pos_patterns.pattern_0	5468			DNASE_1	1.596660e-01	
pos_patterns.pattern_1	3011			DNASE_2	5.999550e-01	
pos_patterns.pattern_2	3000			SMAD3_HUMAN.H11MO.0.B	3.642250e-01	
pos_patterns.pattern_3	1849			ZNF384_MA1125.1	1.463050e-01	
pos_patterns.pattern_4	1083			CTCFL_HUMAN.H11MO.0.A	2.250680e-01	
pos_patterns.pattern_5	1035			PRDM6_HUMAN.H11MO.0.C	8.316880e-02	
pos_patterns.pattern_6	989			CTCFL_MOUSE.H11MO.0.A	6.020230e-05	
pos_patterns.pattern_7	917			TYY1_HUMAN.H11MO.0.A	1.500790e-01	
pos_patterns.pattern_8	744			MEF2B_HUMAN.H11MO.0.A	2.134900e-01	
pos_patterns.pattern_9	393			CTCFL_HUMAN.H11MO.0.A	2.425830e-01	
pos_patterns.pattern_10	275			SP2_HUMAN.H11MO.0.A	1.462600e-09	
pos_patterns.pattern_11	220			ZN257_HUMAN.H11MO.0.C	1.000000e+00	
pos_patterns.pattern_12	192			RARG_HUMAN.H11MO.0.B	1.000000e+00	
pos_patterns.pattern_13	175			DNASE_5	2.742280e-03	
pos_patterns.pattern_14	164			AP2B_HUMAN.H11MO.0.B	1.555480e-01	
pos_patterns.pattern_15	138			ITF2_HUMAN.H11MO.0.C	1.000000e+00	
pos_patterns.pattern_16	138			CPEB1_RRM_1	3.223090e-01	
pos_patterns.pattern_17	134			CTCFL_HUMAN.H11MO.0.A	6.909920e-01	
pos_patterns.pattern_18	70			RARA_HUMAN.H11MO.0.A	9.558640e-02	
pos_patterns.pattern_19	67			ZN250_HUMAN.H11MO.0.C	1.000000e+00	
pos_patterns.pattern_20	62			ZN770_HUMAN.H11MO.0.C	1.242120e-04	
pos_patterns.pattern_21	48			FOXB1_forkhead_2	1.000000e+00	
pos_patterns.pattern_22	31			DNASE_6	7.491160e-02	
neg_patterns.pattern_0	32			CPEB1_RRM_1	3.004510e-01	
