# Supplementary material for: ChromBPNet: bias factorized, base-resolution deep learning models of chromatin accessibility reveal cis-regulatory sequence syntax, transcription factor footprints and regulatory variants
Source: bioRxiv. 2025 Jan 8:2024.12.25.630221. Preprint. [Version 2] doi: 10.1101/2024.12.25.630221 (PMC11741299; doi:10.1101/2024.12.25.630221)
Supplement: Supplement 4 [file media-4.zip › supplementary_files_3/k562_DNASE_bpnet_bias_model/k562_DNASE_raw_bpnet_bias_fold0_profile_modisco.pdf]

pattern	num_seqlets	cwm_fwd	cwm_rev	TOMTOM_match	TOMTOM_qval	TOMTOM_match_logo
pos_patterns.pattern_0	5693			DNASE_1	2.635070e-01	
pos_patterns.pattern_1	3621			CREM_HUMAN.H11MO.0.C	1.000000e+00	
pos_patterns.pattern_2	2711			DNASE_2	3.161020e-04	
pos_patterns.pattern_3	1749			ZNF384_MA1125.1	1.901360e-01	
pos_patterns.pattern_4	921			PRDM6_HUMAN.H11MO.0.C	7.562760e-02	
pos_patterns.pattern_5	921			NaN	NaN	
pos_patterns.pattern_6	859			MEF2B_HUMAN.H11MO.0.A	2.146410e-01	
pos_patterns.pattern_7	630			KLF3_HUMAN.H11MO.0.B	3.518420e-01	
pos_patterns.pattern_8	516			EGR1_MOUSE.H11MO.0.A	1.014840e-01	
pos_patterns.pattern_9	497			ZFX_MOUSE.H11MO.0.B	2.755700e-01	
pos_patterns.pattern_10	455			MXI1_HUMAN.H11MO.0.A	7.342290e-01	
pos_patterns.pattern_11	270			SP1_HUMAN.H11MO.0.A	6.910940e-01	
pos_patterns.pattern_12	268			THAP1_HUMAN.H11MO.0.C	3.162780e-01	
pos_patterns.pattern_13	224			TYY1_HUMAN.H11MO.0.A	1.322730e-02	
pos_patterns.pattern_14	209			TN5_6	9.054130e-21	
pos_patterns.pattern_15	190			ZN770_HUMAN.H11MO.0.C	2.604730e-08	
pos_patterns.pattern_16	161			FOXB1_forkhead_2	1.557050e-01	
pos_patterns.pattern_17	139			ZN770_HUMAN.H11MO.0.C	6.701660e-08	
pos_patterns.pattern_18	133			CTCFL_HUMAN.H11MO.0.A	9.706390e-04	
pos_patterns.pattern_19	122			ZNF384_MA1125.1	1.861490e-01	
pos_patterns.pattern_20	119			TAF1_HUMAN.H11MO.0.A	4.224020e-01	
pos_patterns.pattern_21	90			DNASE_5	5.536750e-01	
pos_patterns.pattern_22	86			STAT1_MOUSE.H11MO.0.A	6.668460e-02	
pos_patterns.pattern_23	86			SP1_MOUSE.H11MO.0.A	3.532660e-08	
pos_patterns.pattern_24	55			ZN121_HUMAN.H11MO.0.C	1.017460e-03	
pos_patterns.pattern_25	41			ZN770_HUMAN.H11MO.0.C	6.074630e-02	
pos_patterns.pattern_26	33			MAF_MOUSE.H11MO.0.A	5.617860e-01	
pos_patterns.pattern_27	32			RARA_HUMAN.H11MO.0.A	1.537110e-01	
pos_patterns.pattern_28	31			DNASE_6	3.557460e-01	
neg_patterns.pattern_0	24			PRDM6_HUMAN.H11MO.0.C	3.875060e-01	
