# Supplementary material for: ChromBPNet: bias factorized, base-resolution deep learning models of chromatin accessibility reveal cis-regulatory sequence syntax, transcription factor footprints and regulatory variants
Source: bioRxiv. 2025 Jan 8:2024.12.25.630221. Preprint. [Version 2] doi: 10.1101/2024.12.25.630221 (PMC11741299; doi:10.1101/2024.12.25.630221)
Supplement: Supplement 4 [file media-4.zip › supplementary_files_3/k562_DNASE_bpnet_bias_model/k562_DNASE_raw_bpnet_bias_fold1_profile_modisco.pdf]

pattern	num_seqlets	cwm_fwd	cwm_rev	TOMTOM_match	TOMTOM_qval	TOMTOM_match_logo
pos_patterns.pattern_0	4184			TFAP2A_AP2_4	2.532460e-01	
pos_patterns.pattern_1	3809			ERR2_HUMAN.H11MO.0.A	1.000000e+00	
pos_patterns.pattern_2	2736			DNASE_2	8.434530e-01	
pos_patterns.pattern_3	1458			FOXJ3_HUMAN.H11MO.0.A	1.777370e-01	
pos_patterns.pattern_4	1351			CTCFL_HUMAN.H11MO.0.A	2.710010e-01	
pos_patterns.pattern_5	1154			ZFX_MOUSE.H11MO.0.B	3.428800e-02	
pos_patterns.pattern_6	856			DNASE_1	1.000000e+00	
pos_patterns.pattern_7	850			PRDM6_HUMAN.H11MO.0.C	8.057040e-02	
pos_patterns.pattern_8	807			CTCFL_MOUSE.H11MO.0.A	7.542130e-04	
pos_patterns.pattern_9	610			MEF2B_HUMAN.H11MO.0.A	2.148970e-01	
pos_patterns.pattern_10	331			NFIB_MOUSE.H11MO.0.C	2.227970e-01	
pos_patterns.pattern_11	247			ZN770_HUMAN.H11MO.0.C	4.280760e-03	
pos_patterns.pattern_12	225			SP1_HUMAN.H11MO.0.A	2.581380e-09	
pos_patterns.pattern_13	193			TN5_6	2.826920e-16	
pos_patterns.pattern_14	171			TN5_6	2.260950e-01	
pos_patterns.pattern_15	159			MYBA_MOUSE.H11MO.0.C	1.253610e-01	
pos_patterns.pattern_16	152			SMAD3_HUMAN.H11MO.0.B	6.498270e-01	
pos_patterns.pattern_17	148			ZN770_HUMAN.H11MO.0.C	9.034850e-12	
pos_patterns.pattern_18	117			ZN250_HUMAN.H11MO.0.C	1.000000e+00	
pos_patterns.pattern_19	80			FOXB1_forkhead_2	2.831350e-01	
pos_patterns.pattern_20	62			DNASE_5	1.207530e-01	
pos_patterns.pattern_21	57			TN5_6	3.335100e-09	
pos_patterns.pattern_22	49			ZN770_HUMAN.H11MO.0.C	6.213010e-04	
pos_patterns.pattern_23	34			ITF2_HUMAN.H11MO.0.C	1.000000e+00	
pos_patterns.pattern_24	30			YY1_MA0095.2	1.565180e-01	
pos_patterns.pattern_25	27			DNASE_5	1.383860e-10	
pos_patterns.pattern_26	25			PO3F1_MOUSE.H11MO.0.C	1.000000e+00	
pos_patterns.pattern_27	25			Arid5a_MA0602.1	1.000000e+00	
pos_patterns.pattern_28	24			RXRA_MOUSE.H11MO.0.A	7.124840e-01	

pattern	num_seqlets	cwm_fwd	cwm_rev	TOMTOM_match	TOMTOM_qval	TOMTOM_match_logo
neg_patterns.pattern_0	30	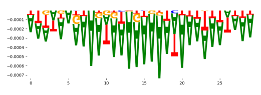	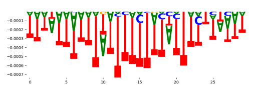	PRDM6_HUMAN.H11MO.0.C	5.031330e-03	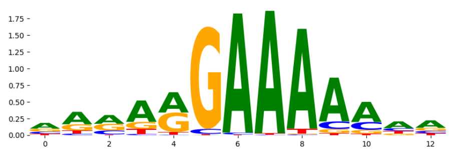
neg_patterns.pattern_1	24	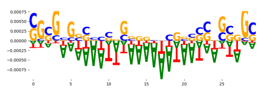	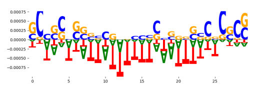	ZNF384_MA1125.1	2.271800e-02	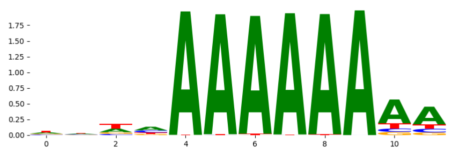
neg_patterns.pattern_2	20	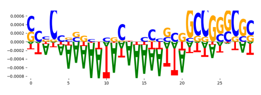	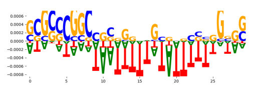	FOXJ3_HUMAN.H11MO.0.A	8.812020e-02	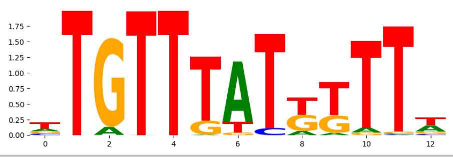
