# Supplementary material for: ChromBPNet: bias factorized, base-resolution deep learning models of chromatin accessibility reveal cis-regulatory sequence syntax, transcription factor footprints and regulatory variants
Source: bioRxiv. 2025 Jan 8:2024.12.25.630221. Preprint. [Version 2] doi: 10.1101/2024.12.25.630221 (PMC11741299; doi:10.1101/2024.12.25.630221)
Supplement: Supplement 4 [file media-4.zip › supplementary_files_3/k562_DNASE_bpnet_bias_model/k562_DNASE_raw_bpnet_bias_fold4_counts_modisco.pdf]

pattern	num_seqlets	cwm_fwd	cwm_rev	TOMTOM_match	TOMTOM_qval	TOMTOM_match_logo
pos_patterns.pattern_0	3985			ZNF384_MA1125.1	1.917260e-01	
pos_patterns.pattern_1	3129			SP2_HUMAN.H11MO.0.A	4.680670e-11	
pos_patterns.pattern_2	1435			SP2_HUMAN.H11MO.0.A	3.113130e-06	
pos_patterns.pattern_3	350			ZNF740_C2H2_2	4.076140e-02	
pos_patterns.pattern_4	322			PRDM6_HUMAN.H11MO.0.C	1.000000e+00	
pos_patterns.pattern_5	122			ZN770_HUMAN.H11MO.0.C	4.871910e-06	
pos_patterns.pattern_6	67			NFAT5_NFAT_1	5.698780e-01	
pos_patterns.pattern_7	62			CPEB1_RRM_1	1.000000e+00	
neg_patterns.pattern_0	1953			MAFK_bZIP_1	1.000000e+00	
neg_patterns.pattern_1	1683			MYB_HUMAN.H11MO.0.A	1.000000e+00	
neg_patterns.pattern_2	1241			ZFX_MOUSE.H11MO.0.B	1.111810e-01	
neg_patterns.pattern_3	469			CTCF_MA0139.1	1.450810e-02	
neg_patterns.pattern_4	359			DNASE_2	2.738920e-03	
neg_patterns.pattern_5	239			NR2C1_HUMAN.H11MO.0.C	5.474930e-01	
neg_patterns.pattern_6	137			KLF5_MOUSE.H11MO.0.A	3.593870e-03	
neg_patterns.pattern_7	109			EGR2_HUMAN.H11MO.0.A	6.224870e-01	
neg_patterns.pattern_8	107			GATA1_HUMAN.H11MO.0.A	7.771510e-03	
neg_patterns.pattern_9	105			EGR2_MOUSE.H11MO.0.A	6.844440e-03	
neg_patterns.pattern_10	102			SALL4_HUMAN.H11MO.0.B	4.211610e-03	
neg_patterns.pattern_11	89			SALL4_HUMAN.H11MO.0.B	4.561280e-01	
neg_patterns.pattern_12	77			ZN281_HUMAN.H11MO.0.A	2.109040e-02	
neg_patterns.pattern_13	69			SP8_C2H2_1	6.087330e-01	
neg_patterns.pattern_14	31			KLF4_MOUSE.H11MO.0.A	1.090060e-02	
neg_patterns.pattern_15	27			CTCFL_HUMAN.H11MO.0.A	7.146070e-02	
neg_patterns.pattern_16	21			FOS_HUMAN.H11MO.0.A	2.965190e-01	
