# Supplementary material for: ChromBPNet: bias factorized, base-resolution deep learning models of chromatin accessibility reveal cis-regulatory sequence syntax, transcription factor footprints and regulatory variants
Source: bioRxiv. 2025 Jan 8:2024.12.25.630221. Preprint. [Version 2] doi: 10.1101/2024.12.25.630221 (PMC11741299; doi:10.1101/2024.12.25.630221)
Supplement: Supplement 4 [file media-4.zip › supplementary_files_3/k562_DNASE_bpnet_bias_model/k562_DNASE_raw_bpnet_bias_fold4_profile_modisco.pdf]

pattern	num_seqlets	cwm_fwd	cwm_rev	TOMTOM_match	TOMTOM_qval	TOMTOM_match_logo
pos_patterns.pattern_0	6193			PRDM9_MOUSE.H11MO.0.C	1.716240e-01	
pos_patterns.pattern_1	4253			ERR2_HUMAN.H11MO.0.A	1.000000e+00	
pos_patterns.pattern_2	2514			DNASE_2	6.439080e-04	
pos_patterns.pattern_3	1772			FOXJ3_HUMAN.H11MO.0.A	2.769370e-01	
pos_patterns.pattern_4	905			ZNF384_MA1125.1	9.670030e-02	
pos_patterns.pattern_5	899			TYY1_HUMAN.H11MO.0.A	5.954010e-02	
pos_patterns.pattern_6	835			Zfx_MA0146.2	5.402300e-01	
pos_patterns.pattern_7	789			MEF2B_HUMAN.H11MO.0.A	2.086250e-01	
pos_patterns.pattern_8	706			DNASE_1	4.744360e-01	
pos_patterns.pattern_9	237			TN5_6	1.480540e-20	
pos_patterns.pattern_10	204			SP1_HUMAN.H11MO.0.A	9.313590e-08	
pos_patterns.pattern_11	198			SP1_MOUSE.H11MO.0.A	7.499990e-01	
pos_patterns.pattern_12	162			ZN770_HUMAN.H11MO.0.C	4.338470e-09	
pos_patterns.pattern_13	133			FOXC1_forkhead_1	6.003630e-02	
pos_patterns.pattern_14	131			ZN770_HUMAN.H11MO.0.C	4.245640e-08	
pos_patterns.pattern_15	113			KLF4_MA0039.3	6.618320e-01	
pos_patterns.pattern_16	111			Arid5a_MA0602.1	1.000000e+00	
pos_patterns.pattern_17	90			IRF3_HUMAN.H11MO.0.B	5.476980e-02	
pos_patterns.pattern_18	81			ZN770_HUMAN.H11MO.0.C	6.396090e-08	
pos_patterns.pattern_19	64			NR4A2_nuclearreceptor_1	6.307350e-01	
pos_patterns.pattern_20	62			ZN121_HUMAN.H11MO.0.C	6.004250e-06	
pos_patterns.pattern_21	61			NKX31_HUMAN.H11MO.0.C	9.798240e-01	
pos_patterns.pattern_22	36			ZN121_HUMAN.H11MO.0.C	1.877100e-02	
pos_patterns.pattern_23	34			ITF2_HUMAN.H11MO.0.C	1.000000e+00	
pos_patterns.pattern_24	27			MAFK_bZIP_3	6.518160e-01	
pos_patterns.pattern_25	24			ZN770_HUMAN.H11MO.0.C	1.622820e-03	
pos_patterns.pattern_26	21			RARA_HUMAN.H11MO.0.A	7.818600e-01	
neg_patterns.pattern_0	50			NF2L2_MOUSE.H11MO.0.A	4.423890e-01	
neg_patterns.pattern_1	47			CPEB1_RRM_1	7.855680e-01	

pattern	num_seqlets	cwm_fwd	cwm_rev	TOMTOM_match	TOMTOM_qval	TOMTOM_match_logo
neg_patterns.pattern_2	45	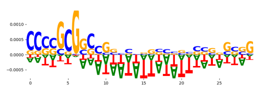	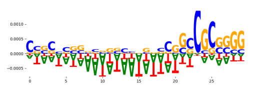	ZNF384_MA1125.1	6.280170e-01	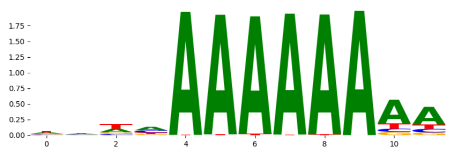
neg_patterns.pattern_3	42	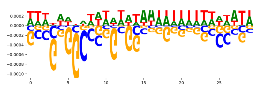	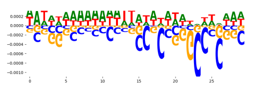	NFATC1_NFAT_1	7.833110e-01	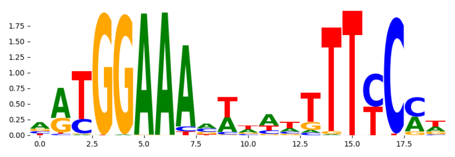
neg_patterns.pattern_4	42	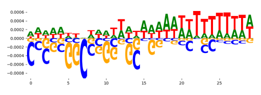	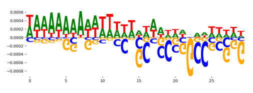	ZNF384_MA1125.1	8.092630e-01	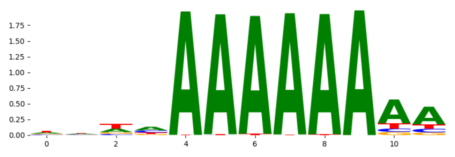
neg_patterns.pattern_5	34	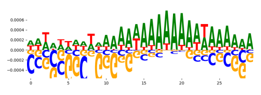	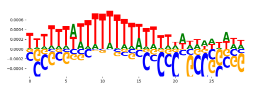	ZNF384_MA1125.1	8.334500e-02	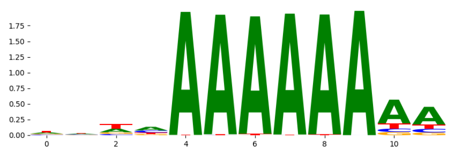
neg_patterns.pattern_6	30	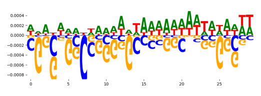	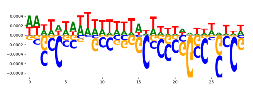	HOXC11_homeodomain_2	2.152600e-01	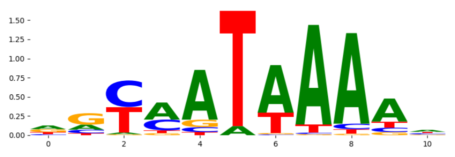
neg_patterns.pattern_7	27	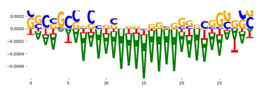	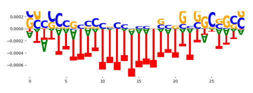	PRDM6_HUMAN.H11MO.0.C	3.082330e-02	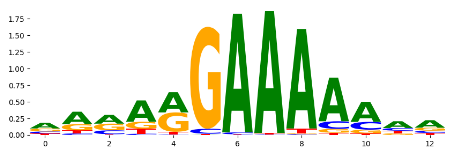
neg_patterns.pattern_8	25	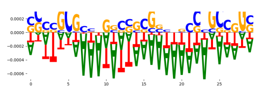	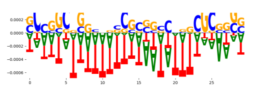	ZNF384_MA1125.1	3.081730e-01	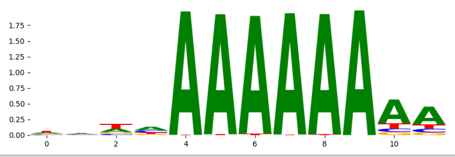
