# Supplementary material for: ChromBPNet: bias factorized, base-resolution deep learning models of chromatin accessibility reveal cis-regulatory sequence syntax, transcription factor footprints and regulatory variants
Source: bioRxiv. 2025 Jan 8:2024.12.25.630221. Preprint. [Version 2] doi: 10.1101/2024.12.25.630221 (PMC11741299; doi:10.1101/2024.12.25.630221)
Supplement: Supplement 4 [file media-4.zip › supplementary_files_3/k562_DNASE_bpnet_bias_model/k562_DNASE_raw_bpnet_bias_fold0_counts_modisco.pdf]

pattern	num_seqlets	cwm_fwd	cwm_rev	TOMTOM_match	TOMTOM_qval	TOMTOM_match_logo
pos_patterns.pattern_0	3334			SP2_HUMAN.H11MO.0.A	3.533940e-09	
pos_patterns.pattern_1	2362			ZNF384_MA1125.1	4.767260e-02	
pos_patterns.pattern_2	1336			DNASE_2	4.345240e-01	
pos_patterns.pattern_3	999			SP2_HUMAN.H11MO.0.A	3.643640e-05	
pos_patterns.pattern_4	616			SP2_HUMAN.H11MO.0.A	3.055720e-04	
pos_patterns.pattern_5	520			PRDM6_HUMAN.H11MO.0.C	1.157740e-02	
pos_patterns.pattern_6	491			FOXB1_forkhead_2	1.576520e-01	
pos_patterns.pattern_7	447			FOXG1_forkhead_1	5.712610e-01	
pos_patterns.pattern_8	238			IRF1_HUMAN.H11MO.0.A	2.938620e-02	
pos_patterns.pattern_9	227			STAT1_MOUSE.H11MO.0.A	3.865050e-01	
pos_patterns.pattern_10	219			NaN	NaN	
pos_patterns.pattern_11	144			ZIM3_HUMAN.H11MO.0.C	1.000000e+00	
pos_patterns.pattern_12	55			SOX8_HMG_3	1.000000e+00	
neg_patterns.pattern_0	1544			DNASE_1	1.000000e+00	
neg_patterns.pattern_1	1309			MYB_HUMAN.H11MO.0.A	2.222720e-01	
neg_patterns.pattern_2	1001			NFIC_MA0161.2	8.809010e-01	
neg_patterns.pattern_3	634			CTCF_C2H2_1	2.439580e-01	
neg_patterns.pattern_4	490			DNASE_2	8.722250e-02	
neg_patterns.pattern_5	457			SALL4_HUMAN.H11MO.0.B	8.932590e-02	
neg_patterns.pattern_6	371			COE1_MOUSE.H11MO.0.A	6.161280e-04	
neg_patterns.pattern_7	291			NHLH1_MA0048.2	5.711200e-02	
neg_patterns.pattern_8	274			NR2E1_nuclearreceptor_2	1.000000e+00	
neg_patterns.pattern_9	264			NFIC_HUMAN.H11MO.0.A	3.037560e-01	
neg_patterns.pattern_10	247			P73_HUMAN.H11MO.0.A	4.394040e-03	
neg_patterns.pattern_11	166			ZN257_HUMAN.H11MO.0.C	6.461030e-01	
neg_patterns.pattern_12	103			ZN770_HUMAN.H11MO.0.C	5.231240e-01	
neg_patterns.pattern_13	56			ZFX_MOUSE.H11MO.0.B	3.834680e-01	
neg_patterns.pattern_14	28			ZN547_HUMAN.H11MO.0.C	7.633560e-02	
