# Supplementary material for: ChromBPNet: bias factorized, base-resolution deep learning models of chromatin accessibility reveal cis-regulatory sequence syntax, transcription factor footprints and regulatory variants
Source: bioRxiv. 2025 Jan 8:2024.12.25.630221. Preprint. [Version 2] doi: 10.1101/2024.12.25.630221 (PMC11741299; doi:10.1101/2024.12.25.630221)
Supplement: Supplement 4 [file media-4.zip › supplementary_files_3/k562_DNASE_bpnet_bias_model/k562_DNASE_raw_bpnet_bias_fold3_counts_modisco.pdf]

pattern	num_seqlets	cwm_fwd	cwm_rev	TOMTOM_match	TOMTOM_qval	TOMTOM_match_logo
pos_patterns.pattern_0	3602			ZNF384_MA1125.1	2.739470e-02	
pos_patterns.pattern_1	2664			SP1_MOUSE.H11MO.0.A	1.288410e-10	
pos_patterns.pattern_2	1720			SP2_HUMAN.H11MO.0.A	8.576680e-06	
pos_patterns.pattern_3	722			Foxj3_MA0851.1	1.000390e-01	
pos_patterns.pattern_4	539			SP1_HUMAN.H11MO.0.A	4.485270e-02	
pos_patterns.pattern_5	194			SP2_HUMAN.H11MO.0.A	1.525860e-02	
neg_patterns.pattern_0	1725			DNASE_2	6.793600e-03	
neg_patterns.pattern_1	1316			INSM1_MA0155.1	9.916480e-01	
neg_patterns.pattern_2	574			TFE2_HUMAN.H11MO.0.A	1.000000e+00	
neg_patterns.pattern_3	480			DNASE_1	2.286400e-01	
neg_patterns.pattern_4	350			Hic1.mouse_C2H2_1	2.417530e-01	
neg_patterns.pattern_5	263			KLF4_MA0039.3	1.057400e-01	
neg_patterns.pattern_6	241			DNASE_2	7.352590e-01	
neg_patterns.pattern_7	209			ISL2_MA0914.1	9.226180e-01	
neg_patterns.pattern_8	201			ZN770_HUMAN.H11MO.0.C	1.000000e+00	
neg_patterns.pattern_9	173			CLOCK_HUMAN.H11MO.0.C	6.414540e-01	
neg_patterns.pattern_10	128			DNASE_5	4.153550e-05	
neg_patterns.pattern_11	108			DNASE_3	7.218670e-01	
neg_patterns.pattern_12	97			ZNF384_MA1125.1	8.771860e-02	
neg_patterns.pattern_13	65			TN5_6	4.132790e-15	
neg_patterns.pattern_14	45			PTF1A_HUMAN.H11MO.0.B	2.151590e-01	
neg_patterns.pattern_15	44			PATZ1_HUMAN.H11MO.0.C	2.255090e-02	
neg_patterns.pattern_16	42			Hoxd8_MA0910.1	1.000000e+00	
neg_patterns.pattern_17	34			DNASE_3	2.315990e-02	
