# Supplementary material for: ChromBPNet: bias factorized, base-resolution deep learning models of chromatin accessibility reveal cis-regulatory sequence syntax, transcription factor footprints and regulatory variants
Source: bioRxiv. 2025 Jan 8:2024.12.25.630221. Preprint. [Version 2] doi: 10.1101/2024.12.25.630221 (PMC11741299; doi:10.1101/2024.12.25.630221)
Supplement: Supplement 4 [file media-4.zip › supplementary_files_3/k562_ATAC_bpnet_bias_model/k562_ATAC_raw_bpnet_bias_fold0_counts_modisco.pdf]

pattern	num_seqlets	cwm_fwd	cwm_rev	TOMTOM_match	TOMTOM_qval	TOMTOM_match_logo
pos_patterns.pattern_0	4623			Zfx_MA0146.2	7.514190e-02	
pos_patterns.pattern_1	4509			SP3_HUMAN.H11MO.0.B	6.076060e-02	
pos_patterns.pattern_2	4452			COE1_HUMAN.H11MO.0.A	1.612260e-01	
pos_patterns.pattern_3	3732			Zfx_MA0146.2	7.383070e-02	
pos_patterns.pattern_4	1924			PRDM6_HUMAN.H11MO.0.C	3.509900e-03	
pos_patterns.pattern_5	1387			SP1_HUMAN.H11MO.0.A	6.590080e-03	
pos_patterns.pattern_6	1248			NFAC2_HUMAN.H11MO.0.B	1.463050e-01	
pos_patterns.pattern_7	1105			ZFX_MOUSE.H11MO.0.B	4.500500e-02	
pos_patterns.pattern_8	1097			EHF_HUMAN.H11MO.0.B	5.983360e-01	
pos_patterns.pattern_9	981			SP5_MOUSE.H11MO.0.C	1.013310e-08	
pos_patterns.pattern_10	923			HNF6_HUMAN.H11MO.0.B	1.069850e-01	
pos_patterns.pattern_11	895			WT1_HUMAN.H11MO.0.C	4.402920e-02	
pos_patterns.pattern_12	856			PRDM6_HUMAN.H11MO.0.C	1.328680e-03	
pos_patterns.pattern_13	815			PTF1A_HUMAN.H11MO.0.B	3.291000e-02	
pos_patterns.pattern_14	785			SP5_MOUSE.H11MO.0.C	2.431270e-08	
pos_patterns.pattern_15	772			Zfp740.mouse_C2H2_1	4.421730e-01	
pos_patterns.pattern_16	665			ZNF384_MA1125.1	2.182940e-01	
pos_patterns.pattern_17	640			NFKB1_HUMAN.H11MO.1.B	6.221000e-01	
pos_patterns.pattern_18	575			COE1_MOUSE.H11MO.0.A	1.000000e+00	
pos_patterns.pattern_19	575			ZN320_HUMAN.H11MO.0.C	8.931030e-01	
pos_patterns.pattern_20	545			Zfx_MA0146.2	1.894700e-01	
pos_patterns.pattern_21	506			ZN490_HUMAN.H11MO.0.C	1.000000e+00	
pos_patterns.pattern_22	481			GATA1_MOUSE.H11MO.0.A	8.308370e-01	
pos_patterns.pattern_23	466			NaN	NaN	
pos_patterns.pattern_24	319			NaN	NaN	
pos_patterns.pattern_25	302			USF2_HUMAN.H11MO.0.A	3.102670e-01	
pos_patterns.pattern_26	256			NR1H3_MOUSE.H11MO.0.A	9.424250e-01	
pos_patterns.pattern_27	201			PAX5_HUMAN.H11MO.0.A	4.151330e-01	
pos_patterns.pattern_28	189			GRHL1_CP2_2	1.000000e+00	
pos_patterns.pattern_29	185			IRF3_HUMAN.H11MO.0.B	2.067950e-01	

pattern	num_seqlets	cwm_fwd	cwm_rev	TOMTOM_match	TOMTOM_qval	TOMTOM_match_logo
pos_patterns.pattern_30	180			MBD2_HUMAN.H11MO.0.B	1.000000e+00	
pos_patterns.pattern_31	162			MAZ_HUMAN.H11MO.0.A	1.468100e-01	
pos_patterns.pattern_32	121			ZN274_HUMAN.H11MO.0.A	1.000000e+00	
pos_patterns.pattern_33	126			HIC1_HUMAN.H11MO.0.C	5.295130e-02	
pos_patterns.pattern_34	103			TN5_4	7.348250e-01	
pos_patterns.pattern_35	100			ZFP57_MOUSE.H11MO.0.B	1.000000e+00	
pos_patterns.pattern_36	67			SP1_HUMAN.H11MO.0.A	1.000000e+00	
pos_patterns.pattern_37	60			NR1H4_HUMAN.H11MO.0.B	4.044930e-02	
pos_patterns.pattern_38	61			ZN121_HUMAN.H11MO.0.C	1.326440e-02	
pos_patterns.pattern_39	63			TP53_MA0106.3	5.534980e-01	
pos_patterns.pattern_40	51			TGIF2_MA0797.1	4.873440e-02	
pos_patterns.pattern_41	49			SP1_HUMAN.H11MO.0.A	1.341510e-01	
pos_patterns.pattern_42	44			SMAD2_MOUSE.H11MO.0.A	1.000000e+00	
pos_patterns.pattern_43	46			SP5_MOUSE.H11MO.0.C	8.803520e-03	
pos_patterns.pattern_44	46			ZSCAN4_C2H2_1	9.999990e-01	
pos_patterns.pattern_45	33			VDR_HUMAN.H11MO.0.A	2.861960e-01	
pos_patterns.pattern_46	38			ESR2_HUMAN.H11MO.0.A	2.129400e-01	
pos_patterns.pattern_47	35			ZNF41_HUMAN.H11MO.0.C	1.000000e+00	
neg_patterns.pattern_0	827			SP3_HUMAN.H11MO.0.B	1.602600e-08	
neg_patterns.pattern_1	616			TN5_2	6.234200e-04	
neg_patterns.pattern_2	368			ZNF384_MA1125.1	9.593720e-04	
neg_patterns.pattern_3	365			TN5_1	1.743260e-03	
neg_patterns.pattern_4	312			TN5_7	2.519440e-02	
neg_patterns.pattern_5	312			SMAD4_HUMAN.H11MO.0.B	6.954020e-02	
neg_patterns.pattern_6	274			NR1D2_MOUSE.H11MO.0.A	8.128240e-01	
neg_patterns.pattern_7	277			Myog_MA0500.1	6.288240e-01	
neg_patterns.pattern_8	198			SP1_MOUSE.H11MO.0.A	5.516340e-04	
neg_patterns.pattern_9	210			SP3_HUMAN.H11MO.0.B	2.494550e-03	
neg_patterns.pattern_10	211			ELF2_MOUSE.H11MO.0.C	2.268930e-01	
neg_patterns.pattern_11	199			USF2_HUMAN.H11MO.0.A	1.629330e-01	

pattern	num_seqlets	cwm_fwd	cwm_rev	TOMTOM_match	TOMTOM_qval	TOMTOM_match_logo
neg_patterns.pattern_12	138	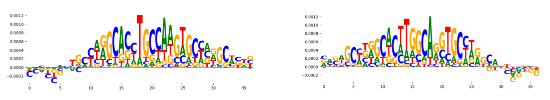		HES5_MA0821.1	3.995470e-03	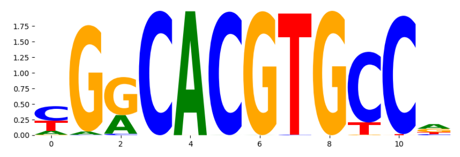
neg_patterns.pattern_13	110	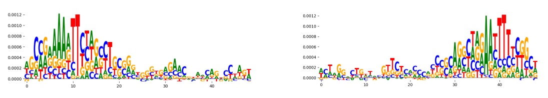		SP2_HUMAN.H11MO.0.A	1.638920e-05	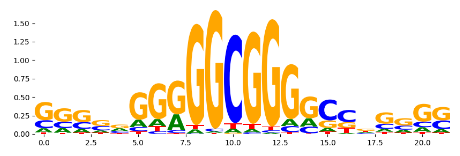
neg_patterns.pattern_14	96	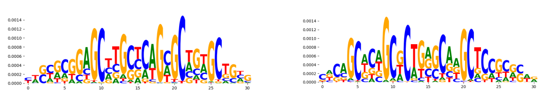		THAP1_HUMAN.H11MO.0.C	1.000000e+00	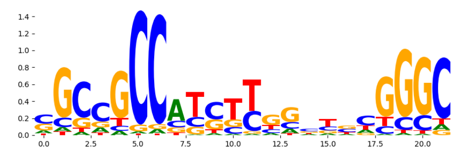
neg_patterns.pattern_15	64	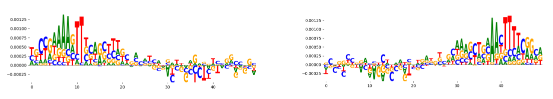		SP1_HUMAN.H11MO.0.A	5.133080e-03	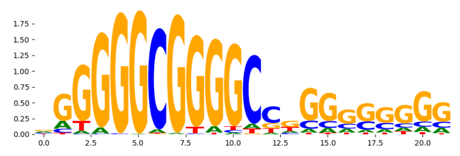
neg_patterns.pattern_16	71	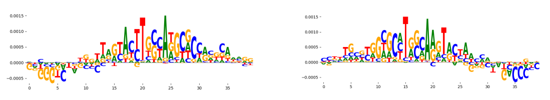		NRF1_MOUSE.H11MO.0.A	4.433670e-02	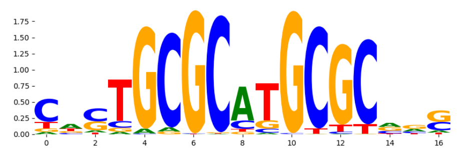
neg_patterns.pattern_17	49	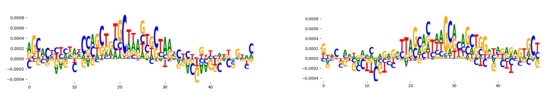		SP2_HUMAN.H11MO.0.A	5.241080e-03	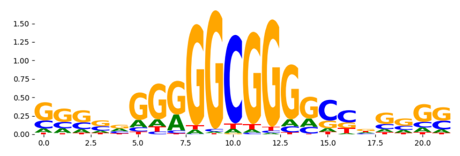
neg_patterns.pattern_18	78	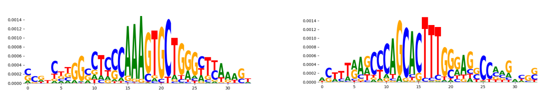		IKZF1_HUMAN.H11MO.0.C	1.000000e+00	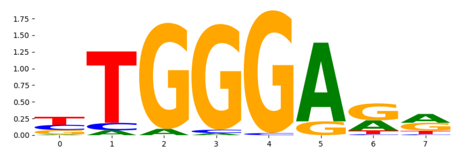
neg_patterns.pattern_19	36	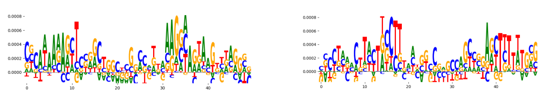		ZFX_MOUSE.H11MO.0.B	4.960440e-03	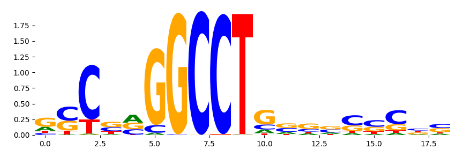
neg_patterns.pattern_20	36	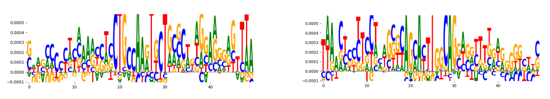		NFIB_MOUSE.H11MO.0.C	8.919150e-03	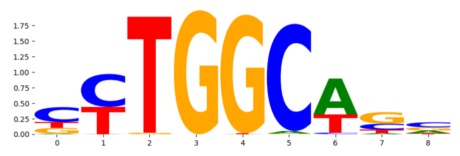
neg_patterns.pattern_21	35	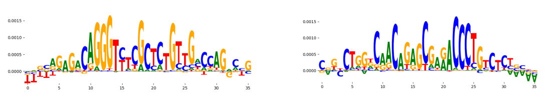		ZN121_HUMAN.H11MO.0.C	1.341930e-08	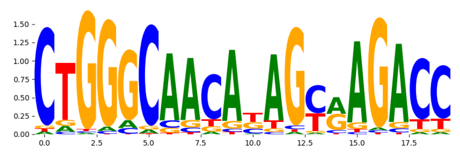
neg_patterns.pattern_22	29	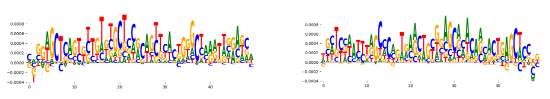		TN5_2	1.257090e-01	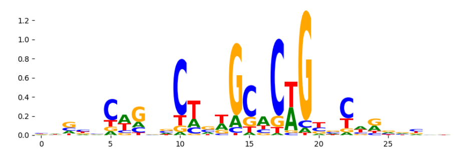
neg_patterns.pattern_23	30	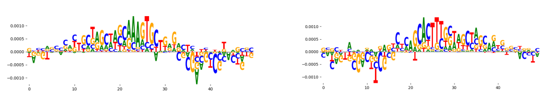		TN5_2	4.920810e-02	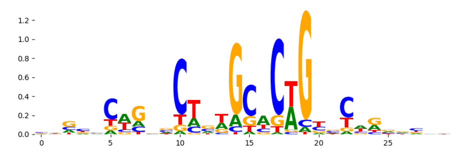
neg_patterns.pattern_24	39	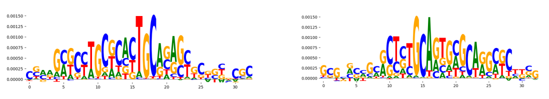		NRF1_MA0506.1	2.476270e-01	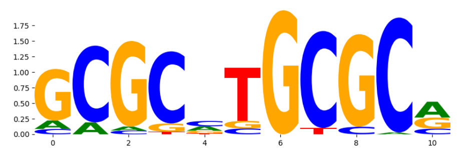
neg_patterns.pattern_25	30	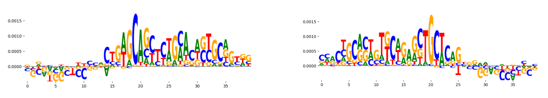		TN5_7	2.002190e-01	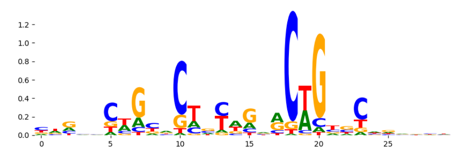
neg_patterns.pattern_26	27	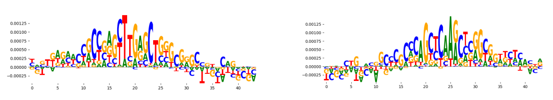		KLF3_HUMAN.H11MO.0.B	2.150620e-01	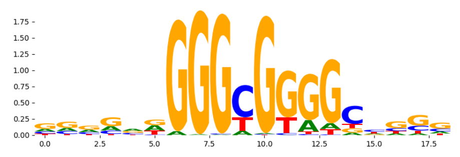
neg_patterns.pattern_27	27	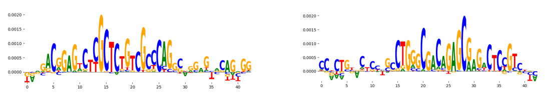		TN5_6	3.212420e-12	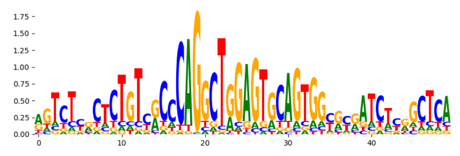
neg_patterns.pattern_28	26	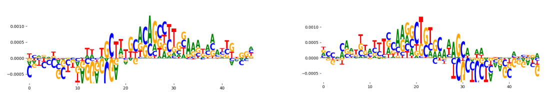		ZFX_MOUSE.H11MO.0.B	3.639610e-02	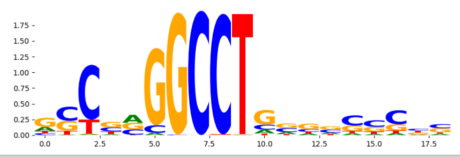
