# Supplementary material for: ChromBPNet: bias factorized, base-resolution deep learning models of chromatin accessibility reveal cis-regulatory sequence syntax, transcription factor footprints and regulatory variants
Source: bioRxiv. 2025 Jan 8:2024.12.25.630221. Preprint. [Version 2] doi: 10.1101/2024.12.25.630221 (PMC11741299; doi:10.1101/2024.12.25.630221)
Supplement: Supplement 4 [file media-4.zip › supplementary_files_3/k562_ATAC_bpnet_bias_model/k562_ATAC_raw_bpnet_bias_fold3_counts_modisco.pdf]

pattern	num_seqlets	cwm_fwd	cwm_rev	TOMTOM_match	TOMTOM_qval	TOMTOM_match_logo
pos_patterns.pattern_0	6027			NaN	NaN	
pos_patterns.pattern_1	4358			ZFX_MOUSE.H11MO.0.B	1.327300e-01	
pos_patterns.pattern_2	2839			ZBT7A_HUMAN.H11MO.0.A	7.431000e-02	
pos_patterns.pattern_3	925			HSF2_HSF_1	1.000000e+00	
pos_patterns.pattern_4	813			RREB1_MA0073.1	4.534670e-02	
pos_patterns.pattern_5	808			ZN436_HUMAN.H11MO.0.C	1.000000e+00	
pos_patterns.pattern_6	608			ZNF384_MA1125.1	3.313860e-02	
pos_patterns.pattern_7	539			DNASE_4	1.000000e+00	
pos_patterns.pattern_8	418			Zfx_MA0146.2	1.000000e+00	
pos_patterns.pattern_9	371			EGR2_HUMAN.H11MO.0.A	1.294610e-03	
pos_patterns.pattern_10	355			RUNX2_HUMAN.H11MO.0.A	6.378170e-01	
pos_patterns.pattern_11	199			SOX9_HMG_1	1.000000e+00	
pos_patterns.pattern_12	188			VEZF1_HUMAN.H11MO.0.C	1.167430e-04	
pos_patterns.pattern_13	181			SP1_HUMAN.H11MO.0.A	1.860660e-06	
pos_patterns.pattern_14	160			SP2_HUMAN.H11MO.0.A	2.389050e-11	
pos_patterns.pattern_15	160			EGR2_HUMAN.H11MO.0.A	1.797250e-04	
pos_patterns.pattern_16	156			NFAC1_HUMAN.H11MO.0.B	2.777270e-01	
pos_patterns.pattern_17	150			SP5_MOUSE.H11MO.0.C	4.528580e-05	
pos_patterns.pattern_18	55			HNF4A_nuclearreceptor_1	4.168720e-01	
pos_patterns.pattern_19	54			SP3_HUMAN.H11MO.0.B	5.632300e-04	
pos_patterns.pattern_20	53			SP3_HUMAN.H11MO.0.B	3.547680e-05	
pos_patterns.pattern_21	22			ZN263_HUMAN.H11MO.0.A	1.255920e-01	
neg_patterns.pattern_0	110			TN5_2	1.074060e-03	
neg_patterns.pattern_1	99			SP2_HUMAN.H11MO.0.A	5.572160e-06	
neg_patterns.pattern_2	91			ZFX_MOUSE.H11MO.0.B	1.269110e-03	
neg_patterns.pattern_3	89			TN5_2	3.501280e-01	
neg_patterns.pattern_4	82			DNASE_3	1.000000e+00	
neg_patterns.pattern_5	82			ZN335_HUMAN.H11MO.0.A	1.000000e+00	
neg_patterns.pattern_6	81			ZN331_HUMAN.H11MO.0.C	6.314360e-01	
neg_patterns.pattern_7	80			SUH_HUMAN.H11MO.0.A	9.042350e-01	

pattern	num_seqlets	cwm_fwd	cwm_rev	TOMTOM_match	TOMTOM_qval	TOMTOM_match_logo
neg_patterns.pattern_8	75	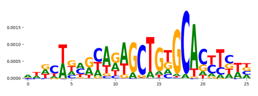	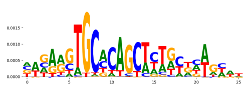	ZFX_MOUSE.H11MO.0.B	1.537290e-02	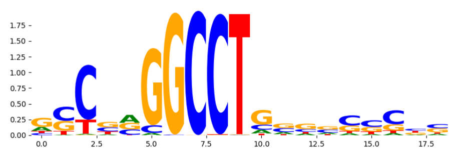
neg_patterns.pattern_9	74	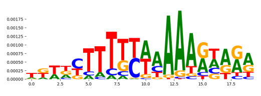	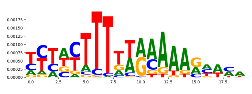	Stat4_MA0518.1	5.268070e-01	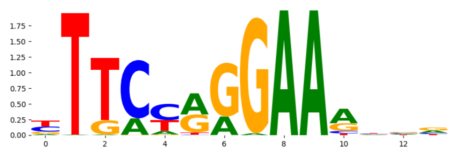
neg_patterns.pattern_10	62	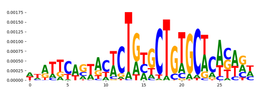	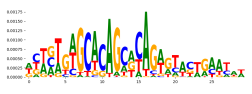	TN5_2	3.466260e-03	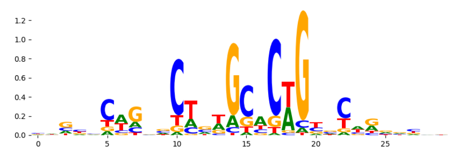
neg_patterns.pattern_11	59	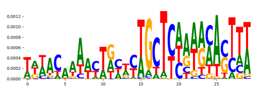	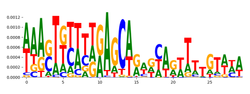	ZEB1_MA0103.3	8.601340e-02	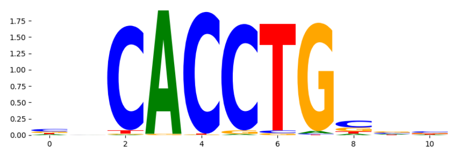
neg_patterns.pattern_12	56	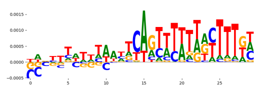	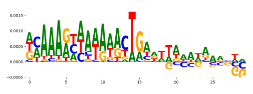	ZN281_MOUSE.H11MO.0.A	1.935400e-01	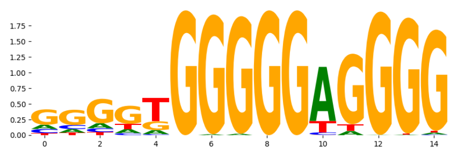
neg_patterns.pattern_13	50	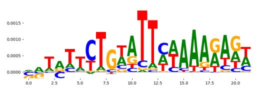	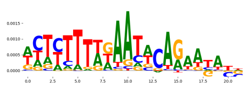	ZN490_HUMAN.H11MO.0.C	8.361060e-01	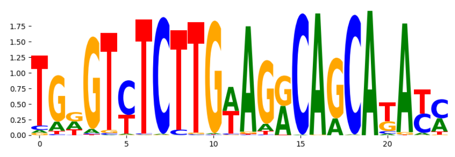
neg_patterns.pattern_14	26	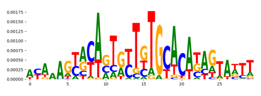	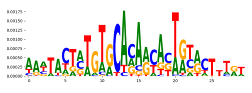	TN5_2	4.132160e-04	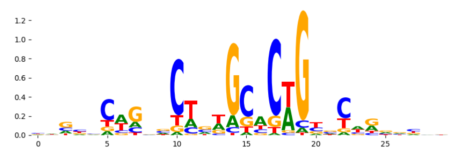
neg_patterns.pattern_15	23	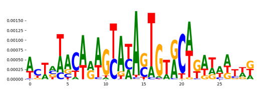	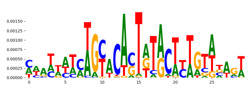	TN5_2	3.918880e-02	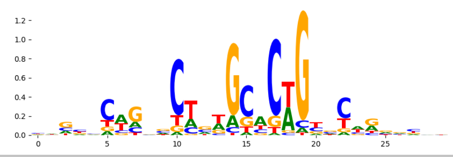
