# Supplementary material for: ChromBPNet: bias factorized, base-resolution deep learning models of chromatin accessibility reveal cis-regulatory sequence syntax, transcription factor footprints and regulatory variants
Source: bioRxiv. 2025 Jan 8:2024.12.25.630221. Preprint. [Version 2] doi: 10.1101/2024.12.25.630221 (PMC11741299; doi:10.1101/2024.12.25.630221)
Supplement: Supplement 4 [file media-4.zip › supplementary_files_3/k562_ATAC_bpnet_bias_model/k562_ATAC_raw_bpnet_bias_fold2_counts_modisco.pdf]

pattern	num_seqlets	cwm_fwd	cwm_rev	TOMTOM_match	TOMTOM_qval	TOMTOM_match_logo
pos_patterns.pattern_0	7909			SP2_HUMAN.H11MO.0.A	1.000000	
pos_patterns.pattern_1	4005			ZFX_MOUSE.H11MO.0.B	0.040608	
pos_patterns.pattern_2	827			ZNF384_MA1125.1	0.006267	
pos_patterns.pattern_3	785			NFATC1_NFAT_1	1.000000	
pos_patterns.pattern_4	733			ZNF524_C2H2_1	1.000000	
pos_patterns.pattern_5	681			NFAC3_HUMAN.H11MO.0.B	0.096131	
pos_patterns.pattern_6	550			ZN214_HUMAN.H11MO.0.C	1.000000	
pos_patterns.pattern_7	525			SP3_HUMAN.H11MO.0.B	0.000057	
pos_patterns.pattern_8	502			ZBT7A_HUMAN.H11MO.0.A	0.439502	
pos_patterns.pattern_9	484			PBX1_MA0070.1	1.000000	
pos_patterns.pattern_10	458			PATZ1_HUMAN.H11MO.0.C	0.000444	
pos_patterns.pattern_11	433			SP1_HUMAN.H11MO.0.A	0.029421	
pos_patterns.pattern_12	283			ESR1_HUMAN.H11MO.0.A	1.000000	
pos_patterns.pattern_13	226			EGR2_HUMAN.H11MO.0.A	0.001542	
pos_patterns.pattern_14	199			SP2_HUMAN.H11MO.0.A	0.419817	
pos_patterns.pattern_15	198			AP2C_MOUSE.H11MO.0.A	0.190258	
pos_patterns.pattern_16	183			ZN281_MOUSE.H11MO.0.A	0.002343	
pos_patterns.pattern_17	174			TCF7L2_MA0523.1	0.410400	
pos_patterns.pattern_18	124			ARI5B_HUMAN.H11MO.0.C	1.000000	
pos_patterns.pattern_19	22			ZN320_HUMAN.H11MO.0.C	0.415961	
neg_patterns.pattern_0	272			TN5_2	0.300609	
neg_patterns.pattern_1	160			NHLH1_bHLH_1	0.006659	
neg_patterns.pattern_2	141			ZFX_MOUSE.H11MO.0.B	0.039905	
neg_patterns.pattern_3	139			THAP1_HUMAN.H11MO.0.C	0.000705	
neg_patterns.pattern_4	124			KLF6_HUMAN.H11MO.0.A	0.444607	
neg_patterns.pattern_5	112			ZN331_HUMAN.H11MO.0.C	0.100857	
neg_patterns.pattern_6	96			THAP1_HUMAN.H11MO.0.C	0.089762	
neg_patterns.pattern_7	94			TN5_2	0.215890	
neg_patterns.pattern_8	94			SP2_HUMAN.H11MO.0.A	0.003688	

pattern	num_seqlets	cwm_fwd	cwm_rev	TOMTOM_match	TOMTOM_qval	TOMTOM_match_logo
neg_patterns.pattern_9	85	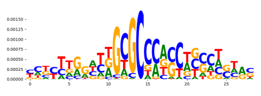	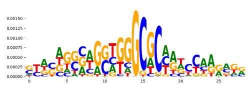	SP2_HUMAN.H11MO.0.A	0.028890	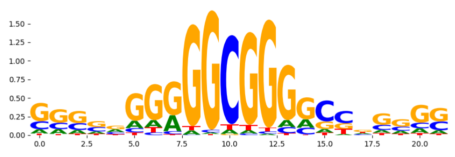
neg_patterns.pattern_10	83	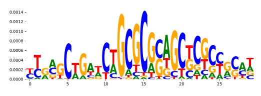	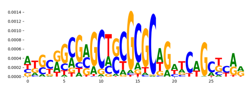	SP2_HUMAN.H11MO.0.A	0.001120	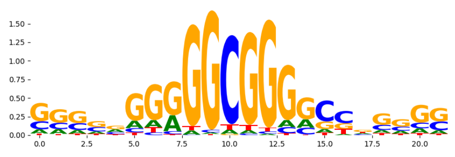
neg_patterns.pattern_11	81	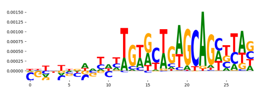	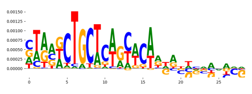	ZN331_HUMAN.H11MO.0.C	0.688227	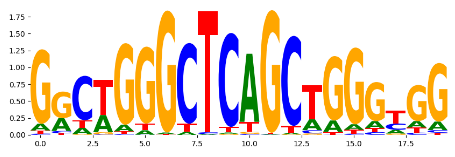
neg_patterns.pattern_12	80	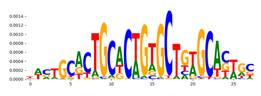	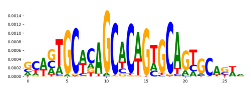	TN5_2	0.004024	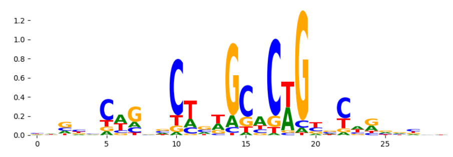
neg_patterns.pattern_13	70	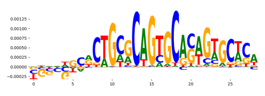	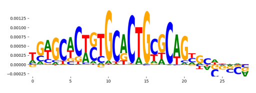	TN5_2	0.001492	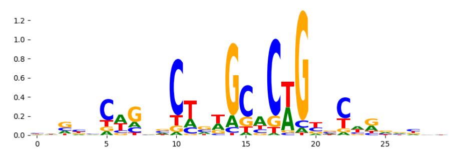
neg_patterns.pattern_14	70	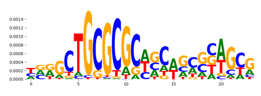	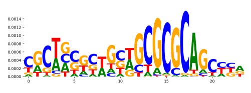	ZBT14_HUMAN.H11MO.0.C	0.274724	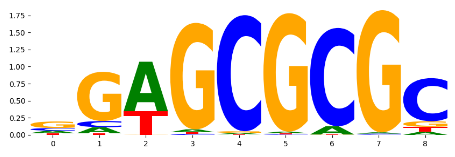
neg_patterns.pattern_15	68	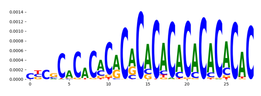	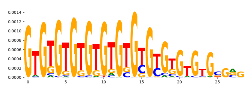	RREB1_MA0073.1	1.000000	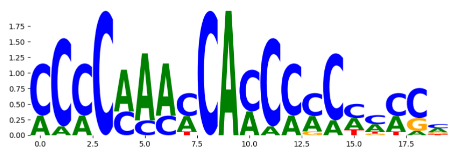
neg_patterns.pattern_16	66	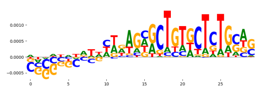	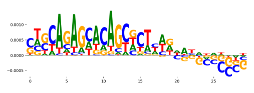	TN5_2	0.007280	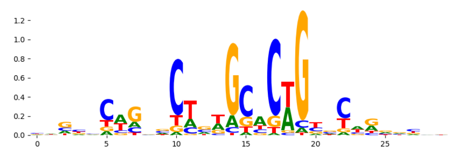
neg_patterns.pattern_17	64	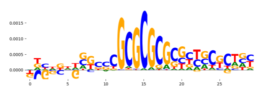	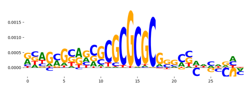	SP2_HUMAN.H11MO.0.A	0.000072	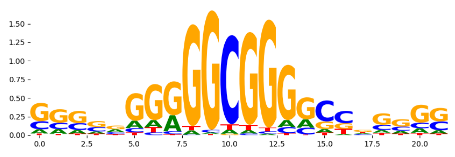
neg_patterns.pattern_18	61	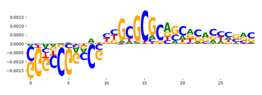	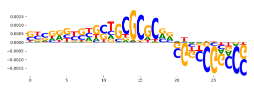	SP2_HUMAN.H11MO.0.A	0.000105	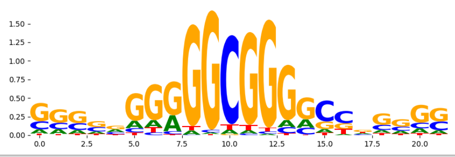
