# Supplementary material for: ChromBPNet: bias factorized, base-resolution deep learning models of chromatin accessibility reveal cis-regulatory sequence syntax, transcription factor footprints and regulatory variants
Source: bioRxiv. 2025 Jan 8:2024.12.25.630221. Preprint. [Version 2] doi: 10.1101/2024.12.25.630221 (PMC11741299; doi:10.1101/2024.12.25.630221)
Supplement: Supplement 4 [file media-4.zip › supplementary_files_3/k562_ATAC_bpnet_bias_model/k562_ATAC_raw_bpnet_bias_fold1_counts_modisco.pdf]

pattern	num_seqlets	cwm_fwd	cwm_rev	TOMTOM_match	TOMTOM_qval	TOMTOM_match_logo
pos_patterns.pattern_0	4940			KLF12_HUMAN.H11MO.0.C	1.784760e-01	
pos_patterns.pattern_1	3022			SP2_HUMAN.H11MO.0.A	9.521130e-03	
pos_patterns.pattern_2	1786			ZBT7A_HUMAN.H11MO.0.A	1.350880e-01	
pos_patterns.pattern_3	954			RREB1_MA0073.1	1.000000e+00	
pos_patterns.pattern_4	773			TN5_2	1.978000e-01	
pos_patterns.pattern_5	764			SOX10_HMG_5	1.000000e+00	
pos_patterns.pattern_6	539			TN5_1	1.000000e+00	
pos_patterns.pattern_7	531			ANDR_HUMAN.H11MO.0.A	2.642490e-01	
pos_patterns.pattern_8	531			HIC1_HUMAN.H11MO.0.C	1.899190e-01	
pos_patterns.pattern_9	518			ZFP57_MOUSE.H11MO.0.B	1.000000e+00	
pos_patterns.pattern_10	496			ETS1_HUMAN.H11MO.0.A	7.790330e-01	
pos_patterns.pattern_11	464			SP2_HUMAN.H11MO.0.A	5.011730e-01	
pos_patterns.pattern_12	454			ARI5B_HUMAN.H11MO.0.C	1.000000e+00	
pos_patterns.pattern_13	306			RREB1_MA0073.1	2.799840e-05	
pos_patterns.pattern_14	271			ESR2_HUMAN.H11MO.0.A	2.373130e-02	
pos_patterns.pattern_15	236			ZNF740_C2H2_2	4.588520e-02	
pos_patterns.pattern_16	232			OSR2_HUMAN.H11MO.0.C	3.293840e-03	
pos_patterns.pattern_17	214			SP2_HUMAN.H11MO.0.A	1.313400e-04	
pos_patterns.pattern_18	208			ANDR_HUMAN.H11MO.0.A	8.861060e-01	
pos_patterns.pattern_19	165			PATZ1_HUMAN.H11MO.0.C	1.635340e-03	
pos_patterns.pattern_20	160			ZN467_HUMAN.H11MO.0.C	1.705560e-04	
pos_patterns.pattern_21	145			SP1_HUMAN.H11MO.0.A	3.274330e-07	
pos_patterns.pattern_22	117			SP1_MOUSE.H11MO.0.A	7.407780e-07	
pos_patterns.pattern_23	74			P63_HUMAN.H11MO.0.A	5.037470e-01	
pos_patterns.pattern_24	67			SP5_MOUSE.H11MO.0.C	2.436330e-02	
pos_patterns.pattern_25	36			TP63_MA0525.2	4.339640e-01	
neg_patterns.pattern_0	139			SP2_HUMAN.H11MO.0.A	8.064840e-07	
neg_patterns.pattern_1	135			SP1_HUMAN.H11MO.0.A	5.436040e-02	
neg_patterns.pattern_2	118			ZN331_HUMAN.H11MO.0.C	1.831570e-03	

pattern	num_seqlets	cwm_fwd	cwm_rev	TOMTOM_match	TOMTOM_qval	TOMTOM_match_logo
neg_patterns.pattern_3	115	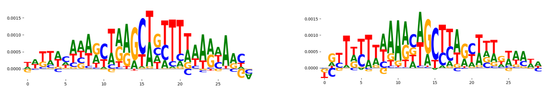		ZEB1_MA0103.3	1.000000e+00	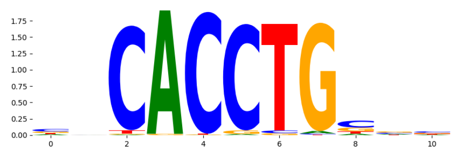
neg_patterns.pattern_4	108	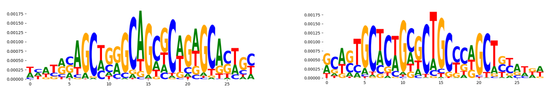		SP1_MOUSE.H11MO.0.A	2.597490e-02	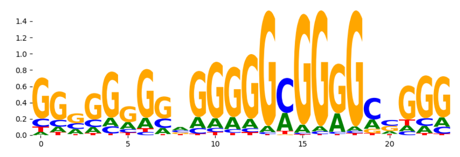
neg_patterns.pattern_5	108	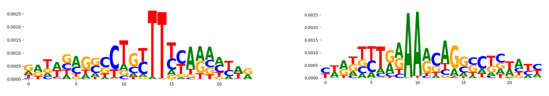		RFX6_MOUSE.H11MO.0.C	1.000000e+00	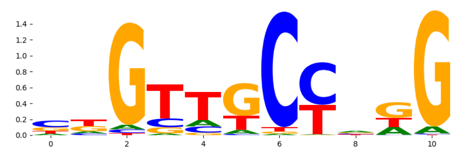
neg_patterns.pattern_6	100	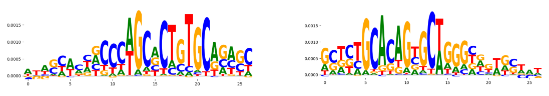		ZFX_MOUSE.H11MO.0.B	1.165950e-02	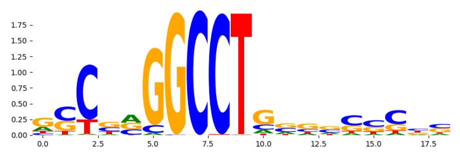
neg_patterns.pattern_7	97	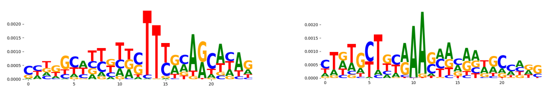		RFX1_HUMAN.H11MO.0.B	8.414950e-02	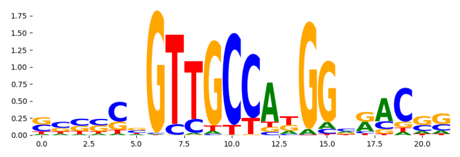
neg_patterns.pattern_8	88	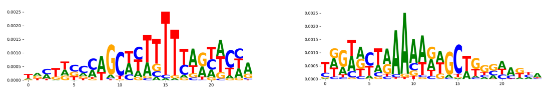		SP3_HUMAN.H11MO.0.B	4.724440e-01	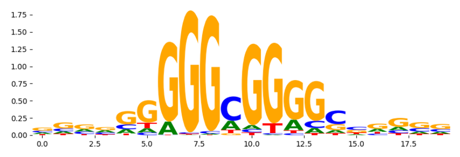
neg_patterns.pattern_9	67	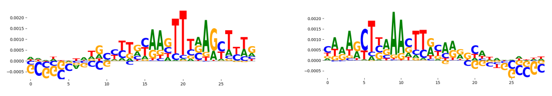		Zfx_MA0146.2	7.526160e-03	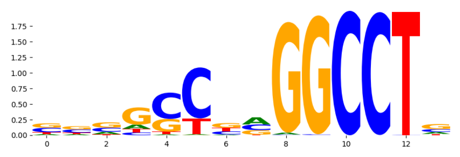
neg_patterns.pattern_10	65	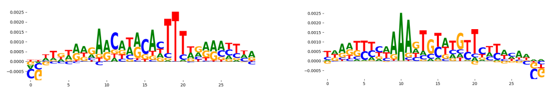		SP1_MOUSE.H11MO.0.A	4.318450e-02	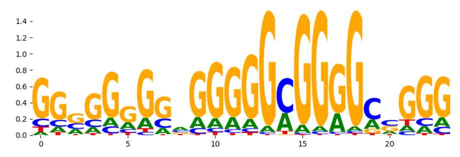
neg_patterns.pattern_11	49	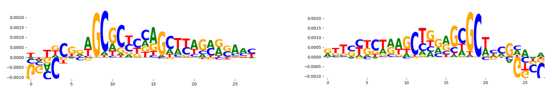		SP2_HUMAN.H11MO.0.A	3.653230e-03	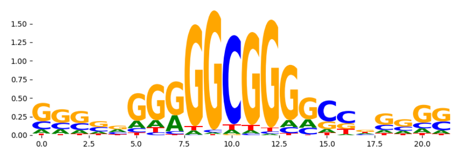
neg_patterns.pattern_12	45	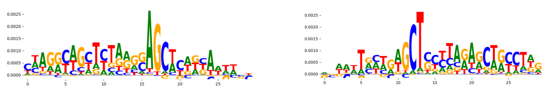		TN5_7	3.167920e-01	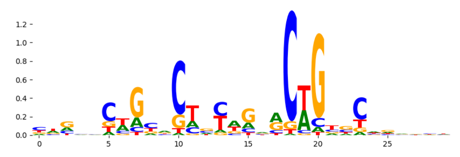
neg_patterns.pattern_13	43	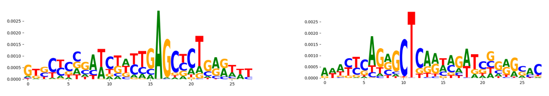		SP1_HUMAN.H11MO.0.A	1.831900e-01	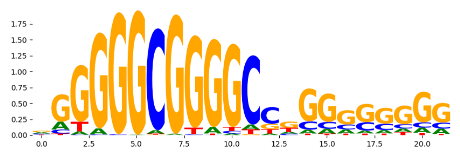
neg_patterns.pattern_14	42	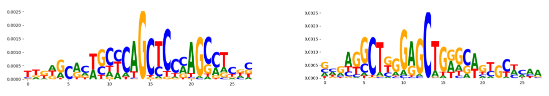		SP1_HUMAN.H11MO.0.A	3.663470e-04	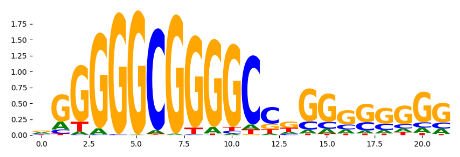
neg_patterns.pattern_15	38	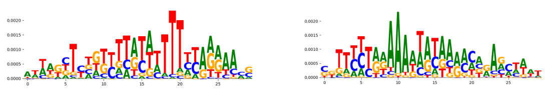		BCL6_HUMAN.H11MO.0.A	1.759410e-01	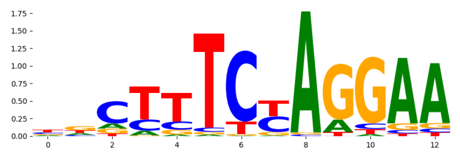
neg_patterns.pattern_16	38	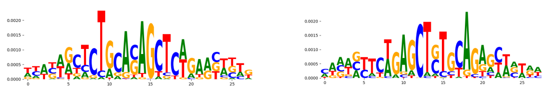		TN5_2	3.021440e-02	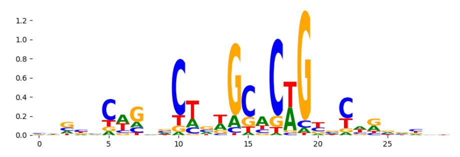
neg_patterns.pattern_17	35	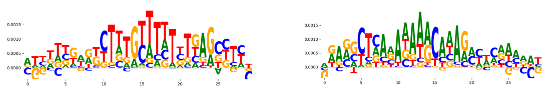		Zic3.mouse_C2H2_1	5.698560e-01	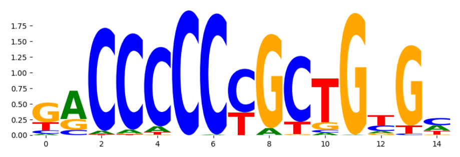
neg_patterns.pattern_18	29	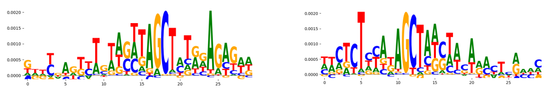		ZFX_MOUSE.H11MO.0.B	2.038440e-01	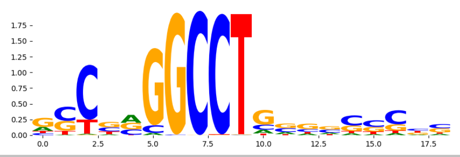
