# Supplementary material for: ChromBPNet: bias factorized, base-resolution deep learning models of chromatin accessibility reveal cis-regulatory sequence syntax, transcription factor footprints and regulatory variants
Source: bioRxiv. 2025 Jan 8:2024.12.25.630221. Preprint. [Version 2] doi: 10.1101/2024.12.25.630221 (PMC11741299; doi:10.1101/2024.12.25.630221)
Supplement: Supplement 4 [file media-4.zip › supplementary_files_3/k562_ATAC_bpnet_bias_model/k562_ATAC_raw_bpnet_bias_fold4_profile_modisco.pdf]

pattern	num_seqlets	cwm_fwd	cwm_rev	TOMTOM_match	TOMTOM_qval	TOMTOM_match_logo
pos_patterns.pattern_0	8647			TN5_1	4.637900e-08	
pos_patterns.pattern_1	8511			TN5_8	2.637640e-02	
pos_patterns.pattern_2	4263			TN5_1	5.456290e-07	
pos_patterns.pattern_3	2571			TN5_2	1.712550e-18	
pos_patterns.pattern_4	989			TN5_1	3.126240e-02	
pos_patterns.pattern_5	894			TN5_3	2.745060e-13	
pos_patterns.pattern_6	769			TN5_3	2.952300e-07	
pos_patterns.pattern_7	737			TN5_3	1.392260e-05	
pos_patterns.pattern_8	679			TN5_6	7.419670e-15	
pos_patterns.pattern_9	488			TN5_7	1.228760e-04	
pos_patterns.pattern_10	463			TN5_3	5.550970e-06	
pos_patterns.pattern_11	324			TN5_1	4.269920e-04	
pos_patterns.pattern_12	226			ZNF384_MA1125.1	4.853100e-02	
pos_patterns.pattern_13	174			TN5_4	5.679640e-02	
pos_patterns.pattern_14	68			TN5_8	7.577830e-06	
pos_patterns.pattern_15	55			ZNF384_MA1125.1	1.971230e-01	
pos_patterns.pattern_16	52			TN5_7	4.432020e-04	
pos_patterns.pattern_17	51			TN5_1	1.654870e-03	
pos_patterns.pattern_18	21			TN5_8	6.126630e-03	
