# Supplementary material for: ChromBPNet: bias factorized, base-resolution deep learning models of chromatin accessibility reveal cis-regulatory sequence syntax, transcription factor footprints and regulatory variants
Source: bioRxiv. 2025 Jan 8:2024.12.25.630221. Preprint. [Version 2] doi: 10.1101/2024.12.25.630221 (PMC11741299; doi:10.1101/2024.12.25.630221)
Supplement: Supplement 4 [file media-4.zip › supplementary_files_3/k562_ATAC_bpnet_bias_model/k562_ATAC_raw_bpnet_bias_fold2_profile_modisco.pdf]

pattern	num_seqlets	cwm_fwd	cwm_rev	TOMTOM_match	TOMTOM_qval	TOMTOM_match_logo
pos_patterns.pattern_0	8012			TN5_1	9.469040e-09	
pos_patterns.pattern_1	5392			TN5_4	2.114050e-02	
pos_patterns.pattern_2	4319			TN5_1	7.896670e-08	
pos_patterns.pattern_3	2950			TN5_2	1.235040e-30	
pos_patterns.pattern_4	2641			TN5_3	1.274990e-01	
pos_patterns.pattern_5	994			TN5_6	4.125130e-19	
pos_patterns.pattern_6	938			TN5_1	4.500240e-02	
pos_patterns.pattern_7	815			TN5_3	1.840150e-14	
pos_patterns.pattern_8	775			TN5_3	1.889700e-08	
pos_patterns.pattern_9	756			TN5_3	7.552660e-07	
pos_patterns.pattern_10	496			TN5_3	3.811370e-04	
pos_patterns.pattern_11	484			TN5_7	1.254610e-06	
pos_patterns.pattern_12	398			TN5_4	2.208020e-05	
pos_patterns.pattern_13	386			TN5_3	8.902310e-04	
pos_patterns.pattern_14	321			TN5_4	6.381050e-01	
pos_patterns.pattern_15	190			ZNF384_MA1125.1	6.461390e-02	
pos_patterns.pattern_16	35			FOXB1_forkhead_2	8.769330e-01	
pos_patterns.pattern_17	34			TN5_3	2.496710e-05	
pos_patterns.pattern_18	30			ZNF384_MA1125.1	9.537570e-02	
