# Supplementary material for: ChromBPNet: bias factorized, base-resolution deep learning models of chromatin accessibility reveal cis-regulatory sequence syntax, transcription factor footprints and regulatory variants
Source: bioRxiv. 2025 Jan 8:2024.12.25.630221. Preprint. [Version 2] doi: 10.1101/2024.12.25.630221 (PMC11741299; doi:10.1101/2024.12.25.630221)

pattern	num_seqlets	cwm_fwd	cwm_rev	TOMTOM_match	TOMTOM_qval	TOMTOM_match_logo
pos_patterns.pattern_0	10471			NaN	NaN	
pos_patterns.pattern_1	2449			ZFX_MOUSE.H11MO.0.B	1.862750e-02	
pos_patterns.pattern_2	1351			NR2F1_nuclearreceptor_3	5.930350e-01	
pos_patterns.pattern_3	813			NaN	NaN	
pos_patterns.pattern_4	638			ZN436_HUMAN.H11MO.0.C	1.000000e+00	
pos_patterns.pattern_5	531			DNASE_4	1.000000e+00	
pos_patterns.pattern_6	417			RUNX2_HUMAN.H11MO.0.A	2.899030e-01	
pos_patterns.pattern_7	353			ZN708_HUMAN.H11MO.0.C	8.424740e-01	
pos_patterns.pattern_8	314			RUNX3_MA0684.1	2.569040e-01	
pos_patterns.pattern_9	298			LHX6_MOUSE.H11MO.0.C	9.999880e-01	
pos_patterns.pattern_10	288			ZNF384_MA1125.1	3.859580e-02	
pos_patterns.pattern_11	244			MAZ_HUMAN.H11MO.0.A	4.578520e-09	
pos_patterns.pattern_12	183			RUNX2_HUMAN.H11MO.0.A	1.896700e-01	
pos_patterns.pattern_13	183			ZBTB6_HUMAN.H11MO.0.C	4.113100e-02	
pos_patterns.pattern_14	168			KLF15_HUMAN.H11MO.0.A	1.795350e-01	
pos_patterns.pattern_15	165			RREB1_MA0073.1	2.428990e-01	
pos_patterns.pattern_16	138			OSR2_HUMAN.H11MO.0.C	1.003230e-01	
pos_patterns.pattern_17	95			ZBT17_MOUSE.H11MO.0.A	9.470300e-03	
neg_patterns.pattern_0	1352			SP3_HUMAN.H11MO.0.B	3.699310e-01	
neg_patterns.pattern_1	56			SP2_HUMAN.H11MO.0.A	1.945760e-03	

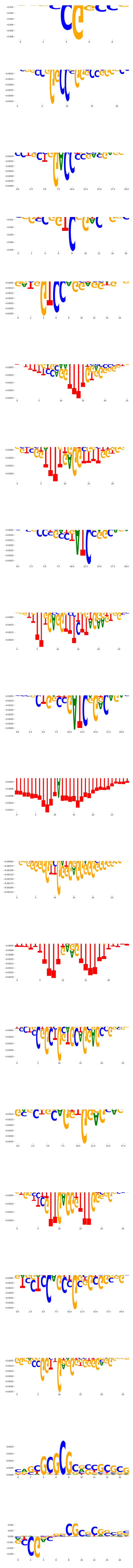

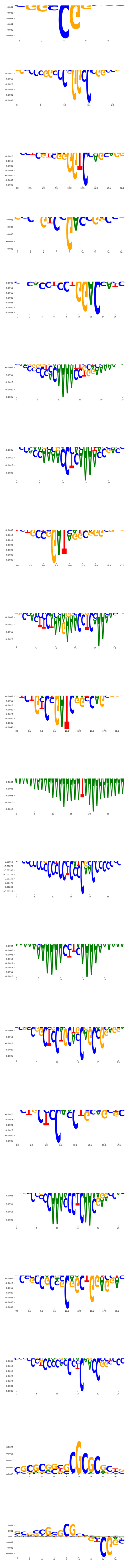

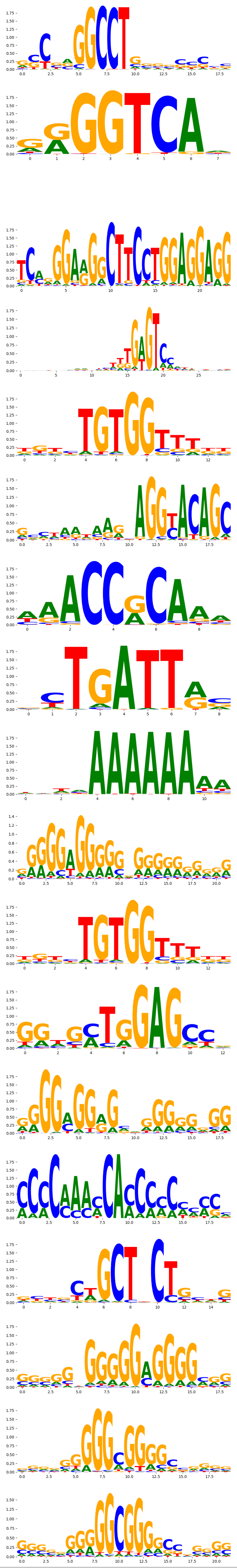

Supplement: Supplement 4 [file media-4.zip › supplementary_files_3/k562_ATAC_bpnet_bias_model/k562_ATAC_raw_bpnet_bias_fold4_counts_modisco.pdf]
