# Supplementary material for: ChromBPNet: bias factorized, base-resolution deep learning models of chromatin accessibility reveal cis-regulatory sequence syntax, transcription factor footprints and regulatory variants
Source: bioRxiv. 2025 Jan 8:2024.12.25.630221. Preprint. [Version 2] doi: 10.1101/2024.12.25.630221 (PMC11741299; doi:10.1101/2024.12.25.630221)
Supplement: Supplement 4 [file media-4.zip › supplementary_files_3/k562_ATAC_bpnet_bias_model/k562_ATAC_raw_bpnet_bias_fold3_profile_modisco.pdf]

pattern	num_seqlets	cwm_fwd	cwm_rev	TOMTOM_match	TOMTOM_qval	TOMTOM_match_logo
pos_patterns.pattern_0	8950			TN5_4	2.675730e-02	
pos_patterns.pattern_1	8338			TN5_1	3.927120e-10	
pos_patterns.pattern_2	4280			TN5_1	6.434130e-07	
pos_patterns.pattern_3	2223			TN5_2	3.441800e-17	
pos_patterns.pattern_4	1042			TN5_7	3.651520e-02	
pos_patterns.pattern_5	991			TN5_3	1.173050e-09	
pos_patterns.pattern_6	877			TN5_3	2.156950e-09	
pos_patterns.pattern_7	800			TN5_3	1.574800e-04	
pos_patterns.pattern_8	773			TN5_6	2.033860e-20	
pos_patterns.pattern_9	502			TN5_3	3.141760e-05	
pos_patterns.pattern_10	363			TN5_3	7.863180e-06	
pos_patterns.pattern_11	343			TN5_3	1.842520e-03	
pos_patterns.pattern_12	318			TN5_7	5.053040e-08	
pos_patterns.pattern_13	279			PRDM6_HUMAN.H11MO.0.C	6.748890e-02	
pos_patterns.pattern_14	69			TN5_4	4.792600e-01	
pos_patterns.pattern_15	28			ZNF384_MA1125.1	2.338480e-02	
