# Supplementary material for: ChromBPNet: bias factorized, base-resolution deep learning models of chromatin accessibility reveal cis-regulatory sequence syntax, transcription factor footprints and regulatory variants
Source: bioRxiv. 2025 Jan 8:2024.12.25.630221. Preprint. [Version 2] doi: 10.1101/2024.12.25.630221 (PMC11741299; doi:10.1101/2024.12.25.630221)
Supplement: Supplement 4 [file media-4.zip › supplementary_files_3/k562_ATAC_bpnet_bias_model/k562_ATAC_raw_bpnet_bias_fold0_profile_modisco.pdf]

pattern	num_seqlets	cwm_fwd	cwm_rev	TOMTOM_match	TOMTOM_qval	TOMTOM_match_logo
pos_patterns.pattern_0	16897			TN5_1	3.311610e-26	
pos_patterns.pattern_1	4237			TN5_2	0.000000e+00	
pos_patterns.pattern_2	3666			TN5_1	1.592680e-06	
pos_patterns.pattern_3	3334			TN5_3	1.435220e-29	
pos_patterns.pattern_4	3274			TN5_4	3.465330e-31	
pos_patterns.pattern_5	1997			TN5_6	2.943660e-23	
pos_patterns.pattern_6	1624			TN5_7	3.107550e-35	
pos_patterns.pattern_7	1053			TN5_8	2.458440e-41	
pos_patterns.pattern_8	925			TN5_8	2.250910e-06	
pos_patterns.pattern_9	551			TN5_4	1.000000e+00	
pos_patterns.pattern_10	470			PRDM6_HUMAN.H11MO.0.C	1.122440e-01	
pos_patterns.pattern_11	233			TN5_6	9.400980e-06	
pos_patterns.pattern_12	187			ZNF384_MA1125.1	1.718190e-02	
pos_patterns.pattern_13	172			ZNF384_MA1125.1	3.074120e-02	
pos_patterns.pattern_14	89			RREB1_MA0073.1	1.000000e+00	
pos_patterns.pattern_15	82			EGR2_HUMAN.H11MO.0.A	1.000000e+00	
pos_patterns.pattern_16	34			TEAD2_MOUSE.H11MO.0.C	9.812700e-01	
pos_patterns.pattern_17	32			TN5_7	2.705120e-01	
pos_patterns.pattern_18	29			NaN	NaN	
pos_patterns.pattern_19	22			TN5_3	1.000000e+00	
