# Supplementary material for: ChromBPNet: bias factorized, base-resolution deep learning models of chromatin accessibility reveal cis-regulatory sequence syntax, transcription factor footprints and regulatory variants
Source: bioRxiv. 2025 Jan 8:2024.12.25.630221. Preprint. [Version 2] doi: 10.1101/2024.12.25.630221 (PMC11741299; doi:10.1101/2024.12.25.630221)
Supplement: Supplement 4 [file media-4.zip › supplementary_files_3/k562_ATAC_bpnet_bias_model/k562_ATAC_raw_bpnet_bias_fold1_profile_modisco.pdf]

pattern	num_seqlets	cwm_fwd	cwm_rev	TOMTOM_match	TOMTOM_qval	TOMTOM_match_logo
pos_patterns.pattern_0	13018	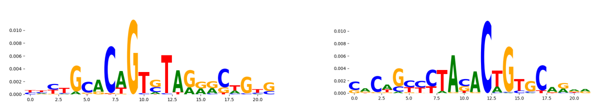		TN5_2	8.201700e-04	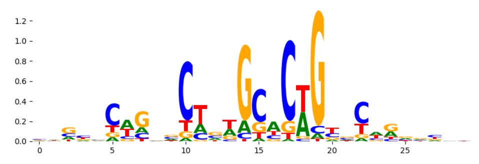
pos_patterns.pattern_1	7535	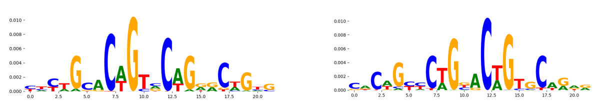		TN5_1	6.700460e-10	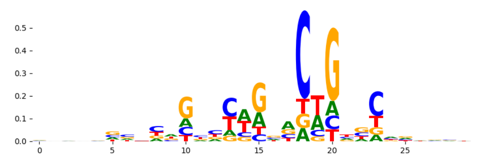
pos_patterns.pattern_2	2572	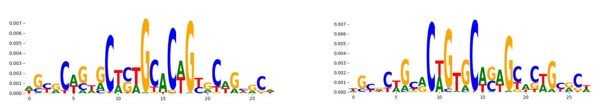		TN5_2	1.089560e-20	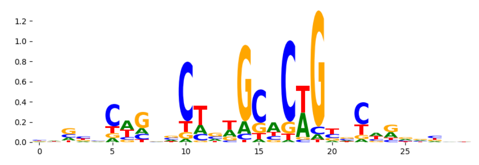
pos_patterns.pattern_3	2294	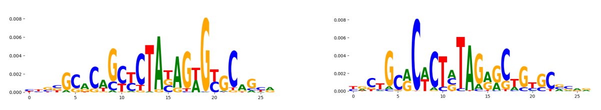		TN5_3	5.824520e-09	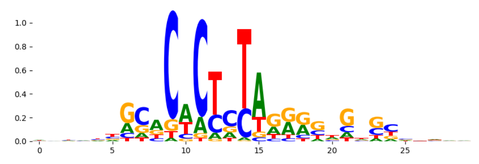
pos_patterns.pattern_4	1032	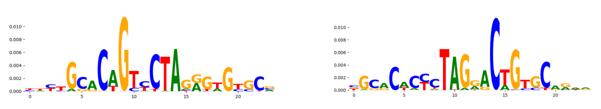		TN5_3	4.443830e-04	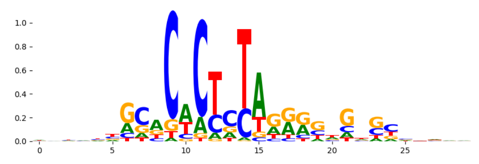
pos_patterns.pattern_5	1029	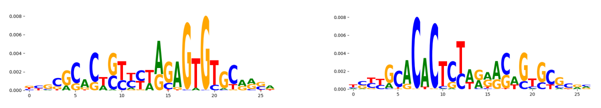		TN5_3	5.368410e-11	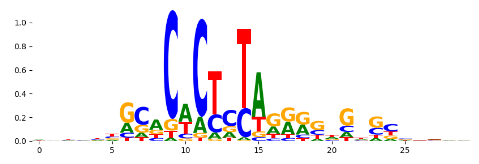
pos_patterns.pattern_6	742	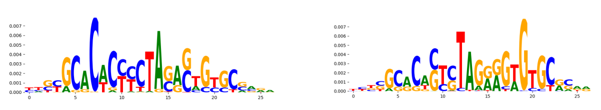		TN5_3	7.226360e-14	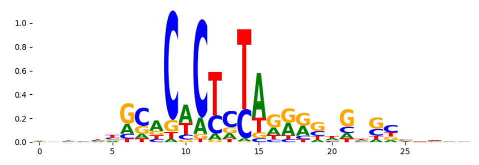
pos_patterns.pattern_7	695	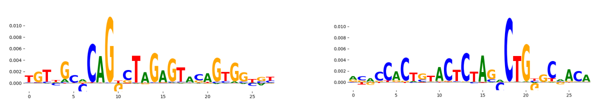		TN5_6	1.512940e-17	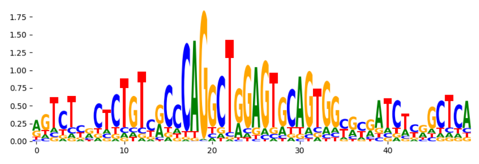
pos_patterns.pattern_8	374	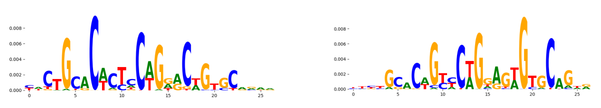		TN5_1	3.391360e-08	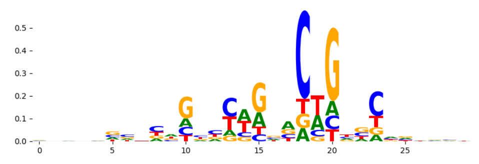
pos_patterns.pattern_9	371	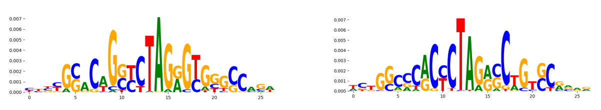		TN5_3	6.608430e-04	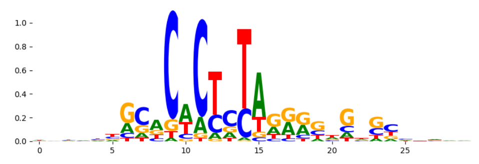
pos_patterns.pattern_10	104	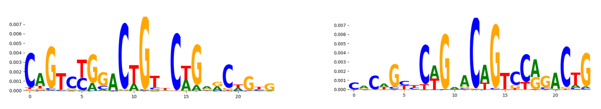		TN5_8	1.819300e-06	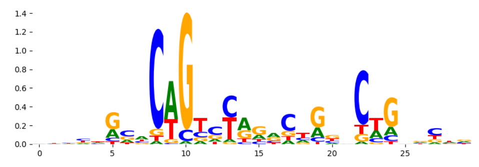
pos_patterns.pattern_11	100	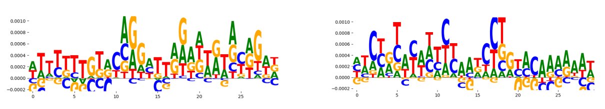		TN5_6	1.213600e-02	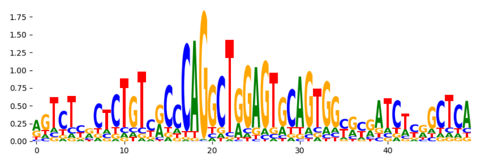
pos_patterns.pattern_12	39	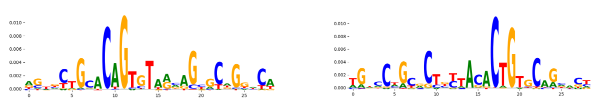		TN5_7	7.886330e-04	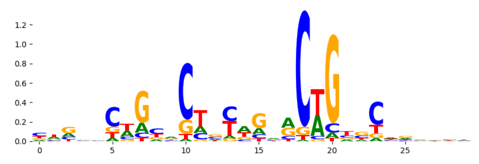
pos_patterns.pattern_13	20	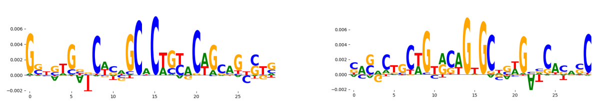		TN5_6	4.468860e-05	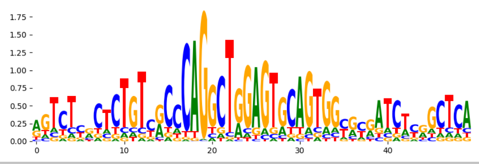
