# Supplementary material for: ChromBPNet: bias factorized, base-resolution deep learning models of chromatin accessibility reveal cis-regulatory sequence syntax, transcription factor footprints and regulatory variants
Source: bioRxiv. 2025 Jan 8:2024.12.25.630221. Preprint. [Version 2] doi: 10.1101/2024.12.25.630221 (PMC11741299; doi:10.1101/2024.12.25.630221)
Supplement: Supplement 4 [file media-4.zip › supplementary_files_3/imr90_ATAC_bpnet_bias_model/imr90_ATAC_raw_bpnet_bias_fold3_counts_modisco.pdf]

pattern	num_seqlets	cwm_fwd	cwm_rev	TOMTOM_match	TOMTOM_qval	TOMTOM_match_logo
pos_patterns.pattern_0	5360			ZNF384_MA1125.1	2.032130e-02	
pos_patterns.pattern_1	3680			NaN	NaN	
pos_patterns.pattern_2	3304			CUX2_CUT_1	2.000880e-01	
pos_patterns.pattern_3	2481			ZNF384_MA1125.1	2.314530e-02	
pos_patterns.pattern_4	1437			HNF6_HUMAN.H11MO.0.B	1.088070e-01	
pos_patterns.pattern_5	1273			DNASE_2	1.070430e-02	
pos_patterns.pattern_6	731			ANDR_HUMAN.H11MO.0.A	3.917290e-02	
pos_patterns.pattern_7	631			SP2_HUMAN.H11MO.0.A	6.045480e-03	
pos_patterns.pattern_8	495			SP2_HUMAN.H11MO.0.A	2.477800e-02	
pos_patterns.pattern_9	463			ZNF384_MA1125.1	1.283060e-03	
pos_patterns.pattern_10	277			PBX3_MOUSE.H11MO.0.A	5.018230e-01	
pos_patterns.pattern_11	231			ZNF384_MA1125.1	6.055020e-01	
pos_patterns.pattern_12	214			SP3_HUMAN.H11MO.0.B	8.195580e-03	
pos_patterns.pattern_13	152			SP2_HUMAN.H11MO.0.A	1.597500e-06	
pos_patterns.pattern_14	150			DNASE_2	4.608560e-01	
pos_patterns.pattern_15	135			ANDR_HUMAN.H11MO.0.A	4.902010e-03	
pos_patterns.pattern_16	116			AP2B_HUMAN.H11MO.0.B	1.213230e-01	
pos_patterns.pattern_17	107			INSM1_MA0155.1	1.104890e-01	
pos_patterns.pattern_18	94			PDX1_HUMAN.H11MO.0.A	2.116730e-01	
pos_patterns.pattern_19	71			FOXL1_forkhead_2	1.000000e+00	
pos_patterns.pattern_20	69			NFAC1_HUMAN.H11MO.0.B	3.373280e-01	
pos_patterns.pattern_21	46			NFAC2_HUMAN.H11MO.0.B	2.725480e-01	
pos_patterns.pattern_22	36			TBX2_TBX_1	4.212570e-02	
pos_patterns.pattern_23	34			ZN563_HUMAN.H11MO.0.C	1.000000e+00	
pos_patterns.pattern_24	25			MESP1_bHLH_1	1.000000e+00	
pos_patterns.pattern_25	21			Foxj3.mouse_forkhead_2	8.732700e-01	
neg_patterns.pattern_0	2840			TN5_4	1.948740e-06	
neg_patterns.pattern_1	2695			TN5_8	9.045920e-01	
neg_patterns.pattern_2	1545			SP5_MOUSE.H11MO.0.C	3.253620e-05	
neg_patterns.pattern_3	951			TN5_1	3.702540e-03	

pattern	num_seqlets	cwm_fwd	cwm_rev	TOMTOM_match	TOMTOM_qval	TOMTOM_match_logo
neg_patterns.pattern_4	769	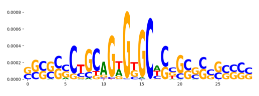	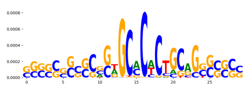	TN5_2	1.276540e-01	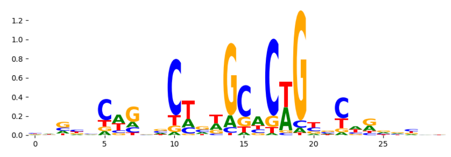
neg_patterns.pattern_5	439	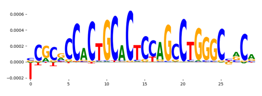	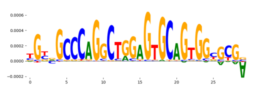	TN5_6	2.175850e-20	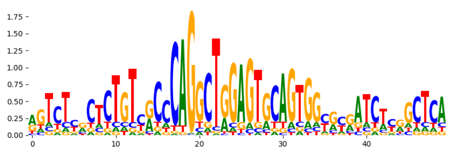
neg_patterns.pattern_6	302	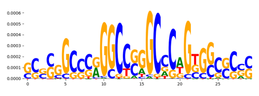	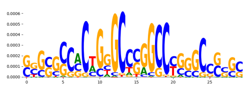	FOS_HUMAN.H11MO.0.A	1.000000e+00	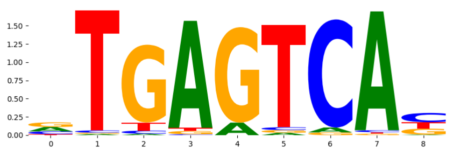
neg_patterns.pattern_7	301	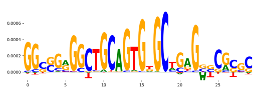	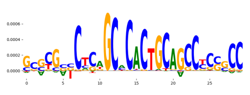	RARG_HUMAN.H11MO.0.B	1.000000e+00	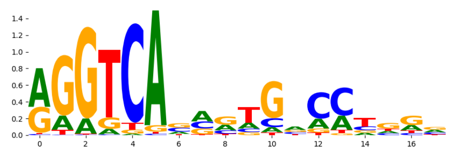
neg_patterns.pattern_8	259	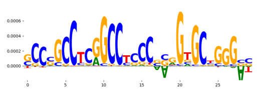	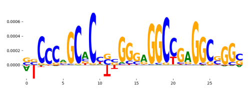	ZN770_HUMAN.H11MO.0.C	4.583540e-02	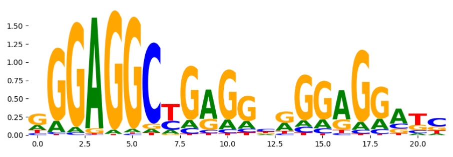
neg_patterns.pattern_9	247	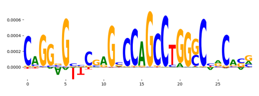	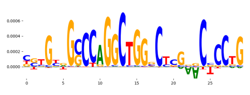	DNASE_5	8.250310e-13	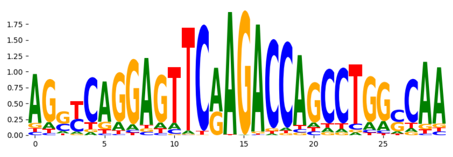
neg_patterns.pattern_10	223	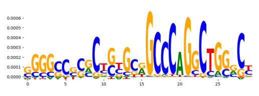	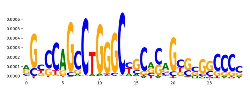	ZN121_HUMAN.H11MO.0.C	1.487870e-08	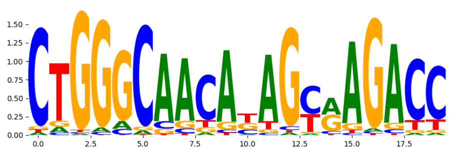
neg_patterns.pattern_11	212	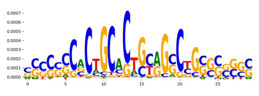	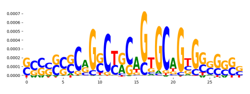	TN5_6	5.425760e-02	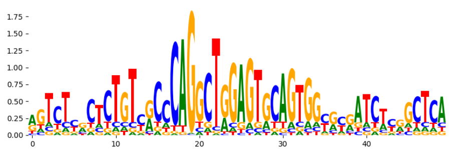
neg_patterns.pattern_12	134	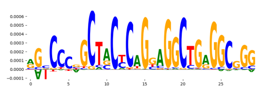	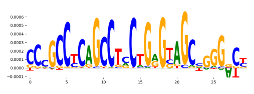	ZN770_HUMAN.H11MO.0.C	3.730220e-01	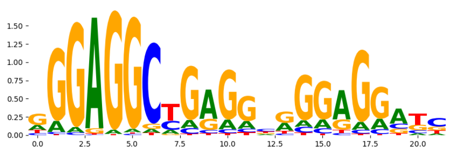
neg_patterns.pattern_13	65	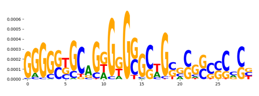	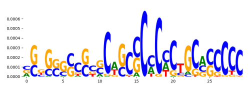	ZN341_HUMAN.H11MO.0.C	2.211780e-01	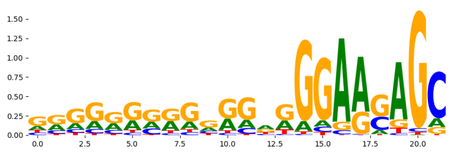
neg_patterns.pattern_14	52	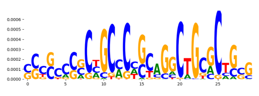	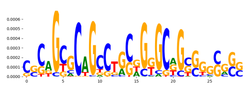	CTCF_MA0139.1	6.181050e-01	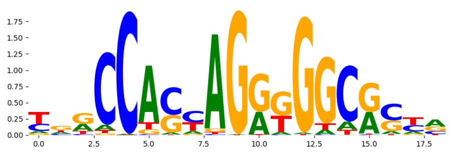
neg_patterns.pattern_15	38	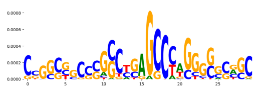	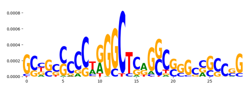	TN5_1	8.383400e-01	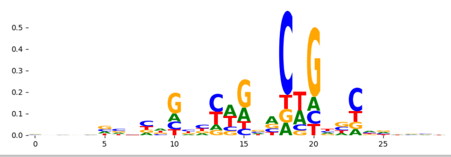
