# Supplementary material for: ChromBPNet: bias factorized, base-resolution deep learning models of chromatin accessibility reveal cis-regulatory sequence syntax, transcription factor footprints and regulatory variants
Source: bioRxiv. 2025 Jan 8:2024.12.25.630221. Preprint. [Version 2] doi: 10.1101/2024.12.25.630221 (PMC11741299; doi:10.1101/2024.12.25.630221)
Supplement: Supplement 4 [file media-4.zip › supplementary_files_3/imr90_ATAC_bpnet_bias_model/imr90_ATAC_raw_bpnet_bias_fold0_counts_modisco.pdf]

pattern	num_seqlets	cwm_fwd	cwm_rev	TOMTOM_match	TOMTOM_qval	TOMTOM_match_logo
pos_patterns.pattern_0	4606			SP2_HUMAN.H11MO.0.A	2.553280e-06	
pos_patterns.pattern_1	3585			ZNF384_MA1125.1	5.533850e-02	
pos_patterns.pattern_2	3245			ZNF384_MA1125.1	1.415450e-04	
pos_patterns.pattern_3	3192			ZNF384_MA1125.1	6.577530e-02	
pos_patterns.pattern_4	2881			ZNF384_MA1125.1	1.269090e-02	
pos_patterns.pattern_5	2495			DNASE_2	8.046790e-01	
pos_patterns.pattern_6	1992			HXB7_HUMAN.H11MO.0.C	1.993840e-02	
pos_patterns.pattern_7	1726			ZNF384_MA1125.1	3.657430e-02	
pos_patterns.pattern_8	1225			PBX1_MA0070.1	1.000000e+00	
pos_patterns.pattern_9	824			ONECUT3_CUT_1	3.017140e-01	
pos_patterns.pattern_10	520			FLI1_ETS_4	8.544260e-01	
pos_patterns.pattern_11	430			ZBT7A_HUMAN.H11MO.0.A	4.922340e-02	
pos_patterns.pattern_12	427			FOXO3_forkhead_3	1.000000e+00	
pos_patterns.pattern_13	129			ZBT7A_HUMAN.H11MO.0.A	4.892730e-01	
pos_patterns.pattern_14	128			DNASE_3	5.311230e-01	
pos_patterns.pattern_15	81			ZSCAN4_C2H2_1	1.000000e+00	
pos_patterns.pattern_16	112			NaN	NaN	
pos_patterns.pattern_17	73			ZBT7A_HUMAN.H11MO.0.A	3.800490e-02	
pos_patterns.pattern_18	45			SP1_HUMAN.H11MO.0.A	5.224730e-01	
pos_patterns.pattern_19	25			ZN274_HUMAN.H11MO.0.A	1.000000e+00	
neg_patterns.pattern_0	6245			TN5_7	2.294010e-04	
neg_patterns.pattern_1	4664			TN5_2	3.166030e-06	
neg_patterns.pattern_2	2904			TN5_6	0.000000e+00	
neg_patterns.pattern_3	2847			ZN467_HUMAN.H11MO.0.C	5.218860e-08	
neg_patterns.pattern_4	1883			P53_MOUSE.H11MO.0.A	4.170980e-02	
neg_patterns.pattern_5	1666			DNASE_5	2.866080e-04	
neg_patterns.pattern_6	1525			TBX1_TBX_1	9.801400e-01	
neg_patterns.pattern_7	1469			ZN770_HUMAN.H11MO.0.C	7.061130e-08	
neg_patterns.pattern_8	1259			BARX1_MOUSE.H11MO.0.C	4.074490e-01	
neg_patterns.pattern_9	1027			TN5_6	1.549250e-01	

pattern	num_seqlets	cwm_fwd	cwm_rev	TOMTOM_match	TOMTOM_qval	TOMTOM_match_logo
neg_patterns.pattern_10	840	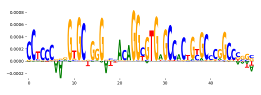	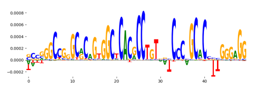	IKZF1_HUMAN.H11MO.0.C	6.237710e-01	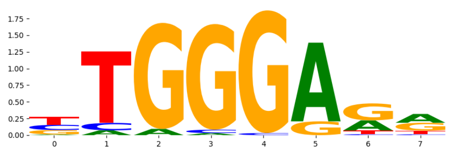
neg_patterns.pattern_11	694	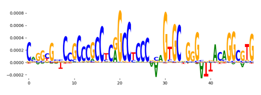	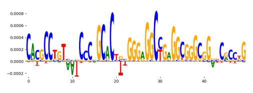	ZN770_HUMAN.H11MO.0.C	1.379390e-06	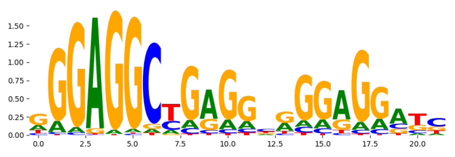
neg_patterns.pattern_12	669	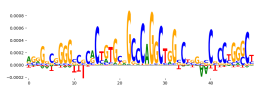	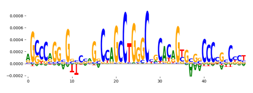	DNASE_5	3.223360e-13	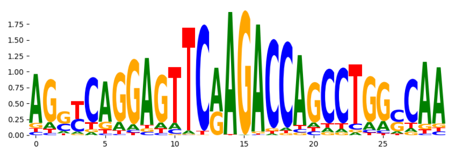
neg_patterns.pattern_13	400	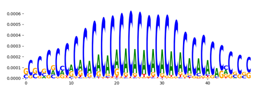	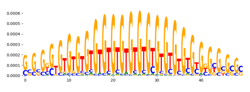	NaN	NaN	
neg_patterns.pattern_14	398	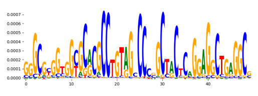	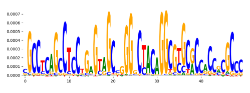	ITF2_HUMAN.H11MO.0.C	1.000000e+00	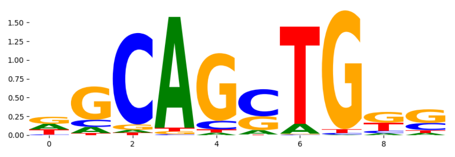
neg_patterns.pattern_15	349	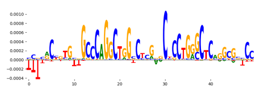	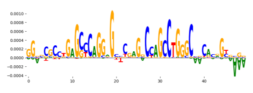	DNASE_5	9.603220e-17	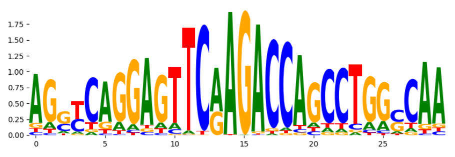
neg_patterns.pattern_16	270	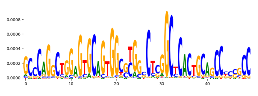	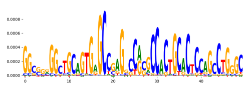	TN5_6	5.215680e-10	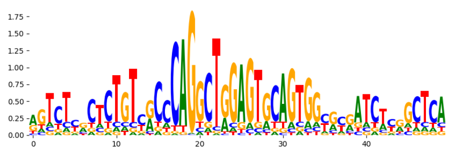
neg_patterns.pattern_17	171	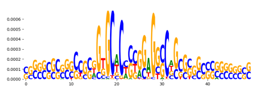	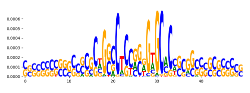	CTCF_MOUSE.H11MO.0.A	1.014050e-01	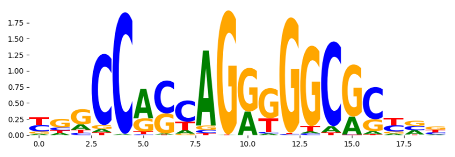
neg_patterns.pattern_18	155	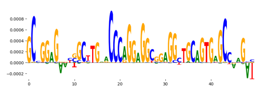	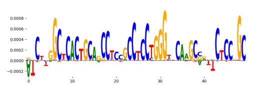	VDR_MA0693.2	1.000000e+00	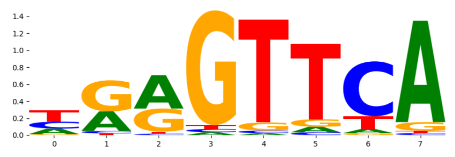
neg_patterns.pattern_19	60	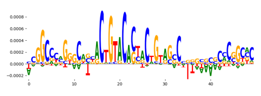	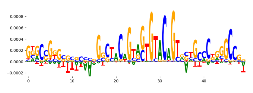	TN5_4	1.000000e+00	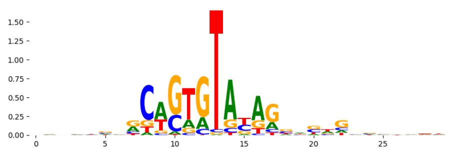
neg_patterns.pattern_20	42	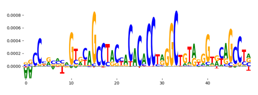	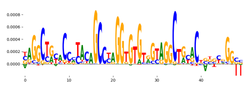	TBX20_MA0689.1	4.554620e-01	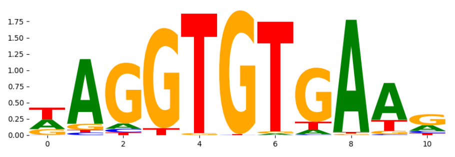
neg_patterns.pattern_21	36	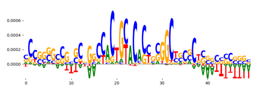	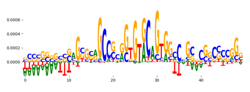	ZNF232_C2H2_1	1.000000e+00	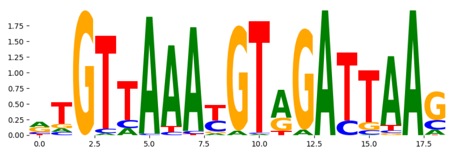
neg_patterns.pattern_22	24	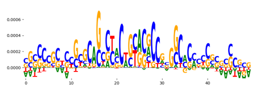	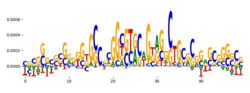	Ar.mouse_nuclearreceptor_1	1.000000e+00	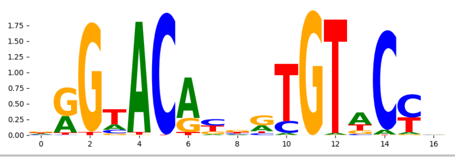
