# Supplementary material for: ChromBPNet: bias factorized, base-resolution deep learning models of chromatin accessibility reveal cis-regulatory sequence syntax, transcription factor footprints and regulatory variants
Source: bioRxiv. 2025 Jan 8:2024.12.25.630221. Preprint. [Version 2] doi: 10.1101/2024.12.25.630221 (PMC11741299; doi:10.1101/2024.12.25.630221)

pattern	num_seqlets	cwm_fwd	cwm_rev	TOMTOM_match	TOMTOM_qval	TOMTOM_match_logo
pos_patterns.pattern_0	9518			TN5_1	2.045570e-08	
pos_patterns.pattern_1	5938			TN5_4	2.607330e-02	
pos_patterns.pattern_2	4189			TN5_1	3.137040e-06	
pos_patterns.pattern_3	3125			TN5_3	1.022420e-03	
pos_patterns.pattern_4	3122			KLF4_MA0039.3	7.096000e-01	
pos_patterns.pattern_5	2641			TN5_3	1.260580e-12	
pos_patterns.pattern_6	718			TN5_3	9.027640e-03	
pos_patterns.pattern_7	603			TN5_3	7.588830e-09	
pos_patterns.pattern_8	591			TN5_3	2.416740e-13	
pos_patterns.pattern_9	400			TN5_3	6.377630e-07	
pos_patterns.pattern_10	296			PRDM6_HUMAN.H11MO.0.C	6.468200e-02	
pos_patterns.pattern_11	206			RREB1_MA0073.1	1.000000e+00	
pos_patterns.pattern_12	132			FOXA2_HUMAN.H11MO.0.A	9.928580e-01	
pos_patterns.pattern_13	92			HOXC13_homeodomain_1	6.092230e-01	
pos_patterns.pattern_14	82			ZN121_HUMAN.H11MO.0.C	3.194300e-04	
pos_patterns.pattern_15	64			ZN121_HUMAN.H11MO.0.C	2.965610e-05	
pos_patterns.pattern_16	46			ZNF384_MA1125.1	2.493950e-02	
pos_patterns.pattern_17	20			TN5_3	3.860090e-02	

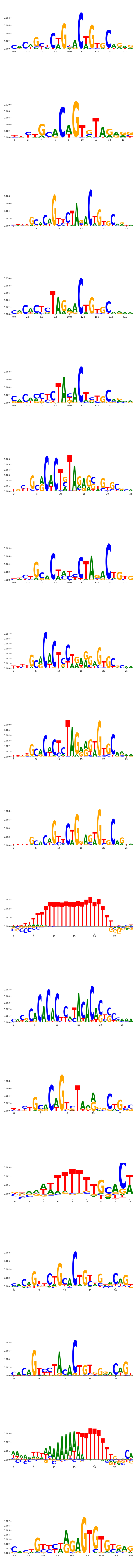

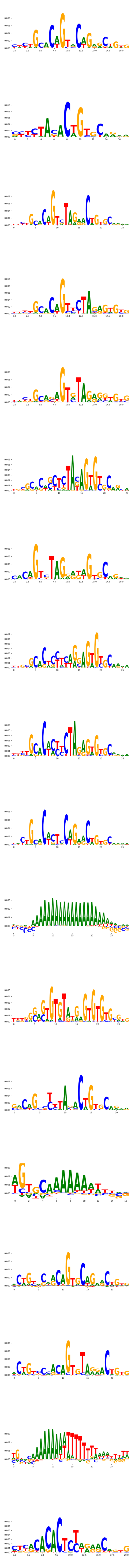

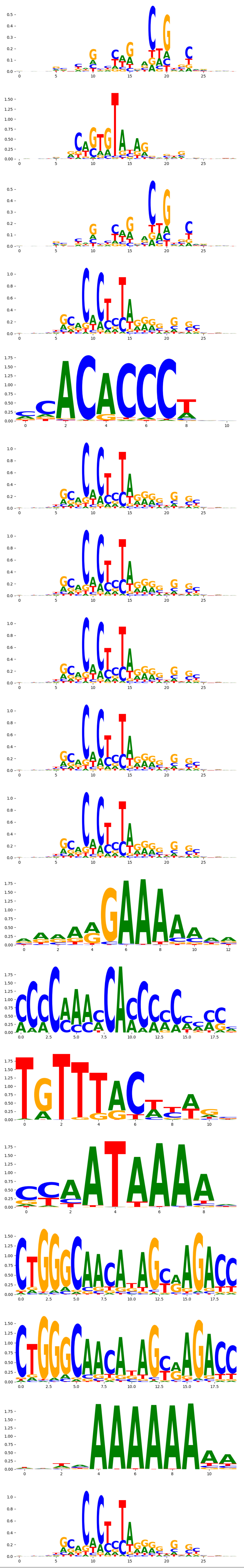

Supplement: Supplement 4 [file media-4.zip › supplementary_files_3/imr90_ATAC_bpnet_bias_model/imr90_ATAC_raw_bpnet_bias_fold1_profile_modisco.pdf]
