# Supplementary material for: ChromBPNet: bias factorized, base-resolution deep learning models of chromatin accessibility reveal cis-regulatory sequence syntax, transcription factor footprints and regulatory variants
Source: bioRxiv. 2025 Jan 8:2024.12.25.630221. Preprint. [Version 2] doi: 10.1101/2024.12.25.630221 (PMC11741299; doi:10.1101/2024.12.25.630221)
Supplement: Supplement 4 [file media-4.zip › supplementary_files_3/imr90_ATAC_bpnet_bias_model/imr90_ATAC_raw_bpnet_bias_fold0_profile_modisco.pdf]

pattern	num_seqlets	cwm_fwd	cwm_rev	TOMTOM_match	TOMTOM_qval	TOMTOM_match_logo
pos_patterns.pattern_0	12385			TN5_8	2.297410e-02	
pos_patterns.pattern_1	9636			TN5_1	4.496600e-10	
pos_patterns.pattern_2	4321			TN5_2	1.962610e-20	
pos_patterns.pattern_3	3255			TN5_3	3.241600e-07	
pos_patterns.pattern_4	2952			TN5_4	1.535820e-09	
pos_patterns.pattern_5	2722			TN5_1	4.538340e-04	
pos_patterns.pattern_6	1671			TN5_3	1.974290e-17	
pos_patterns.pattern_7	1647			TN5_4	1.316630e-03	
pos_patterns.pattern_8	1581			TN5_3	1.152750e-07	
pos_patterns.pattern_9	1140			TN5_7	1.126780e-12	
pos_patterns.pattern_10	884			TN5_8	3.596620e-13	
pos_patterns.pattern_11	645			ZNF384_MA1125.1	7.645990e-02	
pos_patterns.pattern_12	640			TN5_4	8.121710e-03	
pos_patterns.pattern_13	275			TN5_4	4.985440e-04	
pos_patterns.pattern_14	158			TN5_4	7.326300e-04	
pos_patterns.pattern_15	58			TCF4_bHLH_2	6.139190e-01	
pos_patterns.pattern_16	47			TBX20_MA0689.1	2.600740e-01	
pos_patterns.pattern_17	49			TP63_MA0525.2	6.005200e-01	
pos_patterns.pattern_18	31			OVOL1_HUMAN.H11MO.0.C	1.000000e+00	
