# Supplementary material for: ChromBPNet: bias factorized, base-resolution deep learning models of chromatin accessibility reveal cis-regulatory sequence syntax, transcription factor footprints and regulatory variants
Source: bioRxiv. 2025 Jan 8:2024.12.25.630221. Preprint. [Version 2] doi: 10.1101/2024.12.25.630221 (PMC11741299; doi:10.1101/2024.12.25.630221)
Supplement: Supplement 4 [file media-4.zip › supplementary_files_3/imr90_ATAC_bpnet_bias_model/imr90_ATAC_raw_bpnet_bias_fold3_profile_modisco.pdf]

pattern	num_seqlets	cwm_fwd	cwm_rev	TOMTOM_match	TOMTOM_qval	TOMTOM_match_logo
pos_patterns.pattern_0	9972	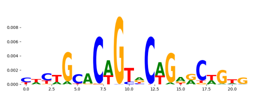	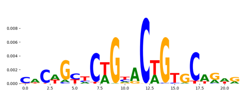	TN5_2	9.959580e-10	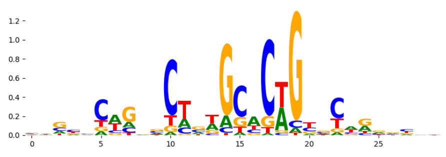
pos_patterns.pattern_1	6642	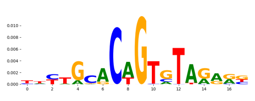	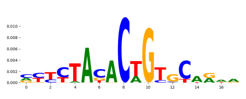	TN5_4	2.181650e-02	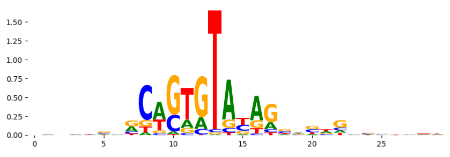
pos_patterns.pattern_2	4680	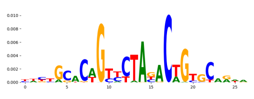	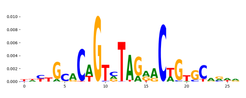	TN5_1	1.621050e-06	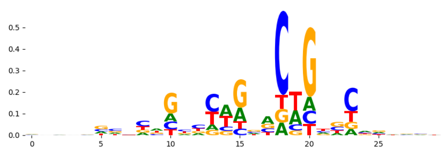
pos_patterns.pattern_3	3879	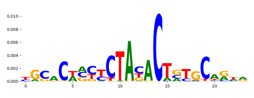	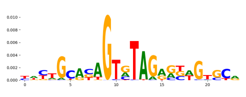	TN5_3	2.762170e-01	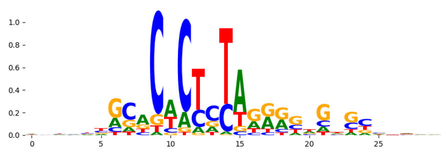
pos_patterns.pattern_4	2863	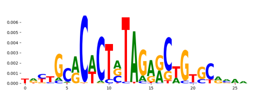	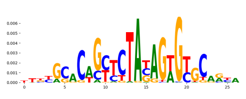	TN5_3	2.195470e-10	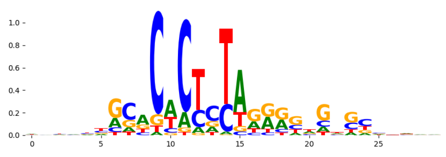
pos_patterns.pattern_5	1502	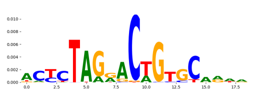	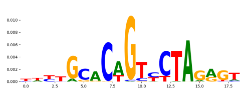	TN5_7	2.439630e-02	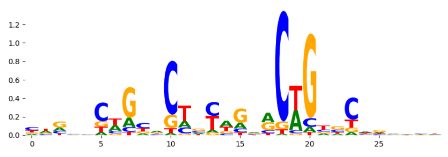
pos_patterns.pattern_6	898	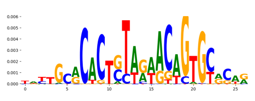	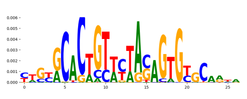	TN5_3	6.512600e-07	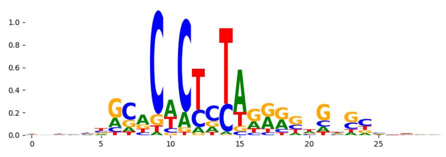
pos_patterns.pattern_7	689	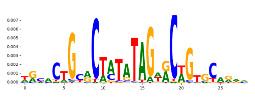	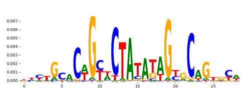	TN5_3	3.450650e-04	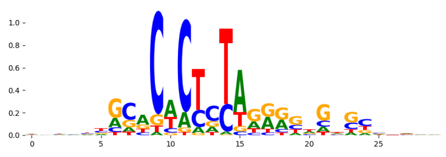
pos_patterns.pattern_8	331	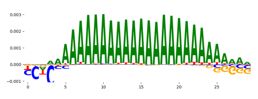	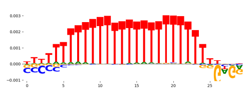	PRDM6_HUMAN.H11MO.0.C	7.555640e-02	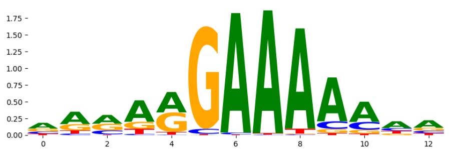
pos_patterns.pattern_9	142	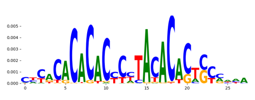	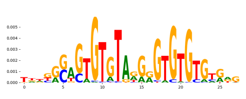	EGR2_HUMAN.H11MO.0.A	1.000000e+00	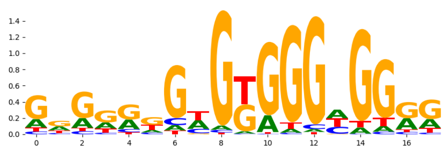
pos_patterns.pattern_10	92	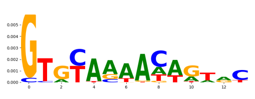	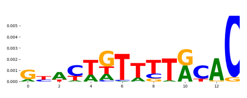	TBX1_TBX_1	5.990980e-01	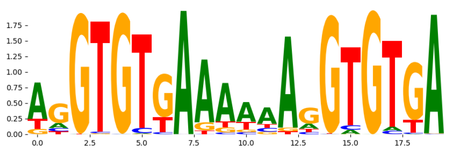
pos_patterns.pattern_11	23	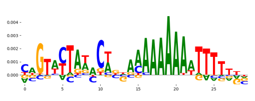	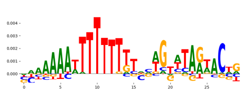	ZNF384_MA1125.1	4.135290e-02	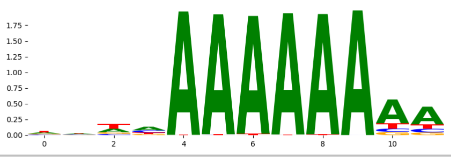
