# Supplementary material for: ChromBPNet: bias factorized, base-resolution deep learning models of chromatin accessibility reveal cis-regulatory sequence syntax, transcription factor footprints and regulatory variants
Source: bioRxiv. 2025 Jan 8:2024.12.25.630221. Preprint. [Version 2] doi: 10.1101/2024.12.25.630221 (PMC11741299; doi:10.1101/2024.12.25.630221)
Supplement: Supplement 4 [file media-4.zip › supplementary_files_3/imr90_ATAC_bpnet_bias_model/imr90_ATAC_raw_bpnet_bias_fold2_profile_modisco.pdf]

pattern	num_seqlets	cwm_fwd	cwm_rev	TOMTOM_match	TOMTOM_qval	TOMTOM_match_logo
pos_patterns.pattern_0	8154			TN5_1	3.495660e-08	
pos_patterns.pattern_1	5724			TN5_4	2.522990e-02	
pos_patterns.pattern_2	4624			TN5_1	7.306880e-05	
pos_patterns.pattern_3	3856			KLF4_MA0039.3	8.149190e-01	
pos_patterns.pattern_4	2580			TN5_2	1.341290e-07	
pos_patterns.pattern_5	1645			TN5_3	7.233370e-11	
pos_patterns.pattern_6	1619			TN5_1	4.388730e-02	
pos_patterns.pattern_7	698			TN5_3	7.475330e-05	
pos_patterns.pattern_8	684			TN5_3	1.464640e-05	
pos_patterns.pattern_9	579			TN5_3	1.796540e-05	
pos_patterns.pattern_10	383			PRDM6_HUMAN.H11MO.0.C	8.213510e-02	
pos_patterns.pattern_11	235			TN5_8	1.000000e+00	
pos_patterns.pattern_12	225			RREB1_MA0073.1	1.000000e+00	
pos_patterns.pattern_13	170			TN5_6	4.246270e-03	
pos_patterns.pattern_14	78			RORA_MA0072.1	1.000000e+00	
pos_patterns.pattern_15	64			TN5_3	1.175170e-02	
