# Supplementary material for: ChromBPNet: bias factorized, base-resolution deep learning models of chromatin accessibility reveal cis-regulatory sequence syntax, transcription factor footprints and regulatory variants
Source: bioRxiv. 2025 Jan 8:2024.12.25.630221. Preprint. [Version 2] doi: 10.1101/2024.12.25.630221 (PMC11741299; doi:10.1101/2024.12.25.630221)
Supplement: Supplement 4 [file media-4.zip › supplementary_files_3/imr90_ATAC_bpnet_bias_model/imr90_ATAC_raw_bpnet_bias_fold1_counts_modisco.pdf]

pattern	num_seqlets	cwm_fwd	cwm_rev	TOMTOM_match	TOMTOM_qval	TOMTOM_match_logo
pos_patterns.pattern_0	3832			ZNF384_MA1125.1	1.000000e+00	
pos_patterns.pattern_1	2085			ZNF384_MA1125.1	1.686180e-02	
pos_patterns.pattern_2	1909			ZNF384_MA1125.1	1.237820e-02	
pos_patterns.pattern_3	1604			ZNF384_MA1125.1	8.381310e-03	
pos_patterns.pattern_4	1484			ZNF384_MA1125.1	5.512490e-02	
pos_patterns.pattern_5	1294			SP3_HUMAN.H11MO.0.B	1.095760e-05	
pos_patterns.pattern_6	1165			ZNF384_MA1125.1	2.662260e-02	
pos_patterns.pattern_7	1119			SP1_HUMAN.H11MO.0.A	5.476220e-02	
pos_patterns.pattern_8	1116			ZNF384_MA1125.1	6.452350e-02	
pos_patterns.pattern_9	988			SP1_HUMAN.H11MO.0.A	2.510720e-02	
pos_patterns.pattern_10	886			SP1_HUMAN.H11MO.0.A	1.312890e-02	
pos_patterns.pattern_11	854			RUNX3_RUNX_2	5.595720e-01	
pos_patterns.pattern_12	637			ZBTB7A_C2H2_1	6.435600e-01	
pos_patterns.pattern_13	522			RELA_MA0107.1	6.012060e-01	
pos_patterns.pattern_14	397			ZFX_MOUSE.H11MO.0.B	7.219730e-05	
pos_patterns.pattern_15	327			SP1_HUMAN.H11MO.0.A	5.048380e-03	
pos_patterns.pattern_16	289			ZN281_MOUSE.H11MO.0.A	4.556590e-02	
pos_patterns.pattern_17	228			ZBT7A_HUMAN.H11MO.0.A	8.670540e-01	
pos_patterns.pattern_18	219			ZNF384_MA1125.1	1.155050e-02	
pos_patterns.pattern_19	126			ZIC3_C2H2_1	1.000000e+00	
pos_patterns.pattern_20	80			MXI1_HUMAN.H11MO.0.A	5.701390e-02	
pos_patterns.pattern_21	68			GLIS2_C2H2_1	8.487280e-01	
pos_patterns.pattern_22	34			CUX2_MOUSE.H11MO.0.C	8.024920e-02	
pos_patterns.pattern_23	26			THA11_MOUSE.H11MO.0.B	1.000000e+00	
pos_patterns.pattern_24	24			PRDM6_HUMAN.H11MO.0.C	8.004980e-02	
neg_patterns.pattern_0	4412			TN5_1	1.486920e-02	
neg_patterns.pattern_1	3899			TN5_8	3.239990e-01	
neg_patterns.pattern_2	3716			TN5_2	1.172430e-06	
neg_patterns.pattern_3	810			TN5_6	1.103230e-24	

pattern	num_seqlets	cwm_fwd	cwm_rev	TOMTOM_match	TOMTOM_qval	TOMTOM_match_logo
neg_patterns.pattern_4	433			RREB1_MA0073.1	1.000000e+00	
neg_patterns.pattern_5	396			ZNF18_HUMAN.H11MO.0.C	3.567590e-01	
neg_patterns.pattern_6	384			TN5_6	2.267270e-04	
neg_patterns.pattern_7	364			EGR1_MOUSE.H11MO.0.A	7.295390e-03	
neg_patterns.pattern_8	364			IKZF1_HUMAN.H11MO.0.C	9.132780e-01	
neg_patterns.pattern_9	363			TN5_2	2.505500e-03	
neg_patterns.pattern_10	310			TN5_4	2.262460e-02	
neg_patterns.pattern_11	272			MGA_MA0801.1	2.546510e-01	
neg_patterns.pattern_12	248			ZN121_HUMAN.H11MO.0.C	3.498450e-03	
neg_patterns.pattern_13	242			Hes1_MA1099.1	1.857480e-01	
neg_patterns.pattern_14	233			SMAD3_HUMAN.H11MO.0.B	4.282920e-01	
neg_patterns.pattern_15	208			TN5_6	7.863910e-03	
neg_patterns.pattern_16	202			TN5_2	4.674830e-02	
neg_patterns.pattern_17	194			TN5_6	6.511850e-05	
neg_patterns.pattern_18	160			ZSCAN4_C2H2_1	1.000000e+00	
neg_patterns.pattern_19	157			BRAC_MOUSE.H11MO.0.B	1.513440e-01	
neg_patterns.pattern_20	156			THA_HUMAN.H11MO.0.C	8.024990e-02	
neg_patterns.pattern_21	116			TN5_8	3.033490e-02	
neg_patterns.pattern_22	71			TN5_6	4.697640e-06	
neg_patterns.pattern_23	50			TN5_6	1.000000e+00	
neg_patterns.pattern_24	42			DNASE_5	1.609920e-04	
neg_patterns.pattern_25	24			HIC2_C2H2_1	8.095220e-01	
neg_patterns.pattern_26	21			KLF4_MA0039.3	1.164220e-01	
neg_patterns.pattern_27	20			SOX9_HMG_2	4.795950e-01	
