# Supplementary material for: ChromBPNet: bias factorized, base-resolution deep learning models of chromatin accessibility reveal cis-regulatory sequence syntax, transcription factor footprints and regulatory variants
Source: bioRxiv. 2025 Jan 8:2024.12.25.630221. Preprint. [Version 2] doi: 10.1101/2024.12.25.630221 (PMC11741299; doi:10.1101/2024.12.25.630221)
Supplement: Supplement 4 [file media-4.zip › supplementary_files_3/imr90_ATAC_bpnet_bias_model/imr90_ATAC_raw_bpnet_bias_fold4_profile_modisco.pdf]

pattern	num_seqlets	cwm_fwd	cwm_rev	TOMTOM_match	TOMTOM_qval	TOMTOM_match_logo
pos_patterns.pattern_0	9822			TN5_2	7.406830e-09	
pos_patterns.pattern_1	6242			TN5_4	2.454310e-02	
pos_patterns.pattern_2	4824			TN5_2	2.585750e-03	
pos_patterns.pattern_3	3650			TN5_3	2.659300e-01	
pos_patterns.pattern_4	2607			TN5_3	1.073280e-06	
pos_patterns.pattern_5	1762			TN5_3	1.207080e-03	
pos_patterns.pattern_6	1019			TN5_3	7.972970e-11	
pos_patterns.pattern_7	393			PRDM6_HUMAN.H11MO.0.C	7.185230e-02	
pos_patterns.pattern_8	212			FOXA2_HUMAN.H11MO.0.A	1.000000e+00	
pos_patterns.pattern_9	172			RREB1_MA0073.1	1.000000e+00	
pos_patterns.pattern_10	132			TN5_1	1.004180e-02	
pos_patterns.pattern_11	78			TN5_7	2.426110e-04	
pos_patterns.pattern_12	71			TN5_6	3.002750e-08	
pos_patterns.pattern_13	67			TN5_4	8.316990e-03	
pos_patterns.pattern_14	29			ZNF384_MA1125.1	2.043820e-02	
