# Supplementary material for: ChromBPNet: bias factorized, base-resolution deep learning models of chromatin accessibility reveal cis-regulatory sequence syntax, transcription factor footprints and regulatory variants
Source: bioRxiv. 2025 Jan 8:2024.12.25.630221. Preprint. [Version 2] doi: 10.1101/2024.12.25.630221 (PMC11741299; doi:10.1101/2024.12.25.630221)
Supplement: Supplement 4 [file media-4.zip › supplementary_files_3/imr90_ATAC_bpnet_bias_model/imr90_ATAC_raw_bpnet_bias_fold2_counts_modisco.pdf]

pattern	num_seqlets	cwm_fwd	cwm_rev	TOMTOM_match	TOMTOM_qval	TOMTOM_match_logo
pos_patterns.pattern_0	2930			ZNF384_MA1125.1	9.681010e-02	
pos_patterns.pattern_1	2920			ZNF384_MA1125.1	3.919540e-03	
pos_patterns.pattern_2	2908			ZNF384_MA1125.1	1.574520e-03	
pos_patterns.pattern_3	2902			FOXJ3_HUMAN.H11MO.0.A	3.902680e-02	
pos_patterns.pattern_4	2555			ZNF384_MA1125.1	7.314620e-02	
pos_patterns.pattern_5	2465			DNASE_2	2.578700e-01	
pos_patterns.pattern_6	1257			MAZ_HUMAN.H11MO.0.A	2.058930e-04	
pos_patterns.pattern_7	806			ZFX_MOUSE.H11MO.0.B	8.234770e-03	
pos_patterns.pattern_8	752			ZBT7A_HUMAN.H11MO.0.A	1.660590e-02	
pos_patterns.pattern_9	629			DNASE_2	1.675300e-01	
pos_patterns.pattern_10	572			ZNF384_MA1125.1	8.056040e-03	
pos_patterns.pattern_11	509			NFIX_NFI_4	1.000000e+00	
pos_patterns.pattern_12	479			SP3_HUMAN.H11MO.0.B	2.686780e-04	
pos_patterns.pattern_13	360			KLF9_HUMAN.H11MO.0.C	9.399620e-01	
pos_patterns.pattern_14	257			SP2_HUMAN.H11MO.0.A	1.163550e-04	
pos_patterns.pattern_15	252			RREB1_MA0073.1	3.139460e-05	
pos_patterns.pattern_16	194			SP1_HUMAN.H11MO.0.A	3.069150e-05	
pos_patterns.pattern_17	180			CTCFL_MOUSE.H11MO.0.A	1.797100e-02	
pos_patterns.pattern_18	52			POU2F2_MA0507.1	3.067460e-01	
pos_patterns.pattern_19	43			FOXD2_forkhead_1	5.494860e-02	
pos_patterns.pattern_20	25			Nfe2l2_MA0150.2	1.000000e+00	
neg_patterns.pattern_0	3581			TN5_2	2.479720e-03	
neg_patterns.pattern_1	3308			TN5_1	1.219860e-05	
neg_patterns.pattern_2	2277			TN5_1	6.221840e-03	
neg_patterns.pattern_3	1713			TN5_2	3.384470e-04	
neg_patterns.pattern_4	806			TN5_6	3.592370e-22	
neg_patterns.pattern_5	579			TN5_8	8.905280e-04	
neg_patterns.pattern_6	577			FOS_HUMAN.H11MO.0.A	1.000000e+00	
neg_patterns.pattern_7	478			TN5_4	1.598690e-02	

pattern	num_seqlets	cwm_fwd	cwm_rev	TOMTOM_match	TOMTOM_qval	TOMTOM_match_logo
neg_patterns.pattern_8	393			ZN770_HUMAN.H11MO.0.C	1.960270e-07	
neg_patterns.pattern_9	352			TN5_2	5.511530e-02	
neg_patterns.pattern_10	293			RARG_HUMAN.H11MO.0.B	1.000000e+00	
neg_patterns.pattern_11	262			Tcfl5_MA0632.1	5.869820e-01	
neg_patterns.pattern_12	173			ZN770_HUMAN.H11MO.0.C	1.042920e-07	
neg_patterns.pattern_13	110			DNASE_5	1.111950e-07	
neg_patterns.pattern_14	92			TN5_1	3.020640e-02	
neg_patterns.pattern_15	74			TFAP2C_TFAP_6	3.242090e-01	
neg_patterns.pattern_16	50			RUNX3_MOUSE.H11MO.0.A	9.944240e-01	
neg_patterns.pattern_17	47			EGR2_HUMAN.H11MO.0.A	3.985070e-02	
neg_patterns.pattern_18	36			EGR1_HUMAN.H11MO.0.A	2.161950e-01	
neg_patterns.pattern_19	35			DNASE_5	5.545120e-01	
neg_patterns.pattern_20	34			SMAD3_HUMAN.H11MO.0.B	2.377830e-01	
neg_patterns.pattern_21	34			DNASE_5	4.803090e-01	
neg_patterns.pattern_22	28			SOX21_HMG_2	6.000370e-01	
neg_patterns.pattern_23	28			KLF5_MOUSE.H11MO.0.A	3.100630e-01	
