# Supplementary material for: ChromBPNet: bias factorized, base-resolution deep learning models of chromatin accessibility reveal cis-regulatory sequence syntax, transcription factor footprints and regulatory variants
Source: bioRxiv. 2025 Jan 8:2024.12.25.630221. Preprint. [Version 2] doi: 10.1101/2024.12.25.630221 (PMC11741299; doi:10.1101/2024.12.25.630221)
Supplement: Supplement 4 [file media-4.zip › supplementary_files_3/imr90_ATAC_bpnet_bias_model/imr90_ATAC_raw_bpnet_bias_fold4_counts_modisco.pdf]

pattern	num_seqlets	cwm_fwd	cwm_rev	TOMTOM_match	TOMTOM_qval	TOMTOM_match_logo
pos_patterns.pattern_0	4588			ONECUT3_CUT_1	4.448680e-01	
pos_patterns.pattern_1	2414			ZNF384_MA1125.1	3.072980e-02	
pos_patterns.pattern_2	1550			DNASE_2	1.544380e-02	
pos_patterns.pattern_3	1404			ZFX_MOUSE.H11MO.0.B	1.489160e-01	
pos_patterns.pattern_4	1279			SP1_HUMAN.H11MO.0.A	8.089270e-02	
pos_patterns.pattern_5	1269			ZFX_MOUSE.H11MO.0.B	8.266320e-04	
pos_patterns.pattern_6	1159			SP2_HUMAN.H11MO.0.A	2.027550e-03	
pos_patterns.pattern_7	992			ZFX_MOUSE.H11MO.0.B	9.948390e-02	
pos_patterns.pattern_8	632			FOXJ3_HUMAN.H11MO.0.A	1.572650e-01	
pos_patterns.pattern_9	573			ZFX_MOUSE.H11MO.0.B	6.144880e-03	
pos_patterns.pattern_10	569			RUNX3_RUNX_3	1.301980e-01	
pos_patterns.pattern_11	527			TBX1_TBX_4	9.865440e-02	
pos_patterns.pattern_12	524			SP2_HUMAN.H11MO.0.A	2.248460e-01	
pos_patterns.pattern_13	500			SP2_HUMAN.H11MO.0.A	6.779970e-04	
pos_patterns.pattern_14	467			PRDM6_HUMAN.H11MO.0.C	4.383070e-01	
pos_patterns.pattern_15	431			RUNX2_HUMAN.H11MO.0.A	4.536790e-01	
pos_patterns.pattern_16	424			TBR1_TBX_2	3.821530e-01	
pos_patterns.pattern_17	364			SIX2_MA1119.1	1.000000e+00	
pos_patterns.pattern_18	248			TN5_8	1.000000e+00	
pos_patterns.pattern_19	217			ZSCAN4_C2H2_1	5.028780e-01	
pos_patterns.pattern_20	184			E2F5_HUMAN.H11MO.0.B	9.445140e-01	
pos_patterns.pattern_21	149			FOXC1_forkhead_1	4.307800e-02	
pos_patterns.pattern_22	139			TCF7L2_MA0523.1	9.547530e-02	
pos_patterns.pattern_23	139			LMX1B_MA0703.1	2.457080e-02	
pos_patterns.pattern_24	136			ZFX_MOUSE.H11MO.0.B	6.294900e-01	
pos_patterns.pattern_25	114			MAFF_MA0495.2	3.922880e-01	
pos_patterns.pattern_26	74			GLIS2_C2H2_1	1.000000e+00	
pos_patterns.pattern_27	69			SP2_HUMAN.H11MO.0.A	2.661840e-04	
pos_patterns.pattern_28	50			Nfe2l2_MA0150.2	1.000000e+00	

pattern	num_seqlets	cwm_fwd	cwm_rev	TOMTOM_match	TOMTOM_qval	TOMTOM_match_logo
pos_patterns.pattern_29	33	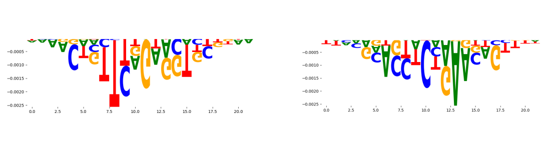		TN5_7	1.000000e+00	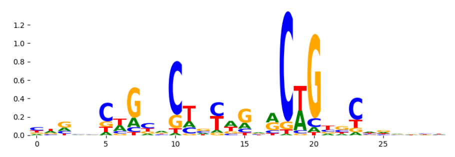
pos_patterns.pattern_30	30	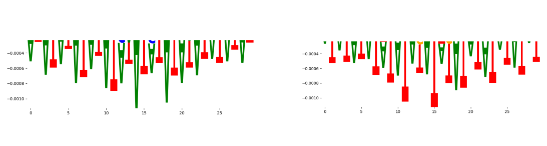		FOXB1_forkhead_2	3.978250e-01	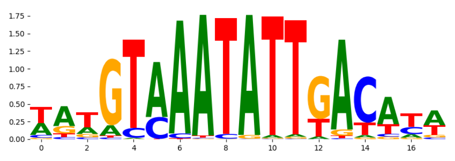
pos_patterns.pattern_31	20	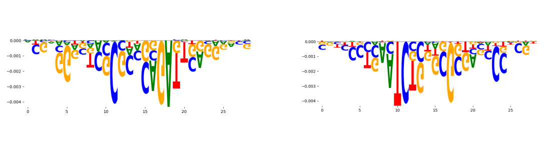		SP1_HUMAN.H11MO.0.A	1.000000e+00	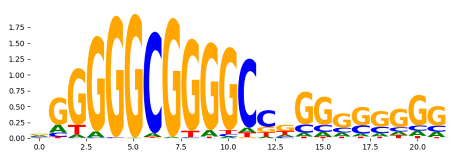
neg_patterns.pattern_0	1216	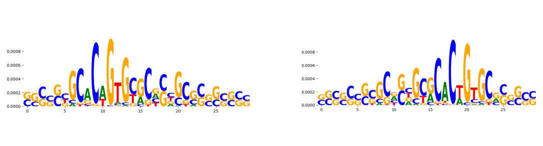		TN5_8	6.350700e-01	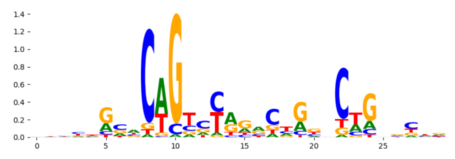
neg_patterns.pattern_1	990	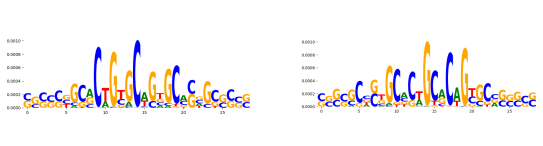		TN5_2	1.048260e-05	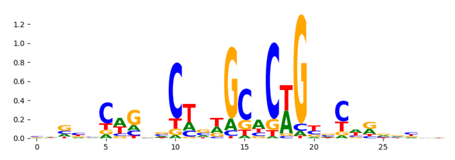
neg_patterns.pattern_2	568	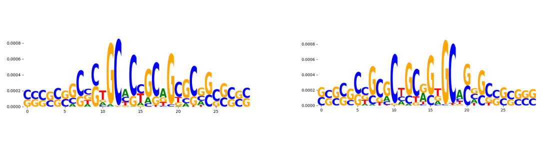		TN5_7	7.113750e-04	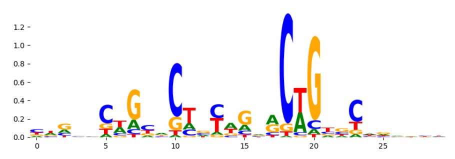
neg_patterns.pattern_3	410	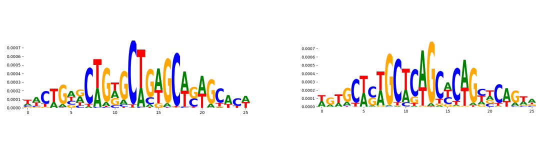		ZN331_HUMAN.H11MO.0.C	1.063680e-04	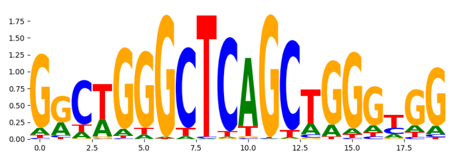
neg_patterns.pattern_4	235	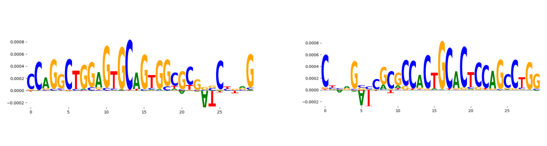		TN5_6	1.050940e-23	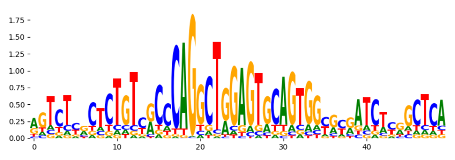
neg_patterns.pattern_5	196	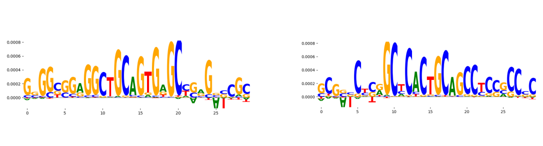		TAF1_HUMAN.H11MO.0.A	1.000000e+00	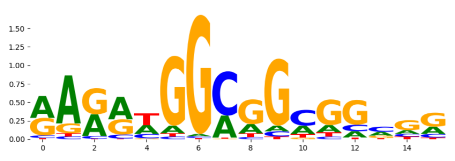
neg_patterns.pattern_6	190	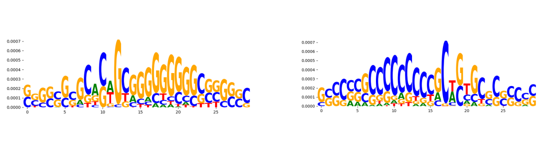		KLF9_HUMAN.H11MO.0.C	1.618260e-02	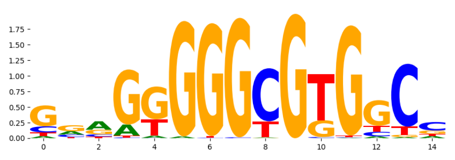
neg_patterns.pattern_7	185	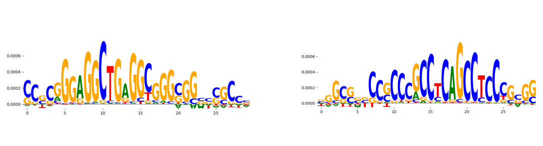		ZN770_HUMAN.H11MO.0.C	5.939930e-13	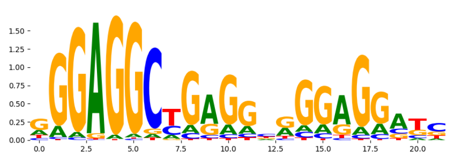
neg_patterns.pattern_8	183	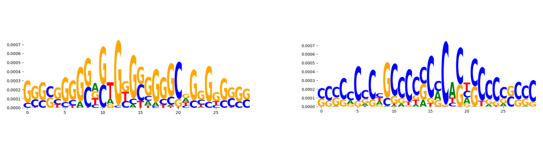		EGR2_HUMAN.H11MO.0.A	2.454810e-03	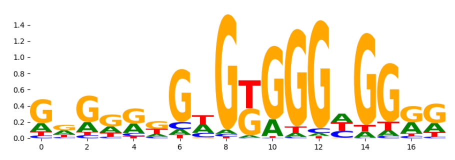
neg_patterns.pattern_9	168	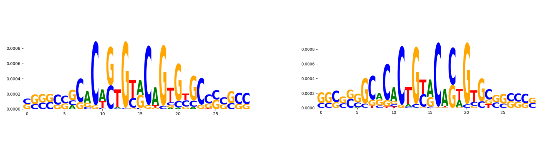		MTF1_C2H2_1	3.001770e-01	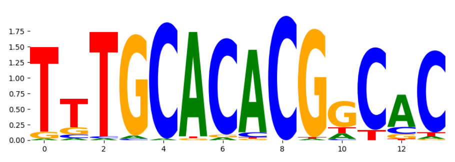
neg_patterns.pattern_10	106	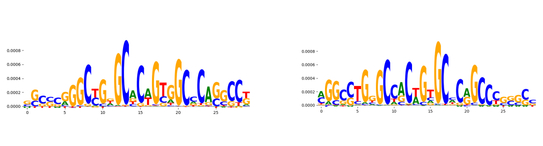		ZN331_HUMAN.H11MO.0.C	1.000000e+00	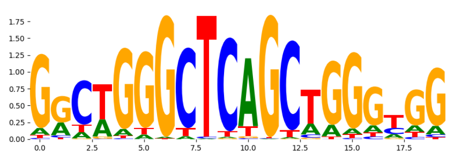
neg_patterns.pattern_11	90	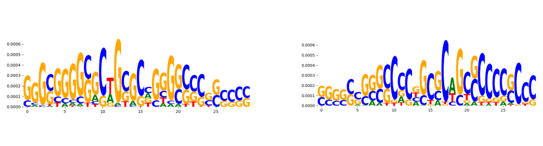		SP2_HUMAN.H11MO.0.A	1.652660e-04	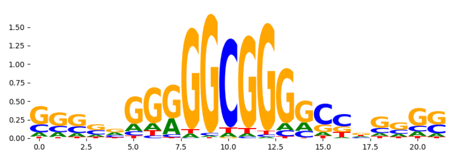
neg_patterns.pattern_12	82	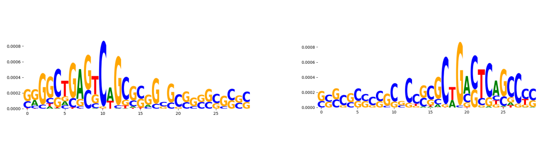		MAFB_HUMAN.H11MO.0.B	8.089600e-04	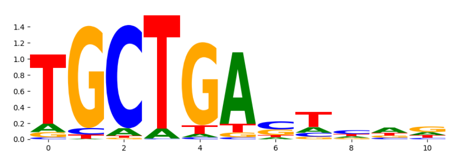
neg_patterns.pattern_13	82	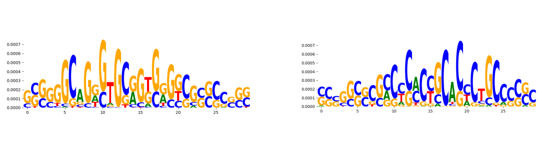		FOSL1_MA0477.1	5.134070e-02	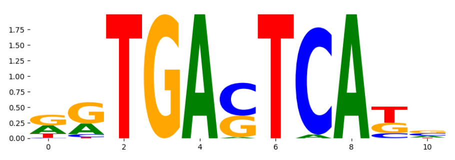
neg_patterns.pattern_14	43	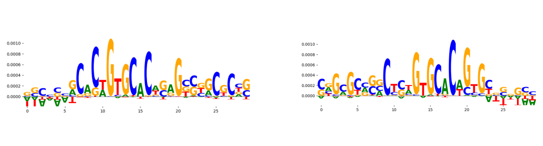		SOX9_HMG_2	1.000000e+00	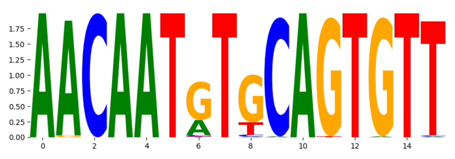
neg_patterns.pattern_15	30	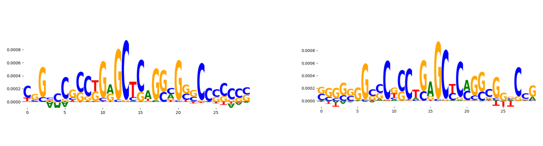		THA_HUMAN.H11MO.0.C	2.503940e-01	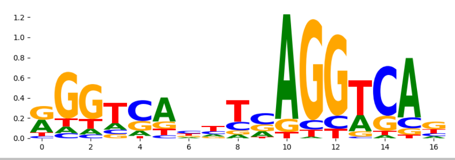
