# Supplementary material for: ChromBPNet: bias factorized, base-resolution deep learning models of chromatin accessibility reveal cis-regulatory sequence syntax, transcription factor footprints and regulatory variants
Source: bioRxiv. 2025 Jan 8:2024.12.25.630221. Preprint. [Version 2] doi: 10.1101/2024.12.25.630221 (PMC11741299; doi:10.1101/2024.12.25.630221)
Supplement: Supplement 4 [file media-4.zip › supplementary_files_3/gm12878_ATAC_bpnet_bias_model/gm12878_ATAC_raw_bpnet_bias_fold1_profile_modisco.pdf]

pattern	num_seqlets	cwm_fwd	cwm_rev	TOMTOM_match	TOMTOM_qval	TOMTOM_match_logo
pos_patterns.pattern_0	7822	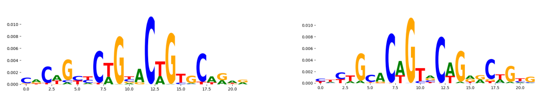		TN5_1	8.270390e-09	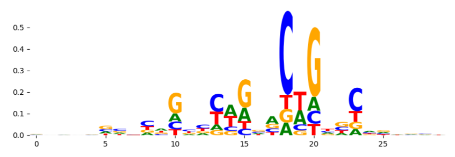
pos_patterns.pattern_1	5272	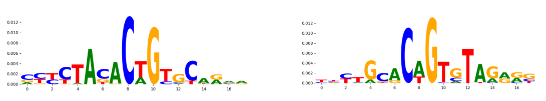		TN5_4	2.229310e-02	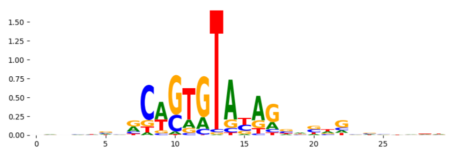
pos_patterns.pattern_2	4320	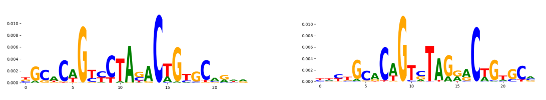		TN5_1	5.971440e-05	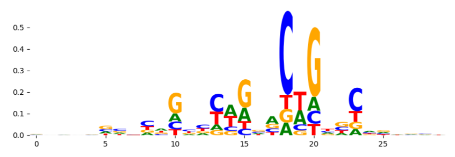
pos_patterns.pattern_3	2739	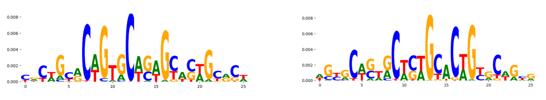		TN5_2	6.309330e-13	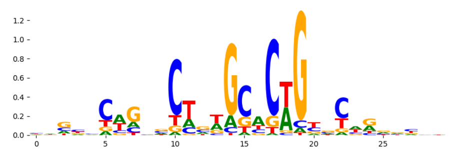
pos_patterns.pattern_4	2423	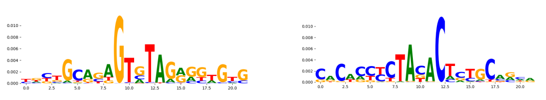		KLF4_MA0039.3	4.298560e-01	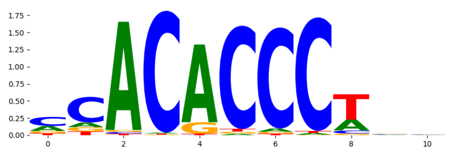
pos_patterns.pattern_5	2224	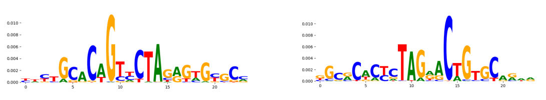		TN5_3	5.846380e-05	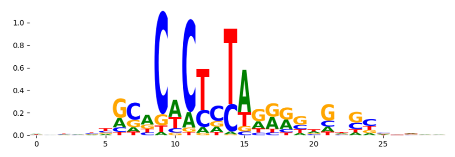
pos_patterns.pattern_6	1812	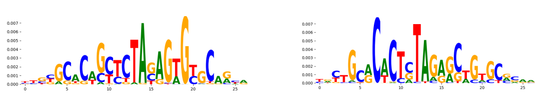		TN5_3	1.512530e-12	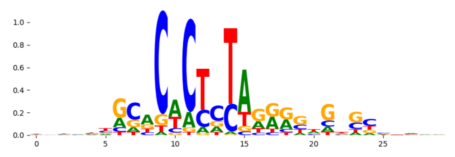
pos_patterns.pattern_7	1128	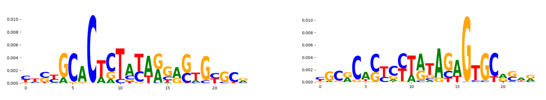		TN5_3	2.447420e-04	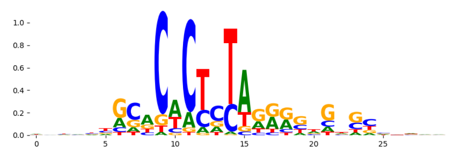
pos_patterns.pattern_8	603	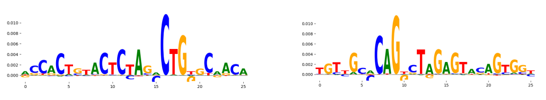		TN5_6	2.250980e-15	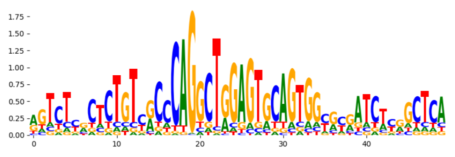
pos_patterns.pattern_9	556	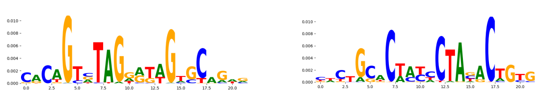		TN5_3	5.321250e-02	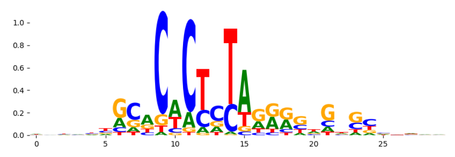
pos_patterns.pattern_10	185	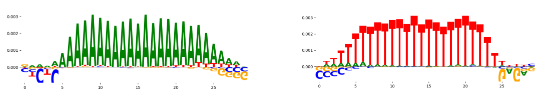		ZNF384_MA1125.1	8.226180e-02	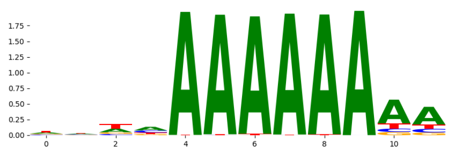
pos_patterns.pattern_11	134	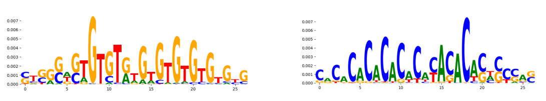		EGR2_HUMAN.H11MO.0.A	1.000000e+00	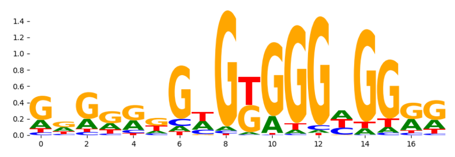
pos_patterns.pattern_12	98	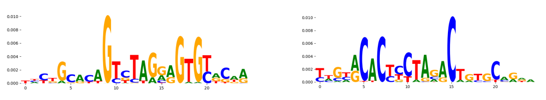		TN5_3	1.509640e-01	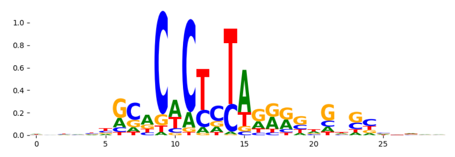
pos_patterns.pattern_13	50	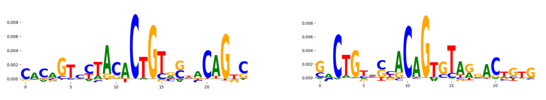		TN5_6	2.004820e-06	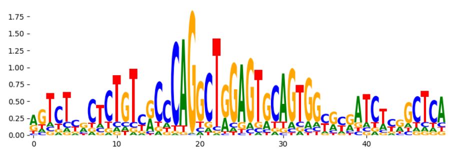
pos_patterns.pattern_14	43	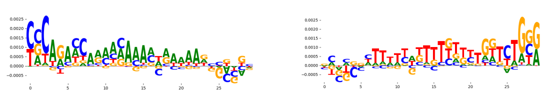		PRDM6_HUMAN.H11MO.0.C	4.299110e-02	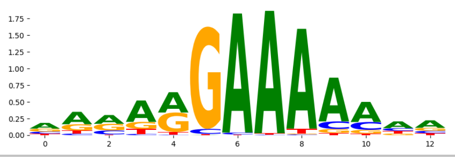
