# Supplementary material for: ChromBPNet: bias factorized, base-resolution deep learning models of chromatin accessibility reveal cis-regulatory sequence syntax, transcription factor footprints and regulatory variants
Source: bioRxiv. 2025 Jan 8:2024.12.25.630221. Preprint. [Version 2] doi: 10.1101/2024.12.25.630221 (PMC11741299; doi:10.1101/2024.12.25.630221)

pattern	num_seqlets	cwm_fwd	cwm_rev	TOMTOM_match	TOMTOM_qval	TOMTOM_match_logo
pos_patterns.pattern_0	16571			TN5_1	7.273770e-06	
pos_patterns.pattern_1	4565			TN5_2	2.013520e-20	
pos_patterns.pattern_2	3604			TN5_1	3.957760e-06	
pos_patterns.pattern_3	3200			TN5_1	1.591750e-06	
pos_patterns.pattern_4	3057			TN5_4	6.834380e-09	
pos_patterns.pattern_5	2439			TN5_7	5.285130e-07	
pos_patterns.pattern_6	2336			TN5_3	2.476830e-04	
pos_patterns.pattern_7	2300			TN5_3	2.384440e-17	
pos_patterns.pattern_8	1444			TN5_3	9.988090e-04	
pos_patterns.pattern_9	1046			TN5_6	7.668230e-14	
pos_patterns.pattern_10	1041			TN5_8	2.961390e-19	
pos_patterns.pattern_11	938			TN5_8	7.516760e-06	
pos_patterns.pattern_12	479			ZNF384_MA1125.1	4.203370e-02	
pos_patterns.pattern_13	280			TCF4_bHLH_2	8.339860e-01	
pos_patterns.pattern_14	257			TCF4_bHLH_2	4.585190e-01	
pos_patterns.pattern_15	185			TN5_4	4.439380e-01	
pos_patterns.pattern_16	165			EGR2_HUMAN.H11MO.0.A	1.000000e+00	
pos_patterns.pattern_17	97			OVOL1_HUMAN.H11MO.0.C	1.000000e+00	
pos_patterns.pattern_18	49			TN5_6	1.168370e-08	
pos_patterns.pattern_19	37			ZNF18_HUMAN.H11MO.0.C	4.381050e-01	
pos_patterns.pattern_20	39			ZN549_HUMAN.H11MO.0.C	1.000000e+00	

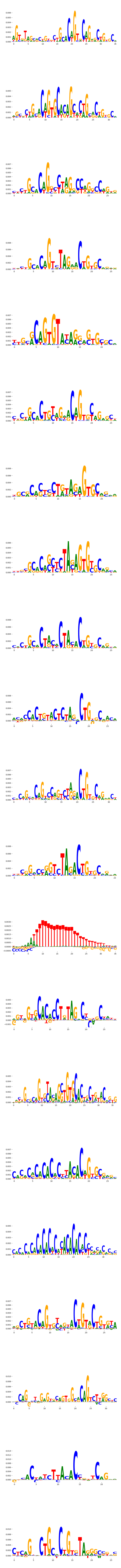
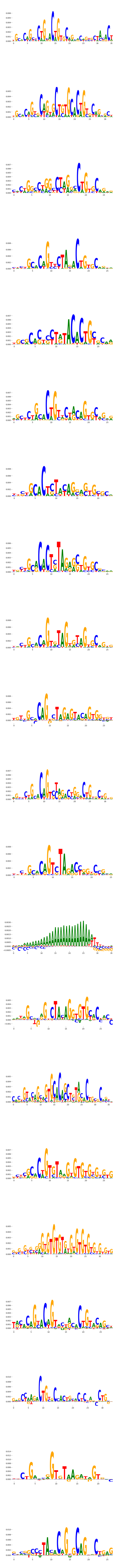
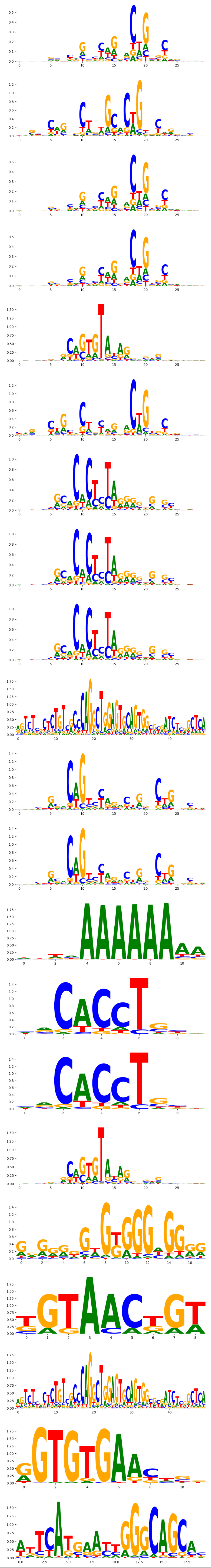

Supplement: Supplement 4 [file media-4.zip › supplementary_files_3/gm12878_ATAC_bpnet_bias_model/gm12878_ATAC_raw_bpnet_bias_fold0_profile_modisco.pdf]
