# Supplementary material for: ChromBPNet: bias factorized, base-resolution deep learning models of chromatin accessibility reveal cis-regulatory sequence syntax, transcription factor footprints and regulatory variants
Source: bioRxiv. 2025 Jan 8:2024.12.25.630221. Preprint. [Version 2] doi: 10.1101/2024.12.25.630221 (PMC11741299; doi:10.1101/2024.12.25.630221)
Supplement: Supplement 4 [file media-4.zip › supplementary_files_3/gm12878_ATAC_bpnet_bias_model/gm12878_ATAC_raw_bpnet_bias_fold3_profile_modisco.pdf]

pattern	num_seqlets	cwm_fwd	cwm_rev	TOMTOM_match	TOMTOM_qval	TOMTOM_match_logo
pos_patterns.pattern_0	9191	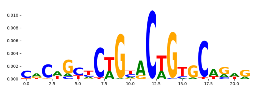	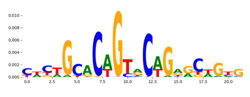	TN5_2	8.122110e-09	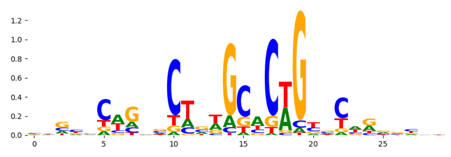
pos_patterns.pattern_1	5255	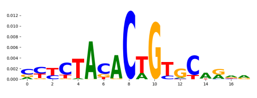	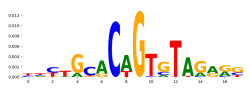	TN5_4	1.493170e-02	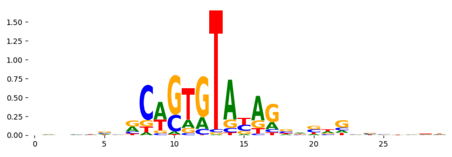
pos_patterns.pattern_2	4201	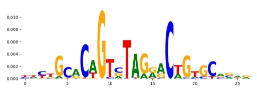	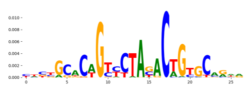	TN5_1	1.115290e-06	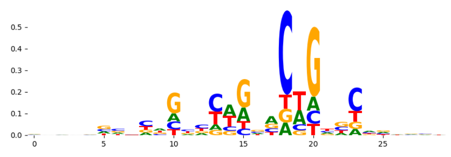
pos_patterns.pattern_3	3464	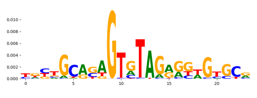	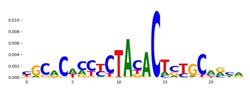	TN5_3	9.726860e-02	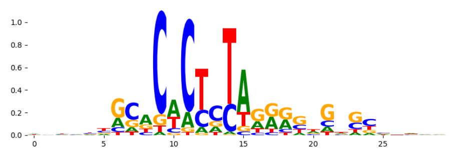
pos_patterns.pattern_4	2934	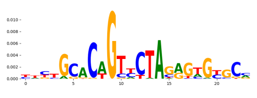	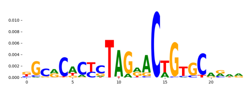	TN5_3	1.030630e-04	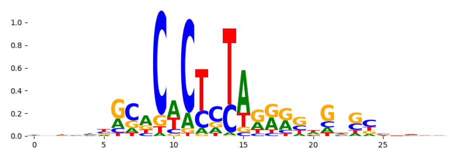
pos_patterns.pattern_5	2895	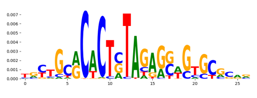	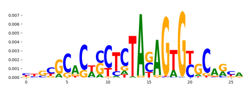	TN5_3	4.175510e-14	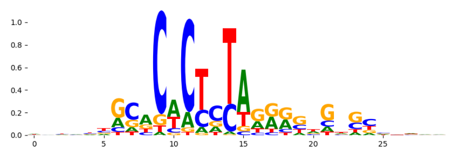
pos_patterns.pattern_6	606	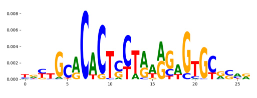	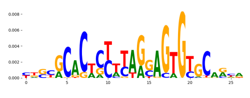	TN5_3	1.062170e-06	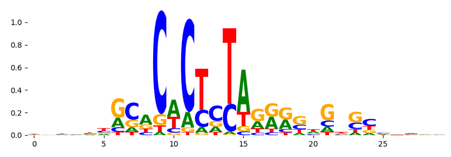
pos_patterns.pattern_7	480	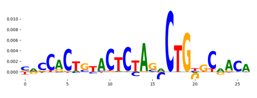	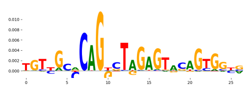	TN5_6	2.994360e-19	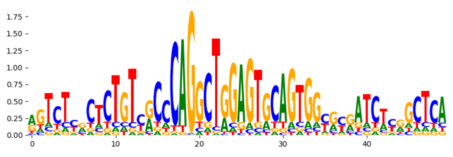
pos_patterns.pattern_8	219	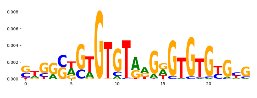	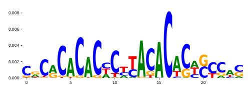	EGR2_HUMAN.H11MO.0.A	1.000000e+00	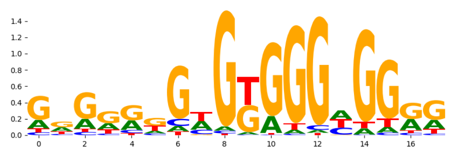
pos_patterns.pattern_9	47	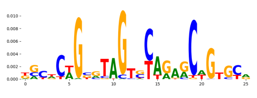	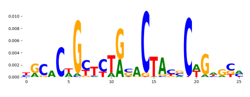	TN5_1	8.274060e-03	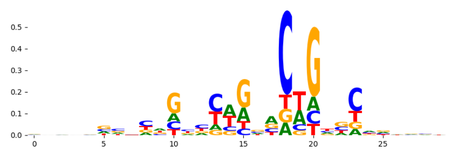
pos_patterns.pattern_10	40	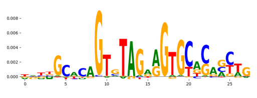	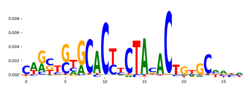	ID4_MA0824.1	1.000000e+00	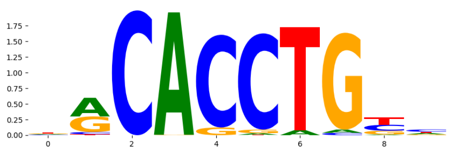
pos_patterns.pattern_11	23	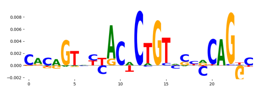	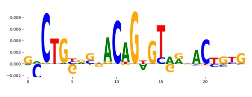	TN5_6	7.961920e-08	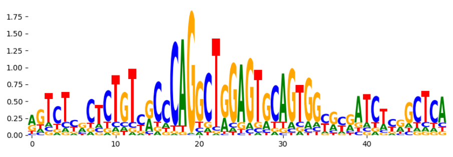
pos_patterns.pattern_12	20	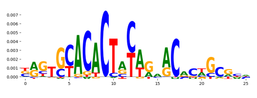	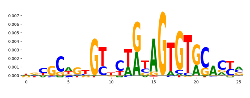	ZN554_HUMAN.H11MO.0.C	1.288120e-01	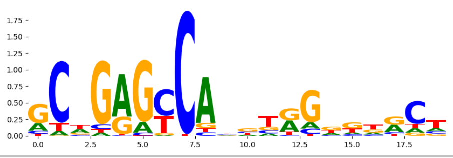
