# Supplementary material for: ChromBPNet: bias factorized, base-resolution deep learning models of chromatin accessibility reveal cis-regulatory sequence syntax, transcription factor footprints and regulatory variants
Source: bioRxiv. 2025 Jan 8:2024.12.25.630221. Preprint. [Version 2] doi: 10.1101/2024.12.25.630221 (PMC11741299; doi:10.1101/2024.12.25.630221)
Supplement: Supplement 4 [file media-4.zip › supplementary_files_3/gm12878_ATAC_bpnet_bias_model/gm12878_ATAC_raw_bpnet_bias_fold2_profile_modisco.pdf]

pattern	num_seqlets	cwm_fwd	cwm_rev	TOMTOM_match	TOMTOM_qval	TOMTOM_match_logo
pos_patterns.pattern_0	7437			TN5_1	7.658590e-10	
pos_patterns.pattern_1	5352			TN5_4	2.418690e-02	
pos_patterns.pattern_2	4089			TN5_1	1.024400e-06	
pos_patterns.pattern_3	2994			TN5_2	4.821820e-15	
pos_patterns.pattern_4	2552			TN5_3	2.025430e-01	
pos_patterns.pattern_5	2006			TN5_3	2.619100e-13	
pos_patterns.pattern_6	1154			TN5_3	1.152600e-05	
pos_patterns.pattern_7	788			TN5_4	6.802640e-06	
pos_patterns.pattern_8	659			TN5_4	5.366920e-06	
pos_patterns.pattern_9	550			TN5_6	4.211230e-16	
pos_patterns.pattern_10	522			TN5_7	1.324550e-07	
pos_patterns.pattern_11	389			TN5_3	5.795100e-04	
pos_patterns.pattern_12	383			TN5_3	5.830120e-04	
pos_patterns.pattern_13	191			ZNF384_MA1125.1	8.557220e-02	
pos_patterns.pattern_14	138			EGR2_HUMAN.H11MO.0.A	1.000000e+00	
pos_patterns.pattern_15	110			TN5_3	2.166000e-04	
pos_patterns.pattern_16	75			TBX1_TBX_1	2.409750e-01	
pos_patterns.pattern_17	74			TN5_6	8.135070e-07	
pos_patterns.pattern_18	55			TN5_6	6.431080e-01	
pos_patterns.pattern_19	38			TN5_6	9.888360e-03	
pos_patterns.pattern_20	27			TN5_3	3.540550e-02	
pos_patterns.pattern_21	20			NaN	NaN	
