# Supplementary material for: ChromBPNet: bias factorized, base-resolution deep learning models of chromatin accessibility reveal cis-regulatory sequence syntax, transcription factor footprints and regulatory variants
Source: bioRxiv. 2025 Jan 8:2024.12.25.630221. Preprint. [Version 2] doi: 10.1101/2024.12.25.630221 (PMC11741299; doi:10.1101/2024.12.25.630221)
Supplement: Supplement 4 [file media-4.zip › supplementary_files_3/gm12878_ATAC_bpnet_bias_model/gm12878_ATAC_raw_bpnet_bias_fold4_profile_modisco.pdf]

pattern	num_seqlets	cwm_fwd	cwm_rev	TOMTOM_match	TOMTOM_qval	TOMTOM_match_logo
pos_patterns.pattern_0	8115			TN5_2	2.446840e-09	
pos_patterns.pattern_1	5347			TN5_4	1.633960e-02	
pos_patterns.pattern_2	4304			TN5_1	8.777700e-05	
pos_patterns.pattern_3	2677			TN5_2	2.040260e-11	
pos_patterns.pattern_4	2464			TN5_3	3.504910e-11	
pos_patterns.pattern_5	2464			TN5_3	2.144590e-01	
pos_patterns.pattern_6	2038			TN5_3	1.059380e-04	
pos_patterns.pattern_7	591			TN5_3	1.926640e-06	
pos_patterns.pattern_8	543			TN5_4	2.793510e-06	
pos_patterns.pattern_9	530			TN5_6	4.519450e-15	
pos_patterns.pattern_10	138			TN5_4	2.263700e-02	
pos_patterns.pattern_11	119			TN5_3	1.196820e-04	
pos_patterns.pattern_12	114			EGR2_HUMAN.H11MO.0.A	1.000000e+00	
pos_patterns.pattern_13	22			TN5_3	2.866730e-04	
