# Supplementary material for: ChromBPNet: bias factorized, base-resolution deep learning models of chromatin accessibility reveal cis-regulatory sequence syntax, transcription factor footprints and regulatory variants
Source: bioRxiv. 2025 Jan 8:2024.12.25.630221. Preprint. [Version 2] doi: 10.1101/2024.12.25.630221 (PMC11741299; doi:10.1101/2024.12.25.630221)
Supplement: Supplement 4 [file media-4.zip › supplementary_files_3/gm12878_ATAC_bpnet_bias_model/gm12878_ATAC_raw_bpnet_bias_fold3_counts_modisco.pdf]

pattern	num_seqlets	cwm_fwd	cwm_rev	TOMTOM_match	TOMTOM_qval	TOMTOM_match_logo
pos_patterns.pattern_0	2898			RARA_HUMAN.H11MO.0.A	1.000000e+00	
pos_patterns.pattern_1	2446			FOXG1_forkhead_1	1.000000e+00	
pos_patterns.pattern_2	1909			ZFX_MOUSE.H11MO.0.B	1.642920e-01	
pos_patterns.pattern_3	1444			TN5_6	1.000000e+00	
pos_patterns.pattern_4	1125			ZFX_MOUSE.H11MO.0.B	8.413770e-01	
pos_patterns.pattern_5	845			ESR1_MA0112.3	1.000000e+00	
pos_patterns.pattern_6	716			CPEB1_RRM_1	7.049260e-01	
pos_patterns.pattern_7	609			NKX32_HUMAN.H11MO.0.C	2.201210e-02	
pos_patterns.pattern_8	593			RREB1_MA0073.1	4.349290e-01	
pos_patterns.pattern_9	588			SP3_HUMAN.H11MO.0.B	3.953510e-01	
pos_patterns.pattern_10	497			ARI5B_HUMAN.H11MO.0.C	5.935980e-01	
pos_patterns.pattern_11	495			SUH_HUMAN.H11MO.0.A	3.182160e-01	
pos_patterns.pattern_12	434			GLI2_C2H2_2	3.161670e-02	
pos_patterns.pattern_13	422			NFAC3_HUMAN.H11MO.0.B	7.273110e-01	
pos_patterns.pattern_14	414			SP1_HUMAN.H11MO.0.A	7.495330e-01	
pos_patterns.pattern_15	400			KLF9_HUMAN.H11MO.0.C	8.648230e-01	
pos_patterns.pattern_16	380			RXRG_nuclearreceptor_4	5.101380e-02	
pos_patterns.pattern_17	376			DNASE_2	2.645940e-01	
pos_patterns.pattern_18	357			RREB1_MA0073.1	1.742160e-01	
pos_patterns.pattern_19	357			SMAD4_MOUSE.H11MO.0.A	1.000000e+00	
pos_patterns.pattern_20	342			NRL_MA0842.1	1.816000e-01	
pos_patterns.pattern_21	338			IRF3_IRF_1	2.686040e-01	
pos_patterns.pattern_22	333			SP1_HUMAN.H11MO.0.A	1.000000e+00	
pos_patterns.pattern_23	277			HOXA13_homeodomain_1	1.263750e-01	
pos_patterns.pattern_24	263			RUNX2_HUMAN.H11MO.0.A	1.041400e-01	
pos_patterns.pattern_25	223			ZFX_MOUSE.H11MO.0.B	9.965160e-01	
pos_patterns.pattern_26	175			NFIB_MOUSE.H11MO.0.C	6.670110e-02	
pos_patterns.pattern_27	85			ZFP28_HUMAN.H11MO.0.C	1.000000e+00	
pos_patterns.pattern_28	78			ZN770_HUMAN.H11MO.0.C	9.184030e-01	

pattern	num_seqlets	cwm_fwd	cwm_rev	TOMTOM_match	TOMTOM_qval	TOMTOM_match_logo
pos_patterns.pattern_29	34			GLI1_MOUSE.H11MO.0.C	2.626020e-01	
neg_patterns.pattern_0	142			SPIB_HUMAN.H11MO.0.A	4.290230e-01	
neg_patterns.pattern_1	116			ZFX_MOUSE.H11MO.0.B	8.505990e-04	
neg_patterns.pattern_2	114			ZFX_MOUSE.H11MO.0.B	4.904940e-04	
neg_patterns.pattern_3	109			ZFX_MOUSE.H11MO.0.B	1.706840e-03	
neg_patterns.pattern_4	106			SP2_HUMAN.H11MO.0.A	6.364530e-07	
neg_patterns.pattern_5	102			ZN667_HUMAN.H11MO.0.C	1.000000e+00	
neg_patterns.pattern_6	98			SP2_HUMAN.H11MO.0.A	2.372990e-03	
neg_patterns.pattern_7	93			TCF7_MOUSE.H11MO.0.A	1.000000e+00	
neg_patterns.pattern_8	88			ZFX_MOUSE.H11MO.0.B	3.690310e-05	
neg_patterns.pattern_9	81			SP1_HUMAN.H11MO.0.A	1.025250e-03	
neg_patterns.pattern_10	73			FOXP2_MA0593.1	2.113070e-01	
neg_patterns.pattern_11	72			TN5_1	9.992260e-01	
neg_patterns.pattern_12	68			TN5_2	1.624960e-02	
neg_patterns.pattern_13	57			TN5_2	4.790880e-02	
neg_patterns.pattern_14	56			TN5_2	4.887350e-02	
neg_patterns.pattern_15	55			TN5_7	6.611330e-01	
neg_patterns.pattern_16	54			TN5_2	2.910640e-02	
neg_patterns.pattern_17	43			COT2_HUMAN.H11MO.0.A	8.533560e-02	
neg_patterns.pattern_18	38			TN5_2	1.379840e-03	
neg_patterns.pattern_19	37			TN5_2	1.552500e-03	
neg_patterns.pattern_20	34			TN5_2	2.809280e-01	
neg_patterns.pattern_21	31			TN5_8	2.540890e-02	
