# Supplementary material for: ChromBPNet: bias factorized, base-resolution deep learning models of chromatin accessibility reveal cis-regulatory sequence syntax, transcription factor footprints and regulatory variants
Source: bioRxiv. 2025 Jan 8:2024.12.25.630221. Preprint. [Version 2] doi: 10.1101/2024.12.25.630221 (PMC11741299; doi:10.1101/2024.12.25.630221)
Supplement: Supplement 4 [file media-4.zip › supplementary_files_3/gm12878_ATAC_bpnet_bias_model/gm12878_ATAC_raw_bpnet_bias_fold0_counts_modisco.pdf]

pattern	num_seqlets	cwm_fwd	cwm_rev	TOMTOM_match	TOMTOM_qval	TOMTOM_match_logo
pos_patterns.pattern_0	3167			ZBT17_HUMAN.H11MO.0.A	7.240880e-01	
pos_patterns.pattern_1	2396			ZFX_MOUSE.H11MO.0.B	1.578660e-02	
pos_patterns.pattern_2	2380			ZN436_HUMAN.H11MO.0.C	1.253010e-01	
pos_patterns.pattern_3	2290			GMEB2_SAND_2	1.000000e+00	
pos_patterns.pattern_4	2255			SP3_HUMAN.H11MO.0.B	7.758650e-03	
pos_patterns.pattern_5	1933			SOX2_HUMAN.H11MO.0.A	1.000000e+00	
pos_patterns.pattern_6	1552			NFATC2_MA0152.1	1.860260e-01	
pos_patterns.pattern_7	1502			RUNX2_HUMAN.H11MO.0.A	1.000000e+00	
pos_patterns.pattern_8	1443			RREB1_MA0073.1	3.905240e-07	
pos_patterns.pattern_9	1270			PAX5_MA0014.3	1.000000e+00	
pos_patterns.pattern_10	1254			RUNX2_HUMAN.H11MO.0.A	1.074140e-01	
pos_patterns.pattern_11	1119			TFAP4_HUMAN.H11MO.0.A	4.682400e-02	
pos_patterns.pattern_12	1081			STAT1_MOUSE.H11MO.0.A	1.000000e+00	
pos_patterns.pattern_13	1011			PRD16_MOUSE.H11MO.0.B	1.000000e+00	
pos_patterns.pattern_14	821			SP1_HUMAN.H11MO.0.A	5.556300e-01	
pos_patterns.pattern_15	816			NR4A2_nuclearreceptor_1	1.000000e+00	
pos_patterns.pattern_16	703			ZFX_MOUSE.H11MO.0.B	6.031190e-01	
pos_patterns.pattern_17	679			GCM1_GCM_3	1.514020e-01	
pos_patterns.pattern_18	595			PBX2_MA1113.1	1.000000e+00	
pos_patterns.pattern_19	589			KLF5_MOUSE.H11MO.0.A	1.000000e+00	
pos_patterns.pattern_20	564			NFAC2_HUMAN.H11MO.0.B	4.804290e-01	
pos_patterns.pattern_21	516			NFATC2_MA0152.1	2.844080e-02	
pos_patterns.pattern_22	431			SOX8_HMG_7	1.000000e+00	
pos_patterns.pattern_23	412			PRD14_HUMAN.H11MO.0.A	5.033940e-01	
pos_patterns.pattern_24	353			ZN140_HUMAN.H11MO.0.C	1.170690e-01	
pos_patterns.pattern_25	324			SOX8_HMG_7	3.262770e-01	
pos_patterns.pattern_26	309			KLF4_MA0039.3	5.791810e-01	
pos_patterns.pattern_27	294			KLF5_MOUSE.H11MO.0.A	8.831600e-01	
pos_patterns.pattern_28	303			NFKB2_MA0778.1	2.552380e-01	

pattern	num_seqlets	cwm_fwd	cwm_rev	TOMTOM_match	TOMTOM_qval	TOMTOM_match_logo
pos_patterns.pattern_29	294			BHA15_HUMAN.H11MO.0.B	1.000000e+00	
pos_patterns.pattern_30	287			NFAC3_HUMAN.H11MO.0.B	1.000000e+00	
pos_patterns.pattern_31	269			ASCL1_MOUSE.H11MO.0.A	6.632690e-01	
pos_patterns.pattern_32	252			SRBP2_HUMAN.H11MO.0.B	4.771640e-01	
pos_patterns.pattern_33	237			COE1_HUMAN.H11MO.0.A	1.000000e+00	
pos_patterns.pattern_34	193			SRY_HMG_2	6.872720e-01	
pos_patterns.pattern_35	144			ASCL1_MOUSE.H11MO.0.A	3.599420e-01	
pos_patterns.pattern_36	139			AP2A_MOUSE.H11MO.0.A	6.657940e-01	
pos_patterns.pattern_37	132			NaN	NaN	
pos_patterns.pattern_38	130			AP2C_MOUSE.H11MO.0.A	3.008310e-01	
pos_patterns.pattern_39	112			ZN547_HUMAN.H11MO.0.C	1.000000e+00	
pos_patterns.pattern_40	113			ZN331_HUMAN.H11MO.0.C	6.761010e-03	
pos_patterns.pattern_41	90			FOXO3_forkhead_3	5.862900e-01	
pos_patterns.pattern_42	84			FOXO3_forkhead_3	1.000000e+00	
pos_patterns.pattern_43	70			SPDEF_ETS_2	1.000000e+00	
pos_patterns.pattern_44	71			MYCN_HUMAN.H11MO.0.A	2.040050e-02	
pos_patterns.pattern_45	81			TFAP2B_MA0812.1	1.838620e-01	
pos_patterns.pattern_46	66			NRF1_MOUSE.H11MO.0.A	3.170360e-01	
pos_patterns.pattern_47	55			SOX4_HMG_1	7.582180e-01	
pos_patterns.pattern_48	42			FOXO3_forkhead_3	8.100940e-01	
pos_patterns.pattern_49	42			Hoxa9_MA0594.1	1.000000e+00	
pos_patterns.pattern_50	42			ZN436_HUMAN.H11MO.0.C	5.236760e-01	
pos_patterns.pattern_51	33			NFAC3_HUMAN.H11MO.0.B	6.859780e-01	
pos_patterns.pattern_52	35			TFAP2C_TFAP_6	6.529020e-01	
pos_patterns.pattern_53	26			ZNF282_C2H2_1	5.043650e-01	
neg_patterns.pattern_0	674			TN5_7	3.319080e-03	
neg_patterns.pattern_1	412			PRDM6_HUMAN.H11MO.0.C	1.326790e-01	
neg_patterns.pattern_2	316			TN5_2	1.090470e-01	
neg_patterns.pattern_3	281			TN5_2	7.529050e-05	
neg_patterns.pattern_4	221			SP2_HUMAN.H11MO.0.A	3.044040e-08	

pattern	num_seqlets	cwm_fwd	cwm_rev	TOMTOM_match	TOMTOM_qval	TOMTOM_match_logo
neg_patterns.pattern_5	227			ZN667_HUMAN.H11MO.0.C	6.987780e-01	
neg_patterns.pattern_6	125			SP2_HUMAN.H11MO.0.A	3.498470e-04	
neg_patterns.pattern_7	119			TN5_2	1.160430e-04	
neg_patterns.pattern_8	78			TN5_2	1.601800e-04	
neg_patterns.pattern_9	67			SP2_HUMAN.H11MO.0.A	7.469830e-08	
neg_patterns.pattern_10	81			Zic3.mouse_C2H2_1	1.000000e+00	
neg_patterns.pattern_11	66			SP2_HUMAN.H11MO.0.A	1.543380e-01	
neg_patterns.pattern_12	74			ZNF384_MA1125.1	1.467600e-02	
neg_patterns.pattern_13	59			PBX3_MA1114.1	2.259020e-01	
neg_patterns.pattern_14	51			RREB1_MA0073.1	1.000000e+00	
neg_patterns.pattern_15	49			ELF1_HUMAN.H11MO.0.A	8.238480e-01	
neg_patterns.pattern_16	49			TN5_2	2.175870e-04	
neg_patterns.pattern_17	38			ZN431_MOUSE.H11MO.0.C	1.000000e+00	
neg_patterns.pattern_18	33			TN5_8	3.674490e-02	
neg_patterns.pattern_19	40			DNASE_5	1.504450e-06	
neg_patterns.pattern_20	30			SP1_HUMAN.H11MO.0.A	2.234150e-05	
neg_patterns.pattern_21	33			NR1D1_HUMAN.H11MO.0.B	9.290610e-02	
neg_patterns.pattern_22	41			TBX20_MOUSE.H11MO.0.C	1.847560e-01	
neg_patterns.pattern_23	37			TN5_6	2.173160e-12	
neg_patterns.pattern_24	25			TP63_MA0525.2	1.000000e+00	
neg_patterns.pattern_25	40			Foxd3_MA0041.1	9.308280e-02	
