# Supplementary material for: ChromBPNet: bias factorized, base-resolution deep learning models of chromatin accessibility reveal cis-regulatory sequence syntax, transcription factor footprints and regulatory variants
Source: bioRxiv. 2025 Jan 8:2024.12.25.630221. Preprint. [Version 2] doi: 10.1101/2024.12.25.630221 (PMC11741299; doi:10.1101/2024.12.25.630221)
Supplement: Supplement 4 [file media-4.zip › supplementary_files_3/gm12878_ATAC_bpnet_bias_model/gm12878_ATAC_raw_bpnet_bias_fold1_counts_modisco.pdf]

pattern	num_seqlets	cwm_fwd	cwm_rev	TOMTOM_match	TOMTOM_qval	TOMTOM_match_logo
pos_patterns.pattern_0	3468			SP2_HUMAN.H11MO.0.A	9.197300e-03	
pos_patterns.pattern_1	2224			ZN384_HUMAN.H11MO.0.C	1.000000e+00	
pos_patterns.pattern_2	807			RREB1_MA0073.1	7.863040e-03	
pos_patterns.pattern_3	802			RHOXF1_homeodomain_1	1.000000e+00	
pos_patterns.pattern_4	757			RORA_nuclearreceptor_1	1.000000e+00	
pos_patterns.pattern_5	739			RELB_HUMAN.H11MO.0.C	2.754320e-01	
pos_patterns.pattern_6	699			TBX20_MA0689.1	4.031450e-01	
pos_patterns.pattern_7	684			RXRG_nuclearreceptor_4	9.522160e-01	
pos_patterns.pattern_8	633			STAT2_HUMAN.H11MO.0.A	8.953190e-01	
pos_patterns.pattern_9	567			THB_HUMAN.H11MO.0.C	1.082970e-01	
pos_patterns.pattern_10	563			RREB1_MA0073.1	2.341980e-02	
pos_patterns.pattern_11	547			BHA15_HUMAN.H11MO.0.B	9.723000e-01	
pos_patterns.pattern_12	540			ZBTB6_HUMAN.H11MO.0.C	1.000000e+00	
pos_patterns.pattern_13	516			Ddit3+Cebpa_MA0019.1	1.000000e+00	
pos_patterns.pattern_14	516			Nr5a2_MA0505.1	4.599300e-01	
pos_patterns.pattern_15	505			ZBTB7A_C2H2_1	8.056770e-01	
pos_patterns.pattern_16	490			ZNF384_MA1125.1	6.929320e-02	
pos_patterns.pattern_17	446			RREB1_MA0073.1	2.350810e-02	
pos_patterns.pattern_18	430			WT1_HUMAN.H11MO.0.C	6.992450e-02	
pos_patterns.pattern_19	428			RREB1_MA0073.1	9.972970e-01	
pos_patterns.pattern_20	407			SP1_HUMAN.H11MO.0.A	4.917680e-01	
pos_patterns.pattern_21	343			ID4_MA0824.1	9.237330e-01	
pos_patterns.pattern_22	331			ASCL1_MA1100.1	8.585940e-01	
pos_patterns.pattern_23	261			ERR1_MOUSE.H11MO.0.A	1.000000e+00	
pos_patterns.pattern_24	238			ZN250_HUMAN.H11MO.0.C	1.000000e+00	
pos_patterns.pattern_25	233			RREB1_MA0073.1	9.153480e-05	
pos_patterns.pattern_26	193			ZNF384_MA1125.1	2.969120e-02	
pos_patterns.pattern_27	143			ZBTB6_HUMAN.H11MO.0.C	2.469610e-01	
pos_patterns.pattern_28	85			IRF8_IRF_1	8.774610e-01	

pattern	num_seqlets	cwm_fwd	cwm_rev	TOMTOM_match	TOMTOM_qval	TOMTOM_match_logo
pos_patterns.pattern_29	84			Nr5a2_MA0505.1	1.000000e+00	
pos_patterns.pattern_30	83			RREB1_MA0073.1	5.745850e-01	
pos_patterns.pattern_31	56			SP3_HUMAN.H11MO.0.B	4.332280e-01	
pos_patterns.pattern_32	50			HXA1_HUMAN.H11MO.0.C	4.626460e-01	
pos_patterns.pattern_33	49			RREB1_MA0073.1	8.210420e-01	
pos_patterns.pattern_34	41			ZNF282_C2H2_1	1.000000e+00	
pos_patterns.pattern_35	36			SP2_HUMAN.H11MO.0.A	3.423870e-01	
pos_patterns.pattern_36	32			NHLH1_bHLH_1	1.000000e+00	
pos_patterns.pattern_37	28			TBX20_TBX_5	1.000000e+00	
neg_patterns.pattern_0	184			SP2_HUMAN.H11MO.0.A	1.106890e-04	
neg_patterns.pattern_1	158			ZN331_HUMAN.H11MO.0.C	8.456850e-03	
neg_patterns.pattern_2	156			SP2_HUMAN.H11MO.0.A	7.873710e-05	
neg_patterns.pattern_3	150			TN5_1	5.538670e-04	
neg_patterns.pattern_4	131			RREB1_MA0073.1	1.000000e+00	
neg_patterns.pattern_5	116			NR4A2_nuclearreceptor_3	2.080070e-01	
neg_patterns.pattern_6	104			ZN341_HUMAN.H11MO.0.C	6.459850e-02	
neg_patterns.pattern_7	104			ZN667_HUMAN.H11MO.0.C	1.445040e-01	
neg_patterns.pattern_8	102			ZFX_MOUSE.H11MO.0.B	7.238720e-04	
neg_patterns.pattern_9	93			PRDM1_MA0508.2	1.334930e-01	
neg_patterns.pattern_10	88			SP2_HUMAN.H11MO.0.A	2.679590e-08	
neg_patterns.pattern_11	79			MECP2_HUMAN.H11MO.0.C	1.424340e-01	
neg_patterns.pattern_12	77			TN5_2	9.999990e-01	
neg_patterns.pattern_13	71			TN5_1	1.000000e+00	
neg_patterns.pattern_14	70			ZFX_MOUSE.H11MO.0.B	1.348220e-04	
neg_patterns.pattern_15	69			NR2C2_MA0504.1	5.361540e-02	
neg_patterns.pattern_16	68			ZN331_HUMAN.H11MO.0.C	3.358310e-01	
neg_patterns.pattern_17	41			EGR1_HUMAN.H11MO.0.A	4.039400e-02	
neg_patterns.pattern_18	36			ZN322_MOUSE.H11MO.0.B	1.000000e+00	
