# Supplementary material for: ChromBPNet: bias factorized, base-resolution deep learning models of chromatin accessibility reveal cis-regulatory sequence syntax, transcription factor footprints and regulatory variants
Source: bioRxiv. 2025 Jan 8:2024.12.25.630221. Preprint. [Version 2] doi: 10.1101/2024.12.25.630221 (PMC11741299; doi:10.1101/2024.12.25.630221)
Supplement: Supplement 4 [file media-4.zip › supplementary_files_3/gm12878_ATAC_bpnet_bias_model/gm12878_ATAC_raw_bpnet_bias_fold2_counts_modisco.pdf]

pattern	num_seqlets	cwm_fwd	cwm_rev	TOMTOM_match	TOMTOM_qval	TOMTOM_match_logo
pos_patterns.pattern_0	5009			Zfx_MA0146.2	3.692740e-01	
pos_patterns.pattern_1	3048			PRD16_MOUSE.H11MO.0.B	2.560730e-03	
pos_patterns.pattern_2	1014			TBX21_TBX_4	6.613920e-01	
pos_patterns.pattern_3	955			KLF5_MOUSE.H11MO.0.A	9.115280e-01	
pos_patterns.pattern_4	865			ZN467_HUMAN.H11MO.0.C	1.522520e-08	
pos_patterns.pattern_5	696			ZNF85_HUMAN.H11MO.0.C	1.000000e+00	
pos_patterns.pattern_6	620			ZNF143_C2H2_1	2.731160e-01	
pos_patterns.pattern_7	568			ZIM3_HUMAN.H11MO.0.C	6.123900e-02	
pos_patterns.pattern_8	552			ZN382_HUMAN.H11MO.0.C	1.414040e-01	
pos_patterns.pattern_9	504			SIX1_HUMAN.H11MO.0.A	9.999980e-01	
pos_patterns.pattern_10	493			ZNF354C_MA0130.1	1.000000e+00	
pos_patterns.pattern_11	490			TF65_HUMAN.H11MO.0.A	2.609800e-01	
pos_patterns.pattern_12	459			ZNF524_C2H2_1	1.557100e-01	
pos_patterns.pattern_13	453			PRDM6_HUMAN.H11MO.0.C	9.410920e-03	
pos_patterns.pattern_14	404			SOX17_HUMAN.H11MO.0.C	7.245020e-01	
pos_patterns.pattern_15	365			FOXO1_HUMAN.H11MO.0.A	5.786890e-01	
pos_patterns.pattern_16	333			BATF3_HUMAN.H11MO.0.B	1.000000e+00	
pos_patterns.pattern_17	296			ARI5B_HUMAN.H11MO.0.C	1.000000e+00	
pos_patterns.pattern_18	289			ZNF384_MA1125.1	8.713580e-02	
pos_patterns.pattern_19	275			PITX1_MOUSE.H11MO.0.C	2.281660e-01	
pos_patterns.pattern_20	251			ZNF384_MA1125.1	1.496470e-02	
pos_patterns.pattern_21	231			BATF_MOUSE.H11MO.0.A	1.000000e+00	
pos_patterns.pattern_22	225			NFAC2_HUMAN.H11MO.0.B	5.257230e-01	
pos_patterns.pattern_23	73			SMAD3_HUMAN.H11MO.0.B	1.000000e+00	
pos_patterns.pattern_24	67			RELA_MA0107.1	9.228360e-01	
pos_patterns.pattern_25	39			THAP1_HUMAN.H11MO.0.C	1.845960e-01	
pos_patterns.pattern_26	25			GLIS1_C2H2_1	1.000000e+00	
neg_patterns.pattern_0	357			ZN331_HUMAN.H11MO.0.C	1.027600e-01	
neg_patterns.pattern_1	152			ERG_HUMAN.H11MO.0.A	3.322090e-01	

pattern	num_seqlets	cwm_fwd	cwm_rev	TOMTOM_match	TOMTOM_qval	TOMTOM_match_logo
neg_patterns.pattern_2	149			TN5_1	4.369900e-01	
neg_patterns.pattern_3	145			ZN667_HUMAN.H11MO.0.C	1.000000e+00	
neg_patterns.pattern_4	138			TN5_2	1.724740e-02	
neg_patterns.pattern_5	131			TN5_2	4.913220e-03	
neg_patterns.pattern_6	131			ETV5_HUMAN.H11MO.0.C	3.620440e-02	
neg_patterns.pattern_7	122			TN5_1	1.199890e-05	
neg_patterns.pattern_8	112			ZN667_HUMAN.H11MO.0.C	1.000000e+00	
neg_patterns.pattern_9	111			TN5_2	7.222020e-02	
neg_patterns.pattern_10	111			PRDM6_HUMAN.H11MO.0.C	5.925330e-02	
neg_patterns.pattern_11	109			TN5_1	1.482230e-01	
neg_patterns.pattern_12	108			TN5_2	1.000000e+00	
neg_patterns.pattern_13	105			NR1H3_MOUSE.H11MO.0.A	5.086500e-01	
neg_patterns.pattern_14	82			ERR1_HUMAN.H11MO.0.A	1.000000e+00	
neg_patterns.pattern_15	77			Z324A_HUMAN.H11MO.0.C	1.648230e-01	
neg_patterns.pattern_16	54			TAL1_HUMAN.H11MO.0.A	5.554190e-01	
neg_patterns.pattern_17	46			SP2_HUMAN.H11MO.0.A	3.132240e-02	
neg_patterns.pattern_18	41			RREB1_MA0073.1	1.000000e+00	
