# Supplementary material for: ChromBPNet: bias factorized, base-resolution deep learning models of chromatin accessibility reveal cis-regulatory sequence syntax, transcription factor footprints and regulatory variants
Source: bioRxiv. 2025 Jan 8:2024.12.25.630221. Preprint. [Version 2] doi: 10.1101/2024.12.25.630221 (PMC11741299; doi:10.1101/2024.12.25.630221)
Supplement: Supplement 4 [file media-4.zip › supplementary_files_3/gm12878_ATAC_bpnet_bias_model/gm12878_ATAC_raw_bpnet_bias_fold4_counts_modisco.pdf]

pattern	num_seqlets	cwm_fwd	cwm_rev	TOMTOM_match	TOMTOM_qval	TOMTOM_match_logo
pos_patterns.pattern_0	3970			NaN	NaN	
pos_patterns.pattern_1	1938			ZBT17_MOUSE.H11MO.0.A	5.572700e-01	
pos_patterns.pattern_2	1263			NaN	NaN	
pos_patterns.pattern_3	993			SP1_HUMAN.H11MO.0.A	3.986790e-02	
pos_patterns.pattern_4	778			TBX21_TBX_4	2.394480e-01	
pos_patterns.pattern_5	762			HIC1_HUMAN.H11MO.0.C	3.479560e-01	
pos_patterns.pattern_6	695			RUNX3_RUNX_3	4.701590e-01	
pos_patterns.pattern_7	655			TF65_HUMAN.H11MO.0.A	3.217300e-01	
pos_patterns.pattern_8	552			STAT1_MOUSE.H11MO.0.A	1.846030e-03	
pos_patterns.pattern_9	457			RELB_HUMAN.H11MO.0.C	1.016070e-01	
pos_patterns.pattern_10	457			RREB1_MA0073.1	7.600580e-02	
pos_patterns.pattern_11	450			RXRG_nuclearreceptor_4	1.000000e+00	
pos_patterns.pattern_12	447			RREB1_MA0073.1	2.713940e-03	
pos_patterns.pattern_13	418			SMAD4_MOUSE.H11MO.0.A	1.000000e+00	
pos_patterns.pattern_14	417			ZBT17_MOUSE.H11MO.0.A	1.036140e-01	
pos_patterns.pattern_15	392			ZN436_HUMAN.H11MO.0.C	1.000000e+00	
pos_patterns.pattern_16	389			RREB1_MA0073.1	1.768800e-02	
pos_patterns.pattern_17	375			ZFX_HUMAN.H11MO.0.A	1.000000e+00	
pos_patterns.pattern_18	372			Bach1+Mafk_MA0591.1	5.560770e-01	
pos_patterns.pattern_19	356			RREB1_MA0073.1	2.065060e-01	
pos_patterns.pattern_20	310			RREB1_MA0073.1	1.504670e-01	
pos_patterns.pattern_21	303			NFAC3_HUMAN.H11MO.0.B	6.362900e-01	
pos_patterns.pattern_22	291			SMAD2_HUMAN.H11MO.0.A	2.561830e-01	
pos_patterns.pattern_23	288			ZFX_MOUSE.H11MO.0.B	5.131410e-01	
pos_patterns.pattern_24	186			SOX8_HMG_6	1.000000e+00	
pos_patterns.pattern_25	181			FOXO1_HUMAN.H11MO.0.A	1.000000e+00	
pos_patterns.pattern_26	164			RUNX3_RUNX_2	1.000000e+00	
pos_patterns.pattern_27	147			HINFP1_C2H2_3	5.035720e-01	
pos_patterns.pattern_28	144			FEV_HUMAN.H11MO.0.B	1.000000e+00	
pos_patterns.pattern_29	128			SALL4_HUMAN.H11MO.0.B	1.000000e+00	

pattern	num_seqlets	cwm_fwd	cwm_rev	TOMTOM_match	TOMTOM_qval	TOMTOM_match_logo
pos_patterns.pattern_30	116			HINFP1_C2H2_3	1.000000e+00	
pos_patterns.pattern_31	103			PBX3_MA1114.1	3.555710e-01	
pos_patterns.pattern_32	76			FOXB1_forkhead_2	2.698020e-01	
pos_patterns.pattern_33	51			ZN502_HUMAN.H11MO.0.C	1.353210e-01	
pos_patterns.pattern_34	34			Zfx_MA0146.2	1.000000e+00	
pos_patterns.pattern_35	29			ZN384_HUMAN.H11MO.0.C	9.938560e-02	
pos_patterns.pattern_36	25			ZN502_HUMAN.H11MO.0.C	1.457090e-07	
pos_patterns.pattern_37	22			USF1_MA0093.2	1.000000e+00	
pos_patterns.pattern_38	21			ZNF524_C2H2_2	1.000000e+00	
neg_patterns.pattern_0	447			TN5_2	3.500820e-03	
neg_patterns.pattern_1	183			TBX20_MOUSE.H11MO.0.C	1.398580e-01	
neg_patterns.pattern_2	167			NKX28_HUMAN.H11MO.0.C	9.664850e-01	
neg_patterns.pattern_3	159			SP2_HUMAN.H11MO.0.A	5.926800e-08	
neg_patterns.pattern_4	149			ZFX_MOUSE.H11MO.0.B	9.049580e-03	
neg_patterns.pattern_5	148			TN5_7	1.613830e-02	
neg_patterns.pattern_6	140			TF7L1_MOUSE.H11MO.0.A	1.000000e+00	
neg_patterns.pattern_7	133			COT2_MOUSE.H11MO.0.A	1.882980e-01	
neg_patterns.pattern_8	128			SP2_HUMAN.H11MO.0.A	1.155020e-05	
neg_patterns.pattern_9	119			TN5_2	3.746920e-07	
neg_patterns.pattern_10	116			ETV2_HUMAN.H11MO.0.B	5.796730e-03	
neg_patterns.pattern_11	113			ZFX_MOUSE.H11MO.0.B	2.137900e-02	
neg_patterns.pattern_12	111			PRDM6_HUMAN.H11MO.0.C	7.472100e-03	
neg_patterns.pattern_13	110			FOXO1_HUMAN.H11MO.0.A	7.695380e-01	
neg_patterns.pattern_14	101			TP53_MA0106.3	1.000000e+00	
neg_patterns.pattern_15	77			SP1_MOUSE.H11MO.0.A	2.256300e-08	
neg_patterns.pattern_16	74			SP3_HUMAN.H11MO.0.B	1.901320e-04	
neg_patterns.pattern_17	65			NRF1_MOUSE.H11MO.0.A	9.720380e-03	
neg_patterns.pattern_18	44			TN5_1	5.125290e-03	
neg_patterns.pattern_19	40			TN5_8	1.000000e+00	
