# Supplementary material for: ChromBPNet: bias factorized, base-resolution deep learning models of chromatin accessibility reveal cis-regulatory sequence syntax, transcription factor footprints and regulatory variants
Source: bioRxiv. 2025 Jan 8:2024.12.25.630221. Preprint. [Version 2] doi: 10.1101/2024.12.25.630221 (PMC11741299; doi:10.1101/2024.12.25.630221)
Supplement: Supplement 4 [file media-4.zip › supplementary_files_3/h1esc_ATAC_bpnet_bias_model/h1esc_ATAC_raw_bpnet_bias_fold4_counts_modisco.pdf]

pattern	num_seqlets	cwm_fwd	cwm_rev
pos_patterns.pattern_0	136	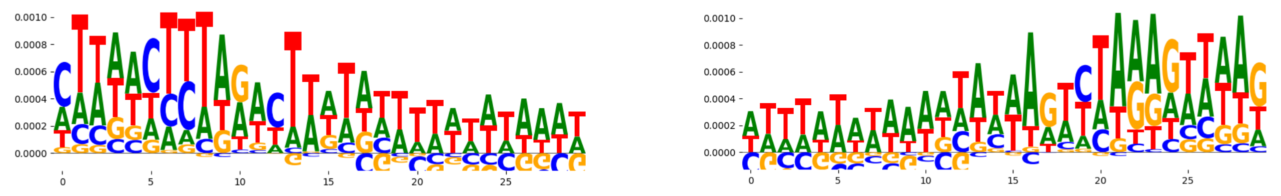	
pos_patterns.pattern_1	127	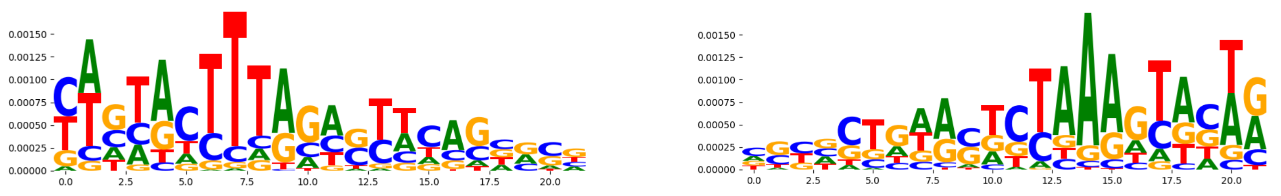	
pos_patterns.pattern_2	81	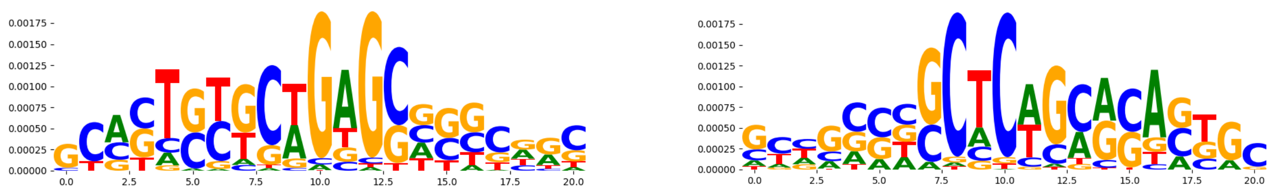	
pos_patterns.pattern_3	79	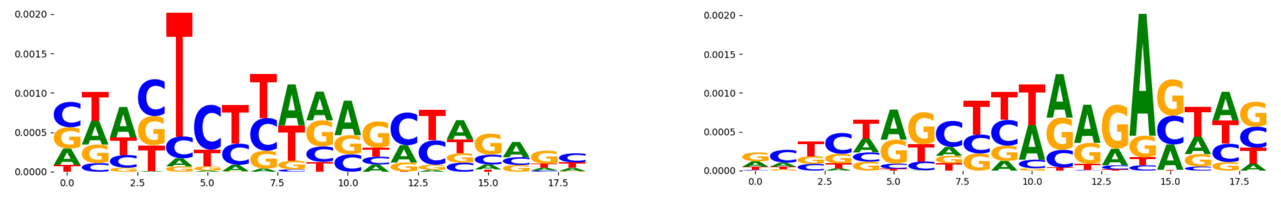	
pos_patterns.pattern_4	67	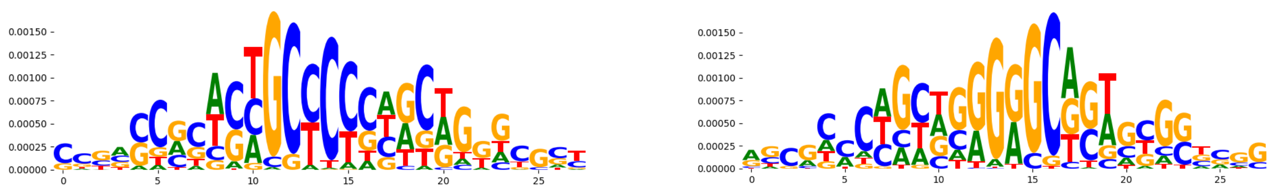	
pos_patterns.pattern_5	64	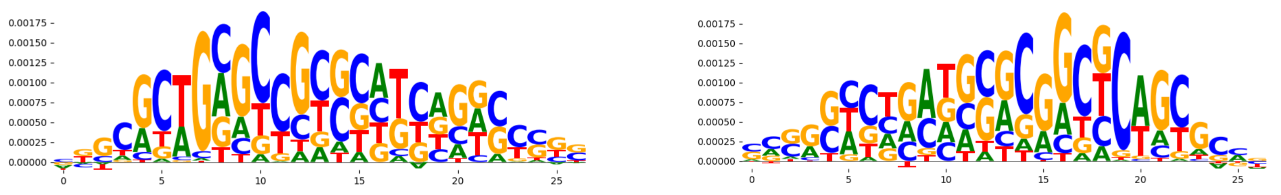	
pos_patterns.pattern_6	61	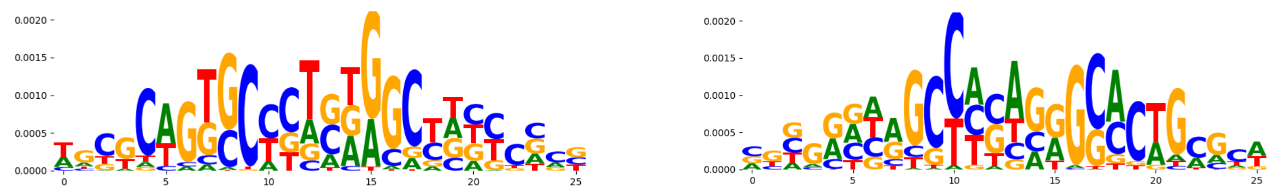	
pos_patterns.pattern_7	55	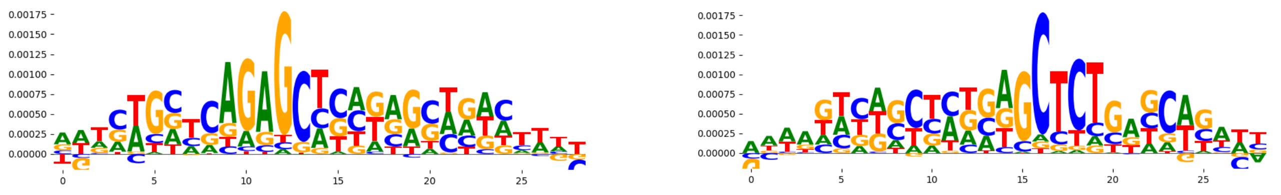	
pos_patterns.pattern_8	45	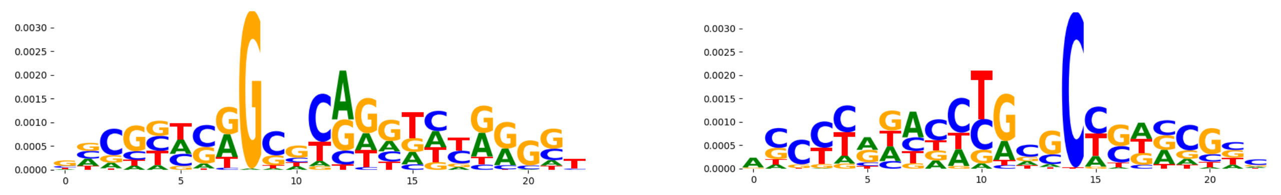	
pos_patterns.pattern_9	45	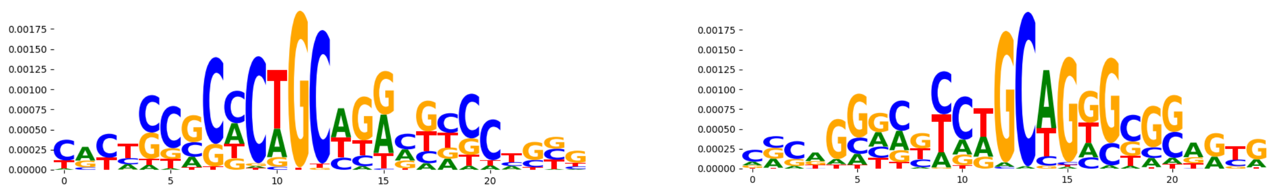	
pos_patterns.pattern_10	44	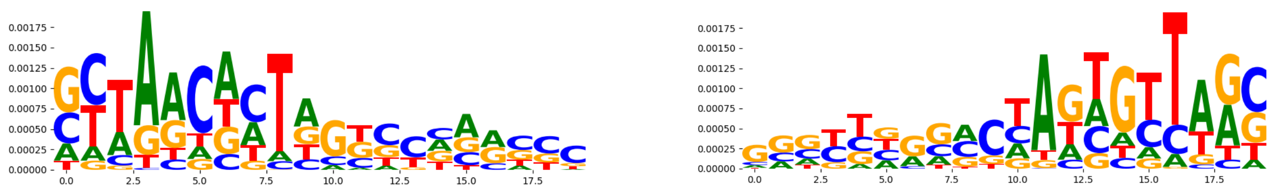	
pos_patterns.pattern_11	28	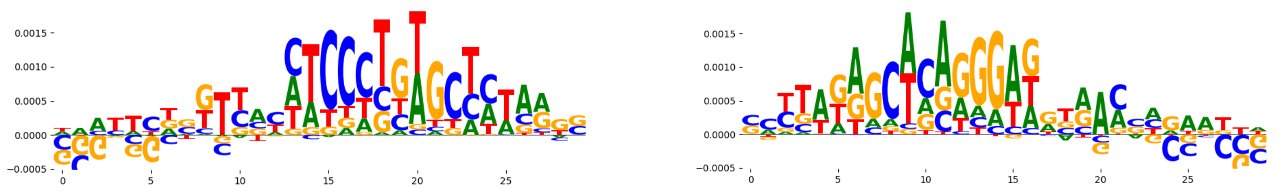	
pos_patterns.pattern_12	22	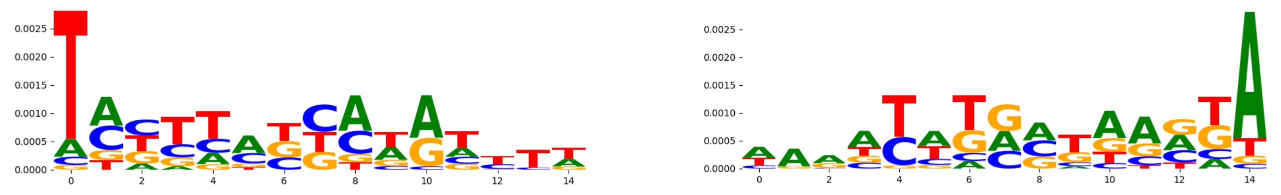	
neg_patterns.pattern_0	2443	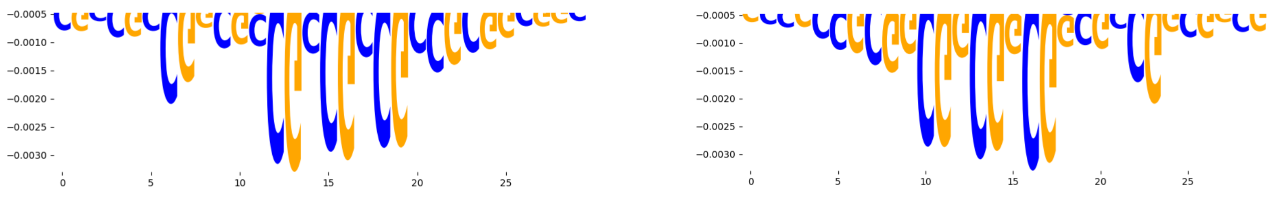	
neg_patterns.pattern_1	2312	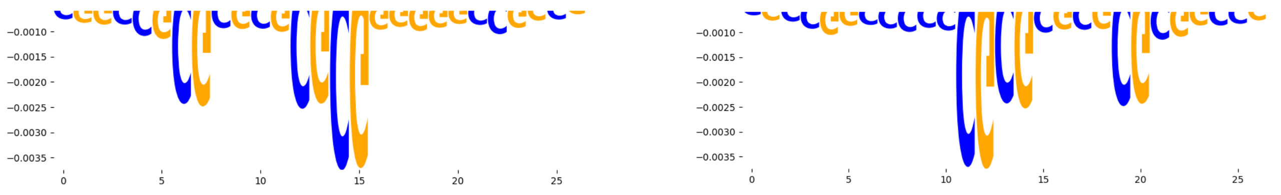	
neg_patterns.pattern_2	1396	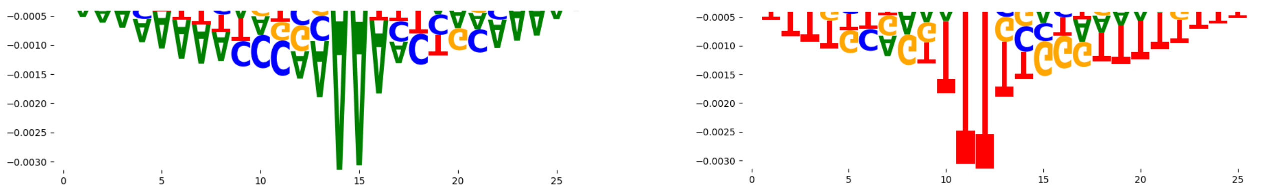	
neg_patterns.pattern_3	1059	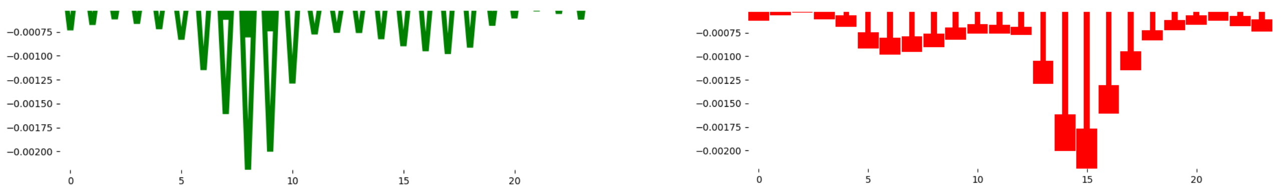	
neg_patterns.pattern_4	953	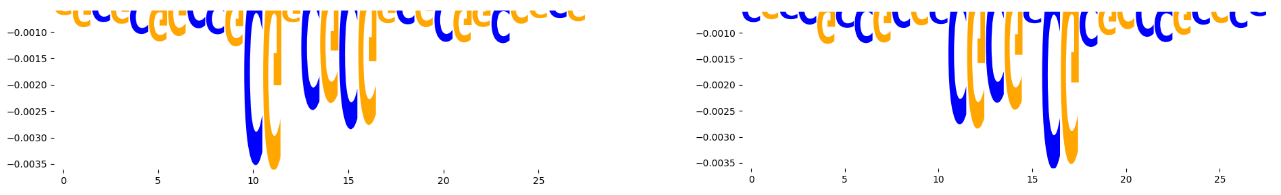	
neg_patterns.pattern_5	788	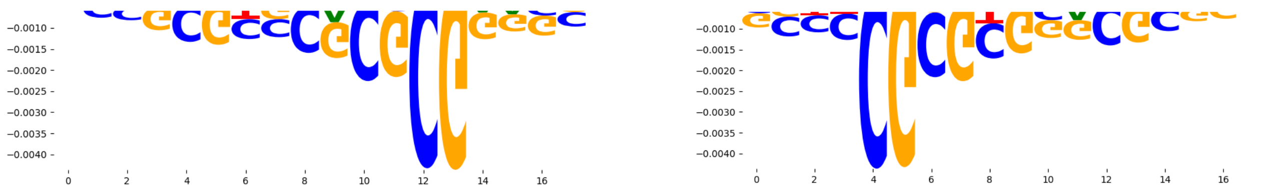	
neg_patterns.pattern_6	737	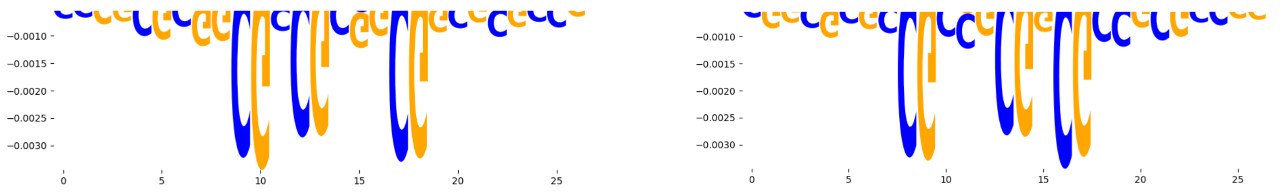	
neg_patterns.pattern_7	440	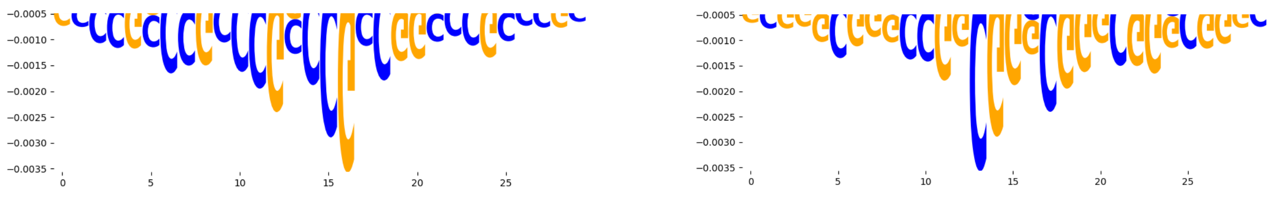	
neg_patterns.pattern_8	400	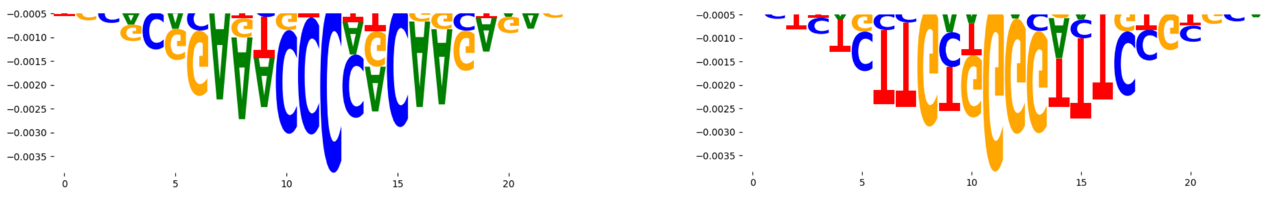	
neg_patterns.pattern_9	373	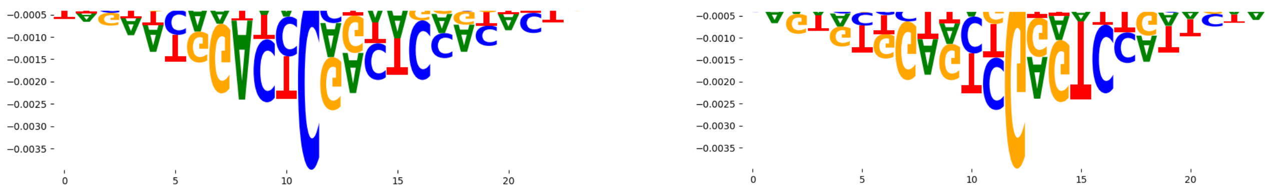	

pattern	num_seqlets	cwm_fwd	cwm_rev
neg_patterns.pattern_10	369	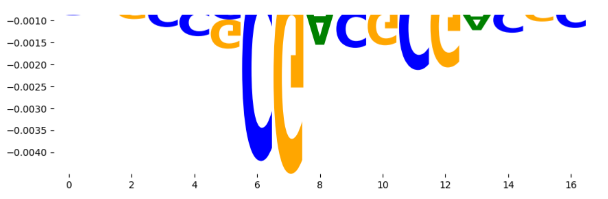	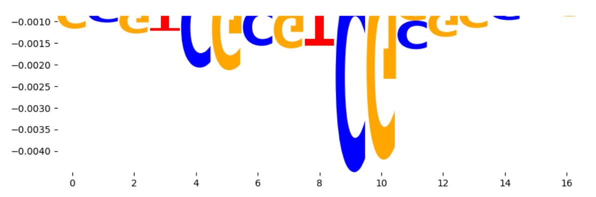
neg_patterns.pattern_11	344	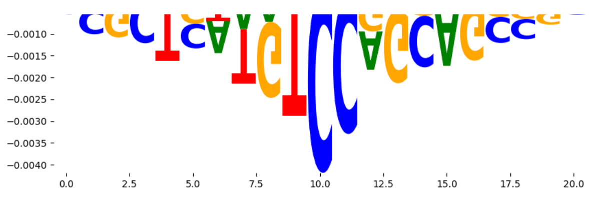	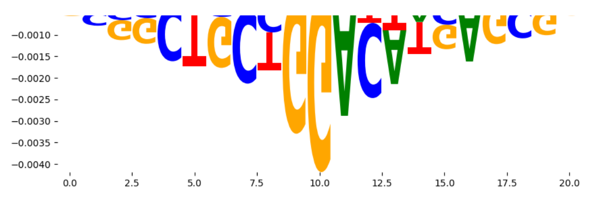
neg_patterns.pattern_12	334	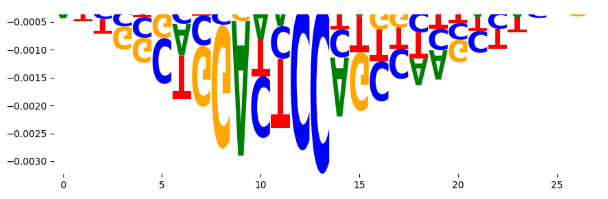	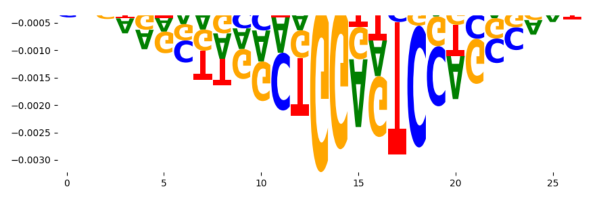
neg_patterns.pattern_13	311	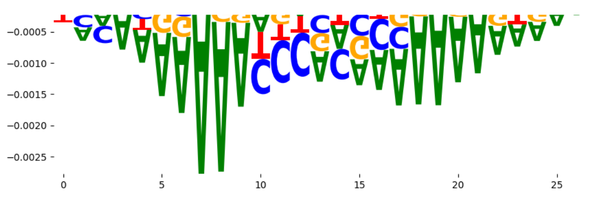	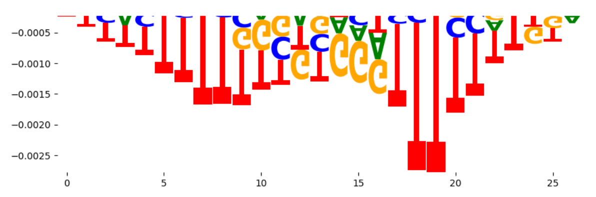
neg_patterns.pattern_14	283	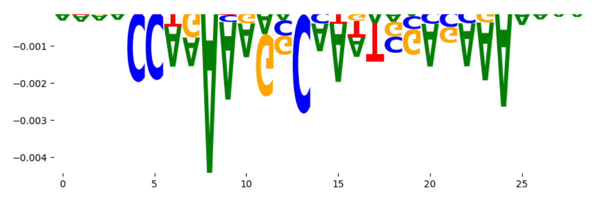	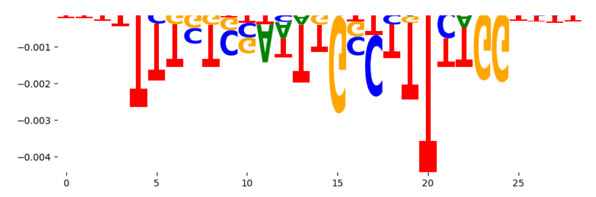
neg_patterns.pattern_15	276	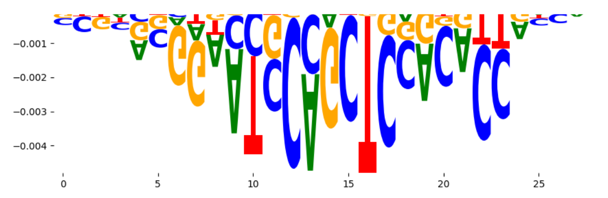	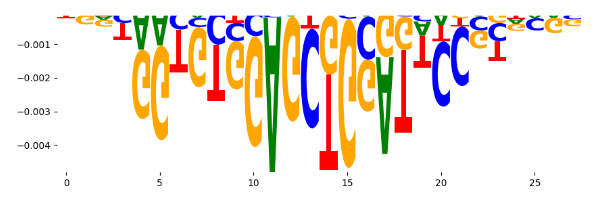
neg_patterns.pattern_16	225	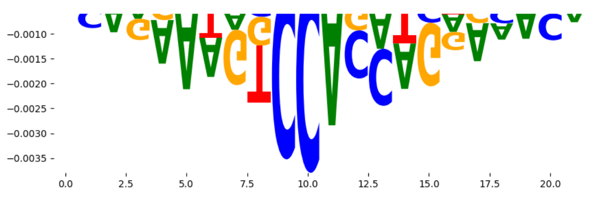	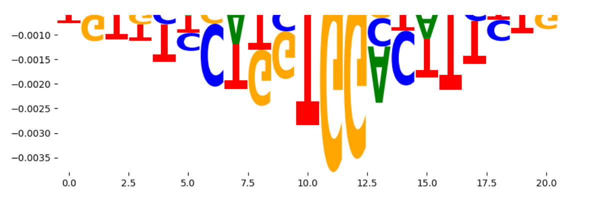
neg_patterns.pattern_17	210	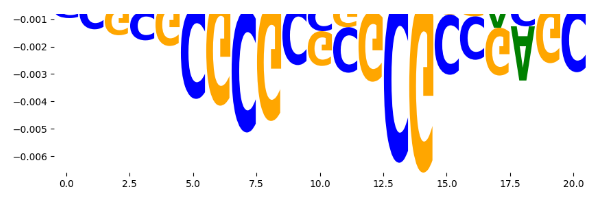	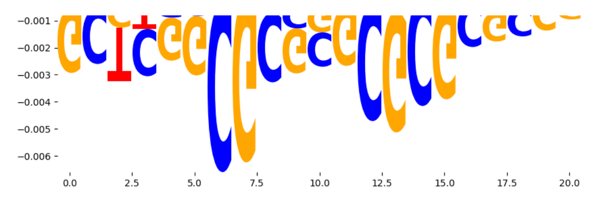
neg_patterns.pattern_18	190	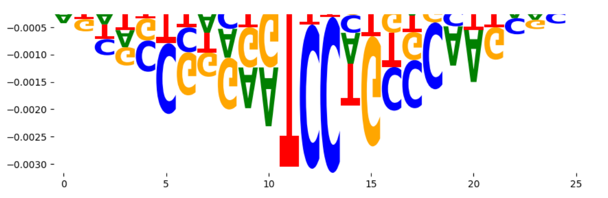	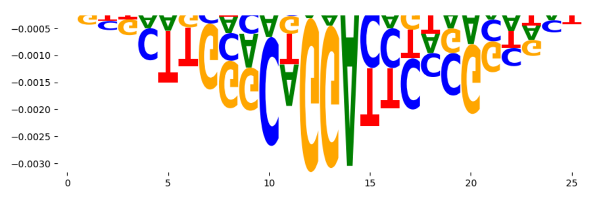
neg_patterns.pattern_19	144	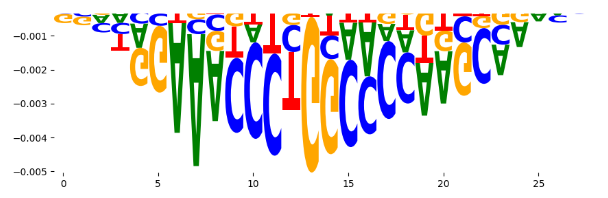	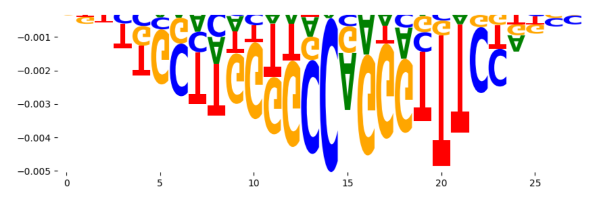
neg_patterns.pattern_20	113	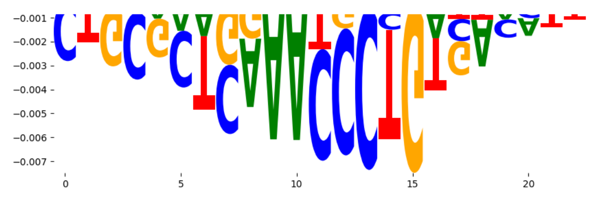	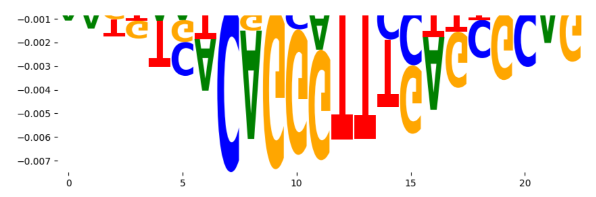
neg_patterns.pattern_21	88	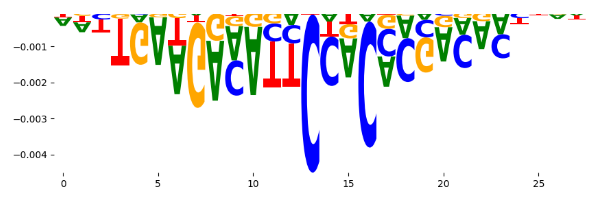	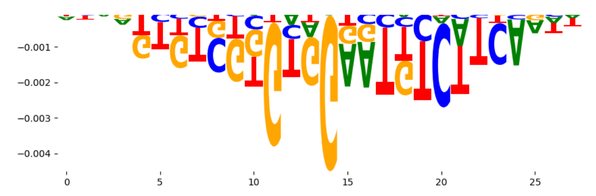
neg_patterns.pattern_22	86	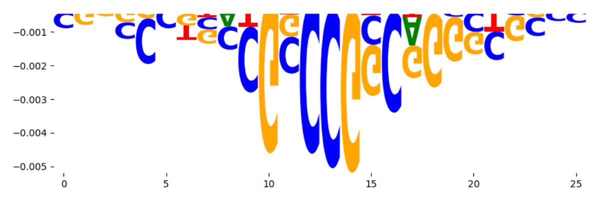	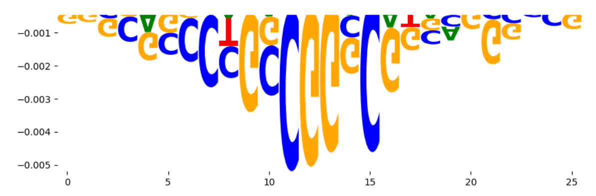
neg_patterns.pattern_23	80	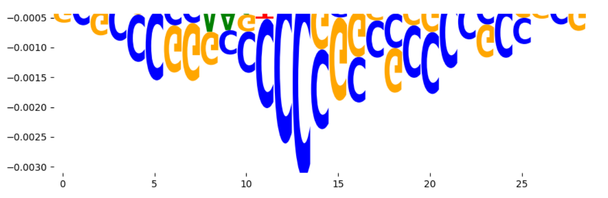	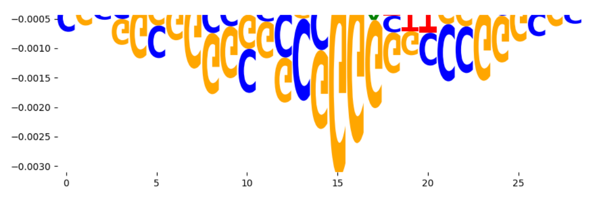
neg_patterns.pattern_24	78	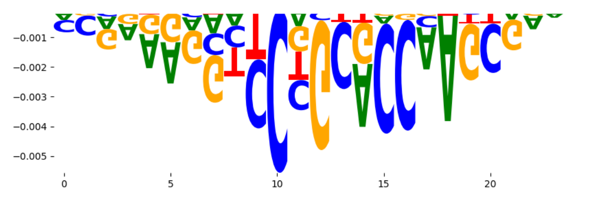	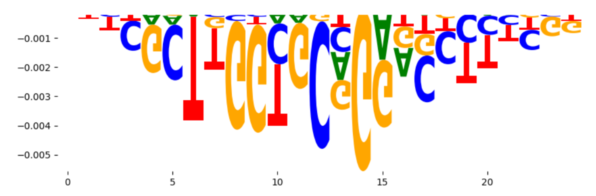
neg_patterns.pattern_25	75	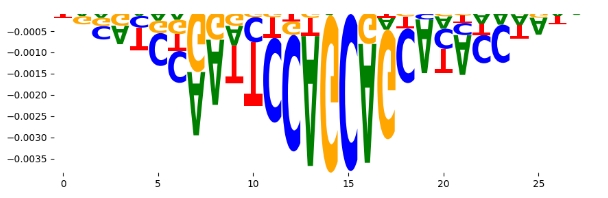	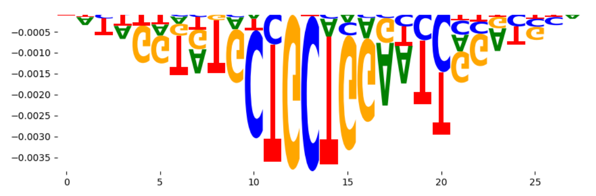
neg_patterns.pattern_26	63	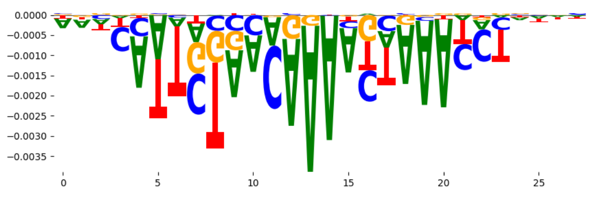	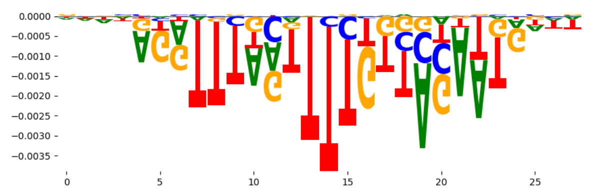
neg_patterns.pattern_27	54	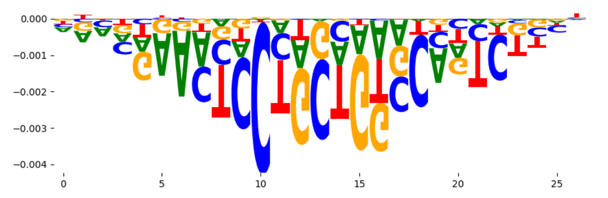	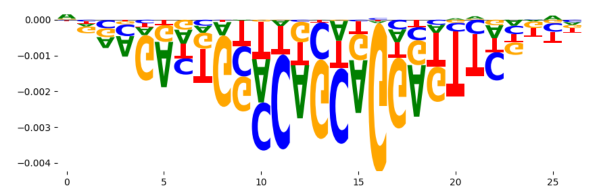
neg_patterns.pattern_28	44	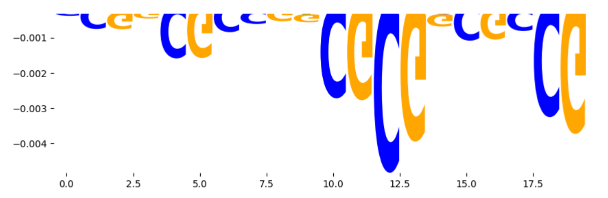	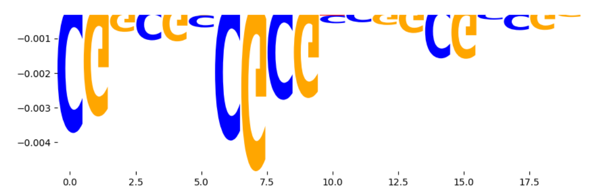
neg_patterns.pattern_29	22	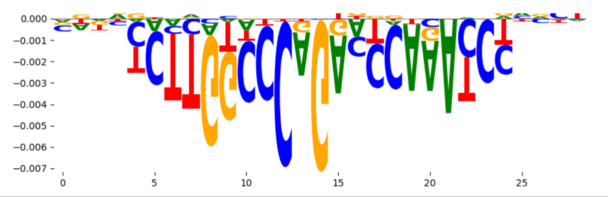	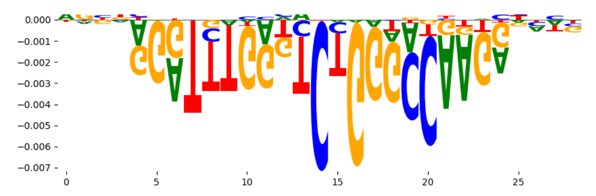
