# Supplementary material for: ChromBPNet: bias factorized, base-resolution deep learning models of chromatin accessibility reveal cis-regulatory sequence syntax, transcription factor footprints and regulatory variants
Source: bioRxiv. 2025 Jan 8:2024.12.25.630221. Preprint. [Version 2] doi: 10.1101/2024.12.25.630221 (PMC11741299; doi:10.1101/2024.12.25.630221)
Supplement: Supplement 4 [file media-4.zip › supplementary_files_3/h1esc_ATAC_bpnet_bias_model/h1esc_ATAC_raw_bpnet_bias_fold1_counts_modisco.pdf]

pattern	num_seqlets	cwm_fwd	cwm_rev	TOMTOM_match	TOMTOM_qval	TOMTOM_match_logo
pos_patterns.pattern_0	7390			ZFX_MOUSE.H11MO.0.B	0.212187	
pos_patterns.pattern_1	2936			SP2_HUMAN.H11MO.0.A	0.188666	
pos_patterns.pattern_2	2406			USF1_MA0093.2	1.000000	
pos_patterns.pattern_3	980			RUNX2_HUMAN.H11MO.0.A	0.664537	
pos_patterns.pattern_4	701			TEAD3_TEA_1	1.000000	
pos_patterns.pattern_5	672			CDX2_MA0465.1	1.000000	
pos_patterns.pattern_6	657			ETS1_ETS_4	0.364602	
pos_patterns.pattern_7	621			TF65_HUMAN.H11MO.0.A	1.000000	
pos_patterns.pattern_8	501			PRDM6_HUMAN.H11MO.0.C	0.098119	
pos_patterns.pattern_9	470			FOXJ3_HUMAN.H11MO.0.A	0.431864	
pos_patterns.pattern_10	417			RREB1_MA0073.1	0.872257	
pos_patterns.pattern_11	303			NFATC2_MA0152.1	1.000000	
pos_patterns.pattern_12	251			HTF4_HUMAN.H11MO.0.A	0.424142	
pos_patterns.pattern_13	132			CTCFL_HUMAN.H11MO.0.A	0.157937	
pos_patterns.pattern_14	108			Hic1.mouse_C2H2_1	1.000000	
pos_patterns.pattern_15	87			FOXB1_forkhead_2	0.414740	
pos_patterns.pattern_16	86			RUNX1_HUMAN.H11MO.0.A	1.000000	
pos_patterns.pattern_17	62			ZIC3_C2H2_1	1.000000	
pos_patterns.pattern_18	49			SP1_HUMAN.H11MO.0.A	0.370660	
pos_patterns.pattern_19	33			KLF12_HUMAN.H11MO.0.C	0.091161	
neg_patterns.pattern_0	39			TN5_2	0.043042	
neg_patterns.pattern_1	38			TN5_1	0.009433	
neg_patterns.pattern_2	36			ZFX_MOUSE.H11MO.0.B	0.003617	
neg_patterns.pattern_3	35			SP2_HUMAN.H11MO.0.A	0.040634	
neg_patterns.pattern_4	33			TN5_2	0.016386	
neg_patterns.pattern_5	28			SP2_HUMAN.H11MO.0.A	0.000033	
neg_patterns.pattern_6	27			Zfx_MA0146.2	0.000031	
neg_patterns.pattern_7	25			TN5_2	0.003152	
