# Supplementary material for: ChromBPNet: bias factorized, base-resolution deep learning models of chromatin accessibility reveal cis-regulatory sequence syntax, transcription factor footprints and regulatory variants
Source: bioRxiv. 2025 Jan 8:2024.12.25.630221. Preprint. [Version 2] doi: 10.1101/2024.12.25.630221 (PMC11741299; doi:10.1101/2024.12.25.630221)
Supplement: Supplement 4 [file media-4.zip › supplementary_files_3/h1esc_ATAC_bpnet_bias_model/h1esc_ATAC_raw_bpnet_bias_fold2_counts_modisco.pdf]

pattern	num_seqlets	cwm_fwd	cwm_rev	TOMTOM_match	TOMTOM_qval	TOMTOM_match_logo
pos_patterns.pattern_0	3323			Zfx_MA0146.2	0.059327	
pos_patterns.pattern_1	3011			SP1_HUMAN.H11MO.0.A	0.024532	
pos_patterns.pattern_2	1553			SP2_HUMAN.H11MO.0.A	0.000013	
pos_patterns.pattern_3	715			ONECUT3_CUT_1	0.048967	
pos_patterns.pattern_4	608			SP2_HUMAN.H11MO.0.A	0.000133	
pos_patterns.pattern_5	590			RUNX3_RUNX_3	1.000000	
pos_patterns.pattern_6	552			SP2_HUMAN.H11MO.0.A	0.000054	
pos_patterns.pattern_7	529			TF7L1_HUMAN.H11MO.0.B	0.246321	
pos_patterns.pattern_8	503			SP1_HUMAN.H11MO.0.A	0.036730	
pos_patterns.pattern_9	460			MXI1_HUMAN.H11MO.0.A	0.161324	
pos_patterns.pattern_10	421			TBX21_TBX_6	1.000000	
pos_patterns.pattern_11	412			SIX4_MOUSE.H11MO.0.C	1.000000	
pos_patterns.pattern_12	403			SP2_HUMAN.H11MO.0.A	0.000067	
pos_patterns.pattern_13	382			SP2_HUMAN.H11MO.0.A	0.000073	
pos_patterns.pattern_14	380			RUNX2_HUMAN.H11MO.0.A	0.880530	
pos_patterns.pattern_15	368			AIRE_HUMAN.H11MO.0.C	1.000000	
pos_patterns.pattern_16	364			COE1_HUMAN.H11MO.0.A	0.589882	
pos_patterns.pattern_17	363			NFATC2_MA0152.1	1.000000	
pos_patterns.pattern_18	257			MYC_MOUSE.H11MO.0.A	0.635173	
pos_patterns.pattern_19	218			SP1_HUMAN.H11MO.0.A	0.000144	
pos_patterns.pattern_20	195			SP2_HUMAN.H11MO.0.A	0.000008	
pos_patterns.pattern_21	174			HOXA13_homeodomain_1	0.060299	
pos_patterns.pattern_22	155			SP1_HUMAN.H11MO.0.A	0.000615	
pos_patterns.pattern_23	137			FOXC1_forkhead_1	0.057777	
pos_patterns.pattern_24	55			CDX2_MOUSE.H11MO.0.A	0.904123	
neg_patterns.pattern_0	79			ZFX_MOUSE.H11MO.0.B	0.000078	
neg_patterns.pattern_1	65			ZFX_MOUSE.H11MO.0.B	0.000580	
neg_patterns.pattern_2	53			ZFX_MOUSE.H11MO.0.B	0.003725	
neg_patterns.pattern_3	42			ZFX_MOUSE.H11MO.0.B	0.004426	

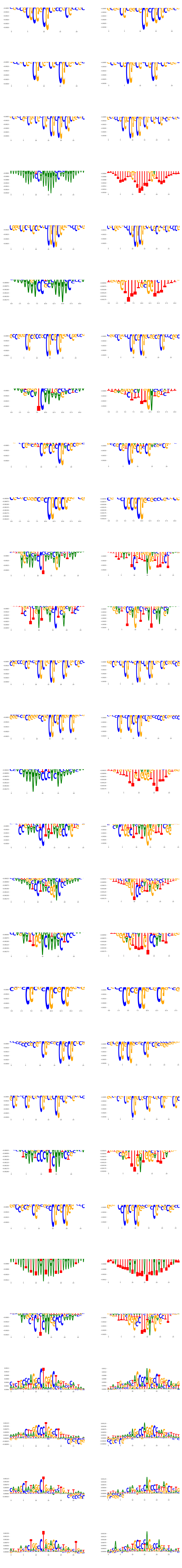

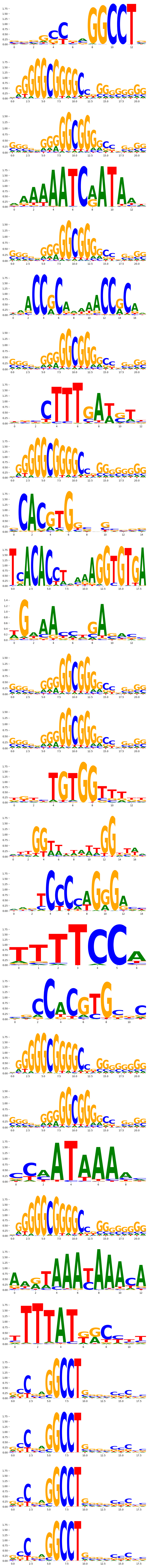

pattern	num_seqlets	cwm_fwd	cwm_rev	TOMTOM_match	TOMTOM_qval	TOMTOM_match_logo
neg_patterns.pattern_4	32	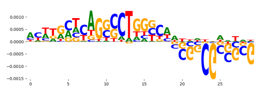	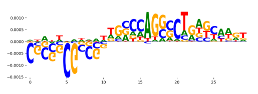	ZFX_MOUSE.H11MO.0.B	0.000003	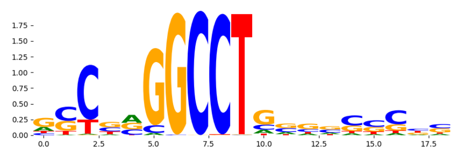
neg_patterns.pattern_5	21	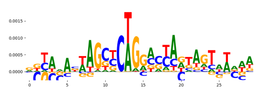	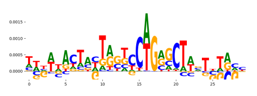	SP1_MOUSE.H11MO.0.A	0.008055	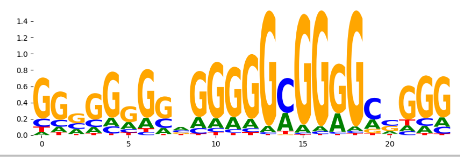
