# Supplementary material for: ChromBPNet: bias factorized, base-resolution deep learning models of chromatin accessibility reveal cis-regulatory sequence syntax, transcription factor footprints and regulatory variants
Source: bioRxiv. 2025 Jan 8:2024.12.25.630221. Preprint. [Version 2] doi: 10.1101/2024.12.25.630221 (PMC11741299; doi:10.1101/2024.12.25.630221)
Supplement: Supplement 4 [file media-4.zip › supplementary_files_3/h1esc_ATAC_bpnet_bias_model/h1esc_ATAC_raw_bpnet_bias_fold2_profile_modisco.pdf]

pattern	num_seqlets	cwm_fwd	cwm_rev	TOMTOM_match	TOMTOM_qval	TOMTOM_match_logo
pos_patterns.pattern_0	9131			TN5_1	1.168880e-08	
pos_patterns.pattern_1	7828			TN5_2	1.386410e-03	
pos_patterns.pattern_2	4650			KLF4_MA0039.3	2.854600e-01	
pos_patterns.pattern_3	4395			TN5_2	3.829520e-17	
pos_patterns.pattern_4	1439			TN5_3	3.179490e-07	
pos_patterns.pattern_5	1076			TN5_3	7.153540e-04	
pos_patterns.pattern_6	956			TN5_1	3.790070e-05	
pos_patterns.pattern_7	938			TN5_3	9.590350e-05	
pos_patterns.pattern_8	799			TN5_3	4.644990e-13	
pos_patterns.pattern_9	579			TN5_1	1.004600e-04	
pos_patterns.pattern_10	541			TN5_7	9.796010e-08	
pos_patterns.pattern_11	454			TN5_3	3.088290e-04	
pos_patterns.pattern_12	260			TN5_3	2.955170e-02	
pos_patterns.pattern_13	202			PRDM6_HUMAN.H11MO.0.C	4.868180e-02	
pos_patterns.pattern_14	122			HOXC12_homeodomain_1	1.313010e-01	
pos_patterns.pattern_15	69			PRDM6_HUMAN.H11MO.0.C	1.183380e-01	
