# Supplementary material for: ChromBPNet: bias factorized, base-resolution deep learning models of chromatin accessibility reveal cis-regulatory sequence syntax, transcription factor footprints and regulatory variants
Source: bioRxiv. 2025 Jan 8:2024.12.25.630221. Preprint. [Version 2] doi: 10.1101/2024.12.25.630221 (PMC11741299; doi:10.1101/2024.12.25.630221)
Supplement: Supplement 4 [file media-4.zip › supplementary_files_3/h1esc_ATAC_bpnet_bias_model/h1esc_ATAC_raw_bpnet_bias_fold3_counts_modisco.pdf]

pattern	num_seqlets	cwm_fwd	cwm_rev	TOMTOM_match	TOMTOM_qval	TOMTOM_match_logo
pos_patterns.pattern_0	2473			MYC_MOUSE.H11MO.0.A	1.000000e+00	
pos_patterns.pattern_1	2120			SP1_HUMAN.H11MO.0.A	6.795050e-05	
pos_patterns.pattern_2	1705			SP1_HUMAN.H11MO.0.A	5.390600e-04	
pos_patterns.pattern_3	1368			ZFX_MOUSE.H11MO.0.B	3.409470e-04	
pos_patterns.pattern_4	1245			NHLH1_bHLH_1	4.422980e-01	
pos_patterns.pattern_5	1018			SP2_HUMAN.H11MO.0.A	1.649610e-07	
pos_patterns.pattern_6	838			SP1_HUMAN.H11MO.0.A	5.603110e-06	
pos_patterns.pattern_7	745			SP1_HUMAN.H11MO.0.A	1.504240e-03	
pos_patterns.pattern_8	615			SP1_HUMAN.H11MO.0.A	2.491780e-05	
pos_patterns.pattern_9	582			SP1_HUMAN.H11MO.0.A	1.743420e-04	
pos_patterns.pattern_10	579			SP1_HUMAN.H11MO.0.A	3.389240e-04	
pos_patterns.pattern_11	557			ZNF713_C2H2_1	3.559350e-01	
pos_patterns.pattern_12	540			NFATC1_NFAT_1	3.030710e-01	
pos_patterns.pattern_13	525			SP1_HUMAN.H11MO.0.A	2.034130e-05	
pos_patterns.pattern_14	485			NFATC1_MA0624.1	1.000000e+00	
pos_patterns.pattern_15	471			PRDM6_HUMAN.H11MO.0.C	9.969850e-02	
pos_patterns.pattern_16	444			MBD2_HUMAN.H11MO.0.B	2.238740e-01	
pos_patterns.pattern_17	442			SP1_HUMAN.H11MO.0.A	3.798030e-04	
pos_patterns.pattern_18	438			NFAC3_HUMAN.H11MO.0.B	2.416170e-01	
pos_patterns.pattern_19	397			SP2_HUMAN.H11MO.0.A	3.810530e-06	
pos_patterns.pattern_20	336			SP2_HUMAN.H11MO.0.A	4.026590e-06	
pos_patterns.pattern_21	334			SP1_HUMAN.H11MO.0.A	1.962430e-03	
pos_patterns.pattern_22	299			SP1_HUMAN.H11MO.0.A	6.606220e-03	
pos_patterns.pattern_23	249			SP1_MOUSE.H11MO.0.A	2.804140e-04	
pos_patterns.pattern_24	243			SP2_HUMAN.H11MO.0.A	1.207740e-01	
pos_patterns.pattern_25	223			SP2_HUMAN.H11MO.0.A	6.907690e-04	
pos_patterns.pattern_26	213			SP1_HUMAN.H11MO.0.A	2.891790e-02	
pos_patterns.pattern_27	212			Hes1_MA1099.1	1.000000e+00	
pos_patterns.pattern_28	202			PBX3_MA1114.1	1.000000e+00	

pattern	num_seqlets	cwm_fwd	cwm_rev	TOMTOM_match	TOMTOM_qval	TOMTOM_match_logo
pos_patterns.pattern_29	193	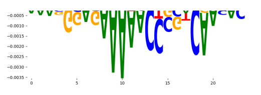	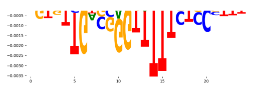	ZN274_HUMAN.H11MO.0.A	1.000000e+00	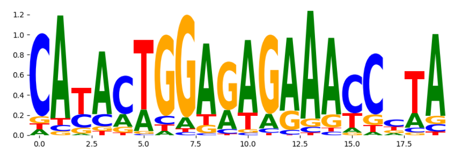
pos_patterns.pattern_30	184	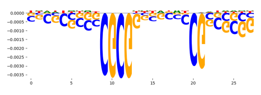	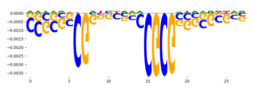	SP2_HUMAN.H11MO.0.A	4.056290e-04	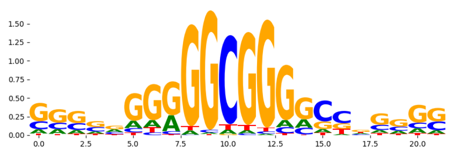
pos_patterns.pattern_31	171	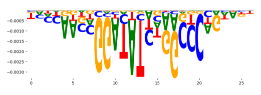	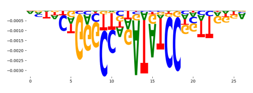	FLI1_ETS_2	1.000000e+00	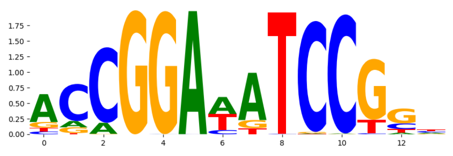
pos_patterns.pattern_32	159	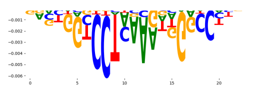	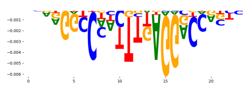	HOXC12_MA0906.1	5.105430e-01	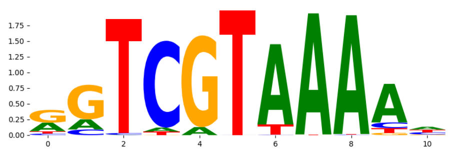
pos_patterns.pattern_33	150	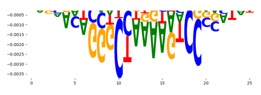	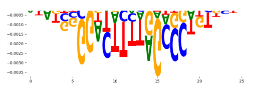	Hoxc9_MA0485.1	6.949450e-01	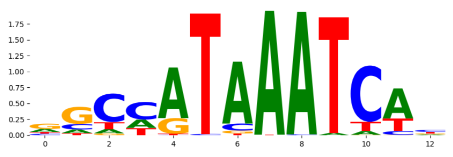
pos_patterns.pattern_34	130	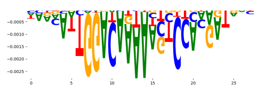	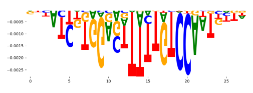	THA11_MOUSE.H11MO.0.B	6.762340e-01	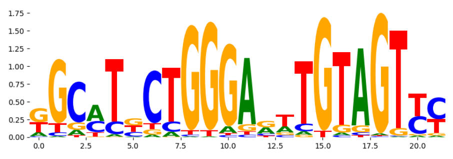
pos_patterns.pattern_35	64	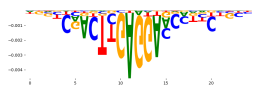	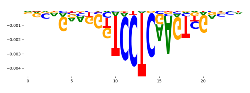	RXRA+VDR_MA0074.1	1.000000e+00	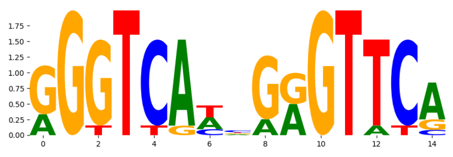
pos_patterns.pattern_36	56	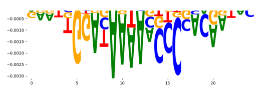	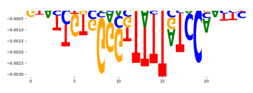	ZN274_HUMAN.H11MO.0.A	1.000000e+00	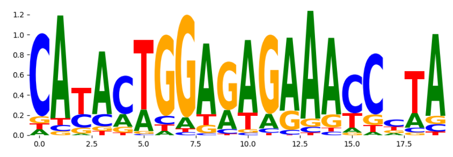
pos_patterns.pattern_37	54	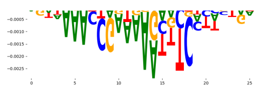	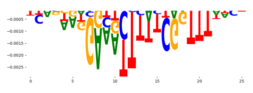	IRF7_HUMAN.H11MO.0.C	9.048840e-01	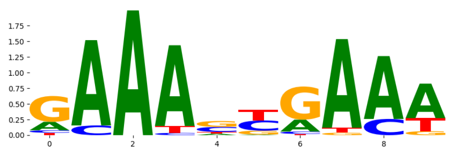
pos_patterns.pattern_38	34	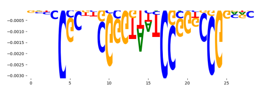	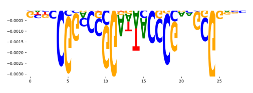	SP1_MOUSE.H11MO.0.A	2.611970e-01	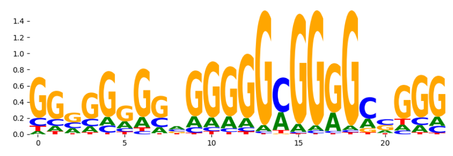
neg_patterns.pattern_0	54	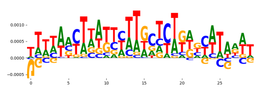	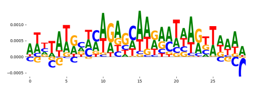	ZN341_HUMAN.H11MO.0.C	9.750740e-03	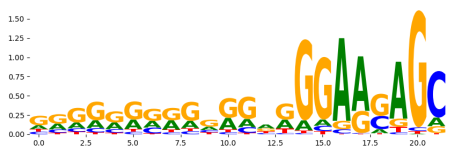
neg_patterns.pattern_1	40	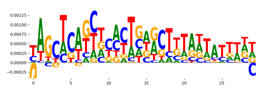	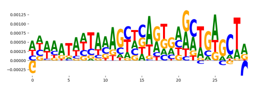	SP1_MA0079.3	4.076930e-02	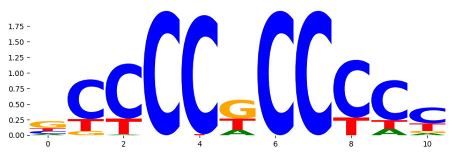
neg_patterns.pattern_2	35	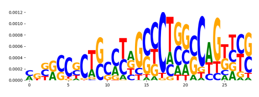	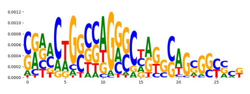	TN5_1	6.171160e-03	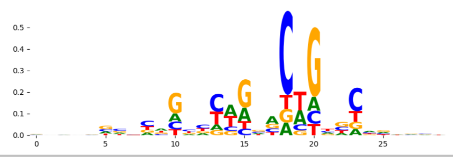
