# Supplementary material for: ChromBPNet: bias factorized, base-resolution deep learning models of chromatin accessibility reveal cis-regulatory sequence syntax, transcription factor footprints and regulatory variants
Source: bioRxiv. 2025 Jan 8:2024.12.25.630221. Preprint. [Version 2] doi: 10.1101/2024.12.25.630221 (PMC11741299; doi:10.1101/2024.12.25.630221)
Supplement: Supplement 4 [file media-4.zip › supplementary_files_3/h1esc_ATAC_bpnet_bias_model/h1esc_ATAC_raw_bpnet_bias_fold3_profile_modisco.pdf]

pattern	num_seqlets	cwm_fwd	cwm_rev	TOMTOM_match	TOMTOM_qval	TOMTOM_match_logo
pos_patterns.pattern_0	8650	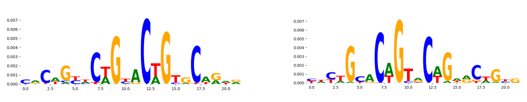		TN5_1	1.589380e-08	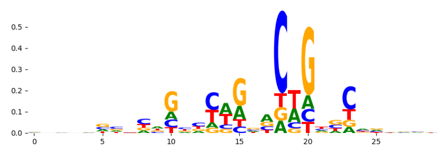
pos_patterns.pattern_1	5671	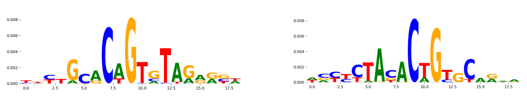		TN5_8	4.565650e-02	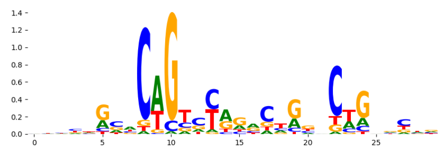
pos_patterns.pattern_2	4599	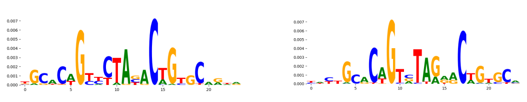		TN5_1	9.031440e-06	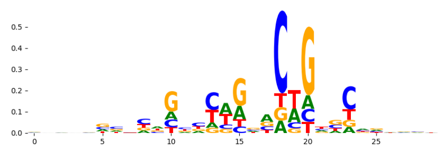
pos_patterns.pattern_3	4067	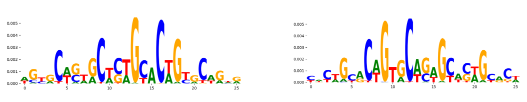		TN5_2	1.602500e-10	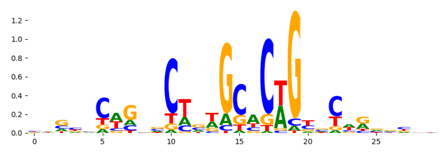
pos_patterns.pattern_4	3103	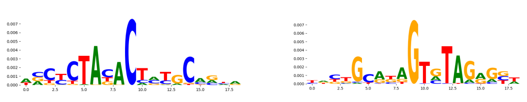		KLF4_MA0039.3	1.511040e-01	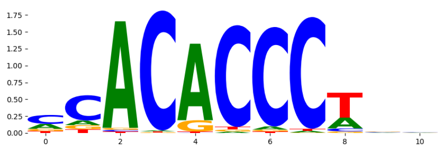
pos_patterns.pattern_5	2835	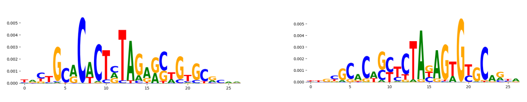		TN5_3	2.571590e-18	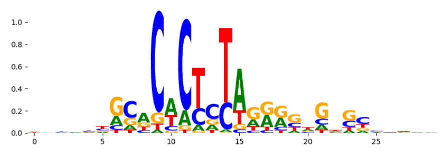
pos_patterns.pattern_6	2306	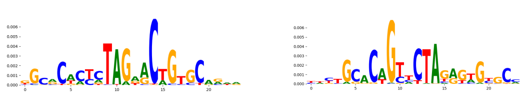		TN5_3	2.587690e-05	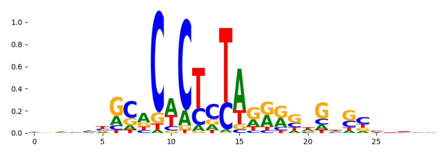
pos_patterns.pattern_7	759	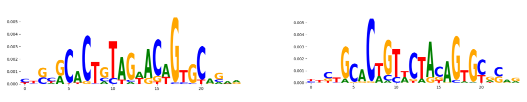		TN5_7	1.719890e-06	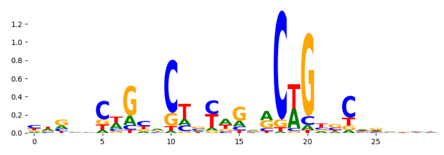
pos_patterns.pattern_8	651	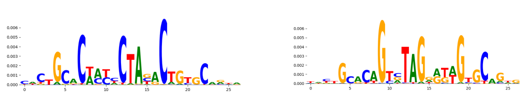		TN5_3	1.172350e-04	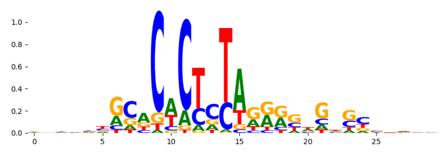
pos_patterns.pattern_9	538	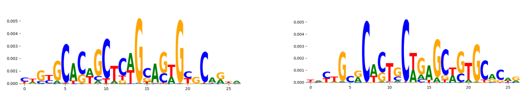		TN5_3	1.437450e-05	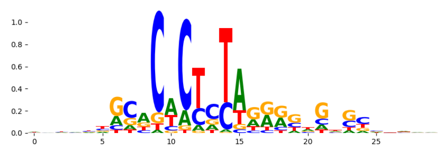
pos_patterns.pattern_10	320	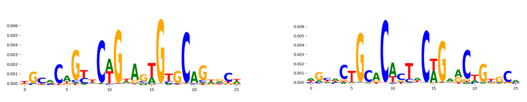		TN5_1	3.460300e-06	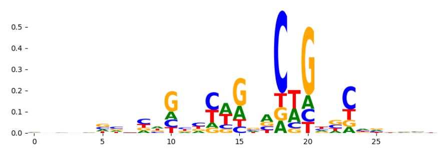
pos_patterns.pattern_11	253	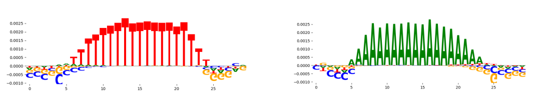		PRDM6_HUMAN.H11MO.0.C	6.236420e-02	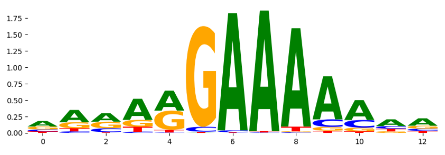
pos_patterns.pattern_12	126	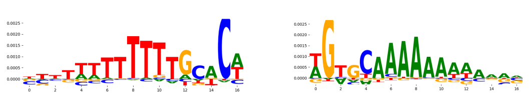		Hoxc10.mouse_homeodomain_2	2.178580e-01	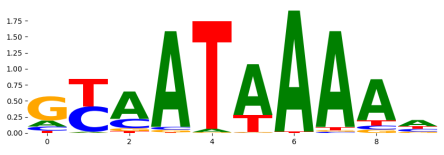
pos_patterns.pattern_13	82	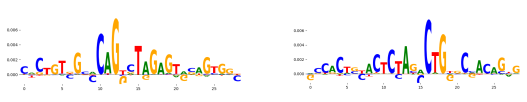		TN5_6	9.169900e-17	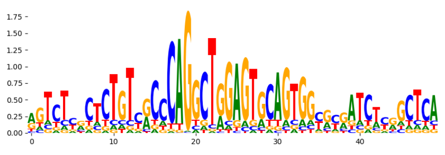
pos_patterns.pattern_14	68	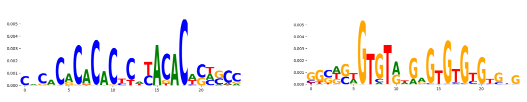		EGR2_HUMAN.H11MO.0.A	1.000000e+00	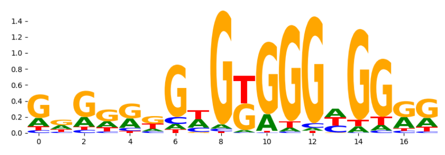
pos_patterns.pattern_15	65	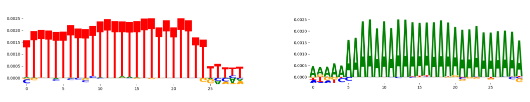		PRDM6_HUMAN.H11MO.0.C	1.124100e-01	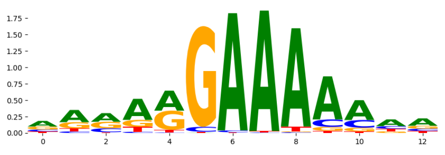
neg_patterns.pattern_0	21	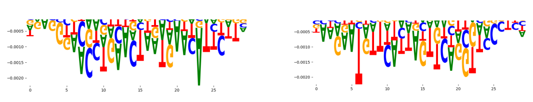		POU3F3_POU_3	2.582030e-03	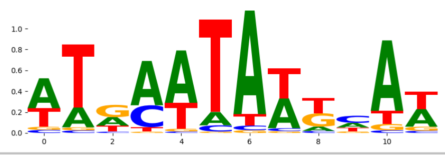
