# Supplementary material for: ChromBPNet: bias factorized, base-resolution deep learning models of chromatin accessibility reveal cis-regulatory sequence syntax, transcription factor footprints and regulatory variants
Source: bioRxiv. 2025 Jan 8:2024.12.25.630221. Preprint. [Version 2] doi: 10.1101/2024.12.25.630221 (PMC11741299; doi:10.1101/2024.12.25.630221)
Supplement: Supplement 4 [file media-4.zip › supplementary_files_3/h1esc_ATAC_bpnet_bias_model/h1esc_ATAC_raw_bpnet_bias_fold0_profile_modisco.pdf]

pattern	num_seqlets	cwm_fwd	cwm_rev	TOMTOM_match	TOMTOM_qval	TOMTOM_match_logo
pos_patterns.pattern_0	8300			TN5_1	2.528420e-10	
pos_patterns.pattern_1	6387			TN5_4	2.016040e-02	
pos_patterns.pattern_2	4160			TN5_1	8.705690e-08	
pos_patterns.pattern_3	4034			TN5_2	7.075140e-12	
pos_patterns.pattern_4	3402			KLF4_MA0039.3	9.051140e-02	
pos_patterns.pattern_5	1151			TN5_3	1.227700e-03	
pos_patterns.pattern_6	1056			TN5_3	2.715650e-07	
pos_patterns.pattern_7	982			TN5_7	3.901690e-08	
pos_patterns.pattern_8	842			TN5_3	3.273350e-04	
pos_patterns.pattern_9	828			TN5_3	6.993060e-08	
pos_patterns.pattern_10	755			TN5_3	1.695140e-06	
pos_patterns.pattern_11	625			TN5_3	3.409020e-05	
pos_patterns.pattern_12	533			TN5_1	1.179610e-04	
pos_patterns.pattern_13	261			PRDM6_HUMAN.H11MO.0.C	4.827240e-02	
pos_patterns.pattern_14	165			EGR2_HUMAN.H11MO.0.A	6.844730e-01	
pos_patterns.pattern_15	111			PRDM6_HUMAN.H11MO.0.C	1.041710e-01	
pos_patterns.pattern_16	72			TN5_6	3.254370e-17	
pos_patterns.pattern_17	61			Hoxc10.mouse_homeodomain_2	3.858620e-01	
pos_patterns.pattern_18	28			ZN331_HUMAN.H11MO.0.C	1.000000e+00	
pos_patterns.pattern_19	23			FOXJ3_HUMAN.H11MO.0.A	1.000000e+00	
pos_patterns.pattern_20	22			TN5_2	6.189360e-02	
pos_patterns.pattern_21	20			TN5_3	1.065650e-02	
neg_patterns.pattern_0	27			ZNF384_MA1125.1	1.786380e-03	
neg_patterns.pattern_1	24			PRDM6_HUMAN.H11MO.0.C	2.132810e-02	
neg_patterns.pattern_2	23			SRY_HUMAN.H11MO.0.B	1.000000e+00	
