# Supplementary material for: ChromBPNet: bias factorized, base-resolution deep learning models of chromatin accessibility reveal cis-regulatory sequence syntax, transcription factor footprints and regulatory variants
Source: bioRxiv. 2025 Jan 8:2024.12.25.630221. Preprint. [Version 2] doi: 10.1101/2024.12.25.630221 (PMC11741299; doi:10.1101/2024.12.25.630221)
Supplement: Supplement 4 [file media-4.zip › supplementary_files_3/h1esc_ATAC_bpnet_bias_model/h1esc_ATAC_raw_bpnet_bias_fold1_profile_modisco.pdf]

pattern	num_seqlets	cwm_fwd	cwm_rev	TOMTOM_match	TOMTOM_qval	TOMTOM_match_logo
pos_patterns.pattern_0	8441			TN5_1	1.554260e-09	
pos_patterns.pattern_1	5578			TN5_8	4.614530e-02	
pos_patterns.pattern_2	4741			TN5_1	4.544490e-06	
pos_patterns.pattern_3	3948			TN5_2	1.604460e-19	
pos_patterns.pattern_4	3643			KLF4_MA0039.3	3.157090e-01	
pos_patterns.pattern_5	1089			TN5_3	3.862770e-05	
pos_patterns.pattern_6	1037			TN5_3	1.330290e-03	
pos_patterns.pattern_7	1036			TN5_3	3.190970e-08	
pos_patterns.pattern_8	893			TN5_3	7.765820e-09	
pos_patterns.pattern_9	764			TN5_3	6.422400e-04	
pos_patterns.pattern_10	747			TN5_3	3.787640e-12	
pos_patterns.pattern_11	459			TN5_1	7.324630e-05	
pos_patterns.pattern_12	354			TN5_7	6.799020e-02	
pos_patterns.pattern_13	286			PRDM6_HUMAN.H11MO.0.C	5.478950e-02	
pos_patterns.pattern_14	126			MEF2C_MA0497.1	5.342060e-01	
pos_patterns.pattern_15	67			TN5_8	1.000000e+00	
pos_patterns.pattern_16	56			VEZF1_HUMAN.H11MO.0.C	7.340360e-02	
pos_patterns.pattern_17	54			RREB1_MA0073.1	1.000000e+00	
neg_patterns.pattern_0	22			CPEB1_RRM_1	1.770700e-01	
neg_patterns.pattern_1	20			ZNF384_MA1125.1	1.035220e-03	
