# Supplementary material for: ChromBPNet: bias factorized, base-resolution deep learning models of chromatin accessibility reveal cis-regulatory sequence syntax, transcription factor footprints and regulatory variants
Source: bioRxiv. 2025 Jan 8:2024.12.25.630221. Preprint. [Version 2] doi: 10.1101/2024.12.25.630221 (PMC11741299; doi:10.1101/2024.12.25.630221)
Supplement: Supplement 4 [file media-4.zip › supplementary_files_3/h1esc_ATAC_bpnet_bias_model/h1esc_ATAC_raw_bpnet_bias_fold4_profile_modisco.pdf]

pattern	num_seqlets	cwm_fwd	cwm_rev
pos_patterns.pattern_0	8668	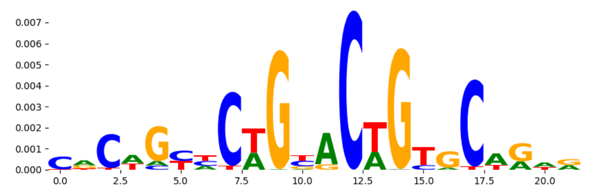	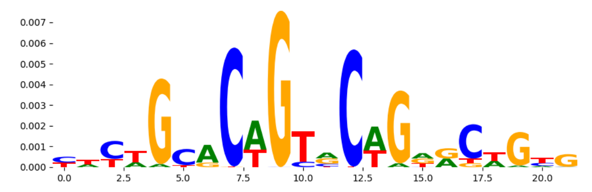
pos_patterns.pattern_1	5608	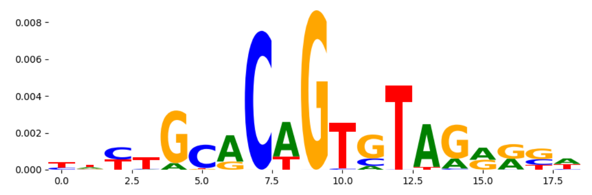	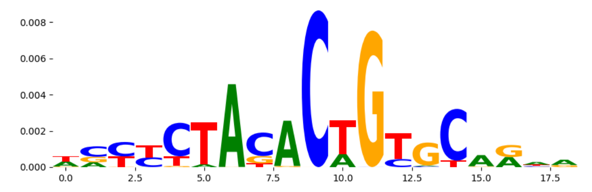
pos_patterns.pattern_2	4298	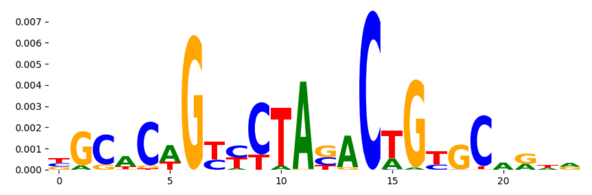	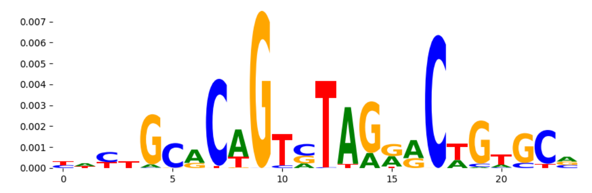
pos_patterns.pattern_3	4027	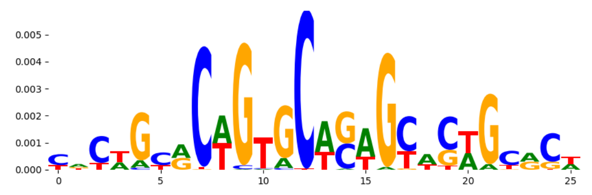	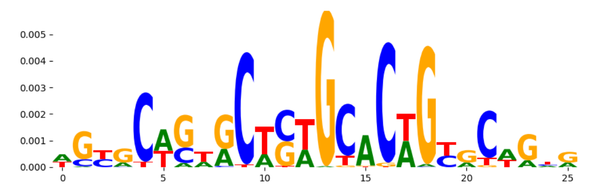
pos_patterns.pattern_4	2732	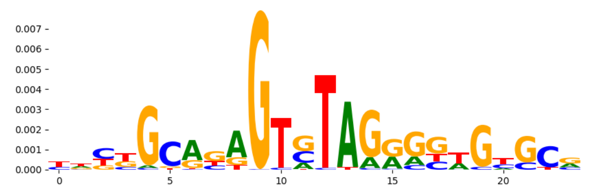	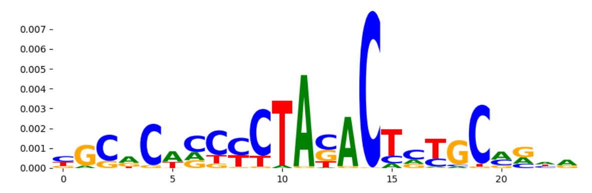
pos_patterns.pattern_5	2347	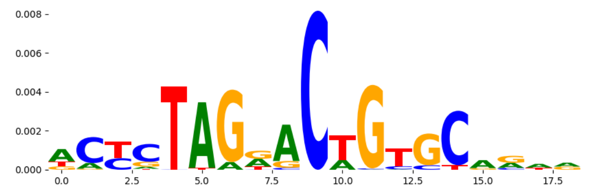	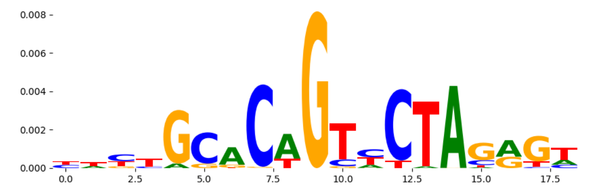
pos_patterns.pattern_6	1908	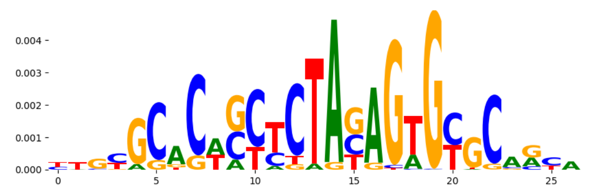	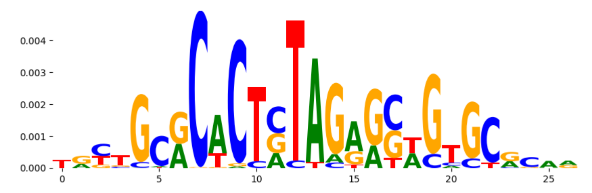
pos_patterns.pattern_7	900	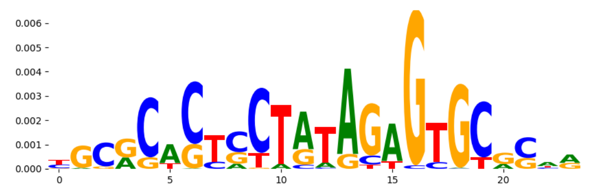	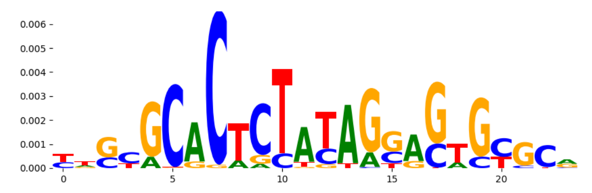
pos_patterns.pattern_8	780	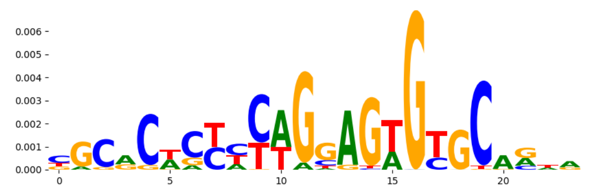	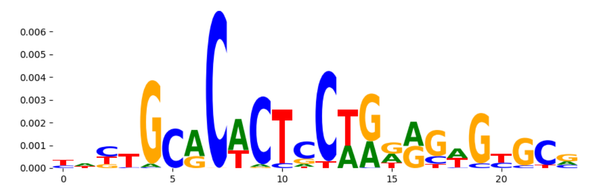
pos_patterns.pattern_9	724	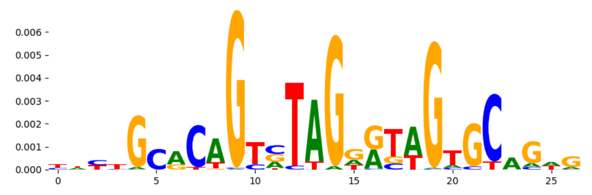	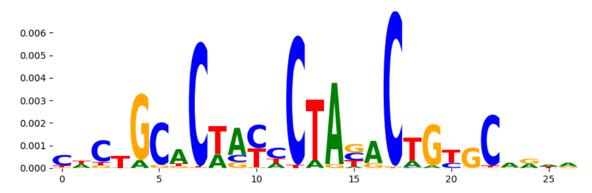
pos_patterns.pattern_10	669	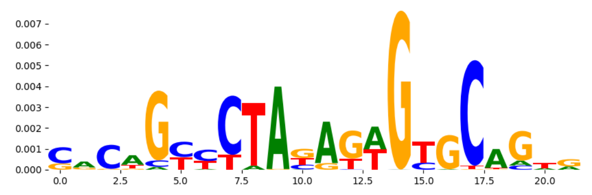	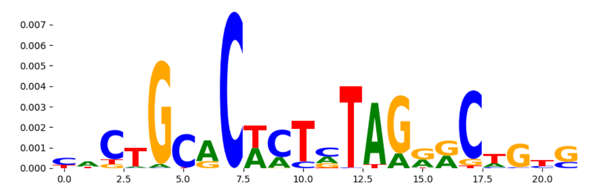
pos_patterns.pattern_11	260	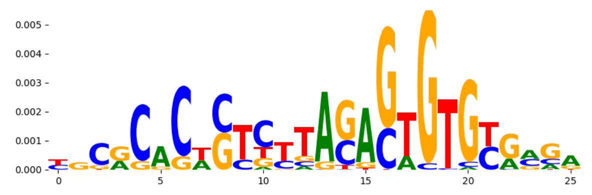	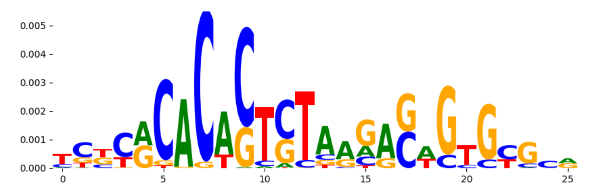
pos_patterns.pattern_12	231	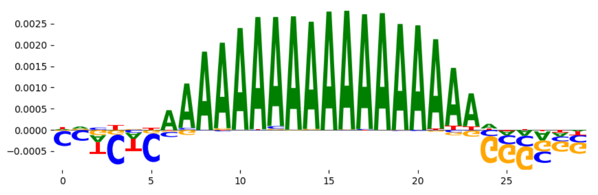	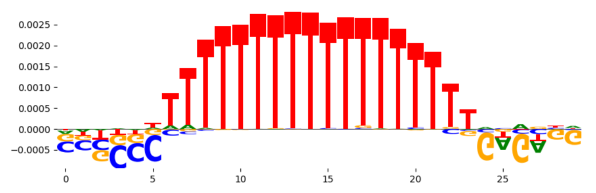
pos_patterns.pattern_13	104	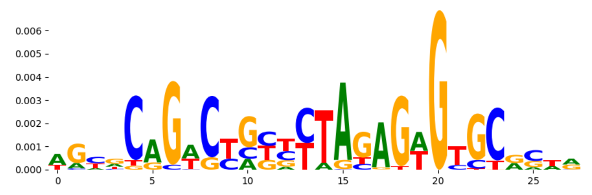	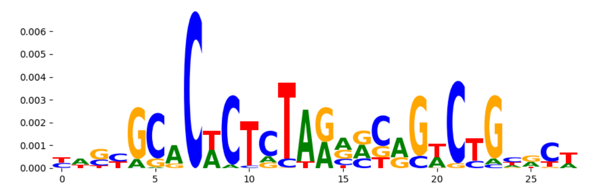
pos_patterns.pattern_14	93	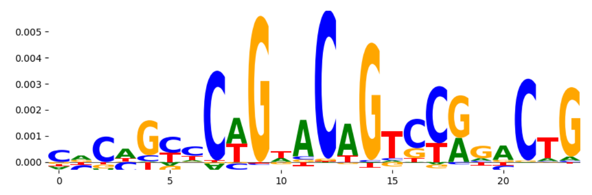	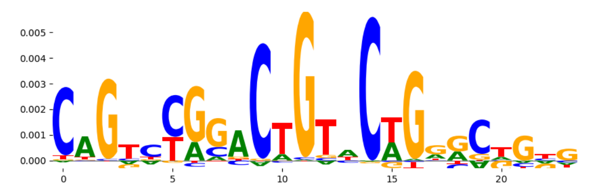
pos_patterns.pattern_15	77	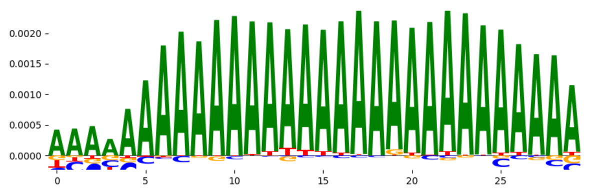	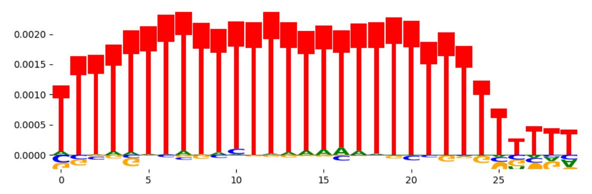
pos_patterns.pattern_16	54	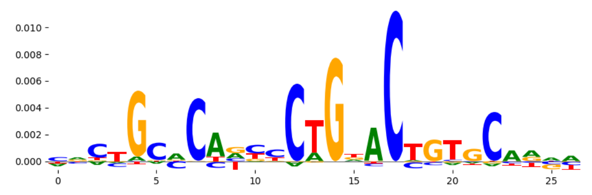	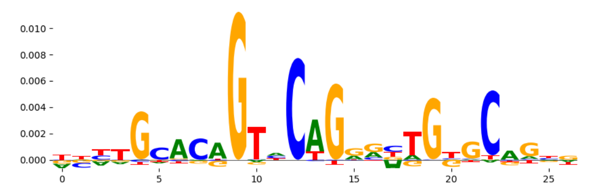
pos_patterns.pattern_17	52	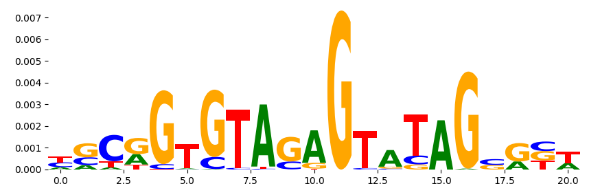	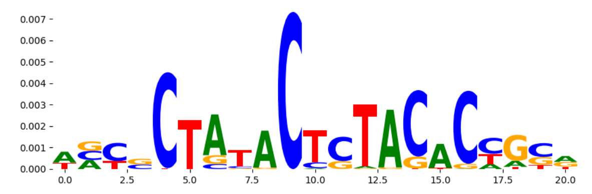
pos_patterns.pattern_18	23	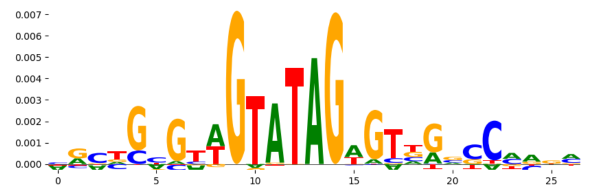	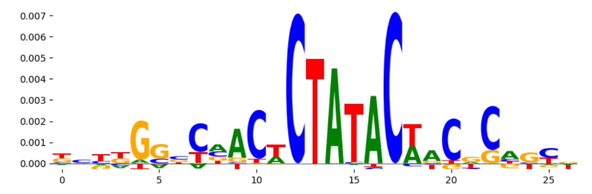
pos_patterns.pattern_19	20	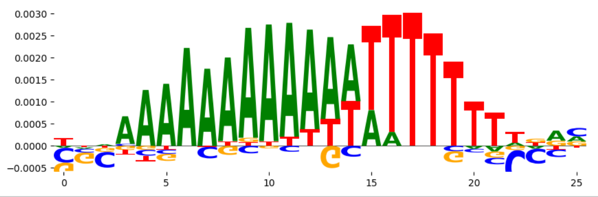	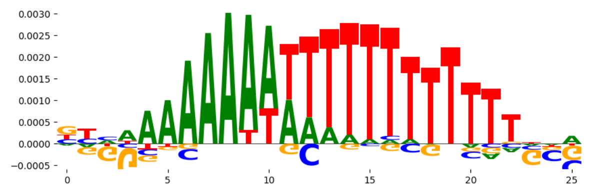
