# Supplementary material for: ChromBPNet: bias factorized, base-resolution deep learning models of chromatin accessibility reveal cis-regulatory sequence syntax, transcription factor footprints and regulatory variants
Source: bioRxiv. 2025 Jan 8:2024.12.25.630221. Preprint. [Version 2] doi: 10.1101/2024.12.25.630221 (PMC11741299; doi:10.1101/2024.12.25.630221)
Supplement: Supplement 4 [file media-4.zip › supplementary_files_3/h1esc_ATAC_bpnet_bias_model/h1esc_ATAC_raw_bpnet_bias_fold0_counts_modisco.pdf]

pattern	num_seqlets	cwm_fwd	cwm_rev	TOMTOM_match	TOMTOM_qval	TOMTOM_match_logo
pos_patterns.pattern_0	3688			Zfx_MA0146.2	0.417986	
pos_patterns.pattern_1	3194			SP3_HUMAN.H11MO.0.B	0.005538	
pos_patterns.pattern_2	2719			ZFX_MOUSE.H11MO.0.B	0.007030	
pos_patterns.pattern_3	706			NR2C2_MOUSE.H11MO.0.A	0.687804	
pos_patterns.pattern_4	663			DNASE_4	0.298947	
pos_patterns.pattern_5	560			SP2_HUMAN.H11MO.0.A	0.132577	
pos_patterns.pattern_6	540			NFATC3_MA0625.1	0.982323	
pos_patterns.pattern_7	533			PRDM6_HUMAN.H11MO.0.C	0.190641	
pos_patterns.pattern_8	528			ZNF384_MA1125.1	0.059846	
pos_patterns.pattern_9	480			HXA13_HUMAN.H11MO.0.C	0.773619	
pos_patterns.pattern_10	405			Z354A_HUMAN.H11MO.0.C	1.000000	
pos_patterns.pattern_11	320			NFAC3_HUMAN.H11MO.0.B	0.030386	
pos_patterns.pattern_12	288			CLOCK_HUMAN.H11MO.0.C	0.502019	
pos_patterns.pattern_13	274			ZIM3_HUMAN.H11MO.0.C	0.956574	
pos_patterns.pattern_14	174			NaN	NaN	
pos_patterns.pattern_15	164			IRF5_IRF_1	1.000000	
pos_patterns.pattern_16	115			ZBT18_HUMAN.H11MO.0.C	1.000000	
pos_patterns.pattern_17	103			NKX2-8_MA0673.1	1.000000	
pos_patterns.pattern_18	73			HIC1_HUMAN.H11MO.0.C	0.932626	
pos_patterns.pattern_19	59			MTF1_C2H2_1	0.817915	
pos_patterns.pattern_20	55			SMCA5_MOUSE.H11MO.0.C	0.018338	
pos_patterns.pattern_21	43			PKNOX2_MA0783.1	0.999997	
pos_patterns.pattern_22	39			HOXD13_MA0909.1	1.000000	
pos_patterns.pattern_23	37			ELK1_ETS_3	1.000000	
neg_patterns.pattern_0	42			SP1_HUMAN.H11MO.0.A	0.006678	
neg_patterns.pattern_1	41			TN5_7	0.133522	
neg_patterns.pattern_2	27			SP1_MOUSE.H11MO.0.A	0.000020	
neg_patterns.pattern_3	22			Zfx_MA0146.2	0.204657	
