# Supplementary material for: ChromBPNet: bias factorized, base-resolution deep learning models of chromatin accessibility reveal cis-regulatory sequence syntax, transcription factor footprints and regulatory variants
Source: bioRxiv. 2025 Jan 8:2024.12.25.630221. Preprint. [Version 2] doi: 10.1101/2024.12.25.630221 (PMC11741299; doi:10.1101/2024.12.25.630221)
Supplement: Supplement 4 [file media-4.zip › supplementary_files_3/h1esc_DNASE_bpnet_bias_model/h1esc_DNASE_raw_bpnet_bias_fold4_counts_modisco.pdf]

pattern	num_seqlets	cwm_fwd	cwm_rev	TOMTOM_match	TOMTOM_qval	TOMTOM_match_logo
pos_patterns.pattern_0	6559			CTCFL_HUMAN.H11MO.0.A	8.883550e-02	
pos_patterns.pattern_1	4210			ERR2_HUMAN.H11MO.0.A	1.000000e+00	
pos_patterns.pattern_2	1806			PATZ1_HUMAN.H11MO.0.C	7.385580e-08	
pos_patterns.pattern_3	1326			MYOD1_HUMAN.H11MO.0.A	2.152600e-02	
pos_patterns.pattern_4	780			FOXC1_HUMAN.H11MO.0.C	6.688190e-01	
pos_patterns.pattern_5	663			IKZF1_HUMAN.H11MO.0.C	9.924120e-01	
pos_patterns.pattern_6	446			MEF2B_HUMAN.H11MO.0.A	2.918930e-01	
pos_patterns.pattern_7	391			RARG_HUMAN.H11MO.0.B	6.618960e-01	
pos_patterns.pattern_8	358			ZNF384_MA1125.1	3.570010e-01	
pos_patterns.pattern_9	300			NaN	NaN	
pos_patterns.pattern_10	262			PRDM6_HUMAN.H11MO.0.C	6.703000e-02	
pos_patterns.pattern_11	221			ZN770_HUMAN.H11MO.0.C	1.589580e-08	
pos_patterns.pattern_12	207			PRDM6_HUMAN.H11MO.0.C	1.030610e-01	
pos_patterns.pattern_13	180			FOS_HUMAN.H11MO.0.A	5.536570e-01	
pos_patterns.pattern_14	92			ZN770_HUMAN.H11MO.0.C	2.129290e-07	
pos_patterns.pattern_15	60			IRF3_HUMAN.H11MO.0.B	6.059640e-03	
pos_patterns.pattern_16	50			PRDM6_HUMAN.H11MO.0.C	3.859810e-02	
neg_patterns.pattern_0	39			ZNF384_MA1125.1	1.707810e-01	
neg_patterns.pattern_1	32			ZNF384_MA1125.1	4.840300e-02	
neg_patterns.pattern_2	29			FOXD2_forkhead_1	1.671690e-01	
