# Supplementary material for: ChromBPNet: bias factorized, base-resolution deep learning models of chromatin accessibility reveal cis-regulatory sequence syntax, transcription factor footprints and regulatory variants
Source: bioRxiv. 2025 Jan 8:2024.12.25.630221. Preprint. [Version 2] doi: 10.1101/2024.12.25.630221 (PMC11741299; doi:10.1101/2024.12.25.630221)
Supplement: Supplement 4 [file media-4.zip › supplementary_files_3/h1esc_DNASE_bpnet_bias_model/h1esc_DNASE_raw_bpnet_bias_fold0_profile_modisco.pdf]

pattern	num_seqlets	cwm_fwd	cwm_rev	TOMTOM_match	TOMTOM_qval	TOMTOM_match_logo
pos_patterns.pattern_0	10825			CTCFL_HUMAN.H11MO.0.A	7.392560e-06	
pos_patterns.pattern_1	2181			FOXB1_forkhead_2	3.328230e-01	
pos_patterns.pattern_2	2160			MEF2B_HUMAN.H11MO.0.A	3.026800e-01	
pos_patterns.pattern_3	1837			TN5_6	8.274170e-02	
pos_patterns.pattern_4	1759			ZN770_HUMAN.H11MO.0.C	4.594410e-06	
pos_patterns.pattern_5	1657			ZN770_HUMAN.H11MO.0.C	5.683580e-08	
pos_patterns.pattern_6	1460			TN5_6	5.142120e-35	
pos_patterns.pattern_7	1383			SP3_HUMAN.H11MO.0.B	4.393010e-02	
pos_patterns.pattern_8	1323			SP1_MOUSE.H11MO.0.A	7.659200e-10	
pos_patterns.pattern_9	1302			TFAP2A_AP2_4	5.691270e-01	
pos_patterns.pattern_10	1296			ZNF384_MA1125.1	8.307610e-02	
pos_patterns.pattern_11	1145			DNASE_5	1.146680e-07	
pos_patterns.pattern_12	731			BARX1_MOUSE.H11MO.0.C	7.613750e-01	
pos_patterns.pattern_13	699			ZN770_HUMAN.H11MO.0.C	4.987530e-08	
pos_patterns.pattern_14	595			NFIC_MA0161.2	1.000000e+00	
pos_patterns.pattern_15	559			DNASE_5	1.451000e-10	
pos_patterns.pattern_16	516			DNASE_5	6.550720e-16	
pos_patterns.pattern_17	464			MNX1_MA0707.1	8.780570e-01	
pos_patterns.pattern_18	403			DNASE_5	2.485640e-15	
pos_patterns.pattern_19	290			ZN770_HUMAN.H11MO.0.C	8.127400e-01	
pos_patterns.pattern_20	260			IKZF1_HUMAN.H11MO.0.C	1.000000e+00	
pos_patterns.pattern_21	219			STAT1_MOUSE.H11MO.0.A	7.357560e-02	
pos_patterns.pattern_22	79			DNASE_5	1.000000e+00	
pos_patterns.pattern_23	49			MAFB_HUMAN.H11MO.0.B	1.000000e+00	
pos_patterns.pattern_24	27			RREB1_MA0073.1	1.000000e+00	
