# Supplementary material for: ChromBPNet: bias factorized, base-resolution deep learning models of chromatin accessibility reveal cis-regulatory sequence syntax, transcription factor footprints and regulatory variants
Source: bioRxiv. 2025 Jan 8:2024.12.25.630221. Preprint. [Version 2] doi: 10.1101/2024.12.25.630221 (PMC11741299; doi:10.1101/2024.12.25.630221)
Supplement: Supplement 4 [file media-4.zip › supplementary_files_3/h1esc_DNASE_bpnet_bias_model/h1esc_DNASE_raw_bpnet_bias_fold1_counts_modisco.pdf]

pattern	num_seqlets	cwm_fwd	cwm_rev	TOMTOM_match	TOMTOM_qval	TOMTOM_match_logo
pos_patterns.pattern_0	4619			CTCFL_MA1102.1	9.079450e-02	
pos_patterns.pattern_1	4094			NFIC_HUMAN.H11MO.0.A	1.339040e-01	
pos_patterns.pattern_2	3056			SP1_HUMAN.H11MO.0.A	6.217670e-03	
pos_patterns.pattern_3	3030			SP2_HUMAN.H11MO.0.A	1.035430e-11	
pos_patterns.pattern_4	885			DNASE_1	1.100790e-01	
pos_patterns.pattern_5	471			DNASE_5	1.939700e-09	
pos_patterns.pattern_6	354			MEF2B_HUMAN.H11MO.0.A	2.400440e-01	
pos_patterns.pattern_7	315			RARG_HUMAN.H11MO.0.B	9.598430e-01	
pos_patterns.pattern_8	311			CPEB1_RRM_1	3.448310e-01	
pos_patterns.pattern_9	210			ZN250_HUMAN.H11MO.0.C	1.000000e+00	
pos_patterns.pattern_10	203			VDR_MA0693.2	1.000000e+00	
pos_patterns.pattern_11	196			ZNF384_MA1125.1	3.157230e-02	
pos_patterns.pattern_12	163			ZN250_HUMAN.H11MO.0.C	1.000000e+00	
pos_patterns.pattern_13	158			ITF2_HUMAN.H11MO.0.C	1.000000e+00	
pos_patterns.pattern_14	155			PRDM6_HUMAN.H11MO.0.C	1.070280e-01	
pos_patterns.pattern_15	136			EGR1_MOUSE.H11MO.0.A	6.046960e-01	
pos_patterns.pattern_16	54			NaN	NaN	
neg_patterns.pattern_0	29			SP3_HUMAN.H11MO.0.B	3.641440e-03	
neg_patterns.pattern_1	22			CPEB1_RRM_1	5.270600e-02	
