# Supplementary material for: ChromBPNet: bias factorized, base-resolution deep learning models of chromatin accessibility reveal cis-regulatory sequence syntax, transcription factor footprints and regulatory variants
Source: bioRxiv. 2025 Jan 8:2024.12.25.630221. Preprint. [Version 2] doi: 10.1101/2024.12.25.630221 (PMC11741299; doi:10.1101/2024.12.25.630221)
Supplement: Supplement 4 [file media-4.zip › supplementary_files_3/h1esc_DNASE_bpnet_bias_model/h1esc_DNASE_raw_bpnet_bias_fold2_counts_modisco.pdf]

pattern	num_seqlets	cwm_fwd	cwm_rev	TOMTOM_match	TOMTOM_qval	TOMTOM_match_logo
pos_patterns.pattern_0	4380			ASCL1_MA1100.1	1.554320e-03	
pos_patterns.pattern_1	3784			SP1_MOUSE.H11MO.0.A	1.540340e-01	
pos_patterns.pattern_2	2636			CTCFL_MOUSE.H11MO.0.A	1.619870e-02	
pos_patterns.pattern_3	2361			SP1_MOUSE.H11MO.0.A	2.901770e-11	
pos_patterns.pattern_4	1020			ERR2_HUMAN.H11MO.0.A	1.000000e+00	
pos_patterns.pattern_5	488			DNASE_1	1.000000e+00	
pos_patterns.pattern_6	472			ZNF384_MA1125.1	6.900920e-02	
pos_patterns.pattern_7	468			DNASE_2	2.089980e-01	
pos_patterns.pattern_8	374			MEF2B_HUMAN.H11MO.0.A	1.652510e-01	
pos_patterns.pattern_9	310			IKZF1_HUMAN.H11MO.0.C	1.000000e+00	
pos_patterns.pattern_10	303			ZN770_HUMAN.H11MO.0.C	6.116850e-01	
pos_patterns.pattern_11	271			NKX3-2_MA0122.2	6.596690e-01	
pos_patterns.pattern_12	189			DNASE_5	1.542050e-08	
pos_patterns.pattern_13	128			RREB1_MA0073.1	6.059040e-02	
pos_patterns.pattern_14	102			ZN770_HUMAN.H11MO.0.C	2.338700e-07	
pos_patterns.pattern_15	78			ZN770_HUMAN.H11MO.0.C	2.630420e-05	
pos_patterns.pattern_16	48			FOSL1_MA0477.1	4.572410e-01	
pos_patterns.pattern_17	32			KLF15_HUMAN.H11MO.0.A	1.355650e-01	
pos_patterns.pattern_18	23			NKX28_HUMAN.H11MO.0.C	1.000000e+00	
neg_patterns.pattern_0	50			ZN281_MOUSE.H11MO.0.A	3.419830e-02	
neg_patterns.pattern_1	26			ZNF384_MA1125.1	3.666560e-03	
neg_patterns.pattern_2	23			ZNF384_MA1125.1	1.929630e-02	
