# Supplementary material for: ChromBPNet: bias factorized, base-resolution deep learning models of chromatin accessibility reveal cis-regulatory sequence syntax, transcription factor footprints and regulatory variants
Source: bioRxiv. 2025 Jan 8:2024.12.25.630221. Preprint. [Version 2] doi: 10.1101/2024.12.25.630221 (PMC11741299; doi:10.1101/2024.12.25.630221)
Supplement: Supplement 4 [file media-4.zip › supplementary_files_3/h1esc_DNASE_bpnet_bias_model/h1esc_DNASE_raw_bpnet_bias_fold3_counts_modisco.pdf]

pattern	num_seqlets	cwm_fwd	cwm_rev	TOMTOM_match	TOMTOM_qval	TOMTOM_match_logo
pos_patterns.pattern_0	4651			CTCFL_MOUSE.H11MO.0.A	1.554180e-03	
pos_patterns.pattern_1	3653			PLAG1_MA0163.1	5.117070e-03	
pos_patterns.pattern_2	2604			SP1_MOUSE.H11MO.0.A	8.787310e-08	
pos_patterns.pattern_3	2312			ZFX_MOUSE.H11MO.0.B	8.513520e-05	
pos_patterns.pattern_4	1836			DNASE_1	1.000000e+00	
pos_patterns.pattern_5	469			IKZF1_HUMAN.H11MO.0.C	1.000000e+00	
pos_patterns.pattern_6	384			DNASE_2	1.810630e-01	
pos_patterns.pattern_7	364			PRDM6_HUMAN.H11MO.0.C	9.201670e-02	
pos_patterns.pattern_8	361			DNASE_2	2.128850e-01	
pos_patterns.pattern_9	352			DNASE_5	2.843870e-09	
pos_patterns.pattern_10	292			RARG_HUMAN.H11MO.0.B	9.315280e-01	
pos_patterns.pattern_11	228			FOXJ3_HUMAN.H11MO.0.A	3.502850e-01	
pos_patterns.pattern_12	122			ZN770_HUMAN.H11MO.0.C	9.429680e-03	
pos_patterns.pattern_13	86			EGR1_MOUSE.H11MO.0.A	5.061020e-01	
pos_patterns.pattern_14	55			Zfx_MA0146.2	9.806720e-01	
pos_patterns.pattern_15	45			TN5_6	1.845540e-21	
