# Supplementary material for: ChromBPNet: bias factorized, base-resolution deep learning models of chromatin accessibility reveal cis-regulatory sequence syntax, transcription factor footprints and regulatory variants
Source: bioRxiv. 2025 Jan 8:2024.12.25.630221. Preprint. [Version 2] doi: 10.1101/2024.12.25.630221 (PMC11741299; doi:10.1101/2024.12.25.630221)
Supplement: Supplement 5 [file media-5.zip › supplementary_files_4/2_naked_dna_DNASE_bias_model/fig4c_naked_dna_DNASE_raw_bias_model_counts_modisco.pdf]

| pattern | num_seqlets | cwm_fwd | cwm_rev | TOMTOM_match | TOMTOM_qval | TOMTOM_match_logo |
|---|---|---|---|---|---|---|
| pos_patterns.pattern_0 | 2213 | 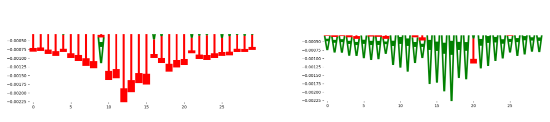 | | ZNF384_MA1125.1 | 0.028745 | 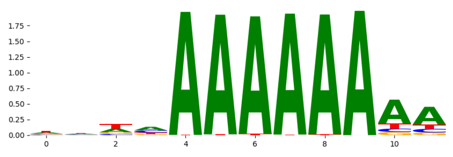 |
| pos_patterns.pattern_1 | 1516 | 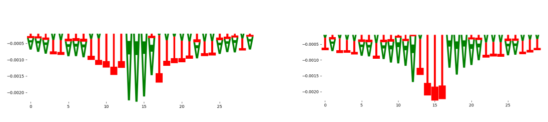 | | DNASE_2 | 1.000000 | 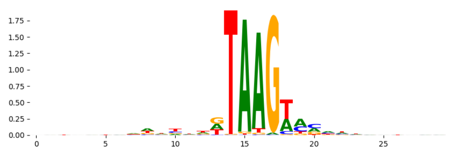 |
| pos_patterns.pattern_2 | 1056 | 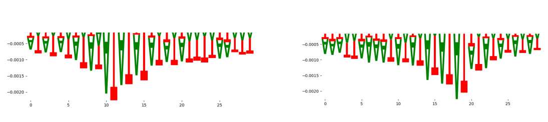 | | FOXB1_forkhead_2 | 0.169313 | 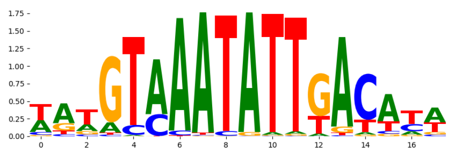 |
| pos_patterns.pattern_3 | 872 | 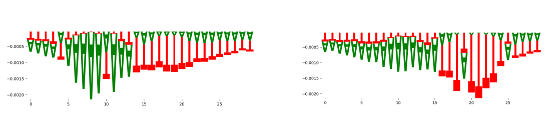 | | ZNF384_MA1125.1 | 0.026828 | 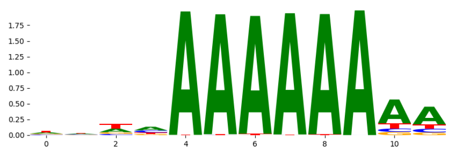 |
| pos_patterns.pattern_4 | 836 | 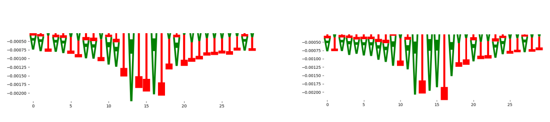 | | DNASE_2 | 0.049511 | 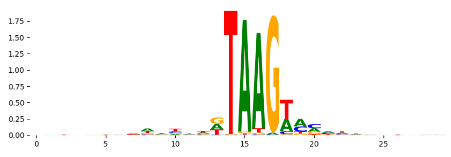 |
| pos_patterns.pattern_5 | 547 | 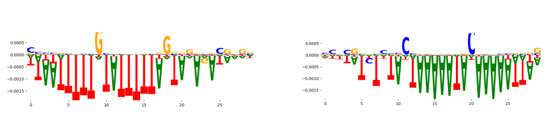 | | MEF2B_HUMAN.H11MO.0.A | 0.281251 | 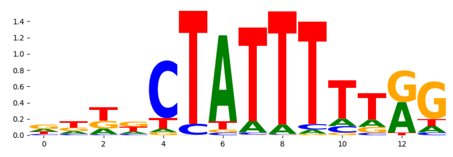 |
| pos_patterns.pattern_6 | 324 | 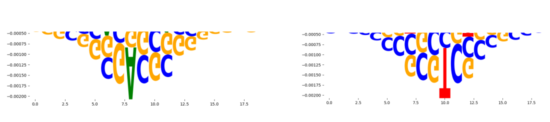 | | SP2_HUMAN.H11MO.0.A | 0.010986 | 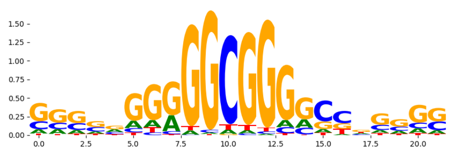 |
| pos_patterns.pattern_7 | 297 | 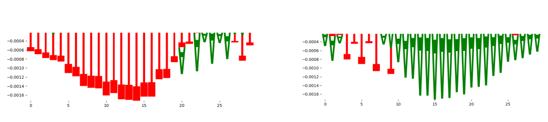 | | PRDM6_HUMAN.H11MO.0.C | 0.038345 | 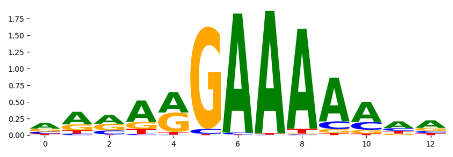 |
| pos_patterns.pattern_8 | 207 | 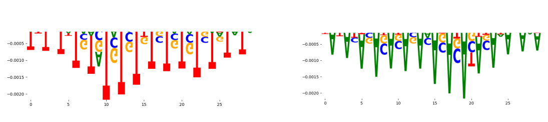 | | BRAC_MOUSE.H11MO.0.B | 1.000000 | 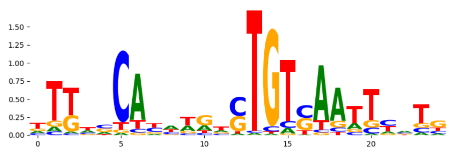 |
| pos_patterns.pattern_9 | 94 | 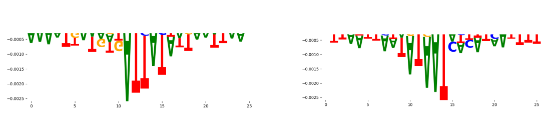 | | ZN264_HUMAN.H11MO.0.C | 1.000000 | 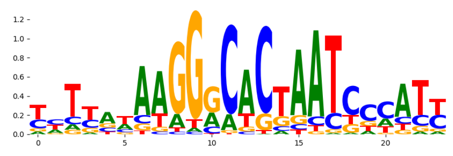 |
| pos_patterns.pattern_10 | 82 | 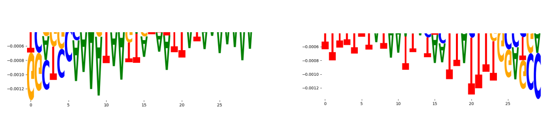 | | ZN770_HUMAN.H11MO.0.C | 0.512755 | 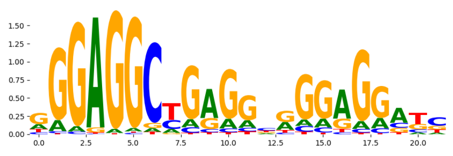 |
| pos_patterns.pattern_11 | 54 | 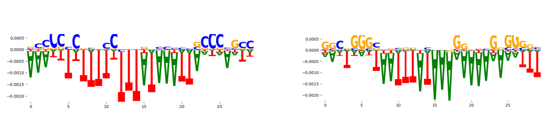 | | ZNF384_MA1125.1 | 1.000000 | 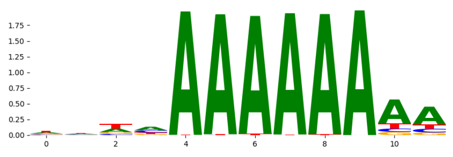 |
| pos_patterns.pattern_12 | 22 | 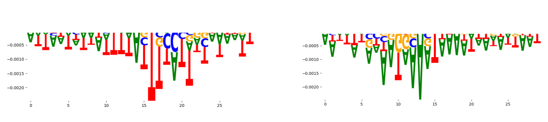 | | IRF3_HUMAN.H11MO.0.B | 0.533869 | 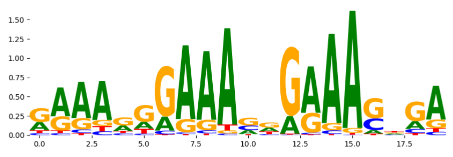 |
| neg_patterns.pattern_0 | 4087 | 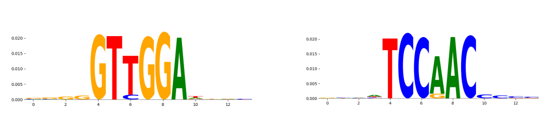 | | MYBA_MOUSE.H11MO.0.C | 1.000000 | 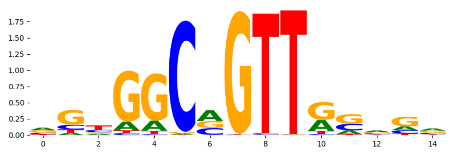 |
| neg_patterns.pattern_1 | 37 | 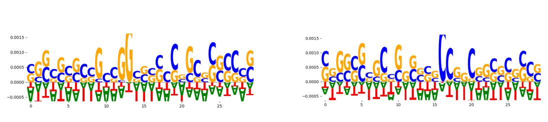 | | ZNF435_C2H2_1 | 0.354321 | 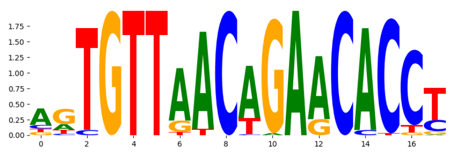 |
| neg_patterns.pattern_2 | 20 | 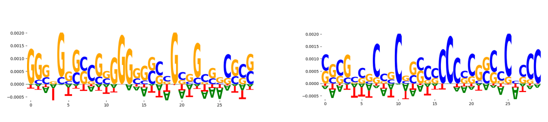 | | FOXH1_MA0479.1 | 1.000000 | 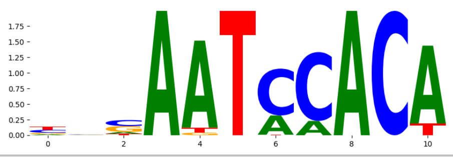 |
