# Supplementary material for: ChromBPNet: bias factorized, base-resolution deep learning models of chromatin accessibility reveal cis-regulatory sequence syntax, transcription factor footprints and regulatory variants
Source: bioRxiv. 2025 Jan 8:2024.12.25.630221. Preprint. [Version 2] doi: 10.1101/2024.12.25.630221 (PMC11741299; doi:10.1101/2024.12.25.630221)
Supplement: Supplement 5 [file media-5.zip › supplementary_files_4/2_naked_dna_DNASE_bias_model/fig4d_naked_dna_DNASE_raw_bias_model_profile_modisco.pdf]

pattern	num_seqlets	cwm_fwd	cwm_rev	TOMTOM_match	TOMTOM_qval	TOMTOM_match_logo
pos_patterns.pattern_0	2214			ZNF384_MA1125.1	7.792200e-02	
pos_patterns.pattern_1	1700			SOX8_HMG_8	6.104490e-01	
pos_patterns.pattern_2	1368			Ar.mouse_nuclearreceptor_1	1.000000e+00	
pos_patterns.pattern_3	1048			IKZF1_HUMAN.H11MO.0.C	1.000000e+00	
pos_patterns.pattern_4	846			TN5_6	4.483670e-23	
pos_patterns.pattern_5	793			RARG_HUMAN.H11MO.0.B	9.358020e-01	
pos_patterns.pattern_6	771			ZN770_HUMAN.H11MO.0.C	8.241370e-09	
pos_patterns.pattern_7	584			DNASE_5	4.886980e-04	
pos_patterns.pattern_8	566			ZN816_HUMAN.H11MO.0.C	1.000000e+00	
pos_patterns.pattern_9	474			MEF2B_HUMAN.H11MO.0.A	2.864880e-01	
pos_patterns.pattern_10	444			DNASE_2	3.083670e-01	
pos_patterns.pattern_11	404			ONECUT3_CUT_1	2.661330e-01	
pos_patterns.pattern_12	404			DNASE_2	4.653150e-01	
pos_patterns.pattern_13	318			RREB1_MA0073.1	1.000000e+00	
pos_patterns.pattern_14	276			RELB_MA1117.1	1.000000e+00	
pos_patterns.pattern_15	211			TN5_6	2.305050e-04	
pos_patterns.pattern_16	207			ZN770_HUMAN.H11MO.0.C	1.732780e-13	
pos_patterns.pattern_17	170			IRF3_HUMAN.H11MO.0.B	2.484470e-02	
pos_patterns.pattern_18	132			MEF2B_HUMAN.H11MO.0.A	2.757370e-01	
pos_patterns.pattern_19	115			E2F7_E2F_1	1.462340e-01	
pos_patterns.pattern_20	86			SP5_MOUSE.H11MO.0.C	7.817040e-08	
pos_patterns.pattern_21	69			FOXC1_forkhead_1	1.533760e-01	
pos_patterns.pattern_22	29			ZNF384_MA1125.1	1.000000e+00	
pos_patterns.pattern_23	20			ZN770_HUMAN.H11MO.0.C	1.569570e-02	
