# Supplementary material for: ChromBPNet: bias factorized, base-resolution deep learning models of chromatin accessibility reveal cis-regulatory sequence syntax, transcription factor footprints and regulatory variants
Source: bioRxiv. 2025 Jan 8:2024.12.25.630221. Preprint. [Version 2] doi: 10.1101/2024.12.25.630221 (PMC11741299; doi:10.1101/2024.12.25.630221)
Supplement: Supplement 5 [file media-5.zip › supplementary_files_4/4_ATAC_bpnet_w_hepg2_bias/fig4g_gm12878_ATAC_raw_bpnet_w_heg2_bias_correction_counts_modisco.pdf]

pattern	num_seqlets	cwm_fwd	cwm_rev	TOMTOM_match	TOMTOM_qval	TOMTOM_match_logo
pos_patterns.pattern_0	5384			IRF1_MOUSE.H11MO.0.A	2.976030e-03	
pos_patterns.pattern_1	3024			ELF5_HUMAN.H11MO.0.A	2.347390e-05	
pos_patterns.pattern_2	2882			CTCF_MA0139.1	1.703230e-15	
pos_patterns.pattern_3	2438			FOS+JUN_MA0099.3	1.978980e-03	
pos_patterns.pattern_4	1920			RUNX3_HUMAN.H11MO.0.A	7.953870e-02	
pos_patterns.pattern_5	1897			NFKB1_HUMAN.H11MO.1.B	9.715450e-07	
pos_patterns.pattern_6	961			IRF4_HUMAN.H11MO.0.A	2.231930e-05	
pos_patterns.pattern_7	713			KLF12_HUMAN.H11MO.0.C	1.858470e-04	
pos_patterns.pattern_8	422			NFYC_HUMAN.H11MO.0.A	1.439830e-03	
pos_patterns.pattern_9	320			NRF1_HUMAN.H11MO.0.A	2.003370e-07	
pos_patterns.pattern_10	255			REL_MA0101.1	9.350560e-01	
pos_patterns.pattern_11	241			CREB1_HUMAN.H11MO.0.A	1.915050e-05	
pos_patterns.pattern_12	217			POU5F1_MA1115.1	3.560680e-03	
pos_patterns.pattern_13	208			EBF1_EBF_1	4.518600e-06	
pos_patterns.pattern_14	194			ETS1_HUMAN.H11MO.0.A	1.303170e-02	
pos_patterns.pattern_15	169			ZN143_MOUSE.H11MO.0.A	8.036540e-11	
pos_patterns.pattern_16	155			SPI1_ETS_1	1.712780e-02	
pos_patterns.pattern_17	134			PAX5_MOUSE.H11MO.0.A	4.597220e-14	
pos_patterns.pattern_18	94			TYY1_HUMAN.H11MO.0.A	2.384500e-06	
pos_patterns.pattern_19	93			KAISO_HUMAN.H11MO.0.A	5.783060e-08	
pos_patterns.pattern_20	63			PAX5_MOUSE.H11MO.0.A	9.624550e-06	
pos_patterns.pattern_21	42			RFX5_RFX_2	5.467310e-06	
pos_patterns.pattern_22	36			MEF2B_MA0660.1	4.334320e-05	
pos_patterns.pattern_23	34			ARNTL_bHLH_1	1.825530e-04	
