# Supplementary material for: ChromBPNet: bias factorized, base-resolution deep learning models of chromatin accessibility reveal cis-regulatory sequence syntax, transcription factor footprints and regulatory variants
Source: bioRxiv. 2025 Jan 8:2024.12.25.630221. Preprint. [Version 2] doi: 10.1101/2024.12.25.630221 (PMC11741299; doi:10.1101/2024.12.25.630221)
Supplement: Supplement 5 [file media-5.zip › supplementary_files_4/4_ATAC_bpnet_w_hepg2_bias/fig4h_gm12878_ATAC_raw_bpnet_w_hepg2_bias_correction_profile_modisco.pdf]

pattern	num_seqlets	cwm_fwd	cwm_rev	TOMTOM_match	TOMTOM_qval	TOMTOM_match_logo
pos_patterns.pattern_0	6200			IRF1_MOUSE.H11MO.0.A	3.150150e-03	
pos_patterns.pattern_1	3999			CTCF_MA0139.1	7.824870e-16	
pos_patterns.pattern_2	2744			SPIC_ETS_1	2.065390e-04	
pos_patterns.pattern_3	2579			JDP2_MA0655.1	2.878720e-03	
pos_patterns.pattern_4	1969			RUNX3_HUMAN.H11MO.0.A	1.026170e-01	
pos_patterns.pattern_5	1527			NFKB1_HUMAN.H11MO.1.B	4.285830e-06	
pos_patterns.pattern_6	1154			KLF12_HUMAN.H11MO.0.C	1.531090e-04	
pos_patterns.pattern_7	990			NFYC_HUMAN.H11MO.0.A	1.764620e-04	
pos_patterns.pattern_8	739			IRF4_HUMAN.H11MO.0.A	1.152220e-05	
pos_patterns.pattern_9	566			SPIB_MA0081.1	1.327240e-01	
pos_patterns.pattern_10	558			FOSL2+JUN_MA1131.1	2.591580e-07	
pos_patterns.pattern_11	412			Pou2f2.mouse_POU_2	5.607450e-03	
pos_patterns.pattern_12	398			BATF_HUMAN.H11MO.0.A	2.301610e-10	
pos_patterns.pattern_13	347			NRF1_MOUSE.H11MO.0.A	1.509740e-06	
pos_patterns.pattern_14	224			TYY1_HUMAN.H11MO.0.A	2.013350e-04	
pos_patterns.pattern_15	210			FOXJ3_HUMAN.H11MO.0.A	7.092900e-02	
pos_patterns.pattern_16	197			ZN143_MOUSE.H11MO.0.A	3.344770e-17	
pos_patterns.pattern_17	119			MITF_HUMAN.H11MO.0.A	2.876540e-05	
pos_patterns.pattern_18	90			PAX2_PAX_1	4.858030e-07	
pos_patterns.pattern_19	87			PAX5_HUMAN.H11MO.0.A	1.344530e-11	
pos_patterns.pattern_20	86			COE1_HUMAN.H11MO.0.A	2.232010e-05	
pos_patterns.pattern_21	81			RELA_MA0107.1	4.932500e-04	
pos_patterns.pattern_22	65			Rfx1_MA0509.1	5.223400e-08	
pos_patterns.pattern_23	61			THAP1_HUMAN.H11MO.0.C	5.516520e-02	
pos_patterns.pattern_24	60			NFKB1_HUMAN.H11MO.1.B	1.733130e-03	
pos_patterns.pattern_25	59			STAT1+STAT2_MA0517.1	2.505030e-05	
pos_patterns.pattern_26	56			KAISO_HUMAN.H11MO.0.A	1.214560e-04	
pos_patterns.pattern_27	37			PRDM1_C2H2_1	3.588410e-01	
pos_patterns.pattern_28	29			BATF+JUN_MA0462.1	3.354280e-02	

pattern	num_seqlets	cwm_fwd	cwm_rev	TOMTOM_match	TOMTOM_qval	TOMTOM_match_logo
pos_patterns.pattern_29	24	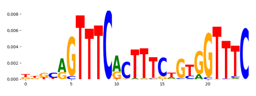	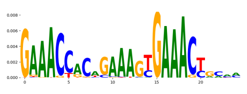	IRF9_HUMAN.H11MO.0.C	1.058150e-02	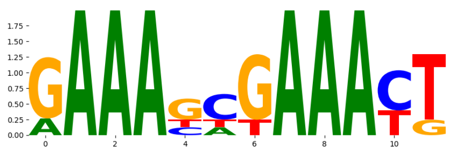
pos_patterns.pattern_30	21	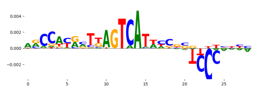	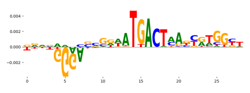	BATF+JUN_MA0462.1	5.976100e-02	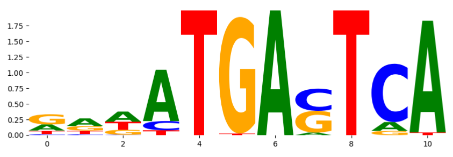
neg_patterns.pattern_0	587	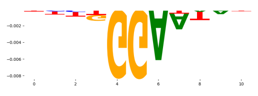	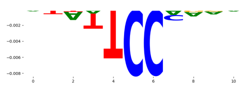	NaN	NaN	
neg_patterns.pattern_1	79	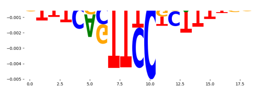	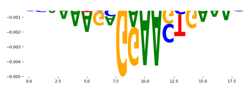	BC11A_HUMAN.H11MO.0.A	9.053620e-04	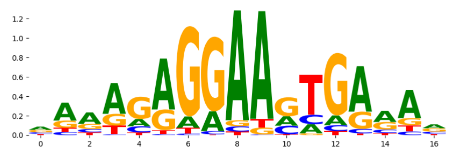
neg_patterns.pattern_2	74	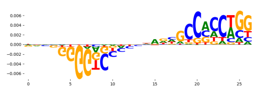	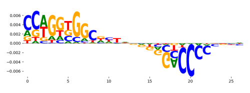	RARA+RXRG_MA1149.1	6.950980e-02	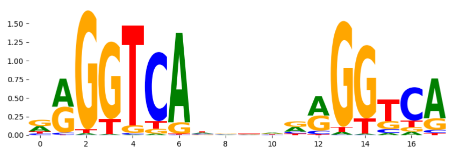
neg_patterns.pattern_3	54	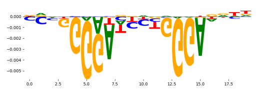	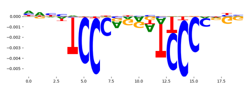	SIX2_HUMAN.H11MO.0.A	1.000000e+00	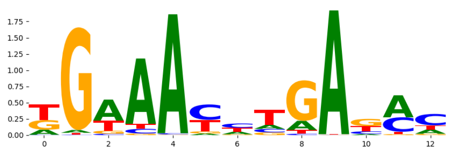
neg_patterns.pattern_4	32	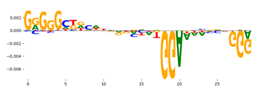	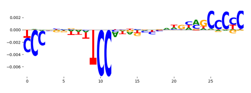	NFATC2_MA0152.1	1.093190e-01	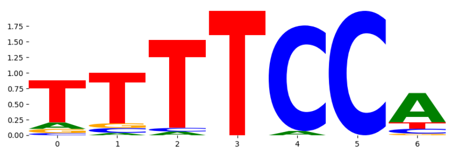
neg_patterns.pattern_5	31	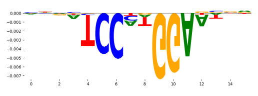	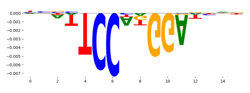	NFATC1_NFAT_3	2.256470e-01	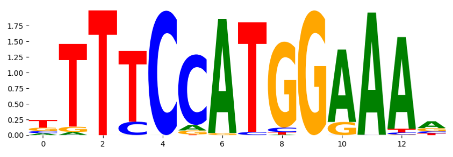
neg_patterns.pattern_6	23	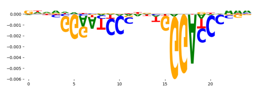	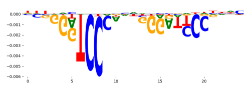	ZIC3_HUMAN.H11MO.0.B	4.980120e-01	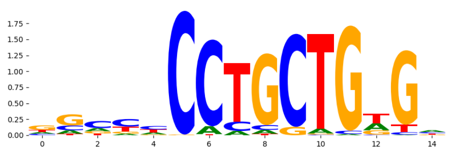
neg_patterns.pattern_7	22	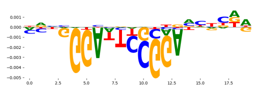	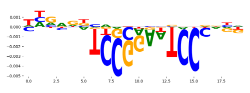	ZFP82_HUMAN.H11MO.0.C	1.000000e+00	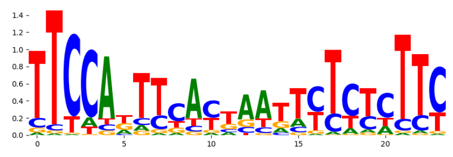
neg_patterns.pattern_8	20	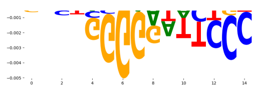	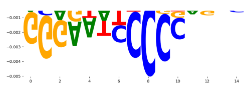	NFKB2_MA0778.1	2.425160e-03	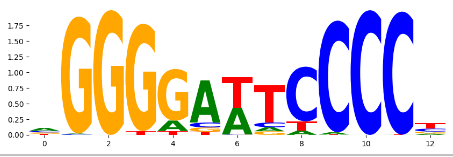
