# Supplementary material for: ChromBPNet: bias factorized, base-resolution deep learning models of chromatin accessibility reveal cis-regulatory sequence syntax, transcription factor footprints and regulatory variants
Source: bioRxiv. 2025 Jan 8:2024.12.25.630221. Preprint. [Version 2] doi: 10.1101/2024.12.25.630221 (PMC11741299; doi:10.1101/2024.12.25.630221)
Supplement: Supplement 5 [file media-5.zip › supplementary_files_4/6_DNASE_bpnet_w_hepg2_bias/fig4l_gm12878_DNASE_raw_bpnet_w_hepg2_bias_correction_profile_modisco.pdf]

pattern	num_seqlets	cwm_fwd	cwm_rev	TOMTOM_match	TOMTOM_qval	TOMTOM_match_logo
pos_patterns.pattern_0	5249			ELF5_HUMAN.H11MO.0.A	9.691420e-05	
pos_patterns.pattern_1	5033			CTCF_MA0139.1	2.194550e-12	
pos_patterns.pattern_2	4134			RUNX3_HUMAN.H11MO.0.A	8.828870e-02	
pos_patterns.pattern_3	3013			STAT1+STAT2_MA0517.1	1.285860e-01	
pos_patterns.pattern_4	2725			JDP2_MA0655.1	2.140440e-03	
pos_patterns.pattern_5	1650			NFKB1_HUMAN.H11MO.1.B	2.103800e-05	
pos_patterns.pattern_6	1550			KLF12_HUMAN.H11MO.0.C	3.349660e-06	
pos_patterns.pattern_7	1273			IRF4_HUMAN.H11MO.0.A	2.337670e-07	
pos_patterns.pattern_8	1070			STAT2_HUMAN.H11MO.0.A	4.737680e-06	
pos_patterns.pattern_9	947			NFYB_HUMAN.H11MO.0.A	2.764350e-04	
pos_patterns.pattern_10	687			NRF1_HUMAN.H11MO.0.A	1.436610e-06	
pos_patterns.pattern_11	551			SP5_MOUSE.H11MO.0.C	2.550590e-07	
pos_patterns.pattern_12	459			POU5F1_MA1115.1	3.140390e-02	
pos_patterns.pattern_13	438			FOSB+JUNB_MA1136.1	3.041810e-05	
pos_patterns.pattern_14	406			ETS1_HUMAN.H11MO.0.A	3.318090e-04	
pos_patterns.pattern_15	316			RFX3_MOUSE.H11MO.0.C	8.473740e-09	
pos_patterns.pattern_16	309			MITF_MA0620.2	9.471860e-06	
pos_patterns.pattern_17	270			SP5_MOUSE.H11MO.0.C	4.327930e-08	
pos_patterns.pattern_18	263			ZN143_MOUSE.H11MO.0.A	2.385520e-19	
pos_patterns.pattern_19	262			EBF1_EBF_1	1.091940e-05	
pos_patterns.pattern_20	214			ZN281_MOUSE.H11MO.0.A	2.328410e-04	
pos_patterns.pattern_21	207			PAX2_PAX_1	1.733700e-09	
pos_patterns.pattern_22	119			ETV6_ETS_1	8.093770e-02	
pos_patterns.pattern_23	111			ZBTB33_MA0527.1	1.473020e-04	
pos_patterns.pattern_24	96			HNF1A_HUMAN.H11MO.0.C	8.929480e-06	
pos_patterns.pattern_25	74			ZN143_HUMAN.H11MO.0.A	1.830400e-07	
pos_patterns.pattern_26	70			PAX2_PAX_1	4.364550e-04	
pos_patterns.pattern_27	68			IKZF1_HUMAN.H11MO.0.C	8.640450e-01	
pos_patterns.pattern_28	68			SP1_MOUSE.H11MO.0.A	1.626670e-05	

pattern	num_seqlets	cwm_fwd	cwm_rev	TOMTOM_match	TOMTOM_qval	TOMTOM_match_logo
pos_patterns.pattern_29	52			BATF+JUN_MA0462.1	1.290240e-03	
pos_patterns.pattern_30	42			BATF+JUN_MA0462.1	7.203670e-03	
pos_patterns.pattern_31	39			ZN549_HUMAN.H11MO.0.C	1.000000e+00	
pos_patterns.pattern_32	38			Gfi1b_MA0483.1	1.000000e+00	
pos_patterns.pattern_33	34			TFCP2_CP2_2	1.000000e+00	
pos_patterns.pattern_34	33			MEF2D_MA0773.1	4.503870e-02	
pos_patterns.pattern_35	26			NFIA_HUMAN.H11MO.0.C	3.381870e-04	
pos_patterns.pattern_36	22			DNASE_5	2.572300e-04	
neg_patterns.pattern_0	112			TEAD3_MA0808.1	8.774540e-01	
neg_patterns.pattern_1	90			ETS1_HUMAN.H11MO.0.A	8.614510e-06	
neg_patterns.pattern_2	75			PROP1_MA0715.1	1.022970e-03	
neg_patterns.pattern_3	69			IRF1_MOUSE.H11MO.0.A	4.594720e-05	
neg_patterns.pattern_4	33			ZFX_MOUSE.H11MO.0.B	2.394470e-01	
neg_patterns.pattern_5	28			NFKB2_HUMAN.H11MO.0.B	9.446830e-03	
neg_patterns.pattern_6	25			SP4_HUMAN.H11MO.0.A	3.061250e-04	
neg_patterns.pattern_7	24			STA5A_HUMAN.H11MO.0.A	7.568250e-02	
neg_patterns.pattern_8	22			JUNB_MOUSE.H11MO.0.A	8.143200e-03	
neg_patterns.pattern_9	20			RUNX3_HUMAN.H11MO.0.A	9.070560e-02	
