# Supplementary material for: ChromBPNet: bias factorized, base-resolution deep learning models of chromatin accessibility reveal cis-regulatory sequence syntax, transcription factor footprints and regulatory variants
Source: bioRxiv. 2025 Jan 8:2024.12.25.630221. Preprint. [Version 2] doi: 10.1101/2024.12.25.630221 (PMC11741299; doi:10.1101/2024.12.25.630221)
Supplement: Supplement 5 [file media-5.zip › supplementary_files_4/6_DNASE_bpnet_w_hepg2_bias/fig4k_gm12878_DNASE_raw_bpnet_w_heg2_bias_correction_counts_modisco.pdf]

pattern	num_seqlets	cwm_fwd	cwm_rev	TOMTOM_match	TOMTOM_qval	TOMTOM_match_logo
pos_patterns.pattern_0	4730			ELF5_HUMAN.H11MO.0.A	1.547520e-05	
pos_patterns.pattern_1	3426			CTCF_MA0139.1	1.524140e-11	
pos_patterns.pattern_2	3347			IRF4_HUMAN.H11MO.0.A	1.119210e-02	
pos_patterns.pattern_3	3026			RUNX3_HUMAN.H11MO.0.A	9.365550e-02	
pos_patterns.pattern_4	2229			NFKB1_HUMAN.H11MO.1.B	7.809360e-07	
pos_patterns.pattern_5	2171			JDP2_MA0655.1	3.396910e-03	
pos_patterns.pattern_6	1777			IRF4_HUMAN.H11MO.0.A	4.690170e-08	
pos_patterns.pattern_7	679			SP1_HUMAN.H11MO.0.A	3.160590e-05	
pos_patterns.pattern_8	614			BATF3_HUMAN.H11MO.0.B	1.967330e-07	
pos_patterns.pattern_9	506			ETS1_HUMAN.H11MO.0.A	8.071990e-02	
pos_patterns.pattern_10	418			NRF1_HUMAN.H11MO.0.A	3.575780e-08	
pos_patterns.pattern_11	324			NFYC_HUMAN.H11MO.0.A	1.341650e-03	
pos_patterns.pattern_12	301			POU5F1_MA1115.1	3.380980e-03	
pos_patterns.pattern_13	258			ZN143_MOUSE.H11MO.0.A	3.858330e-15	
pos_patterns.pattern_14	238			XBP1_bZIP_1	3.835180e-04	
pos_patterns.pattern_15	219			EBF1_EBF_1	8.724300e-06	
pos_patterns.pattern_16	176			SPI1_ETS_1	2.157080e-02	
pos_patterns.pattern_17	156			PAX2_PAX_1	1.332250e-09	
pos_patterns.pattern_18	97			HNF1B_HUMAN.H11MO.0.A	1.083950e-05	
pos_patterns.pattern_19	92			ZBTB33_MA0527.1	6.556330e-06	
pos_patterns.pattern_20	66			TYY1_HUMAN.H11MO.0.A	1.018530e-04	
pos_patterns.pattern_21	55			PAX2_PAX_1	2.307710e-05	
pos_patterns.pattern_22	54			BATF+JUN_MA0462.1	2.162940e-03	
pos_patterns.pattern_23	47			Rfx1_MA0509.1	1.501770e-08	
pos_patterns.pattern_24	40			ZNF524_C2H2_1	5.388540e-02	
pos_patterns.pattern_25	39			MEF2D_HUMAN.H11MO.0.A	2.220990e-04	
pos_patterns.pattern_26	35			MAZ_HUMAN.H11MO.0.A	3.010770e-05	
pos_patterns.pattern_27	27			RORA_nuclearreceptor_1	1.000000e+00	
pos_patterns.pattern_28	27			SP2_MA0516.1	6.836270e-02	

pattern	num_seqlets	cwm_fwd	cwm_rev	TOMTOM_match	TOMTOM_qval	TOMTOM_match_logo
pos_patterns.pattern_29	24	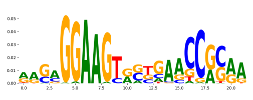	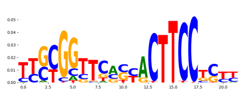	RUNX3_HUMAN.H11MO.0.A	1.214550e-01	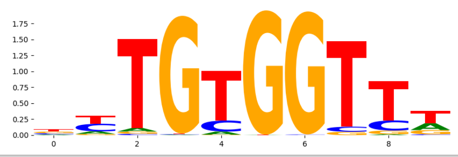
