# Supplementary material for: ChromBPNet: bias factorized, base-resolution deep learning models of chromatin accessibility reveal cis-regulatory sequence syntax, transcription factor footprints and regulatory variants
Source: bioRxiv. 2025 Jan 8:2024.12.25.630221. Preprint. [Version 2] doi: 10.1101/2024.12.25.630221 (PMC11741299; doi:10.1101/2024.12.25.630221)
Supplement: Supplement 5 [file media-5.zip › supplementary_files_4/5_DNASE_bpnet_w_naked_dna_bias/fig4j_gm12878_DNASE_raw_bpnet_w_naked_dna_bias_correction_profile_modisco.pdf]

pattern	num_seqlets	cwm_fwd	cwm_rev	TOMTOM_match	TOMTOM_qval	TOMTOM_match_logo
pos_patterns.pattern_0	4270			CTCF_MA0139.1	9.019020e-12	
pos_patterns.pattern_1	3318			IRF4_MOUSE.H11MO.0.A	5.473920e-05	
pos_patterns.pattern_2	2783			ETS1_HUMAN.H11MO.0.A	8.936240e-04	
pos_patterns.pattern_3	1971			ATF3_MOUSE.H11MO.0.A	3.759360e-03	
pos_patterns.pattern_4	1584			RUNX3_HUMAN.H11MO.0.A	2.269830e-03	
pos_patterns.pattern_5	1058			KLF12_HUMAN.H11MO.0.C	8.237540e-05	
pos_patterns.pattern_6	1025			RELB_HUMAN.H11MO.0.C	5.611040e-07	
pos_patterns.pattern_7	816			ZN667_HUMAN.H11MO.0.C	1.000000e+00	
pos_patterns.pattern_8	770			NFYB_HUMAN.H11MO.0.A	6.101990e-04	
pos_patterns.pattern_9	724			ZNF384_MA1125.1	6.522740e-03	
pos_patterns.pattern_10	717			ZNF384_MA1125.1	8.926680e-02	
pos_patterns.pattern_11	590			PRDM6_HUMAN.H11MO.0.C	9.864940e-02	
pos_patterns.pattern_12	579			NRF1_MOUSE.H11MO.0.A	4.311620e-08	
pos_patterns.pattern_13	529			TBX1_TBX_1	7.227480e-01	
pos_patterns.pattern_14	525			SP5_MOUSE.H11MO.0.C	1.752400e-07	
pos_patterns.pattern_15	429			BATF3_HUMAN.H11MO.0.B	5.567150e-06	
pos_patterns.pattern_16	391			ZNF384_MA1125.1	6.292510e-02	
pos_patterns.pattern_17	317			TN5_6	1.462770e-20	
pos_patterns.pattern_18	311			FOSB+JUNB_MA1136.1	2.066430e-05	
pos_patterns.pattern_19	287			EBF1_EBF_1	1.139310e-05	
pos_patterns.pattern_20	277			RREB1_MA0073.1	1.000000e+00	
pos_patterns.pattern_21	244			ZN143_MOUSE.H11MO.0.A	5.324780e-17	
pos_patterns.pattern_22	229			Pou2f2.mouse_POU_2	4.285970e-03	
pos_patterns.pattern_23	222			SP5_MOUSE.H11MO.0.C	5.818330e-07	
pos_patterns.pattern_24	196			PAX5_MA0014.3	1.000000e+00	
pos_patterns.pattern_25	178			ARNTL_bHLH_1	1.914400e-05	
pos_patterns.pattern_26	149			MEF2B_HUMAN.H11MO.0.A	2.436510e-01	
pos_patterns.pattern_27	133			PAX1_MA0779.1	5.781960e-05	
pos_patterns.pattern_28	108			DNASE_5	1.911520e-03	

pattern	num_seqlets	cwm_fwd	cwm_rev	TOMTOM_match	TOMTOM_qval	TOMTOM_match_logo
pos_patterns.pattern_29	104			IRF3_HUMAN.H11MO.0.B	1.353080e-02	
pos_patterns.pattern_30	91			CRX_HUMAN.H11MO.0.B	1.000000e+00	
pos_patterns.pattern_31	87			SP1_HUMAN.H11MO.0.A	7.849410e-10	
pos_patterns.pattern_32	72			THA_HUMAN.H11MO.0.C	1.000000e+00	
pos_patterns.pattern_33	71			RUNX1_MOUSE.H11MO.0.A	1.145040e-02	
pos_patterns.pattern_34	68			ZBTB33_MA0527.1	7.716610e-05	
pos_patterns.pattern_35	52			THA11_HUMAN.H11MO.0.B	6.607040e-06	
pos_patterns.pattern_36	50			Six3_MA0631.1	1.000000e+00	
pos_patterns.pattern_37	46			RUNX3_MOUSE.H11MO.0.A	5.178750e-03	
pos_patterns.pattern_38	44			ZN121_HUMAN.H11MO.0.C	3.891050e-03	
pos_patterns.pattern_39	39			RFX5_RFX_2	1.530100e-08	
pos_patterns.pattern_40	26			CTCFL_MA1102.1	1.000000e+00	
pos_patterns.pattern_41	26			DNASE_2	2.725410e-01	
pos_patterns.pattern_42	24			ETV6_MOUSE.H11MO.0.C	1.638190e-01	
pos_patterns.pattern_43	21			FOXB1_forkhead_2	6.569020e-01	
pos_patterns.pattern_44	20			PAX6_PAX_1	4.642670e-04	
neg_patterns.pattern_0	108			ERG_HUMAN.H11MO.0.A	5.002480e-06	
neg_patterns.pattern_1	83			IRF1_MOUSE.H11MO.0.A	8.981530e-07	
neg_patterns.pattern_2	59			CTCF_MA0139.1	5.809740e-10	
neg_patterns.pattern_3	59			Egr1.mouse_C2H2_1	6.336640e-02	
neg_patterns.pattern_4	52			FOSL1_MA0477.1	7.326350e-06	
neg_patterns.pattern_5	49			ZNF384_MA1125.1	5.278780e-01	
neg_patterns.pattern_6	49			ZNF384_MA1125.1	1.180350e-02	
neg_patterns.pattern_7	41			PROP1_homeodomain_2	2.710320e-01	
neg_patterns.pattern_8	34			NFKB1_HUMAN.H11MO.1.B	4.702380e-05	
neg_patterns.pattern_9	32			TEAD4_MOUSE.H11MO.0.A	1.000000e+00	
neg_patterns.pattern_10	22			MYB_MOUSE.H11MO.0.A	6.650920e-02	
