# Supplementary material for: ChromBPNet: bias factorized, base-resolution deep learning models of chromatin accessibility reveal cis-regulatory sequence syntax, transcription factor footprints and regulatory variants
Source: bioRxiv. 2025 Jan 8:2024.12.25.630221. Preprint. [Version 2] doi: 10.1101/2024.12.25.630221 (PMC11741299; doi:10.1101/2024.12.25.630221)
Supplement: Supplement 5 [file media-5.zip › supplementary_files_4/5_DNASE_bpnet_w_naked_dna_bias/fig4i_gm12878_DNASE_raw_bpnet_w_naked_dna_bias_correction_counts_modisco.pdf]

pattern	num_seqlets	cwm_fwd	cwm_rev	TOMTOM_match	TOMTOM_qval	TOMTOM_match_logo
pos_patterns.pattern_0	5051			IRF1_MOUSE.H11MO.0.A	3.028240e-04	
pos_patterns.pattern_1	4705			ELF5_HUMAN.H11MO.0.A	2.137510e-05	
pos_patterns.pattern_2	3938			CTCF_MA0139.1	3.953770e-12	
pos_patterns.pattern_3	2445			ATF3_MOUSE.H11MO.0.A	4.622950e-03	
pos_patterns.pattern_4	2227			RUNX3_HUMAN.H11MO.0.A	1.765460e-02	
pos_patterns.pattern_5	1713			NFKB1_HUMAN.H11MO.1.B	3.333090e-07	
pos_patterns.pattern_6	1238			KLF12_HUMAN.H11MO.0.C	2.043950e-05	
pos_patterns.pattern_7	737			NRF1_MA0506.1	2.261740e-06	
pos_patterns.pattern_8	608			NFYB_HUMAN.H11MO.0.A	7.535850e-03	
pos_patterns.pattern_9	600			ATF1_MOUSE.H11MO.0.B	1.557860e-03	
pos_patterns.pattern_10	475			POU5F1_MA1115.1	3.272310e-03	
pos_patterns.pattern_11	406			ETS1_HUMAN.H11MO.0.A	2.050190e-03	
pos_patterns.pattern_12	370			COE1_MOUSE.H11MO.0.A	3.416240e-05	
pos_patterns.pattern_13	362			ZN143_MOUSE.H11MO.0.A	1.135250e-19	
pos_patterns.pattern_14	300			NFKB1_HUMAN.H11MO.1.B	6.442470e-03	
pos_patterns.pattern_15	149			PAX1_MA0779.1	1.031160e-07	
pos_patterns.pattern_16	134			REL_MA0101.1	4.615740e-01	
pos_patterns.pattern_17	104			SPIB_ETS_1	3.048380e-02	
pos_patterns.pattern_18	104			ZBTB33_MA0527.1	6.383250e-05	
pos_patterns.pattern_19	64			MEF2D_MOUSE.H11MO.0.A	4.916110e-05	
pos_patterns.pattern_20	44			IRF4_IRF_1	1.493280e-02	
pos_patterns.pattern_21	26			RUNX3_RUNX_1	4.937990e-02	
pos_patterns.pattern_22	23			IRF7_HUMAN.H11MO.0.C	2.574340e-05	
pos_patterns.pattern_23	23			Rfx1_MA0509.1	2.090690e-07	
pos_patterns.pattern_24	23			TYY1_HUMAN.H11MO.0.A	9.047930e-05	
