# Supplementary material for: ChromBPNet: bias factorized, base-resolution deep learning models of chromatin accessibility reveal cis-regulatory sequence syntax, transcription factor footprints and regulatory variants
Source: bioRxiv. 2025 Jan 8:2024.12.25.630221. Preprint. [Version 2] doi: 10.1101/2024.12.25.630221 (PMC11741299; doi:10.1101/2024.12.25.630221)
Supplement: Supplement 5 [file media-5.zip › supplementary_files_4/3_ATAC_bpnet_w_naked_dna_bias/fig4e_gm12878_ATAC_raw_bpnet_w_naked_dna_bias_correction_counts_modisco.pdf]

pattern	num_seqlets	cwm_fwd	cwm_rev	TOMTOM_match	TOMTOM_qval	TOMTOM_match_logo
pos_patterns.pattern_0	3371			CTCF_MA0139.1	3.367020e-15	
pos_patterns.pattern_1	3167			ELF5_HUMAN.H11MO.0.A	1.709640e-05	
pos_patterns.pattern_2	2828			IRF4_MOUSE.H11MO.0.A	4.495630e-02	
pos_patterns.pattern_3	2645			FOSL2+JUN_MA1130.1	2.656940e-03	
pos_patterns.pattern_4	1796			RUNX1_HUMAN.H11MO.0.A	7.671330e-02	
pos_patterns.pattern_5	1713			IRF1_MOUSE.H11MO.0.A	1.617020e-05	
pos_patterns.pattern_6	1520			NFKB2_HUMAN.H11MO.0.B	5.195800e-06	
pos_patterns.pattern_7	734			SP1_HUMAN.H11MO.0.A	6.772420e-05	
pos_patterns.pattern_8	353			NFYB_HUMAN.H11MO.0.A	1.160880e-02	
pos_patterns.pattern_9	353			POU5F1_MA1115.1	4.286820e-03	
pos_patterns.pattern_10	352			NFKB1_HUMAN.H11MO.1.B	8.951890e-03	
pos_patterns.pattern_11	336			BATF_HUMAN.H11MO.0.A	1.347630e-07	
pos_patterns.pattern_12	300			NRF1_HUMAN.H11MO.0.A	1.316570e-06	
pos_patterns.pattern_13	270			REL_MA0101.1	7.345320e-01	
pos_patterns.pattern_14	266			IRF4_HUMAN.H11MO.0.A	1.119760e-03	
pos_patterns.pattern_15	251			HNF1A_HUMAN.H11MO.0.C	1.063610e-07	
pos_patterns.pattern_16	241			PAX5_MOUSE.H11MO.0.A	2.608270e-12	
pos_patterns.pattern_17	217			COE1_HUMAN.H11MO.0.A	2.717330e-07	
pos_patterns.pattern_18	156			SPIB_ETS_1	6.161940e-03	
pos_patterns.pattern_19	143			ATF1_MOUSE.H11MO.0.B	2.089910e-03	
pos_patterns.pattern_20	139			MEF2B_MA0660.1	1.317560e-07	
pos_patterns.pattern_21	114			ZN143_MOUSE.H11MO.0.A	4.571410e-17	
pos_patterns.pattern_22	97			IRF9_IRF_1	2.099090e-03	
pos_patterns.pattern_23	78			FOSL2+JUN_MA1131.1	7.084870e-05	
pos_patterns.pattern_24	74			PAX2_PAX_1	8.249670e-09	
pos_patterns.pattern_25	53			KAISO_HUMAN.H11MO.0.A	1.145200e-07	
pos_patterns.pattern_26	50			TYY1_HUMAN.H11MO.0.A	8.027640e-06	
pos_patterns.pattern_27	34			THA11_HUMAN.H11MO.0.B	1.533700e-06	
pos_patterns.pattern_28	27			PAX5_MOUSE.H11MO.0.A	2.136890e-04	

pattern	num_seqlets	cwm_fwd	cwm_rev	TOMTOM_match	TOMTOM_qval	TOMTOM_match_logo
pos_patterns.pattern_29	24	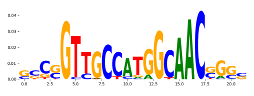	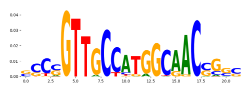	RFX2_HUMAN.H11MO.0.A	2.687770e-09	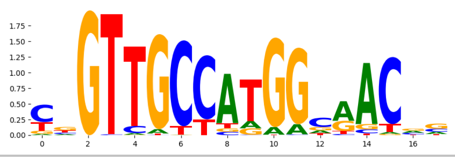
