# Supplementary material for: ChromBPNet: bias factorized, base-resolution deep learning models of chromatin accessibility reveal cis-regulatory sequence syntax, transcription factor footprints and regulatory variants
Source: bioRxiv. 2025 Jan 8:2024.12.25.630221. Preprint. [Version 2] doi: 10.1101/2024.12.25.630221 (PMC11741299; doi:10.1101/2024.12.25.630221)
Supplement: Supplement 5 [file media-5.zip › supplementary_files_4/3_ATAC_bpnet_w_naked_dna_bias/fig4f_gm12878_ATAC_raw_bpnet_w_naked_dna_bias_correction_profile_modisco.pdf]

pattern	num_seqlets	cwm_fwd	cwm_rev	TOMTOM_match	TOMTOM_qval	TOMTOM_match_logo
pos_patterns.pattern_0	4013			CTCF_MA0139.1	6.793640e-17	
pos_patterns.pattern_1	1975			SP2_HUMAN.H11MO.0.A	5.910480e-10	
pos_patterns.pattern_2	1959			SP2_HUMAN.H11MO.0.A	7.311640e-05	
pos_patterns.pattern_3	1601			ATF3_MOUSE.H11MO.0.A	1.871830e-03	
pos_patterns.pattern_4	1175			ELF5_HUMAN.H11MO.0.A	7.760780e-05	
pos_patterns.pattern_5	1101			IRF1_MOUSE.H11MO.0.A	2.154140e-03	
pos_patterns.pattern_6	863			SP2_HUMAN.H11MO.0.A	7.617620e-19	
pos_patterns.pattern_7	787			RELB_HUMAN.H11MO.0.C	6.935320e-06	
pos_patterns.pattern_8	662			KLF5_MOUSE.H11MO.0.A	1.060060e-04	
pos_patterns.pattern_9	624			IRF4_HUMAN.H11MO.0.A	1.261150e-10	
pos_patterns.pattern_10	622			IRF1_HUMAN.H11MO.0.A	1.047540e-06	
pos_patterns.pattern_11	532			RUNX3_HUMAN.H11MO.0.A	4.602700e-01	
pos_patterns.pattern_12	516			SP2_HUMAN.H11MO.0.A	3.356900e-06	
pos_patterns.pattern_13	377			MYOD1_HUMAN.H11MO.0.A	2.843030e-03	
pos_patterns.pattern_14	343			NFYB_HUMAN.H11MO.0.A	3.196650e-07	
pos_patterns.pattern_15	127			BATF_HUMAN.H11MO.0.A	1.678740e-05	
pos_patterns.pattern_16	92			MSX2_homeodomain_1	3.105830e-01	
pos_patterns.pattern_17	88			Pou2f2.mouse_POU_2	1.832920e-02	
pos_patterns.pattern_18	84			NRF1_MA0506.1	3.717790e-04	
pos_patterns.pattern_19	63			ATF2_HUMAN.H11MO.0.B	1.401630e-04	
pos_patterns.pattern_20	60			CTCF_MA0139.1	4.398870e-04	
pos_patterns.pattern_21	54			STAT1_MOUSE.H11MO.0.A	6.753500e-01	
pos_patterns.pattern_22	27			JUN_MA0489.1	1.196910e-02	
pos_patterns.pattern_23	26			TYY1_HUMAN.H11MO.0.A	2.238340e-05	
pos_patterns.pattern_24	25			SPI1_MOUSE.H11MO.0.A	6.180360e-02	
pos_patterns.pattern_25	22			ZNF76_HUMAN.H11MO.0.C	7.640220e-12	
neg_patterns.pattern_0	135			Stat5a+Stat5b_MA0519.1	1.000000e+00	
neg_patterns.pattern_1	63			ZNF384_MA1125.1	5.151300e-01	
neg_patterns.pattern_2	48			IRF4_HUMAN.H11MO.0.A	9.631840e-05	

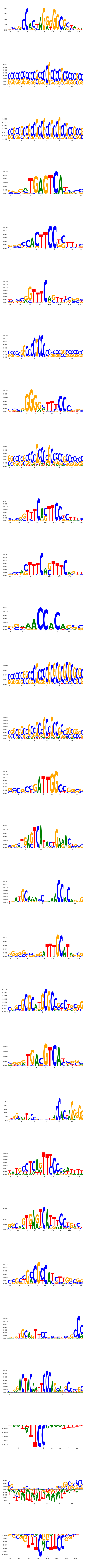
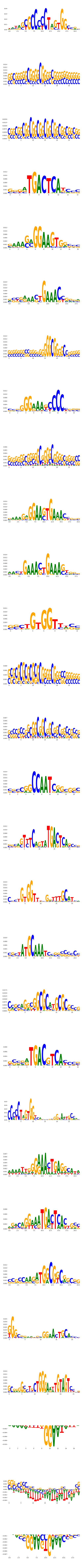
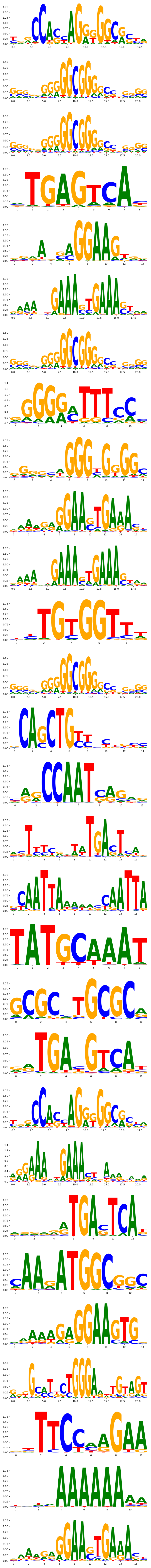

pattern	num_seqlets	cwm_fwd	cwm_rev	TOMTOM_match	TOMTOM_qval	TOMTOM_match_logo
neg_patterns.pattern_3	42	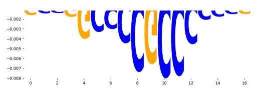	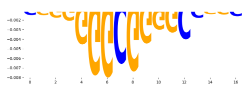	KLF12_HUMAN.H11MO.0.C	2.936490e-04	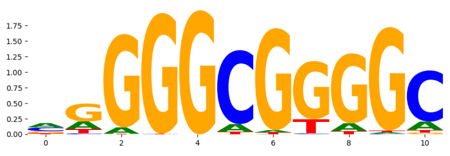
neg_patterns.pattern_4	31	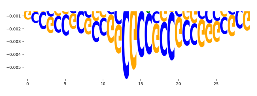	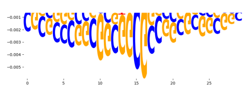	SP2_HUMAN.H11MO.0.A	6.515550e-07	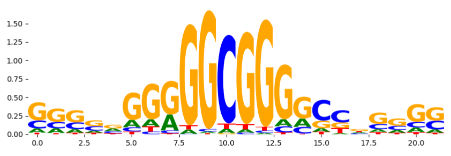
neg_patterns.pattern_5	29	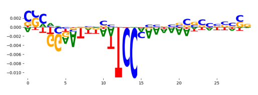	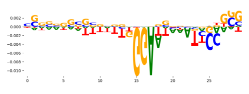	STAT3_MA0144.2	1.178100e-01	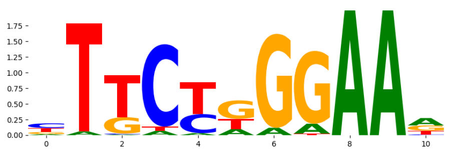
neg_patterns.pattern_6	29	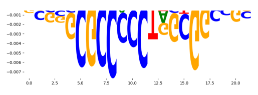	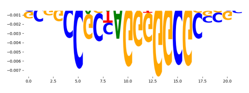	CTCFL_MOUSE.H11MO.0.A	5.807460e-07	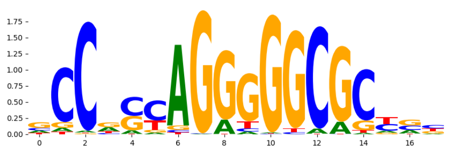
neg_patterns.pattern_7	24	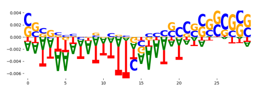	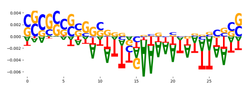	MXI1_HUMAN.H11MO.0.A	4.364370e-01	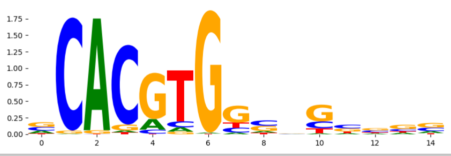
