# Supplementary material for: ChromBPNet: bias factorized, base-resolution deep learning models of chromatin accessibility reveal cis-regulatory sequence syntax, transcription factor footprints and regulatory variants
Source: bioRxiv. 2025 Jan 8:2024.12.25.630221. Preprint. [Version 2] doi: 10.1101/2024.12.25.630221 (PMC11741299; doi:10.1101/2024.12.25.630221)
Supplement: Supplement 5 [file media-5.zip › supplementary_files_4/1_naked_dna_ATAC_bias_model/fig4b_naked_dna_ATAC_raw_bias_model_profile_modisco.pdf]

pattern	num_seqlets	cwm_fwd	cwm_rev	TOMTOM_match	TOMTOM_qval	TOMTOM_match_logo
pos_patterns.pattern_0	13284	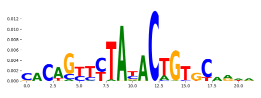	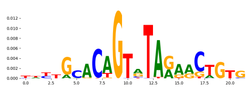	TN5_2	2.585450e-02	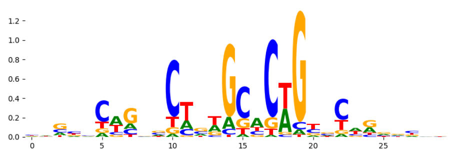
pos_patterns.pattern_1	7275	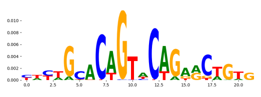	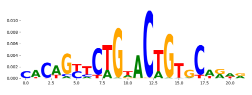	TN5_2	7.139180e-06	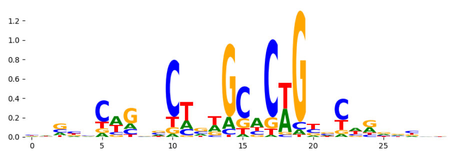
pos_patterns.pattern_2	4538	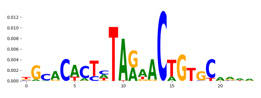	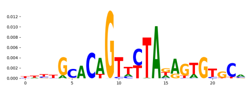	TN5_3	2.477680e-04	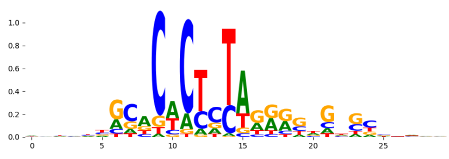
pos_patterns.pattern_3	3923	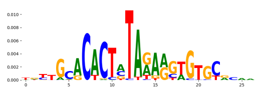	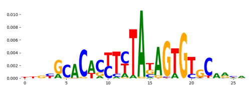	TN5_3	5.278590e-10	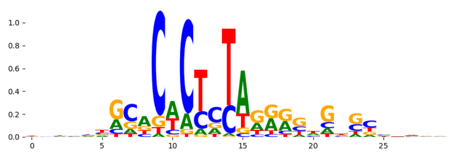
pos_patterns.pattern_4	190	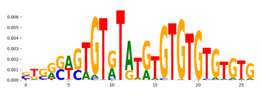	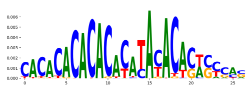	EGR2_HUMAN.H11MO.0.A	1.000000e+00	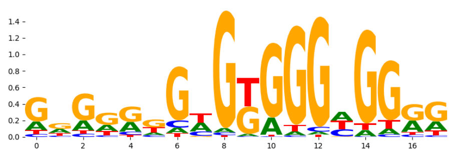
pos_patterns.pattern_5	175	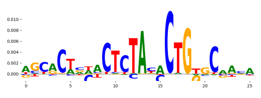	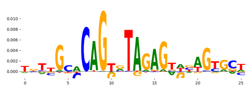	TN5_6	3.491220e-07	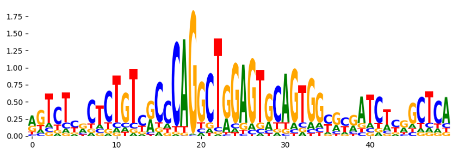
pos_patterns.pattern_6	131	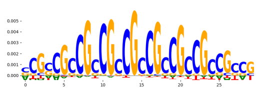	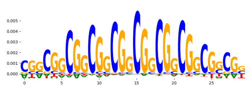	SP2_HUMAN.H11MO.0.A	8.263200e-04	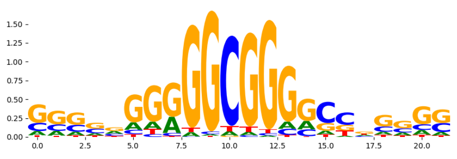
pos_patterns.pattern_7	47	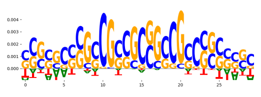	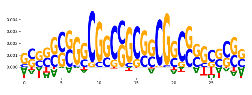	SP2_HUMAN.H11MO.0.A	5.127050e-07	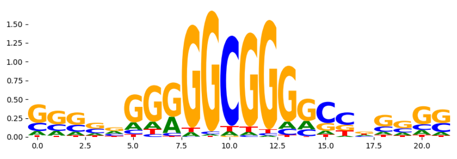
pos_patterns.pattern_8	31	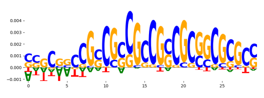	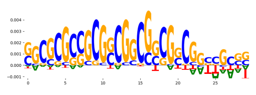	SP2_HUMAN.H11MO.0.A	2.250760e-04	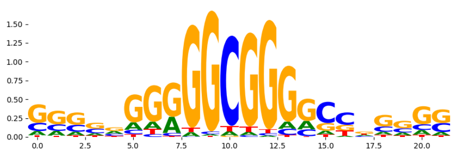
pos_patterns.pattern_9	26	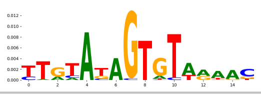	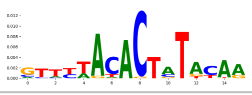	NaN	NaN	
