# Supplementary material for: ChromBPNet: bias factorized, base-resolution deep learning models of chromatin accessibility reveal cis-regulatory sequence syntax, transcription factor footprints and regulatory variants
Source: bioRxiv. 2025 Jan 8:2024.12.25.630221. Preprint. [Version 2] doi: 10.1101/2024.12.25.630221 (PMC11741299; doi:10.1101/2024.12.25.630221)
Supplement: Supplement 5 [file media-5.zip › supplementary_files_4/1_naked_dna_ATAC_bias_model/fig4a_naked_dna_ATAC_raw_bias_model_counts_modisco.pdf]

pattern	num_seqlets	cwm_fwd	cwm_rev	TOMTOM_match	TOMTOM_qval	TOMTOM_match_logo
pos_patterns.pattern_0	2702			SP2_HUMAN.H11MO.0.A	3.291210e-05	
pos_patterns.pattern_1	2581			SP2_HUMAN.H11MO.0.A	5.786870e-11	
pos_patterns.pattern_2	1966			SP2_HUMAN.H11MO.0.A	7.391890e-07	
pos_patterns.pattern_3	1501			SP2_HUMAN.H11MO.0.A	1.611420e-07	
pos_patterns.pattern_4	948			SP2_HUMAN.H11MO.0.A	5.495660e-07	
neg_patterns.pattern_0	489			ELF5_MOUSE.H11MO.0.A	7.347790e-04	
neg_patterns.pattern_1	203			ZBT17_MOUSE.H11MO.0.A	1.915460e-02	
neg_patterns.pattern_2	129			TAF1_HUMAN.H11MO.0.A	3.322220e-01	
neg_patterns.pattern_3	124			SP2_HUMAN.H11MO.0.A	6.445520e-06	
neg_patterns.pattern_4	92			Zfx_MA0146.2	7.398490e-02	
neg_patterns.pattern_5	69			SP1_HUMAN.H11MO.0.A	1.091220e-05	
neg_patterns.pattern_6	65			SP1_HUMAN.H11MO.0.A	1.721450e-04	
neg_patterns.pattern_7	64			SP2_HUMAN.H11MO.0.A	7.595640e-05	
neg_patterns.pattern_8	64			BHA15_HUMAN.H11MO.0.B	3.930840e-01	
neg_patterns.pattern_9	54			SP1_HUMAN.H11MO.0.A	3.405030e-02	
neg_patterns.pattern_10	46			RXRG_MOUSE.H11MO.0.B	1.336440e-01	
neg_patterns.pattern_11	46			TAF1_HUMAN.H11MO.0.A	1.909640e-01	
